# Oral prednisolone for acute otitis media in children: protocol of a pilot randomised, open-label, controlled study (OPAL study)

**DOI:** 10.1186/s40814-018-0337-x

**Published:** 2018-09-10

**Authors:** Respati W. Ranakusuma, Amanda R. McCullough, Eka D. Safitri, Yupitri Pitoyo, Widyaningsih, Christopher B. Del Mar, Elaine M. Beller

**Affiliations:** 1 Centre for Research in Evidence-Based Practice Faculty of Health Sciences and Medicine Bond University, 14 University Drive, Robina, 4226 Queensland Australia; 2 Clinical Epidemiology and Evidence-Based Medicine Unit, Dr. Cipto Mangunkusumo General Hospital – Faculty of Medicine Universitas Indonesia, Diponegoro 71, Jakarta, 10430 Indonesia

**Keywords:** Acute otitis media, Antibiotics, Corticosteroids, Middle ear effusion, Mechanistic sub-study, Tympanometry, Trial protocol

## Abstract

**Background:**

Acute otitis media (AOM) is an acute inflammation of the middle ear commonly found in children, for which antibiotics are frequently prescribed. However, antibiotics are beneficial for only one third of AOM cases, and then, with only modest benefit. Since antibiotic use leads to risk of side effects and resistance, effective alternative treatments are required. Corticosteroids are a candidate because of their anti-inflammatory effects, although evidence of their efficacy and harms is insufficient. Accordingly, we plan a large, rigorous clinical trial to test this. Initially, we will test pre-specified methods and procedures (including the overall process, resources, management, and scientific components) in a pilot study of corticosteroids for AOM, which will inform a future, definitive trial.

**Methods:**

This is a pilot pragmatic, randomised, open-label, single-blind, controlled study of corticosteroids as either monotherapy or an addition to antibiotics in 60 children aged 6 months to 12 years with AOM in two cities (Jakarta and Bekasi) in Indonesia. We will randomise eligible children to prednisolone or control. We will also stratify by disease severity and randomise those with mild AOM to expectant observation plus prednisolone or observation alone and those with severe AOM to prednisolone plus antibiotic or antibiotic alone. Our outcomes are to determine (1) recruitment rates, (2) the success of the study procedures, (3) the ability to measure planned outcomes of the proposed main study, (4) the compliance to study visits and study medication, and (5) verification of the sample size calculation for the main study. We will also assess middle ear effusion using tympanometry as part of a mechanistic sub-study.

**Discussion:**

This study will test all procedures in preparation for the main study, including several potential obstacles and challenges from the perspective of participating physicians, nurses, pharmacists, and the parents of eligible children. This information will be useful for developing strategies to overcome practical and procedural issues. This study may also provide information about the effects of corticosteroids on middle ear effusion in AOM.

**Trial registration:**

Study registry number: ACTRN12618000049279. Name of registry: the Australian New Zealand Clinical Trials Registry (ANZCTR). Date of registration: 16 January 2018.

**Electronic supplementary material:**

The online version of this article (10.1186/s40814-018-0337-x) contains supplementary material, which is available to authorized users.

## Background

Antibiotic resistance is a major global health threat, which is mostly driven by antibiotic prescribing [1]. Antibiotics are commonly prescribed in primary care for acute respiratory infections, including acute otitis media [2–4]. Acute otitis media (AOM) is an inflammation of the middle ear commonly found in children [5]. Approximately 75% of children experience AOM before the age of five [6]. Recurrent AOM, defined as three or more AOM episodes in the past 6 months or four or more episodes in the past 12 months with at least one episode in the past 6 months, occurs in 24% of American children up to aged three [5, 7]. In Europe, 2% of children under six have had three or more episodes of AOM the previous year [6].

In the management of AOM, expectant observation with pain management is commonly recommended for children with mild AOM (e.g. mild ear pain, fever < 39 °C) who can be reliably followed up [5]. Those with severe symptoms, bilateral AOM in young age children, or with perforated ear drums are more likely to benefit from antibiotics [5, 8]. The option of using antibiotics must be balanced against common adverse effects (e.g. vomiting, diarrhoea, or rash) [9, 10]. In Australian general practice, 89% of new AOM cases are managed with antibiotics [11]. Similarly, in Indonesia, our survey study demonstrated about 88% of physicians would prescribe antibiotics for children with mild AOM. Indonesian practice guidelines on the criteria for antibiotic use for AOM are vague [12, 13].

Alternative treatments have been proposed for treating AOM, including herbal preparations, decongestants, and corticosteroids [14–16]. However, the evidence for these is too weak to be recommended in clinical practice. The anti-inflammatory effect of corticosteroids suggests it could be a viable treatment alternative for AOM [17]. Moreover, corticosteroids are effective additions to treatment for other, more serious acute respiratory infections, including pneumonia [18] and bacterial meningitis [19]. However, there is insufficient evidence of efficacy and harms for AOM. A Cochrane review of randomised controlled trials (RCTs) of corticosteroids for AOM (two small studies; very low to low quality) indicated it might be useful clinically, but the small sample size and wide confidence intervals around the observed results leave too much uncertainty [20]. An additional RCT of corticosteroids showed a reduction of the duration of ear discharge in children with AOM and ventilation tubes [21]. Use of corticosteroids over a short duration is unlikely to cause harm. A systematic review identified side effects of short-course of corticosteroids (less than 2 weeks) in children, such as gastrointestinal disturbances and some behavioural changes. Due to diversity of corticosteroids’ types and duration in included studies, the results were uncertain for both important beneficial and harmful effects of corticosteroids [22].

Accordingly, we plan to conduct an adequately powered clinical trial to address the uncertainties around the effectiveness of corticosteroids for AOM in children. Initially, we plan to conduct the pilot study described here with an associated mechanistic sub-study using tympanometry. The pilot study will test the feasibility of characteristics of our main study design and all the study procedures, as well as other operational strategies in our proposed main study. As one of our outcomes is to assess the ability to measure planned outcomes of the proposed main study, we will also obtain the outcomes and report these narratively. The mechanistic sub-study will explore the potential mechanism of action of corticosteroids in the resolution of middle ear effusion in AOM.

## Methods/design

### Study aims and objectives

As this is a pilot study, we aim to test all pre-specified methods and procedures that will be implemented in the main study, including the overall process, resources, management, and scientific components, in a smaller size study.

The objectives for the pilot study are (1) to assess the overall process and procedures of the main study (e.g. the recruitment, randomisation, outcome measurement), (2) to identify the experience and obstacles of physicians and patients during the study, and (3) to verify the sample size calculation for the main study. The objective for the mechanistic sub-study is to assess the mechanistic effect of corticosteroids in improving middle ear effusion in children with AOM using tympanometry.

### Study design and setting

This is a pilot parallel, pragmatic, stratified, randomised, open-label, single-blind, controlled study in an allocation ratio of 1:1 (see Additional file 1. Protocol – Pilot OPAL Study).

We are going to conduct this study in seven hospitals in Jakarta and Bekasi: (1) Dr. Cipto Mangunkusumo Hospital, (2) Persahabatan Hospital, (3) Gatot Subroto Army Hospital, (4) Antam Medika Hospital, (5) Cempaka Putih Islamic Hospital, (6) Proklamasi ENT Hospital, and (7) Hermina Bekasi Hospital.

### Participants

#### Inclusion criteria

We will include children aged 6 months to 12 years old with AOM, defined as current onset (within 48 h) of AOM-relevant symptoms (e.g. earache, ear tugging/rubbing or irritability in non-verbal children). Otoscopic findings of acute inflammation (e.g. erythema) and middle ear effusion (e.g. bulged tympanic membrane, immobile tympanic membrane, air fluid level) will confirm the diagnosis. Due to the pragmatic nature of this study, we have chosen to reflect real practice, where physicians often solely diagnose AOM based on symptoms alone because of several limitations in visualising the ear drums (e.g. non-cooperative children, narrow ear canals, obstructing ear wax). Therefore, the otoscopic examination is not compulsory in diagnosing AOM. However, prior to the study, we will emphasise the importance of the use of otoscope in diagnosing AOM to the participating physicians and conduct training to visually identify clinical signs of AOM using otoscope.

#### Exclusion criteria

We will exclude children (1) with major and severe medical conditions (e.g. heart diseases, kidney failure, tuberculosis), (2) who are immunocompromised (e.g. HIV, in cancer treatment), (3) with congenital malformations and/or syndromes (e.g. cleft palate, Down’s syndrome), (4) who have high risk of strongyloidiasis infections, (5) with ear ventilation tube(s), (6) who have been exposed to persons with varicella (chicken pox) or active Zoster infection in the past 3 weeks without prior varicella immunisation or infection, (7) who have taken systemic (oral, injection) or topical steroids in the preceding 4 weeks, (8) who have taken antibiotics in the preceding 2 weeks, and (9) who are hypersensitive to prednisolone or prednisone, or other corticosteroids.

#### Study intervention arm

Prednisolone tablets (Lupred®5) will be given at a dose of 1–2 mg/kg of body weight per day. As there is a wide therapeutic dose window for prednisolone, this will enable us to operationalise the dose as 10 mg/day for children aged 6 months to up to 2 years; 20 mg/day for children aged 2 up to 6 years; and 30 mg/day for children aged 6 to 12 years, simplifying both randomisation and dosage instructions. We determined the dose and duration of prednisolone based on the paediatric otitis media studies and other national and international practice guidelines of inflammatory and infectious diseases in children (e.g. bronchial asthma, juvenile rheumatoid arthritis, acute bacterial meningitis) [23–27]. An animal study [28] using mice infected with common causative bacteria of AOM (*Streptococcus pneumoniae*, non-typeable *Haemophilus influenzae*) demonstrated that most AOM-related cytokines (e.g. interleukin 1 alpha/IL-1α, tumour necrosis factor alpha/TNF-α) peaked at 3 to 6 h, progressively reduced on day 4 to day 6, and eventually were resolved after day 6. We will give the prednisolone for 5 days to boost the natural resolution process in middle ear inflammation and to minimise potential harms of corticosteroid use. A morning single daily dose (6 to 8 am) is preferable over divided doses to prevent the hypothalamic–pituitary–adrenal (HPA) axis suppression and for the convenience of the children and parents. Due to the bitter taste of prednisolone despite the addition of sweetener, we will advise the parents to mix the powder with jam or sweet juice to make it more palatable for the children.

We will stratify by disease severity and randomise children with mild AOM to receive prednisolone plus expectant observation, or expectant observation alone. Those with severe AOM will be randomly allocated to receive prednisolone plus antibiotic, or antibiotic alone.

#### Control arm

Budget constraints preclude us using matched placebo; children allocated to control group will not receive prednisolone but will receive standard care based on the severity of their AOM (i.e. observation for mild AOM, antibiotics for severe AOM).

#### Concurrent treatment

Prior to the randomisation, physicians may prescribe symptomatic medications (e.g. antipyretic, analgesic, decongestant) according to their usual practice. The physicians will not prescribe systemic corticosteroids. Therefore, the choice in prescribing these medications will not be influenced by subsequent knowledge of allocated treatment group.

#### Criteria for study drug discontinuation or modification

If children vomit less than 30 min after having a dose of prednisolone, parents should give the same dose again. However, if they vomit again after 30 min, parents should not give another dose of prednisolone until the next dose on the next day. If children keep vomiting after receiving prednisolone, parents should contact the research team. If the parents forget to give prednisolone to their children, they can give the missed dose as soon as they remember on the same day. To prevent this, we developed several reminder strategies, such as daily text-message reminders and a reminder note at the end of the first 5 days in a daily symptom dairy. This will remind the parents to give the study medication after completing the symptom diary on that particular day.

If there are any adverse events and adverse drug reactions which have been assessed by the research team that would require the discontinuation of drug study and further assessment and treatment, the treatment will be discontinued for this particular case; however, follow-up will continue, where possible.

#### Adherence monitoring

Participating physicians will provide information regarding the administration of the prednisolone with the prescription. One researcher will send daily text-message reminders to all of the parents in both prednisolone and control groups to (1) take the study medication regularly (during the intervention period of 5 days), (2) complete the symptom diary daily until day 14 (2 weeks only), and (3) visit the clinic for re-assessment at day 3 (visit 1), day 7 (visit 2), day 30 (visit 3), and day 90 (visit 4). At visit 1 and visit 2, the parents will return the first and second mini booklets of symptom diary and the left-over drug to the appointed nurse (at visit 2) for assessment of adherence to study medication. We will visit the patients’ homes at day 14 to collect the last (the third) mini booklet of symptom diary that will record the symptoms from day 7 to day 14 after the baseline visit (see Additional file 2. Case report forms – Pilot OPAL Study: CRF06. Symptom diary).

For participants and parents who are no longer willing to participate in the study (e.g. withdrawal from the study, not taking the study medication), we will still encourage them to come to their scheduled follow-up visit. This will enable us to collect the outcome data for those who are no longer in the study.

#### Outcomes

Our outcomes in this pilot study are to determine (1) the recruitment rates, (2) the success of the study procedures, (3) the ability to measure planned outcomes in the main study, (4) the compliance to study visits and study medication, and (5) the verification of sample size calculation for the main study.

Recruitment rate is defined as the proportion of consultations with potentially eligible children who provide their consent to be included in the study. This is a crucial aspect in a clinical trial. Low recruitment can result in a discontinuation of an on-going study [29]. We will assess this outcome at each month for the overall 6-month recruitment duration of the pilot study.

We will assess the success of the study procedures by identifying the process and obstacles during the following procedures: (1) obtaining informed consent from the patients and their parents; (2) recruitment using prespecified eligibility criteria and the use of otoscope to confirm AOM if feasible; (3) stratification and randomisation, including accessing the randomisation system and dispensing the study medication; and (4) identification of AOM symptoms and signs by clinical history taking and examination using otoscope and tympanometry. In AOM cases with earwax, we will extract the earwax before performing the otoscopy and tympanometry examination. If the earwax extraction is not feasible (e.g. uncooperative patients, cerumen prop), we will not include this patient in the mechanistic sub-study, but we still include this patient in the pilot study (as long as this patient fulfils the study criteria). Patients with tympanic membrane perforation will also not undergo tympanometry examination and will be included in the pilot study only. We will assess this outcome at the baseline visit (visit 0), day 3 (visit 1), day 7 (visit 2), day 30 (visit 3), and day 90 (visit 4).

We will identify the ability and challenges in measuring planned outcomes in the main study from the perspectives of participating physicians, nurses, and audiologists, as well as the eligible children and their parents. For example, we want to know whether it is difficult for the physicians to identify the pain severity using visual analogue scale (VAS) and acute otitis media severity of symptoms scale (AOM-SOS), as these are not common tools that are used in the management of AOM.

The compliance to study visits and study medication is defined as a proportion of children who regularly take the study medication according to the prespecified dose and duration (assessed using the symptom diary and the number of any left-over drug) and who come to follow-up visits per protocol. Participants will be followed closely by physicians and research staff. Children will return for a visit at day 3 after randomisation (visit 1), ensuring collection of the primary outcome.

The last outcome in this pilot study is to verify sample size calculation for the main study. Based on our size calculation, we must enrol 760 children with AOM. We estimated that there will be 35% of the total sample of children with AOM in the severe group (i.e. children with severe symptoms, fever ≥ 39 °C, children aged < 2 years with bilateral AOM, AOM with perforation of tympanic membrane). Within this pilot study, we will identify whether there will be a sufficient number of children for our main study in each stratum and the event rate in the control group.

We will also conduct a mechanistic sub-study using tympanometry. As a primary outcome, we will assess the change of middle ear effusion at similar time points with the pilot study. We will measure middle ear effusion using static acoustic admittance, defined as ‘the amount of energy absorbed by the tympanic membrane and middle ear, measured in millimetre ohm or millilitre’ [30]. The secondary outcomes are determining (1) the duration of middle ear effusion and (2) the correlation between ear pain and other symptoms (i.e. ear tugging, irritability, crying, lack of sleep, lack of appetite, loss of playfulness, fever) with the changes in middle ear effusion at various time points.

Assessing the feasibility of measuring planned outcomes in the main study (e.g. proportion of children with pain at day 3, proportion of children with pain and other non-specific AOM symptoms at various time points, adverse effects, recurrence) will provide the results for these outcomes. However, we will report these narratively due to a limited sample size and insufficient formal power calculation, which makes us unable to detect actual effects of corticosteroids to improve clinical outcomes in AOM.

### Study procedure

#### Study site selection and training

In 2016, we tested feasibility of this study by surveying physicians (general practitioners; ear, nose, throat specialists; and paediatricians) in three cities (DKI Jakarta, Depok, Bekasi) in Indonesia, asking about current management of AOM in children, and their willingness to participate in our proposed main study. We found there were sufficient physicians who would prescribe corticosteroids for AOM among the 171 physicians from 87 primary/secondary to tertiary healthcare centres, in our proposed study. For practical reasons, we will only include seven hospitals in Jakarta and Bekasi.

To ensure that all procedures and the outcome data can be sufficiently conducted, collected, and recorded properly according to prespecified plans, we will conduct training for participating physicians prior to the implementation of the study. The training will include the implementation of a clinical trial based on good clinical practice guidelines, the summary of our study, and the procedural steps in our study from the eligibility identification, stratification, to data collection and management. We will also provide training for nurses, pharmacists, and tympanometry technicians in terms of the randomisation process, dispensing and preparing the study medication, and conducting and completing the outcome form for tympanometry examination (see Additional file 3. Manual of operations – Pilot OPAL Study and Additional file 4. Training slides – Pilot OPAL Study).

#### Recruitment and stratification

**Fig. 1:**
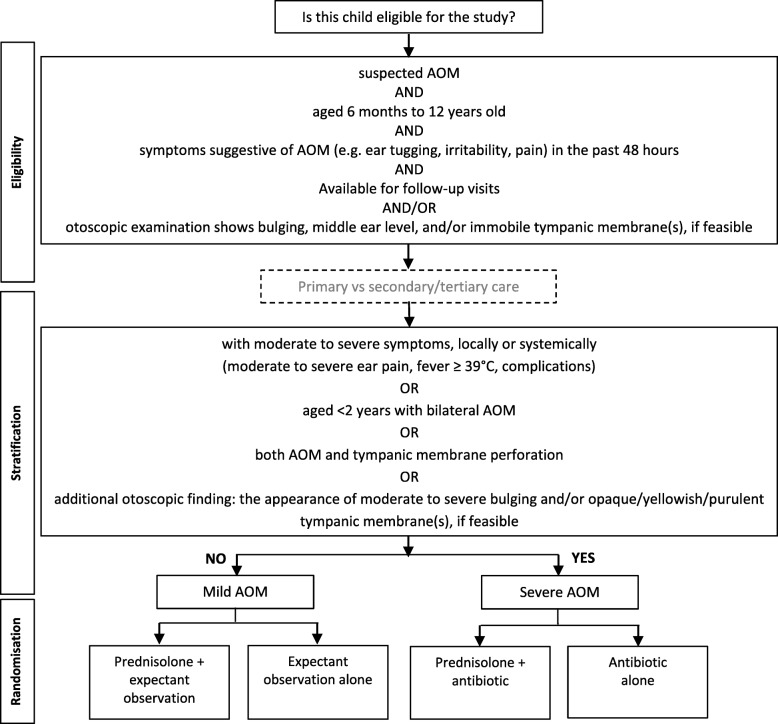
Flow chart of the stratification and randomisation of the study

In the main study, we will stratify eligible children by the clinical specialty (primary care or secondary/tertiary healthcare centres) and severity of AOM (mild or severe). In this pilot study, we will only include ear, nose, throat (ENT) specialists who work in tertiary healthcare centres (see Fig. 1). Therefore, we will stratify the eligible children only based on their AOM severity. Children with mild AOM symptoms and signs (e.g. mild ear pain, fever < 39 °C) will be considered the mild AOM group, whilst those with moderate to severe symptoms and signs (e.g. moderate to severe ear pain, fever ≥ 39 °C, moderate to severe bulging of tympanic membrane, children aged < 2 years with bilateral AOM, AOM with perforated tympanic membrane, complications) will be considered the severe AOM group. We will then randomly allocate them to receive either single dose prednisolone for 5 days as an addition to expectant observation compared to observation alone (mild AOM group) or as an addition to antibiotics compared to antibiotic treatment alone (severe AOM group).

#### Randomisation and allocation concealment

All consenting children and their parents who are eligible will be enrolled and stratified based on their AOM severity by the participating physician. The eligibility and stratification which is provided by the physicians will help the appointed nurses to obtain the information from the randomisation website, developed by Centre for Research in Evidence-Based Practice (CREBP) Bond University, Queensland, Australia. The randomisation information for each subject is the intervention allocation and two-digit randomisation ID. A permuted block randomisation sequence will be computer-generated, prior to study commencement. The children will then be randomly allocated to either prednisolone and expectant observation or expectant observation alone in the mild group and either antibiotic with prednisolone or antibiotic alone in the severe group.

During the consultation, the physician will prescribe study medication for every study participant with the dose based on age, and the nurse will give the prescriptions to the participants who are allocated to the intervention group (prednisolone group). Using the prescription, the pharmacist will prepare the prednisolone by crushing the tablets based on the prescribed dose, mixing them with sweeteners, packing the mixed powder in daily paper medication packs for 5 days. This medication preparation procedure is commonly implemented for paediatric populations in Indonesia. The pharmacist will then dispense the study medication along with instructions for preparation and record the dispensing on the form provided by the study for this purpose. Batches of study medication will be dispatched to participating centres from a central pharmacy facility at the Clinical Research Supporting Unit, Faculty of Medicine Universitas Indonesia (CRSU FMUI).

#### Blinding

The appointed nurses, the study participants, and their parents will know the allocation of the intervention. We will ensure that the participating physicians and the audiologists/tympanometry technicians will be blinded to the intervention allocation until all study outcomes, particularly pain, at day 3 (visit 1) are collected. At day 3, study participants will meet the appointed nurses before having a consultation with the physicians or undergoing the tympanometry examination to ensure blinding of outcome assessors. Emergency unblinding, before day 3, can occur if there are serious adverse events (SAEs) and only limited to the particular physician and patient who is experiencing the SAE.

#### Follow-up timeline

We will measure the outcomes at various time points: (1) visit 1 after 48-h observation (day 3), (2) visit 2 (day 7), (3) visit 3 (day 30), and (4) visit 4 (day 90). In the main study, patients will visit the hospital at visit 1 and visit 2, whilst the last two visits will be home visits. However, since the pilot study will be conducted simultaneously with the mechanistic sub-study, all the patients will have four hospital visits for tympanometry examination. We will do a home visit at the second week to collect the symptom diary (see Table 1).

**Table 1:**
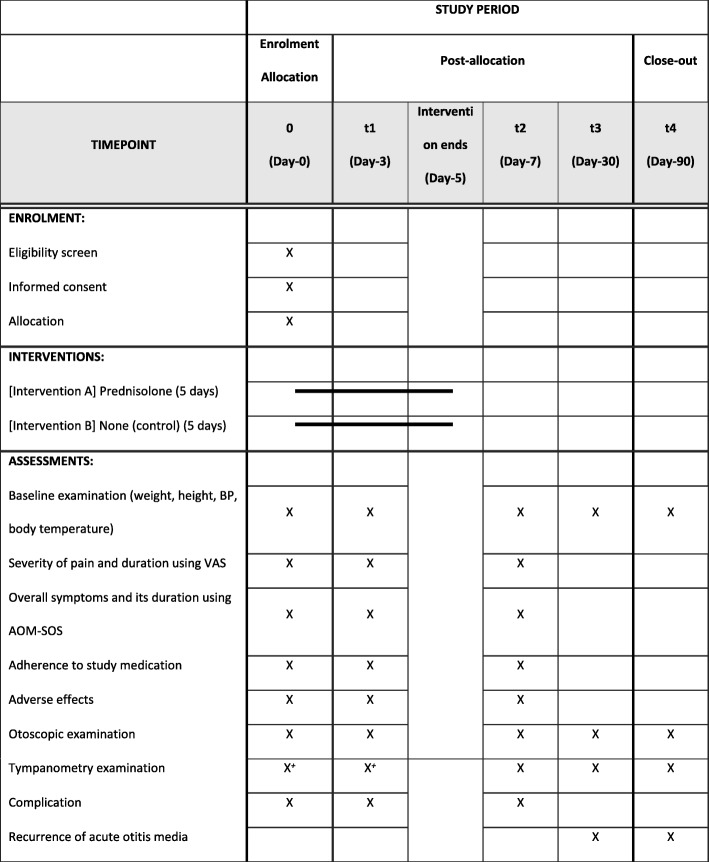
The schedule of enrolment, interventions, and assessments

### Data collection

We will use a consent form, a recruitment log book, case report forms (CRFs), and a symptom diary to measure and record all outcomes. The case report forms consist of (1) eligibility form, (2) baseline information form, (3) outcomes form, (4) randomisation form, (5) study medication dispensing and return form, (6) feedback form, and (7) serious adverse effects form.

We will identify the recruitment rate by assessing the proportion of parents/children who provide their consent divided by the proportion of consultations with potentially eligible children during the study. We will use a study recruitment log book to record the reason(s) why children were not randomised.

We will assess the success of the study procedures using a feedback form. Using the feedback form, physicians, nurses, pharmacists, and the parents will rate their understanding and challenges they encountered during the implementation of study, including the completion of the case report forms and the symptom diary, the randomisation process, and dispensing and the preparing of study medication. They will grade the severity of the challenges or obstacles on a scale with a range from very easy to very difficult (see Additional file 2. Case report forms—Pilot OPAL Study: CRF11. Feedback form).

To assess the ability to measure planned outcomes in the main study, we will also use a feedback form to identify the understanding, the challenges, and the complexity of the outcome assessment tools utilised for this study (i.e. CRFs and patient symptom diary) from the perspective of the study participants, their parents, and the participating physicians (see Additional file 2. Case report forms—Pilot OPAL Study: CRF05. Outcome form and CRF06. Symptom diary). The CRFs and symptom diary will record the clinical history and symptoms (e.g. VAS, AOM-SOS), as well as physical examination (e.g. temperature, blood pressure, otoscopic examination if feasible). The VAS is acknowledged as a well-established and validated scale for assessing pain [31]. It has a 100-mm horizontal scale with ‘no pain’ anchor at the left and ‘the most severe pain’ at the right endpoint of the scale. The scale will be determined by measuring the distance from the left endpoint (‘no pain’) to the line representing the pain level, marked by the parents or older study participants (≥ 8 years old) [32]. A 10-mm difference has been reported to indicate a clinically significant change [33, 34]. The AOM–SOS is used to assess the severity of other acute otitis media-relevant symptoms daily and activity limitation due to acute otitis media in the proceeding 12 to 24 h [35], particularly in non-verbal children, using a scale of ‘no’, ‘a little’, and ‘a lot’. Shaikh et al. [35] used the mean of 4.2 points as a minimal important difference. We have translated the original (English) version of AOM–SOS to an Indonesian version of AOM–SOS through forward and backward translation process.

The compliance to the study and study medication will be measured by assessing the completion of CRFs and symptom diary, particularly the attendance of the study participants and their parents to their scheduled follow-up visits, the completion of the study medication based on the symptom diary, and the left-over drug.

We will use the CRFs to assess the verification of sample size calculation for the main study.

### Data management

The integrity and completion of data will be maintained through consistency checks during data entry and cross-checks between items after data entry. All the actions and modifications to data stored in the database will be documented and retrievable for viewing. Any modification to original forms will be documented with the date, name, and signature on paper and electronic versions. Missing data or errors will be detected before final submission to the electronic central database. This central database will be checked regularly for its validity and completeness of study data and will be protected with a regular complete backup system.

### Sample size

Even though our sample size calculation for our proposed main study demonstrated that we need to recruit 760 children with AOM, we did not formally determine the sample size for this pilot study. There are several suggestions in calculating the sample size for a pilot study (e.g. at least 55 participants or at least 9% of the sample size of the main study) [36]. Since we will need 60 children for our mechanistic sub-study, we will also include 60 children with AOM in our pilot study. The sample size of the mechanistic sub-study was determined based on the main primary outcome, which is the mean value of static acoustic admittance or acoustic compliance in the tympanometry findings. In a previous study of children with middle ear effusion who underwent tympanometry assessment and had a history of chronic or recurrent middle ear disease [37], the response within each subject group was normally distributed with standard deviation 0.3. If the true difference in the experimental and control means is 0.3 units, we will need to study 22 experimental subjects and 22 control subjects to be able to reject the null hypothesis that the population means of the experimental and control groups are equal with probability (power) 0.9. The type I error probability associated with this test of this null hypothesis is 0.05. With a 20% allowance for dropouts, the total sample size becomes 56; consequently, we will include 60 children for this pilot study.

We will recruit children with AOM from seven tertiary healthcare centres that have tympanometry and are located in eastern and central Jakarta. Our survey study demonstrated that there were sufficient numbers of potential paediatric AOM patients (97 children with AOM in a week) and physicians (50 physicians) who were willing to participate in the study at these hospitals, meaning physicians see between 1 and 2 potentially eligible patients/week. Using a worst-case scenario, we assumed that only 30% of physicians would participate and only 25% of patients would give consent to participate in our study, equating to a maximum of 29 children per month. However, it is likely to take several weeks to months for sites to recruit to optimum levels, so we have allowed 6 months to recruit 60 children with AOM. The study duration will be 9 months: 6 months for recruitment plus 3 months for final follow-up data collection (3 months post-enrolment).

In order to achieve adequate participant enrolment, we will train nurses in each hospital on how to screen all children with acute ear symptoms (e.g. ear pain, ear tugging, ear discharge). We will ensure all study documents are accessible by providing binders of study recruitment log book and case report forms at nursing stations. In the first 2 weeks of the study (at least 3 days per week), a researcher will also stand-by at the hospital to help the nurses to identify and screen children with suspected AOM.

### Statistical methods

For the recruitment rate, we report the outcome as the proportion of children in percentages. For the success of the study procedures and the ability to measure planned outcomes in the main study, we will report the outcomes as the proportion of physicians in percentages based on the grading scale of their feedback report on prespecified outcome measure tools. For the compliance to study visits and study medication, we will report the outcomes as the proportion of children in percentages who attend the follow-up visits and complete the cycle of study medication.

To assess the verification of sample size calculation for main study, we will report this outcome as the proportion of children in each stratum (mild and severe acute otitis media group) and those with pain at day 3 after randomisation in the control group.

Although we will not formally report the clinical outcomes due to a limited sample size and insufficient formal power calculation of this pilot study, we will report and analyse the clinical outcomes of the mechanistic sub-study using tympanometry. We plan to analyse by intention-to-treat; however, if there is loss to follow-up, we will not impute data, but use an available case analysis, due to the small sample size. We will still record data on those who stop study medication, where possible, and will include them in the analysis. For the mechanistic sub-study, we will report continuous variables (i.e. the change in middle ear effusion at various time points (mean in day; standard deviation), the duration of middle ear effusion, the difference between two groups) as a mean difference with 95% confidence intervals (CI). We will also report the correlation between ear pain and other symptoms with the changes in middle ear effusion at various time points.

### Data monitoring

Since this is a short study, this pilot study does not require a data monitoring committee. However, independent personnel from Clinical Epidemiology and Evidence-Based Medicine (CEEBM) Unit, Dr. Cipto Mangunkusumo Hospital (CMH) – Faculty of Medicine Universitas Indonesia (FMUI), who are not involved in this study, will assess the process and the quality of patient recruitment, data entry, and a compilation of research data in the central database. Serious adverse event cases will be identified, assessed, and managed by physicians. These cases will then be recorded and reported to, as well as be reviewed by the Medical Ethics Committee Faculty of Medicine Universitas Indonesia (FMUI) (Indonesia) and the Bond University’s Human Research Ethics Committee (BUHREC) (Australia).

### Interim analysis

Due to the small number of recruited patients to the study and because the duration of the study will be less than 1 year, we will not conduct an interim analysis.

### Harms

An adverse event is defined as any untoward medical occurrence in a patient or clinical investigation subject administered a pharmaceutical product and which does not necessarily have to have a causal relationship with study medication, whereas adverse effects or adverse drug reactions are defined as all noxious and unintended responses to a study medication related to any dose. Adverse events and adverse drug reaction will be collected after parents of the eligible children sign the written consent and being enrolled in the study. All adverse events occurring after the enrolment into the study, during the additional treatment, or hospitalisation due to adverse events and/or adverse drug reaction will be recorded. A subject who experiences serious adverse events, defined as any untoward medical occurrence at any dose that may result in-patient and/or prolonged hospitalisation, persistent or significant disability, medically important events, life-threatening events, and death, will receive sufficient treatment and will be recorded and reported to the Medical Ethics Committee FMUI and the BUHREC. We will not report serious adverse events occurring after the study discontinuation, unless there is a temporal relationship between study medications or other protocol procedure to the events, as well as whether the event is unexpected or unexplained given the subject’s clinical course, previous medical conditions, and concomitant medications. All the serious adverse events will be recorded in the serious adverse event form.

### Auditing

For the main study, we will establish an audit committee from the CRSU FMUI and CEEBM Unit CMH-FMUI which is independent from the study investigators. Observation and quality assessment of the study will be ensured to be always in accordance with the protocol and International Conference Harmonization – Good Clinical Practice (ICH-GCP) standards. However, we will not conduct this in the pilot study because it is a short and small size study.

### Protocol amendments

Any modifications to the protocol which may impact on the study process (e.g. modification of study objectives, study design, study population, sample sizes, study procedures), potential benefits, and safety of the patients will require a formal amendment to the protocol. This amendment will be notified and approved by the Ethics committee prior to its implementation. Notification will also be sent to the health authorities in accordance with local regulations. Minor modifications that may not impact on the study process will only be notified to the Ethics committee.

### Confidentiality

All information related to the study will be securely stored using password-protected access systems. These forms will be kept confidential by only using coded patient IDs as identifiers and will be stored separately from all forms and records that contain names or other identifiers (e.g. informed consent forms). All counselling sessions, including general, ear-nose-throat, and tympanometry examinations, will be conducted in private rooms. All the involved research staff such as physicians, nurses, audiologists, and pharmacists will be required to sign agreements to preserve the confidentiality of all participants. The confidentiality of every participant will be maintained and will not be distributed externally without the written permission of the participant, except for medical and research safety purposes by national regulatory authorities if necessary.

### Access to data

The principal investigator will be given access to the cleaned data sets. She will also have direct access to each site’s data sets and by request. Project data sets will be secured using passwords. To ensure confidentiality, data dispersed to project team members will be blinded of any identifying participant information.

### Ancillary and post-study care

Short-term corticosteroids are very unlikely to have harm outside those we will be measuring. However, we will observe any potential adverse effects from the study medication using a symptom diary. The symptom diary will record adverse effects commonly found in corticosteroid treatment (e.g. gastrointestinal disturbance, behavioural changes), AOM complications (e.g. eardrums perforation, mastoiditis), and also the severity of pain and other non-specific symptoms of AOM. We will provide a 24-h call centre for any emergency assistance and send regular text reminders to the parents (for taking medication and completing the diary), where parents will be able to report any deteriorating or worsening symptoms of AOM. We will also provide a list of healthcare providers to manage emergency cases that might occur during the study. We will be responsible for the adverse effects that will occur from the study medication during and after the study related to study medication. The compensation will include the treatment cost relevant with the study medication, such as consultation visits, additional examinations, and treatment (e.g. medicine, hospitalisation cost). Due to other potential concurrent treatments within the study medication, there will be robust review and analysis process to conclude the cause of adverse events. Information of management of adverse effects will be provided by physicians during the process of consent approval before entering the study. We will also include this information in the patient symptom diary, including the 24-h emergency call and list of recommended healthcare providers.

### Dissemination policy

Study results, either statistically significant or non-significant, will be reported in a journal manuscript after being distributed to all the investigators to be reviewed.

The authorships and contributions of this study will be acknowledged on the protocol, manuscript, and the report. Before the publication in medical journal or paper presentation, the principal investigators will provide written consent of their acknowledgment and contribution in the reported study.

### Reproducible research

We will make the full protocol of this study to be publicly available to maintain its transparency and reproducibility. This full protocol will include detailed information regarding the study, particularly on study design and conduct that not are commonly included in the published protocol or information description in clinical trial registry. We have registered the protocol at the Australian and New Zealand clinical trial registry (https://www.anzctr.org.au/Trial/Registration/TrialReview.aspx?ACTRN=12618000049279). We will also publish the results of this study in relevant medical journals as two separate papers as the following: (1) results of the pilot study and (2) results of the mechanistic sub-study. If necessary, we will include the anonymised participant-level dataset in its appendix or online. Unpublished outcomes will be reported in the full study report that will be linked to the published study.

## Discussion

By conducting this pilot study and mimicking all the procedures in the main study, it enables us to identify any practical or operational issues in performing the main study (e.g. the recruitment and stratification process, outcome measurement, randomisation process, the compliance to the study). Our mechanistic sub-study will demonstrate whether the potential mechanism of action of corticosteroid will improve the resolution of middle ear effusion in AOM cases and whether the changes correlate with clinical symptoms of AOM.

We also presume that there are will be several challenges during the implementation of the main study, particularly in these following processes: (1) data collection using otoscopes, as is not compulsory due to the pragmatic design and the potential difficulties to visualise the children’s tympanic membranes; (2) the stratification and the management of AOM according to the AOM severity, particularly for children in the mild AOM group, due to unclear classification of AOM severity and antibiotic treatment for AOM in Indonesia; (3) clinical outcome measurement using the AOM symptom reporting tools (e.g. VAS, AOM-SOS, symptom diary) as this is still not practiced in the management of AOM; and (4) the randomisation process using a randomisation centre website to simplify the randomisation allocation process. This will be a new and challenging experience for most of the participating nurses who will run the randomisation.

We have identified several limitations in our study. The first limitation is we will only include seven hospitals in Jakarta and Bekasi, which will not represent the coverage of the main study that will involve more than 50 primary/secondary and tertiary healthcare centres. The second one is limited reliability of outcome assessment instruments due to wide range of ages in this study. The VAS has a limited reliability in self-report in young children due to their lack of cognitive skills and experience with scaling and estimating the magnitude. The parents will assess the severity of the pain on children aged up to 8 years old [34], whilst the AOM–SOS will also be used to assess the symptoms and activity limitation due to acute otitis media, which might be better in younger children (< 2 years).

This pilot study is crucial to ensure the successful implementation of the main study. The main study will provide high-quality evidence about the value of corticosteroid in improving the resolution of AOM (e.g. pain and other symptoms, middle ear effusion, recurrence). If positive, it will provide an alternative to antibiotics for children with mild symptoms, and a useful addition to antibiotics in those with severe disease. If negative, it will provide opportunity for researchers to test other potential alternatives for improving the clinical outcomes of AOM.

By conducting this study, we will determine the importance of (1) the identification of AOM severity in determining a sufficient, comprehensive, and evidence-based management of AOM and (2) the use of symptom assessment tools in the management AOM by introducing several feasible validated tools. This can improve the quality of the management of AOM, particularly in reducing the use of antibiotics for mild AOM. As part of capacity building support for health practitioners (i.e. physicians, nurses, audiologists, pharmacists) in Indonesia, this study will provide opportunity for them to be directly involved in clinical research and to develop their capacity for future research.

## Study status

We began recruitment on 22 February 2018. The protocol as described here was finalised on 17 October 2017.

## Additional files

Additional file 1: Protocol—Pilot OPAL Study. This file is a protocol of a pilot pragmatic, randomised, open-label, controlled study of an oral prednisolone for acute otitis media in children. This file can be accessed at https://pure.bond.edu.au/ws/portalfiles/portal/27513682/Additional_File_1._Protocol_Pilot_OPAL_Study.pdf (PDF 491 kb)

Additional file 2: Case report forms—Pilot OPAL Study. This file includes case report forms that are used in the pilot study: CRF01. Participant information sheet and consent form; CRF02. Study registration form; CRF03. Eligibility form; CRF04. Baseline information form; CRF05. Outcome form; CRF06. Symptom diary; CRF07. Prescription of study medication; CRF08. Randomisation form; CRF09. Follow-up visit card; CRF10. Serious adverse events reporting form; CRF11. Feedback form; FORM01. Study recruitment log book; FORM02. Study medication stock book; FORM03. Study medication dispensing form; FORM04. Study medication return form; FORM05. Completed case report form; FORM06. Recapitulation of non-participating subject form; FORM07. Guideline of antibiotics for acute otitis media; FORM08. Prednisolone dose for OPAL study; FORM09. Instruction for using prednisolone for parents; FORM10. Lupred pharmaceutical brochure. This file can be accessed at https://pure.bond.edu.au/ws/portalfiles/portal/27513684/Additional_File_2._Case_report_forms_Pilot_OPAL_Study.pdf (PDF 4716 kb)

Additional file 3: Manual of operations—Pilot OPAL Study. This file includes step-by-step manual for physicians, audiologists, nurses, and pharmacists who participate in the study. The manual of operations handbook was distributed during the trainings for participating physicians, audiologists, nurses, and pharmacists. This file can be accessed at https://pure.bond.edu.au/ws/portalfiles/portal/27513686/Additional_File_3._Manual_of_Operations_Pilot_OPAL_Study.pdf (PDF 7905 kb)

Additional file 4: Training slides—Pilot OPAL Study. The training slide was presented during the training for participating physicians, audiologists, nurses, and pharmacists. The training was conducted prior to the study commencement. This file can be accessed at https://pure.bond.edu.au/ws/portalfiles/portal/27513688/Additional_File_4._Training_Slides_Pilot_OPAL_Study.pdf (PDF 5528 kb)

## Abbreviations

ADR: Adverse drug reaction
AOM: Acute otitis media
AOM-SOS: Acute otitis media severity of symptoms scale
BUHREC: Bond University’s Human Research Ethics Committee
CEEBM CMH – FMUI: Clinical Epidemiology and Evidence-Based Medicine Unit, Dr. Cipto Mangunkusumo General Hospital – Faculty of Medicine Universitas Indonesia
CRF: Case report form
CRSU FMUI: Clinical Research Supporting Unit, Faculty of Medicine Universitas Indonesia
ICH-GCP: International Conference Harmonization – Good Clinical Practice
ID: Identifier
IL-1α: Interleukin-1 alpha
IL-6: Interleukin-6
NTHi: Non-typeable *Haemophilus influenzae*
ORL: Otorhinolaryngologist
RCT: Randomised controlled trial
SAE: Serious adverse event
TNF-α: Tumour necrosis factor alpha
VAS: Visual analogue scale
