# Supplementary material for: Oral prednisolone for acute otitis media in children: protocol of a pilot randomised, open-label, controlled study (OPAL study)
Source: Pilot Feasibility Stud. 2018 Sep 10;4:146. doi: 10.1186/s40814-018-0337-x (PMC6130070; doi:10.1186/s40814-018-0337-x)
Supplement: Supplementary file 1 — Protocol—Pilot OPAL Study. This file is a protocol of a pilot pragmatic, randomised, open-label, controlled study of an oral prednisolone for acute otitis media in children. This file can be accessed at https://pure.bond.edu.au/ws/portalfiles/portal/27513682/Additional_File_1._Protocol_Pilot_OPAL_Study.pdf (PDF 491 kb) [file 40814_2018_337_MOESM1_ESM.pdf]

Oral Prednisolone for Acute otitis media in chiLdren:

A pilot pragmatic, randomised, open-label, single-blind, controlled study (OPAL Study)

Centre for Research in Evidence-Based Practice
Faculty of Health Sciences and Medicine Bond University
Queensland, Australia

Clinical Epidemiology and Evidence-Based Medicine
Dr Cipto Mangunkusumo Hospital
Faculty of Medicine Universitas Indonesia
Jakarta, Indonesia

Administrative information

Protocol title

Oral **P**rednisolone for **A**cute otitis media in chi**L**dren: a pilot pragmatic randomised open-label single-blind study (**OPAL study**)

Authors

Respati W. Ranakusuma, MD, ORL

Dr. Amanda McCullough, PhD, PGCHET, BSc (Hons)

Associate Professor Elaine Beller, BSc, MAppStat

Professor Christopher Del Mar, FAFPHM, MA, MD, FRACGP, BSc

Professor Dr. Sudigdo Sastroasmoro, MD, PhD, Paed.

Eka Dian Safitri, MD, ORL

Yupitri Pitoyo, MD, ORL

Widyaningsih, MPH

Arie Sulistyowati, MD, MSc, Paed.

Table of Contents

Administrative information 1
Protocol title 1
Authors 1
Trial registration 4
The World Health Organisation Trial Registration Data set 4
Protocol version 6
Funding 7
Roles and responsibilities 7
Protocol contributions 7
Contact information of trial sponsor or funder(s) 8
Contact information of research team and others overseeing the trial 8

CHAPTER 1 – INTRODUCTION 10
1.1 Background and rationale 10
1.1.1 Background 10
1.1.2 Rationale for the proposed study 10
1.1.3 Rationale for pilot study 13
1.2 Objectives 14

CHAPTER 2 – METHODS 15
2.1 Trial design 15
2.2 Participants, interventions, and outcomes 15
2.2.1 Study setting 15
2.2.2 Eligibility criteria 15
2.2.3 Interventions 16
2.2.4 Outcomes 17
2.2.5 Participant timeline 19
2.1.6 Sample size 21
2.1.7 Recruitment 21
2.3 Assignment of interventions 21
2.3.1 Allocation 21
2.3.2 Blinding (masking) 22
2.4 Data collection, management, and analysis 22
2.4.1 Data collection methods 22
2.4.2 Data management 24
2.4.3 Statistical methods 24
2.5 Monitoring 25
2.5.1 Data monitoring 25
2.5.2 Harms 25
2.5.3 Auditing 26
2.6 Ethics and dissemination 26
2.6.1 Research ethics approval 26
2.6.2 Protocol amendments 26

2.6.3 Consent 26
2.6.4 Confidentiality 27
2.6.5 Declaration of interests 27
2.6.7 Ancillary and post-trial care 28
2.6.8 Dissemination policy 28

APPENDICES 29

REFERENCES 30

Trial registration

Registry name: http://www.ANZCTR.org.au/ACTRN12618000049279.aspx

The World Health Organisation Trial Registration Data set

Primary Registry and Trial Identifying Number	ACTRN12618000049279
Date of Registration in Primary Registry	16 January 2018
Secondary Identifying Numbers	-
Source(s) of Monetary or Material Support	Self-funded research
Primary Sponsor	Respati W. Ranakusuma, MD, ORL<br>Centre for Research in Evidence-Based Practice<br>Faculty of Health Sciences and Medicine Bond University, QLD, Australia<br>14 University Drive, Robina 4226, Queensland<br>Phone number: +61424957129 (Australia) / +6228111012185 (Indonesia)<br>Email: rranakus@bond.edu.au
Secondary Sponsor(s)	None
Contact for Public Queries	Respati W. Ranakusuma, MD, ORL<br>Centre for Research in Evidence-Based Practice<br>Faculty of Health Sciences and Medicine Bond University, QLD, Australia<br>14 University Drive, Robina 4226, Queensland<br>Phone number: +61424957129 (Australia) / +6228111012185 (Indonesia)<br>Email: rranakus@bond.edu.au
Contact for Scientific Queries	Respati W. Ranakusuma, MD, ORL<br>Email: rranakus@bond.edu.au<br>Phone number: +61424957129 (Australia) / +6228111012185 (Indonesia)<br>Centre for Research in Evidence-Based Practice Faculty of Health Sciences and Medicine Bond University, QLD, Australia<br>14 University Drive, Robina 4226, Queensland<br>Email: OPAL.study@bond.edu.au<br>Phone number: (+61) 7 559 51588
Public Title	Oral prednisolone for acute middle ear infection in children
Scientific Title	Oral prednisolone for acute otitis media in children: a pilot pragmatic randomised open-label single-blind study (OPAL study)
Countries of recruitment	Indonesia
Health Condition(s) or Problem(s) Studied	Acute otitis media in children
Intervention(s)	Intervention Name:<br>• Active intervention: Prednisolone tablet<br>• Active comparator: None<br>Intervention Description:<br>• Prednisolone tablet with doses based on range of age for five days:<br>o 6 months – <2 years: 10 mg/day<br>o 2 – <6 years: 20 mg/day<br>o 6 – 12 years: 30 mg/day<br>• None
Key Inclusion and Exclusion Criteria	Ages eligible for study: 6 months to 12 years<br>Sexes eligible for study: both males and females<br>Accepts healthy volunteers: no<br>Inclusion criteria:<br>• children (6 months – 12 years) with acute otitis media, defined as a current onset within 48 hours of ear-related symptoms (e.g. ear pain, ear tugging/rubbing or irritability) and if possible to assess, otoscopic findings of acute inflammation (e.g. erythema) and middle ear effusion (e.g. bulging, air-fluid level)<br><br>Exclusion criteria:<br>• children with major and severe medical conditions (e.g. heart failure, kidney failure)<br>• immunocompromised children (e.g. HIV, children receiving cancer treatment)

	• children with congenital malformations and/or syndromes (e.g. cleft palate, Down's syndrome)<br>• children with high risk of risk of strongyloidiasis infection<br>• children with ear ventilation tube(s)<br>• children who had exposed to persons with varicella (chicken pox) or active Zoster infection in the past 3 weeks without any prior varicella immunisation or infection<br>• children who have taken systemic (i.e. oral, injection) or topical steroids in the preceding four weeks<br>• children who have taken antibiotics in the preceding two weeks; a<br>• children who are hypersensitive to prednisolone or prednisone, or other corticosteroids.
Study Type	Type of study: interventional<br>Method of allocation: stratified, randomised<br>Masking: open-label, single-blind (outcome assessor)<br>Assignment: parallel<br>Purpose: Efficacy<br>Phase III
Date of first enrolment	01 January 2018
Target sample size	60 children
Recruitment status	Pending (not started): participants are not yet being recruited or enrolled at any site
Outcome(s)	(1) Outcome Name: Recruitment rates<br>Metric/method of measurement: Informed consent form and case report forms (CRFs)<br>The timepoint(s) of interest: Baseline visit (visit-0)<br>(2) Outcome Name: The success of the study procedures<br>Metric/method of measurement: CRFs (i.e. eligibility and randomisation form, outcomes form)<br>The timepoint(s) of interest: Baseline visit (visit-0)<br>(3) Outcome Name: Ability to measure planned outcomes in main study<br>Metric/method of measurement: CRFs (i.e. eligibility and randomisation form, outcomes form) and symptom diary (e.g. Visual Analogue Scale/VAS and Acute Otitis Media – Severity of Symptoms Scale/AOM-SOS), and feedback form<br>The timepoint(s) of interest: Baseline visit, visit-1 (day-3 to-5), visit-2 (day-7 to -9), day3, visit-3 (day-3 to -40), and visit-4 (day-90 to -100)<br>(4) Outcome Name: Compliance to study and study drug<br>Metric/method of measurement: CRF (i.e. outcomes form), the symptom diary, and number of left-over drug<br>The timepoint(s) of interest: Visit-1 (day-3 to-5), visit-2 (day-7 to -9), day3, visit-3 (day-3 to -40), and visit-4 (day-90 to -100)<br>(5) Outcome Name: The verification of sample size calculation for main study<br>Metric/method of measurement: CRFs (i.e. eligibility and randomisation form)<br>The timepoint(s) of interest: Baseline visit (visit-0)

Protocol version

Protocol version No.	Date issued
Protocol OPAL Study Version 1.0.0 (V1.0.0)	27 July 2017
Protocol OPAL Study Version 1.1.0 (V1.1.0)	17 October 2017

Protocol Amendment Number: AM1.0

Amendment history:

Amendment No.	Protocol version no.	Date issued	Author(s) of changes	Detail of changes made
Amendment No. 1.0	Protocol V1.1.0	17 October 2017	Respati W Ranakusuma	1. The modification of the form of trial drug<br>We planned to use prednisolone liquid. Due to administrative issues where the proposed pharmaceutical manufacturer was unable to share confidential documents that were required for importing the trial drug to Indonesia, we therefore will use prednisolone tablet for this trial. The pharmacist will crush the prednisolone tablets and mix it with sweetener. The crushed tablet will be packed in daily paper-package. The parents will mix the crushed tablet with juice or honey. This method is commonly practiced in Indonesia, particularly for paediatric patients.<br>2. The duration of the trial drug use<br>We planned to give prednisolone for seven days. Although 7-day duration of corticosteroid use is considered as a short-term use, we will reduce its duration to five days to minimise the potential harms caused by corticosteroids.

Funding

This research is supported by self-funding of the principal investigator (Dr. Respati W. Ranakusuma, ORL).

We will purchase the trial drug, prednisolone tablets, from PT Pratapa Nirmala, Tangerang, Indonesia. This pharmaceutical manufacturer is not linked to this study and does not have authority over any procedural implementation, scientific process, or decision in the study.

Roles and responsibilities

Protocol contributions

Name	Affiliation	Role of protocol contributors
**Dr. Respati W. Ranakusuma, ORL**<br>ENT surgeon, PhD candidate<br>Telp: +61 424 957 129 / +62 8111 012 185<br>Fax: N/A<br>Email: rranakus@bond.edu.au / respati.ranakusuma@ceebm.org	• Centre for Research in Evidence-Based Practice (CREBP) Faculty of Health Sciences and Medicine, Bond University 14 University Drive, Robina QLD 4226 Australia<br>• Clinical Epidemiology and Evidence-Based Medicine Unit, Dr. Cipto Mangunkusumo Hospital – Faculty of Medicine Universitas Indonesia Jl. Diponegoro 71 Jakarta 10430 Indonesia	Initiated the study design and methodology; developed the protocol including case report forms (CRFs), informed consent, and symptom diary
**Dr. Amanda McCullough, PhD, PGCHET, BSc (Hons)**<br>Epidemiologist, expert in acute respiratory infections and antibiotic resistance<br>Telp: +61 7 559 55204<br>Fax: N/A<br>Email: amccullo@bond.edu.au	Centre for Research in Evidence-Based Practice (CREBP) Faculty of Health Sciences and Medicine, Bond University, 14 University Drive, Robina QLD 4226 Australia	Initiated the study design from the clinical epidemiologic perspective; refined the protocol including case report forms (CRFs), informed consents, and symptom diary
**Associate Professor Elaine Beller, BSc, MAppStat**<br>Clinical trialist, biostatistician<br>Telp: +61 7 559 55523<br>Fax: N/A<br>Email: ebeller@bond.edu.au	Centre for Research in Evidence-Based Practice (CREBP) Faculty of Health Sciences and Medicine, Bond University, 14 University Drive, Robina QLD 4226 Australia	Initiated the study design and methodology from the clinical trialist and biostatistician perspectives; refined the protocol including case report forms (CRFs), informed consents, and symptom diary
**Professor Christopher Del Mar, FAFPHM, MBBChir, MA, MD, FRACGP, BSc**<br>Epidemiologist, evidence-based practitioner, expert in acute respiratory infection and antibiotic resistance<br>Telp: +61 7 559 52504<br>Fax: N/A<br>Email: cdelmar@bond.edu.au	Centre for Research in Evidence-Based Practice (CREBP) Faculty of Health Sciences and Medicine, Bond University, 14 University Drive, Robina QLD 4226 Australia	Initiated the study design from the clinical perspective; refined the protocol including case report forms (CRFs), informed consents, and symptom diary
**Professor Dr. Sudigdo Sastroasmoro, PhD, Paed**<br>Paediatrician, epidemiologist, evidence-based practitioner<br>Telp: +62 21 316 1760<br>Fax: N/A<br>Email: sudigdo1947@gmail.com	• Clinical Epidemiology and Evidence-Based Medicine Unit, Dr. Cipto Mangunkusumo Hospital – Faculty of Medicine Universitas Indonesia Jl. Diponegoro 71 Jakarta 10430 Indonesia<br>• Department of Child Health Faculty of Medicine Unit, Universitas Indonesia – Dr. Cipto Mangunkusumo Hospital DIponegoro 71 Jakarta 10430 Indonesia	Provided advice in paediatric perspectives, supervising the trial in Jakarta

Contact information of trial sponsor or funder(s)

Name	Contact information	Role of study sponsor and funders
**Dr. Respati W. Ranakusuma, ORL**	• Centre for Research in Evidence-Based Practice (CREBP) Faculty of Health Sciences and Medicine, Bond University 14 University Drive, Robina QLD 4226 Australia<br>• Clinical Epidemiology and Evidence-Based Medicine Unit, Dr. Cipto Mangunkusumo Hospital – Faculty of Medicine Universitas Indonesia Jl. Diponegoro 71 Jakarta 10430, Indonesia	She is the principal investigator

Contact information of research team and others overseeing the trial

Name	Affiliation	Roles and responsibilities
**Associate Investigators**		
**Dr. Eka Dian Safitri, ORL**<br>ENT surgeon<br>Telp: +62 21 316 1760<br>Fax: N/A<br>Email: ekadian.safitri@ceebm.org	Clinical Epidemiology and Evidence-Based Medicine Unit, Dr. Cipto Mangunkusumo Hospital – Faculty of Medicine Universitas Indonesia<br>Jl. Diponegoro 71 Jakarta 10430 – Indonesia	(1) involved in the development of tympanometry study; (2) providing training related to using and interpreting tympanometry to physicians, nurses, and tympanometry technicians
**Dr. Yupitri Pitoyo, ORL**<br>ENT surgeon<br>Telp: +62 21 316 1760<br>Fax: N/A<br>Email: yupitri.pitoyo@ceebm.org	Clinical Epidemiology and Evidence-Based Medicine Unit, Dr. Cipto Mangunkusumo Hospital – Faculty of Medicine Universitas Indonesia<br>Jl. Diponegoro 71 Jakarta 10430 – Indonesia	Recruiting secondary and tertiary healthcare centres including the physicians and nurses
**Dr. Arie Sulistyowati, MSc, Paed.**<br>Paediatrician, epidemiologist<br>Telp: +62 21 316 1760<br>Fax: N/A<br>Email: arie.sulistyowati@ceebm.org	Clinical Epidemiology and Evidence-Based Medicine Unit, Dr. Cipto Mangunkusumo Hospital – Faculty of Medicine Universitas Indonesia<br>Jl. Diponegoro 71 Jakarta 10430 – Indonesia	Providing advice and expertise in terms of paediatric patients
**Widyaningsih, MPH**<br>Public Health, qualitative study expert<br>Telp: +62 21 316 1760<br>Fax: N/A<br>Email: widyaningsih.ade@ceebm.org	Clinical Epidemiology and Evidence-Based Medicine Unit, Dr. Cipto Mangunkusumo Hospital – Faculty of Medicine Universitas Indonesia<br>Jl. Diponegoro 71 Jakarta 10430 – Indonesia	(1) submitting a research ethics application to the Indonesian Medical Research Ethics Committee; (2) recruiting primary healthcare centres including the physicians and nurses
**Research Coordinator**		
**Dr. Respati W. Ranakusuma, ORL**<br>ENT surgeon, PhD candidate<br>Telp: +62 8111 012 185<br>Fax: N/A<br>Email: rranakus@bond.edu.au / respati.ranakusuma@ceebm.org	Clinical Epidemiology and Evidence-Based Medicine Unit, Dr. Cipto Mangunkusumo Hospital – Faculty of Medicine Universitas Indonesia<br>Jl. Diponegoro 71 Jakarta 10430 – Indonesia	
**Co-investigators**		
**Dr. Tri Juda Airlangga H, ORL**<br>ENT surgeon<br>Telp: +6221 1500 135<br>Fax: N/A<br>Email: airlanggamd@gmail.com	Department of Ear, Nose, and Throat Head and Neck Surgery<br>Dr. Cipto Mangunkusumo Hospital – Faculty of Medicine Universitas Indonesia<br>Jl. Diponegoro 71<br>Central Jakarta 10430 – Indonesia	(1) Recruiting patients<br>(2) Coordinating patient recruitment among other clinicians in the hospital
**Dr. Yulvina, ORL**<br>ENT surgeon<br>Telp: +6221 470 1133<br>Fax: N/A<br>Email: yulie_dj@yahoo.com	Department of Ear, Nose, and Throat Head and Neck Surgery<br>Persahabatan General Hospital<br>Jl. Persahabatan Raya 1<br>East Jakarta 13230 – Indonesia	(1) Recruiting patients<br>(2) Coordinating patient recruitment among other clinicians in the hospital

**Dr. Evita Fitria Edyani, ORL**<br>ENT surgeon<br>Telp: +6221 344 1008<br>Fax: N/A<br>Email: evitafitria@yahoo.com	Department of Ear, Nose, and Throat Head and Neck Surgery<br>Gatot Subroto Army Hospital<br>Jl. Dr Abdul Rahman Saleh 24<br>Central Jakarta 10410 – Indonesia	(1) Recruiting patients<br>(2) Coordinating patient recruitment among other clinicians in the hospital
**Dr. Yupitri Pitoyo, ORL**<br>ENT surgeon<br>Telp: +6221 884 2121<br>Fax: N/A<br>Email: yupitri.pitoyo@ceebm.org	Department of Ear, Nose, and Throat Head and Neck Surgery<br>Hermina Bekasi Hospital<br>Jl. Kemakmuran 39, South Bekasi<br>West Java 17141 – Indonesia	(1) Recruiting patients<br>(2) Coordinating patient recruitment among other clinicians in the hospital
**Dr. Respati W. Ranakusuma, ORL**<br>ENT surgeon<br>Telp: +6221 2937 8939<br>Fax: N/A<br>Email: rranakus@bond.edu.au	Department of Ear, Nose, and Throat Head and Neck Surgery<br>Antam Medika Hospital<br>Jl. Pemuda 1A<br>East Jakarta 13210 – Indonesia	(1) Recruiting patients<br>(2) Coordinating patient recruitment among other clinicians in the hospital
**Dr. Eka Dian Safitri, ORL**<br>ENT surgeon<br>Telp: +6221 425 0451<br>Fax: N/A<br>Email: ekadian.safitri@ceebm.org	Department of Ear, Nose, and Throat Head and Neck Surgery<br>Jl. Cempaka Putih Tengah I/1<br>Central Jakarta 10510 – Indonesia	(1) Recruiting patients<br>(2) Coordinating patient recruitment among other clinicians in the hospital
**Dr. Hably Warganegara, ORL**<br>ENT surgeon<br>Telp: +6221 390 0002<br>Fax: N/A<br>Email: hablywarganegara@gmail.com	Proklamasi Ear, Nose, Throat (ENT) Centre<br>Jl. Proklamasi 43<br>Central Jakarta 10230 – Indonesia	(1) Recruiting patients<br>(2) Coordinating patient recruitment among other clinicians in the hospital
**Data Manager**		
**Respati W. Ranakusuma, MD, ORL**<br>ENT surgeon, PhD candidate<br>Telp: +61 424 957 129 / +62 8111 012 185<br>Fax: N/A<br>Email: rranakus@bond.edu.au /<br>respati.ranakusuma@ceebm.org	Clinical Epidemiology and Evidence-Based Medicine Unit, Dr. Cipto Mangunkusumo Hospital – Faculty of Medicine Universitas Indonesia<br>Jl. Diponegoro 71 Jakarta 10430<br>Indonesia	

CHAPTER 1 – INTRODUCTION

1.1 Background and rationale

1.1.1 Background

Antibiotic resistance, a major global threat, impacts more than two million people with illness and accounts for 23 thousand deaths annually in the United States [1]. One of the key drivers of the development of antibiotic resistance is antibiotic prescribing [2]. Antibiotics are mostly prescribed for common diseases, such as acute respiratory infections (ARIs) [3]. One of the ARIs commonly found in paediatric population with high antibiotic prescribing is acute otitis media [4,5]. In East Jakarta, Indonesia, the prevalence of AOM in children (< 18 years) was 5.4% [6]. In contrast, in Australia, there were an average of 35 new AOM cases reported by general practitioners per year (April 2010 – March 2015) [7].

Acute otitis media (AOM) is characterised by rapid onset of symptoms (e.g. earache, ear tugging/rubbing, irritability), middle ear effusion (e.g. bulging, immobile tympanic membrane, air fluid level), and acute inflammation (e.g. erythema) [8]. Almost three quarters of children have an episode of AOM in their first five years of life, with the peak incidence at the age of six to 12 months [9,10]. Guidelines recommend close monitoring for 48 hours (expectant observation) along with adequate pain management for mild AOM (e.g. mild symptoms, fever < 39°C) [8]. Children with severe symptoms, young age (< 2 years) with bilateral AOM, and AOM with tympanic membrane perforation are more likely to benefit from antibiotic treatment [11]. A high rate of antibiotic prescription for AOM is evident. Eighty-nine per cent new AOM cases were managed by antibiotics in Australia during 2010 to 2015 [7]. In Indonesia, a survey study using clinical scenarios, demonstrated that up to 88% of physicians would prescribe antibiotics for mild case of AOM. Unclear clarification of antibiotic use in the Indonesian practice guideline for AOM in the primary care may contribute to this [12]. The option of using antibiotics also must be balanced against the risks, such as adverse effects (e.g. vomiting, diarrhoea, rash) and antibiotic resistance [13,14].

1.1.2 Rationale for the proposed study

Prednisolone

Reducing antibiotic use is crucial to lowering the risk of antibiotic resistance. One of the methods is to use an alternative treatment that does not involve antibiotics. The current alternative treatments (i.e. ear drops, herbal products, probiotics, zinc, decongestants) demonstrate insufficient evidence on their benefits for AOM [15,16]. It is important to understand the pathophysiology of AOM, which is an inflammatory process involving both cellular and chemical inflammatory mediators (i.e. cytokines, chemokines, mast cells, prostaglandins, leukotrienes) in the middle ear. These inflammatory mediators contribute in altering the vascular permeability, increasing mucous glycoprotein secretion, as well as stimulating the chemotaxis process, the activity of epithelial secretion and other mediators [17]. An intervention suppressing this inflammatory process, could have an important role in the resolution of AOM. Corticosteroids suppress the inflammation process by inhibiting the mediators and cytokines characteristic of AOM, the recruitment of leukocytes and monocyte-macrophages into affected areas, and the synthesis and/or release of numerous inflammatory mediators and cytokine, and also reducing vascular permeability [18].

Corticosteroids are produced in the adrenal cortex. Cortisol (glucocorticoids), one of the most common corticosteroids, is responsible for anti-inflammatory effects. The production of glucocorticoids is controlled by hypothalamus, pituitary, and adrenal (HPA) axis. Corticosteroid treatment may affect the production of natural corticosteroid by suppressing the HPA axis [19,20]. We have identified prednisolone is commonly used and safe in the treatment of inflammatory and autoimmune diseases in children. Prednisolone, a synthetic intermediate-acting glucocorticoid with a biological half-life of 12 to 36 hours, is commonly used in the treatment of inflammatory and autoimmune diseases in children. Although prednisolone has a lesser anti-inflammatory potency compared to other common corticosteroids (i.e. methylprednisolone, dexamethasone), but it has lesser growth effect which is one of the concerning issues in the disease management in paediatric population [19].

We will give prednisolone at a dose of 1 mg/kg to 2 mg/kg body weight based on age category, once daily for five days. As there is a wide therapeutic dose window for prednisolone, this will enable us to operationalise the dose as 10 mg/day for children aged six months less than two years; 20 mg/day for children aged two to five years; and 30 mg/day for children aged six to 12 years, simplifying both randomisation and dosage instructions. The current treatment for AOM does not include corticosteroids in the guidelines. Therefore, we determined the dose and duration of prednisolone based on the doses regularly used in the paediatric otitis media trials and regular dose for other inflammatory and infection diseases in children based on the international and national practice guidelines, such as bronchial asthma, juvenile rheumatoid arthritis, and acute bacterial meningitis [21-25]. The duration of corticosteroid use in otitis media trials varies between three to seven days [18, 26-28]. An animal study [29] using mice infected with Streptococcus pneumoniae and non-typeable H. influenzae (NTHi) bacteria demonstrated that most of AOM-related cytokines peaks at three to six hours after the infections (interleukin-6/IL-6, interleukin-1 alpha/IL-1α, tumor necrosis factor alpha/TNF-α) an at six hours to three days (interleukin-10/IL-10). In general, these cytokines will be progressively reduced between the fourth to sixth day of infection and the acute otitis media will be resolved after the sixth day [29]. Therefore, we will give the prednisolone for five days in order to boost the natural resolution mechanism in AOM cases and to minimize the potential harms of corticosteroid use even though 7-day duration is still regarded as short-term use.

A single daily dose is preferable over divided doses to prevent the hypothalamic-pituitary-adrenal (HPA) axis suppression. Prednisolone should be given in a single dose at 6 to 8 am in the morning to mimic the normal diurnal rhythm of cortisol production [19,20] and because it is also more convenient for children and parents in the study to just take a trial drug once a day.

Potential harms

Despite the favourable effect of corticosteroids for inflammation, there are still several potential adverse effects related to its short-term use. A systematic review identified side effects of short-course of corticosteroids (less than two weeks) in children, such as gastrointestinal disturbances (i.e. vomiting, gastritis, nausea), behavioural changes (i.e. mood swings, nervousness), HPA axis suppression, increased blood pressure, hyperglycaemia, weight gain, and decreased bone mineralisation [30]. Even though there were more children experiencing these side effects compared to placebo, the included studies used a diverse of corticosteroids' types and duration, as well as the results were uncertain and include both important beneficial and harmful effects of

corticosteroid. Vomiting and behavioural disturbances (i.e. anxiety, aggressive behaviour) are the common side effects [30].

Regarding vomiting, there were three studies comparing prednisolone to placebo or control (other type of corticosteroids). A good quality RCT [31] included children aged 10 to 60 months with virus-induced wheezing who received a single dose of inhaled albuterol. These children then were randomly allocated to either prednisolone (10 mg oral prednisolone for children aged 2 years and younger; 20 mg for aged >2 years) group (n=343) or placebo (n=344). No significant differences on clinical outcomes (e.g. time to hospital discharge) or adverse effects between two groups were detected. There was one child from prednisolone group who vomited that required the discontinuation of the prednisolone [odds ratio (OR) 3.02 (95% confidence interval (CI) 0.12 to 74.33; p-value=0.50; number needed to harm (NNTH; number of children who are treated with prednisolone that will result in one additional event of side effects) = 34 children)] [31]. One study [32] on children aged one to 17 years with acute asthma presenting to the emergency department (ED). The children received a dose of inhaled albuterol and either a single dose of oral prednisone 2 mg/kg (n=41) or placebo (n=40). There was no a significant difference in the incidence of vomiting after taking the prednisone between the prednisone group (n=3; 7.3%) and the placebo group (n=1; 2.5%) [OR 3.08 (95% CI 0.31 to 30.92; p-value=0.34; NNH=21)] [32]. The same author with similar inclusion criteria [33] demonstrated a significant difference on the incidence of vomiting between children who received single dose prednisone 2 mg/kg/day (n=10/66) and nebulised dexamethasone 1.5 mg/kg (n=0/62), however the confidence interval was very wide that included a high number of NNTH (if we treat more than 50 children with prednisone, then we will expect one additional event of vomiting) [OR 23.23 (95% CI 1.33 to 405.54; p-value=0.03; NNH=7)] [33].

Regarding behavioural changes, one RCT [34] randomly allocated children aged two to 16 years with acute exacerbation of mild persistent asthma to receive either oral prednisone/prednisolone high dose (2 mg/kg/day) or low dose (1 mg/kg/day) for five days. There were significant differences in observed adverse events between high-dose and low-dose groups in regard to anxiety (9/43 vs 2/43, respectively) [OR 5.43 (95% CI 1.10 to 26.83; p-value=0.04; NNTH=7)] and aggressive behaviour (9/43 vs 0/43) [OR 23.96 (95% CI 1.35 to 426.33; p-value=0.03; NNTH=5)]. The wide of intervals demonstrated a wide variance in the number of children needed to treat to expect one additional adverse event. There were no significant differences in other unfavourable effects (i.e. facial fullness and erythema, abdominal pain, diarrhea, euphoria, depression, and hyperactive) [34]. Another RCT [35] included children aged two to 15 years with acute exacerbation of asthma who were randomly allocated to receive oral prednisolone 1 mg/kg/day for three days (5-day group) vs same dose of prednisolone for three days (3-day group). There were no significant differences in regard to the incidence of rash and behavioural disturbance (e.g. angriness, aggressiveness, crankiness, irritability) between the two groups [35].

An RCT of the use of prednisolone for pediatric AOM reported no significant differences between children who received oral prednisolone 2 mg/kg/day for five days and placebo group who experienced moderate side effects (e.g. drowsiness, nervousness, diaper rash, dry mouth) [18]. This study also demonstrated that there was no correlation between the use of corticosteroid and the persistence or the emergence of viral infections [18]. Other potential side effects correlated with the use of corticosteroids are fluid retention and headache [18,30].

Based on these trials, we consider 5-day duration of prednisolone for this study is appropriate and safe for children.

Clinical trial of corticosteroids for acute otitis media in children

Evidence has demonstrated insufficient benefits and harms of corticosteroids. An RCT demonstrated that corticosteroid reduced the duration of ear discharge in AOM children with ventilation tubes [26]. Another RCT demonstrated a temporary resolution of middle ear effusion after five days of corticosteroid treatment [18]. In a systematic review of randomised placebo-controlled trials (RCTs) of steroids for AOM, only two small trials [18,27,36] (very low to low quality) indicated corticosteroids could be useful in this condition. However, our confidence in the results is low, due to small sample size and very wide confidence intervals around the observed results. This insufficient evidence creates a research gap in the management of AOM, particularly in non-severe cases, where antibiotics are not required. Therefore, we propose an adequately powered clinical trial to address this uncertainty.

We will conduct a large, parallel, pragmatic, multicentre, stratified, double-blind, randomised, placebo-controlled trial with the allocation ratio 1:1 to test the effectiveness of corticosteroids for 760 children with AOM including 60 children for a tympanometric mechanistic sub-study and pilot study, described further in this protocol. As a comparator to prednisolone, we will use a placebo for the following advantages: (1) it is the most accurate test in assessing the efficacy of a treatment; (2) it will show the true additional benefits and/or harms of the prednisolone; and (3) it is crucial when the outcome is assessed using subjective measurements. The primary objective and outcome of this proposed trial is to assess the effectiveness of corticosteroids as a monotherapy in children with mild AOM, and as an addition to antibiotics in children with severe AOM, on ear pain at three days after randomisation using visual analogue scale (VAS). The secondary outcomes include ear pain at other time points, total duration and severity of pain, adverse effects, complications of AOM (e.g. perforation of tympanic membrane, mastoiditis), and AOM recurrence.

1.1.3 Rationale for pilot study

Prior to our main study, we will conduct a pilot study, described in this protocol. This study will mimic the main study in terms of its process and procedures, but on a smaller scale. However, due to budget constraints, we will conduct a pilot study as a pragmatic, randomised, open-label, single-blind study. We will blind outcome assessors (i.e. physicians and tympanometry technicians), so they will not aware of the allocation of the intervention.

The main study will involve many participating physicians and healthcare facilities across Jakarta, Bekasi, and Depok, most of whom have not been involved in a clinical trial before, will have a long follow-up period up to three months, and will utilise a symptom diary and a specific translated instrument to assess the severity of symptoms (acute otitis media – severity of symptom scale or AOM-SOS) which is not widely recognised by physicians in Indonesia. Therefore, this pilot study is crucial to test the feasibility of the main study, including the characteristics of our main study design, all the study processes and procedures (e.g. the recruitment, stratification, randomisation, outcome measurement), and other operational strategies in our proposed main study.

1.2 Objectives

The first objective of our pilot study is to assess the overall process and procedures of the main study, as follows: (1) the recruitment criteria; (2) stratification and randomisation processes; (3) outcome measures using validated and customised tools (e.g. visual analogue scale/VAS, Acute Otitis Media – Severity of Symptoms Scale/AOM-SOS, case report forms/CRFs, symptom diary); (4) identification of any potential practical and operational issues that may appear in the main study which will require re-structuration of the planned methods and procedures after commencing this pilot study; and (5) verification of sample size calculation for main study.

Our second objective is to conduct a mechanistic explanatory study using tympanometry. It aims to assess the efficacy of corticosteroids in improving the resolution of middle ear effusion in AOM.

CHAPTER 2 – METHODS

2.1 Trial design

This study is a pilot of a parallel, pragmatic, stratified, randomised, open-label, single-blind controlled trial of corticosteroids, as monotherapy for mild AOM, and in addition to individually prescribed antibiotics for severe AOM. In the main study, we will stratify eligible children based on the clinical specialty (primary care or secondary/tertiary care) and severity of AOM (mild or severe). However, for this pilot study, we only include ear-nose-throat-specialists (ENTs) who work at tertiary centres for the convenience of the implementation of the mechanistic sub-study as tympanometry is only available at the hospitals (tertiary centres). Therefore, we will stratify the children based on their AOM severity and then will randomly allocate to corticosteroid (prednisolone) or control (usual care without prednisolone) (Figure 1) with the allocation ratio of 1:1. Because it is a n open-label study, the parents/caregivers and an appointed nurse who will perform the randomisation will be aware of treatment allocation, whilst the clinicians and tympanometry technicians will remain unaware of the treatment.

2.2 Participants, interventions, and outcomes

2.2.1 Study setting

Prior to this study, we conducted a feasibility study to survey the current management of AOM in children in three cities in Indonesia and to identify the willingness of physicians to participate in our proposed clinical trial of corticosteroids for AOM in children. Based on clinical scenarios, there were sufficient number of physicians who would prescribe corticosteroids for AOM. There were 171 physicians from 87 primary/secondary to tertiary centres (public and private) in DKI Jakarta, Depok, and Bekasi who were willing to participate in our proposed main study. However, we will only pilot this study at seven public and private hospitals in Jakarta and Bekasi: (1) Dr Cipto Mangunkusumo Hospital; (2) Persahabatan Hospital; (3) Gatot Subroto Army Hospital; (4) Antam Medika Hospital; (5) Cempaka Putih Islamic Hospital; (6) Proklamasi ENT Hospital; and (7) Hermina Bekasi Hospital.

2.2.2 Eligibility criteria

2.2.2.1 Inclusion criteria

We will include 60 children aged six months to 12 years old with AOM, defined as current onset (48 to 72 hours) of AOM-relevant symptoms (e.g. earache, ear tugging/rubbing or irritability in non-verbal children). If it is feasible, otoscopic findings of middle ear effusion (e.g. bulged tympanic membrane, limited or absent mobility of the tympanic membrane, air fluid level, ear discharge) and acute inflammation (e.g. erythema) will confirm the diagnosis.

2.2.2.2 Exclusion criteria

We will exclude children:

1. with major and severe medical conditions (e.g. heart diseases, kidney failure)
2. who are immunocompromised (e.g. HIV, children receiving cancer treatment)
3. with congenital malformations and/or syndromes (e.g. cleft palate, Down's syndrome)
4. with ear ventilation tube(s)

5. exposed to persons with varicella (chicken pox) or active Zoster infection in the past three weeks without prior varicella immunisation or infection
6. who have high risk of strongyloidiasis infections with symptoms and signs of unexplained eosinophilia, skin reaction due to larvae penetration into the skin (e.g. inflammation, oedema, petechiae, severe pruritus), particularly on the feet regions, pulmonary (e.g. dry cough, throat irritation, dyspnoea, wheezing, haemoptysis, repeated episodes of fever and mild pneumonitis), or gastrointestinal symptoms (e.g. upper abdominal pain, diarrhea, anorexia, nausea, epigastric pain, malabsorption, and vomiting)
7. who have taken systemic (i.e. oral, injection) or topical steroids in the preceeding four weeks
8. who have taken antibiotics in the preceeding two weeks
9. who are hypersensitive to prednisolone or prednisone, or other corticosteroids

2.2.3 Interventions

Children in the mild AOM group will be randomly allocated to receive either a single dose of prednisolone tablets daily for five days as an addition to expectant observation or expectant observation alone (without prednisolone). At the baseline visit, the participating physicians will inform the parents/caregivers to closely observe the children for 48 hours without immediate antibiotic treatment.

Children in the severe AOM group will be randomly allocated to receive either a single dose of prednisolone tablets daily for five days as an addition to antibiotics according to physicians' preferences or antibiotic alone (without prednisolone). Antibiotic treatment is commonly prescribed for AOM with severe symptoms. The information regarding the antibiotics (e.g. antibiotic type, dose, duration) will be recorded in the case report forms (CRFs).

2.2.3.1 Prednisolone and control group

Prednisolone group

Prednisolone tablets will be given in the intervention groups in both mild and severe groups. It is given at a dose of 1 mg/kg to 2 mg/kg body weight, once daily for five days. The pharmacist will crush the tablets, mix them with sweeteners, and pack them in a daily paper-pack. It should be given in a single dose at 6 to 8 am in the morning to mimic the normal diurnal rhythm of cortisol production [19,20]. Participating physicians will advise the parents to give the prednisolone at about the same time each day as a daily routine for children which will also help parents to remember.

Control group

Due to budget constraints, we are not able to provide a matched placebo as prespecified for the main study. Therefore, we will not give placebo for children in the control group, either in the mild or severe group. Children in the control group of the mild AOM group will still receive expectant observation and those in the severe AOM group will receive antibiotics, and other concomitant treatment based on physicians' preferences (if necessary). The only difference between the intervention and control group is solely whether they receive prednisolone (intervention group) or not (control group).

2.2.3.2 Criteria for trial drug discontinuation or modification

When giving the prednisolone to a child, parent can mix the prednisolone powder with jelly or juice. If children vomit less than 30 minutes after having a dose of prednisolone, parents should give the same dose again. However, if they vomit again after 30 minutes, parents should not give another dose of prednisolone until the next dose on the next day. This should be noted in the symptom diary. If children keep vomiting after receiving prednisolone, parents should contact the research team. If the parents forget to give prednisolone to their children, they can give the missed dose as soon as they remember on the same day and they should also note this in the symptom diary.

If there are any adverse events and adverse drug reactions which have been assessed by research team that would require the discontinuation of drug trial and further assessment and treatment, then the treatment will be discontinued for this particular case, however follow-up will continue, where possible. This will be reported in the CRFs. Serious adverse events will be reported to Bond University's Human Research Ethics Committee (BUHREC) and the Research Committee Ethics Faculty of Medicine Universitas Indonesia (FMUI) – Dr. Cipto Mangunkusumo Hospital (CMH).

2.2.3.3 Adherence monitoring

Participating physicians will provide information regarding the administration of the prednisolone. The researcher will send a daily text-message reminder to all the parents in both prednisolone and control groups during the intervention period of five days to take the drug regularly and to complete the symptom diary daily. The text-message will also remind all the parents to visit the clinic after the 48-hour expectant observation or visit-1 (day-3), visit-2 (day-7), visit-3 (day-30), and visit-4 (day-90) for re-assessment. At each visit-1 and visit-2, the parents will return the first and second mini-booklets of symptom diary and the left-over drug to the appointed nurse (at visit-2). The nurse will then check the symptom diaries and the left-over drug for the adherence in taking intervention drugs. We will visit the patients' homes to collect the third mini booklet of symptom diary that will record the symptoms from day-7 to day-14 after the baseline visit.

2.2.3.4 Concomitant care and interventions

Physicians may give symptomatic medicine (i.e. ibuprofen, acetaminophen, decongestant, mucolytic) according to their usual practice and these will be recorded in the CRFs and symptom diary. Parents will record the use of these medicine in the symptom diary. The decision on concomitant medication will be made without knowledge of allocation to the prednisolone or control group.

2.2.4 Outcomes

2.2.4.1. The pilot study outcomes

Recruitment rate

Recruitment rate is defined as the proportion of consultations with potentially eligible children who provide their consent to be included in the study. As this is recognised as a crucial aspect of conducting a clinical trial and may cause study discontinuation due to low recruitment [37], we will assess this in our pilot study. We will identify the rates and challenges during the recruitment process and determine the best strategy for the main study to overcome these challenges and obstacles.

The success of the study procedures

The success of the study procedures includes the following: (1) obtaining informed consent from the patients and their parents; (2) the recruitment based prespecified eligibility criteria, including the use of otoscope to diagnose AOM if feasible; and (3) the stratification and randomisation process, including stratifying eligible children based on the severity and obtaining the allocation result whether the children will be allocated to the prednisolone or control group.

Ability to measure planned outcomes in the main study

We will assess the ability to measure planned outcomes in main study, which are: (1) the proportion of children with pain reduced by at least the minimum clinically important amount, at day-3 after randomisation. This will be assessed using a VAS recorded in the patient symptom diary; (2) the severity of pain and other AOM-relevant symptoms at various time points using VAS, AOM-SOS, and the symptom diary; (3) duration to AOM resolution; (4) adverse effects, defined using standard clinical trials criteria, recorded in the diary, and reported to the central office and ethics committee as required.; (5) complications of AOM (e.g. perforation of tympanic membrane(s), mastoiditis); and (6) AOM recurrence, defined as a new episode of AOM at one to three months after randomisation. We will report these narratively due to a limited sample size and insufficient formal power calculation for this pilot study to be able to detect actual effects of corticosteroids to improve clinical outcomes in AOM.

The compliance to study and study drug

The compliance to study and study drug is defined as a proportion of children who regularly take the study drug (assessed using the symptom diary and any left-over drug) and come to follow-up visits per protocol. Participants will be followed-up closely by clinicians and research staff. Children will return for a visit at day-3 after randomisation, ensuring collection of the primary outcome.

The verification of sample size calculation for main study

Based on our size calculation, we plan to enrol 760 children in the main study. We estimate that there will be 35% of the total sample of children with AOM in the severe group (i.e. children with severe symptoms, fever ≥ 39°C, children aged < 2 years with bilateral AOM, AOM with perforation of tympanic membrane). Therefore, within this pilot study, we can assess the accuracy of this assumption. Also, we will check another assumption of the sample size calculation, which was the proportion in the control group with the resolution of pain at three days, which was 42.5%.

2.2.4.2. The mechanistic or tympanometry sub-study outcomes

Primary outcomes

The change in middle ear effusion (MEE) at various time points

We will assess the change in the MEE at the following time points: baseline visit, visit-1 (day-3), visit-2 (day-7), visit-3 (day-30), and visit-4 (day-90). We will measure MEE using static acoustic admittance, defined as “the amount of energy absorbed by the tympanic membrane and middle ear, measured in mmho or mL” [38]. We will also measure the difference of this results between the intervention and control group.

Secondary outcomes

Duration of MEE

We will also assess the duration to the resolution of MEE using tympanometry.

The correlation between ear pain and other symptoms with the changes in MEE at various time points

We will identify the correlation between ear pain and other symptoms (i.e. ear tugging, irritability, crying, lack of sleep, lack of appetite, less of playfulness, fever) with the changes in MEE at various time points.

2.2.5 Participant timeline

Table 1 illustrates the timeline for visits and follow-ups. We will measure the outcomes at various time points: (1) visit-1 after the 48-hour observation (day-3); (2) visit-2 (day-7); (3) visit-3 (day-30); and (4) visit-4 (day-90). Patients will visit the hospital at visit-1 and visit-2, whilst the last two visits will be a home-visit. However, children in the mechanistic sub-study have to visit the hospitals at those follow-up visits. To improve the compliance of the study, the research personnel will send a daily text-message reminder to all the parents in the study during treatment period to take the drug, complete the symptom diary, and visit the clinic after the 48-hour expectant observation and other time points. At visit-2, parents will return the symptom diary and any left-over drug.

Table 1. Follow-up timeline

	**STUDY PERIOD**					
	**Enrolment Allocation**	**Post-allocation**				**Close-out**
**TIMEPOINT**	**0 (Day-0)**	**t1 (Day-3)**	**Intervention ends (Day-5)**	**t2* (Day-7)**	**t3* (Day-30)**	**t4* (Day-90)**
**ENROLMENT:**						
Eligibility screen	X					
Informed consent	X					
Allocation	X					
**INTERVENTIONS:**						
[Intervention A] Prednisolone (5 days)	———	———	———			
[Intervention B] Control (5 days)	———	———	———			
**ASSESSMENTS:**						
Baseline examination (weight, height, BP, body temperature)	X	X		X	X*	X*
Severity of pain and duration using VAS	X	X		X		
Overall symptoms and its duration using AOM-SOS	X	X		X		
Adherence to trial drug	X	X		X		
Adverse effects	X	X		X		
Otoscopic examination	X	X		X	X*	X*
Tympanometry examination	X^{+}	X^{+}		X^{+}	X^{+}	X^{+}
Complication	X	X		X		
Recurrence of AOM					X*	X*

BP=blood pressure; VAS=visual analogue scale; AOM-SOS: acute otitis media-severity of symptoms scale

**The follow-up will be carried out by home-visit and phone, but those in the mechanistic sub-study will have their follow-up visits to the clinics; $^{+}$These time-point assessments will be applied only for children in the mechanistic sub-study*

Participant enrolment

In the main study, we will stratify eligible children by the clinical specialty (primary care or secondary/tertiary care) and severity of AOM (mild or severe). However, we only include ear-nose-throat-specialists (ENTs) who work at tertiary care in this pilot study. Therefore, we will stratify the children based on their AOM severity and then will randomly allocate these eligible children to receive either a single dose prednisolone for five days or without, as an addition to expectant

observation in the mild AOM group or as an addition to antibiotics according to physicians' preferences (e.g. antibiotic type, dose, duration) in the severe AOM group (see Figure 1).

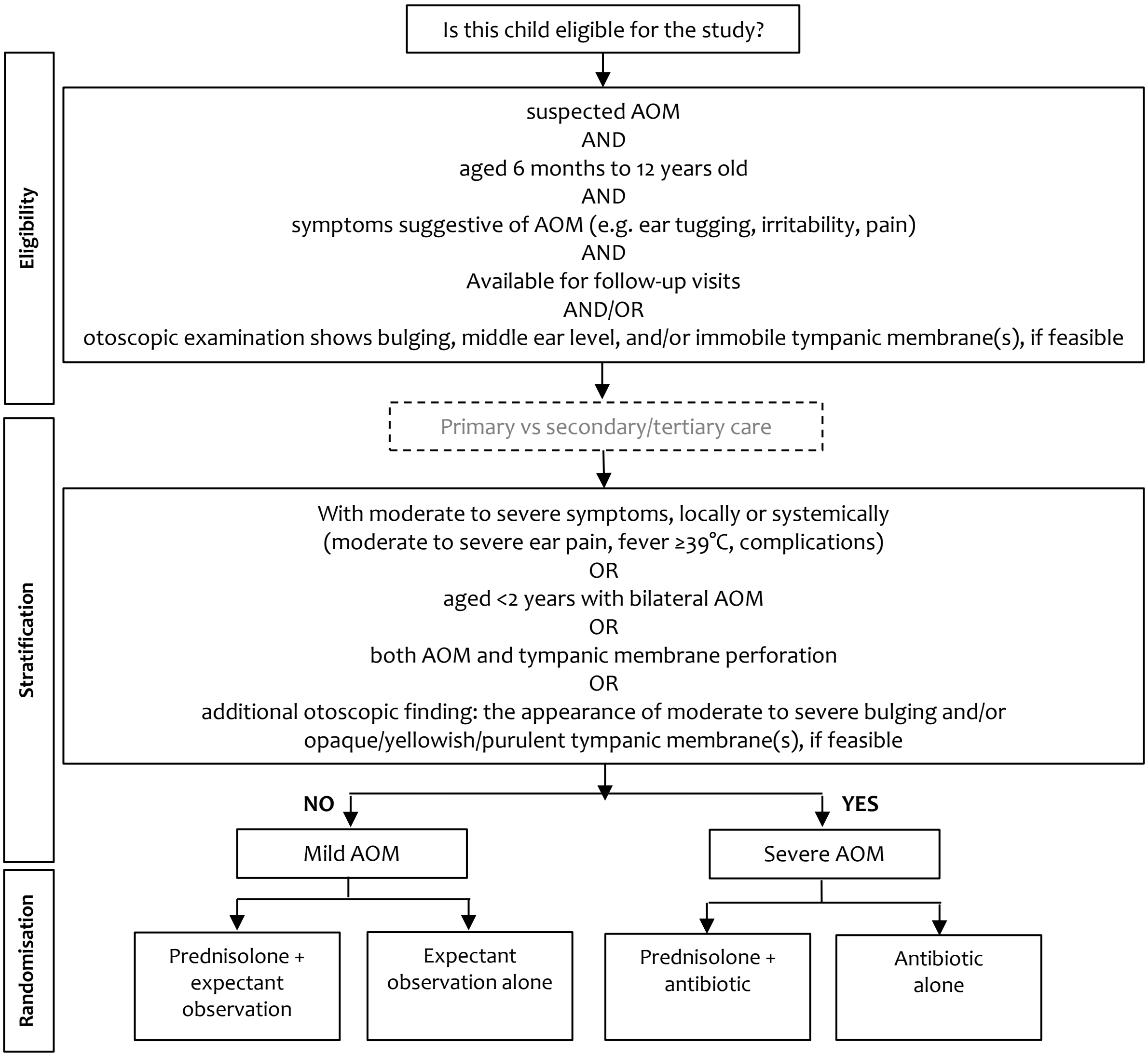

Figure 1. Flow chart of the stratification and randomization of the study

Participating physicians will assess the eligibility of children who come to the hospital based on their symptoms and clinical features of AOM. The eligible children will then be stratified based on the severity of AOM to either mild or severe AOM group. This process will be assisted using an eligibility and randomisation form. The participating clinicians will then obtain a clinical history and perform a baseline examination, including general, otoscopic, and tympanometry examination. The results of these procedures will be recorded at the baseline history form and outcomes form. The tympanometry examination will be conducted by an audiologist or a tympanometry technician. The physician will assess and analyse the tympanometry findings and record the results in the outcome form. After these procedures, the children will be sent to the appointed nurse who will perform the randomisation and dispense the prescription for a study medication (prednisolone) if the patient is assigned to the intervention group.

2.1.6 Sample size

We did not determine a sample size for the pilot study. There are several suggestions in calculating the sample size for a pilot study (e.g. at least 55 participants or at least 9% of the sample size of the main study) [39]. Since we will need 60 children in the mechanistic sub-study, we will include 60 children with AOM in our pilot study. The sample size of the mechanistic study was determined based on the main primary outcome, which is the mean value of static acoustic admittance or acoustic compliance in the tympanogram. In a previous study of children with middle ear effusion (MEE) who underwent tympanometry assessment and had a history of chronic or recurrent middle ear disease [40], the response within each subject group was normally distributed with standard deviation 0.3. If the true difference in the experimental and control means is 0.3 units, we will need to study 22 experimental subjects and 22 control subjects to be able to reject the null hypothesis that the population means of the experimental and control groups are equal with probability (power) 0.9. The Type I error probability associated with this test of this null hypothesis is 0.05. With a 20% allowance for dropouts, the total sample size becomes 56 or we will include 60 children for this pilot study.

Using the worst-scenario that of 50 physicians who work at seven appointed hospitals and have approximately 97 patients with AOM per week, we estimate that only 30% of the physicians will confirm their participation and 25% of the patients will give their consents to participate in our study. We then estimate that it will require nine months to recruit 60 children with AOM in this pilot study, including the extra months for potential slow recruitment and last follow-up (three months after the baseline visit).

2.1.7 Recruitment

Recruitment of physicians

Our feasibility survey study (April–August 2016) demonstrated there were 352 physicians (general practitioners, ear-nose-throat specialists, and paediatricians) who were willing to participate in our main study and they had 705 paediatric patients with AOM in the past seven days. For our pilot study, we identified 50 physicians from the most feasible seven hospitals located in DKI Jakarta who were willing to participate in our study and had approximately 97 paediatric patients with AOM in a week.

Recruitment of participants

After physicians deliver the patient information and obtain the consent from the eligible children, they will use the eligibility and randomisation form to assist them in stratifying the children based on their AOM severity. Physicians then will complete other CRFs, as the following: baseline history form and outcome form. Physician will assist the parents to complete the symptom diary. The similarity on the forms and sequences of both outcome CRF and symptom diary will help parents to be able to fill those correctly.

2.3 Assignment of interventions

2.3.1 Allocation

Sequence generation and implementation

All children and their parents who are eligible and consented will be enrolled, and stratified based on their AOM severities or clinical features. The randomisation process will be performed by the

appointed nurse who will randomly allocate children to either prednisolone and expectant observation or control (expectant observation alone) in the mild group and either antibiotic with prednisolone or control (antibiotic alone) in the severe group. A permuted block randomisation sequence will be computer-generated by the Centre for Research in Evidence-Based Practice Bond University, Gold Coast, Australia. Random numbers will not be disclosed to the outcome assessors (i.e. participating clinicians and audiologists/tympanometry technicians), to ensure allocation concealment. Batches of intervention packages will be dispatched to participating centres from a central pharmacy facility at the Clinical Research Supporting Unit, Faculty of Medicine Universitas Indonesia (CRSU FMUI).

Allocation concealment mechanism and implementation

The information of the eligibility and stratification which is provided by the participating physicians will help the appointed nurses to obtain the information from the randomisation website, whether the children will be allocated to the prednisolone or control group, identified by the 2-digit patient ID numbers. During the consultation, the physician will prescribe study medication for every subject with the dose based on the patient age and insert the prescription in the CRF folder. The nurse who performs the randomisation will give the prescription to the subjects who are allocated to the intervention group (prednisolone group). The subject then will give the prescription to the pharmacy, where the pharmacist will prepare the prednisolone by crushing the tablets, mixing them with sweeteners, packing the prednisolone mixed powder in a daily individual paper-pack for five days, and dispense these to the subjects along with instructions for preparation. The pharmacist will record the dispensing on the form provided by the study for this purpose.

2.3.2 Blinding (masking)

In this study, the appointed nurses and the children and their parents will know the allocation of the intervention. We will ensure that the participating physicians and the audiologists/ tympanometry technicians will be blinded to the intervention allocation during the study.

Emergency unblinding

The unblinding process should be done if there are serious adverse events and limited only to that particular participating physician.

2.4 Data collection, management, and analysis

2.4.1 Data collection methods

We will assess the outcomes using CRFs, patient symptom diary, and feedback forms. The CRFs consist of eligibility-and-randomisation, baseline history, outcomes, serious adverse events (if applicable), and drug dispensing and returned forms. The outcome form will record the severity of pain and overall symptoms with their durations using VAS and AOM-SOS, as well as the resolution of AOM signs using otoscopic and tympanometry examinations (for the mechanistic study).

We will identify the recruitment rate by assessing the proportion of children who provide their consents divided by the proportion of consultations with potentially eligible children during the trial. We will use the informed consent and a study recruitment log book to record the reason(s) why children were not randomised.

We will assess the success of the study procedures using feedback forms. On the feedback form, patients and their parents and participating physicians will rate their understanding and challenges

they have during the implementation of study procedures (e.g. obtaining the randomised 2-digit patient ID numbers and their allocation of the treatment, completing the informed consent forms and CRFs) using grading scale ranged from one to five (1=very easy; 2= easy; 3=moderate/neutral; 4=difficult; 5=very difficult).

To assess the ability to measure planned outcomes in main study, we will also use a feedback form to identify the understanding, the challenges, the complexity of the outcome assessment tools utilised for this study (e.g. CRFs and patient symptom diary which includes VAS and AOM-SOS) from the perspective of the patients and their parents and the participating physicians. The CRFs and symptom diary will record the clinical history and symptoms (e.g. VAS, AOM-SOS), as well as physical examination (e.g. temperature, blood pressure, otoscopic examination if feasible). This information will be obtained from the perspectives of the patients and their parents and the clinicians.

The VAS is acknowledged as a well-established and validated scale for assessing pain [41]. It has a 100-mm horizontal scale with 'no pain' anchor at the left endpoint and 'the most severe pain' at the right endpoint of the scale. The patient will mark a vertical line along the horizontal line as the representation of their pain level. The scale will be determined by measuring the distance from the left endpoint ('no pain') to the marked line [42]. A 10-mm difference has been reported to indicate a clinically significant change [43,44]. The AOM-SOS is used to assess the severity of other AOM-relevant symptoms daily. particularly in non-verbal children. Table 2 illustrates several AOM-related symptoms described as "no", "a little", and "a lot". This scale was developed as an outcome reporting tool scoping symptom and activity limitation due to AOM in the proceeding 12 to 24 hours [45]. Shaikh et al. [45] used the mean of 4.2 points as a minimal important difference. We have translated the original English) version of AOM-SOS to Indonesian version of AOM through forward and backward translation process.

Table 2. Acute otitis media severity of symptoms scale (AOM-SOS) [45]

We are interest finding out how your child has been doing. For each question, please place a check mark in the box corresponding to your child's symptoms. Please answer all questions

	No	A Little	A Lot
Over the past 12 h, has your child been tugging, rubbing, or holding the ear(s) more than usual?	☐	☐	☐
Over the past 12 h, has your child been crying more than usual?	☐	☐	☐
Over the past 12 h, has your child been more irritable or fussy than usual?	☐	☐	☐
Over the past 12 h, has your child been having more difficulty sleeping than usual?	☐	☐	☐
Over the past 12 h, has your child been less playful or active than usual?	☐	☐	☐
Over the past 12 h, has your child been eating less than usual?	☐	☐	☐

Over the past 12 h, has your child been having fever or feeling warm to touch?	☐	☐	☐

The compliance to the study and study drug will be identified by assessing the completion of CRFs and symptom diary, particularly on the attendance of patients and the parents on their scheduled follow-up visits, the completion of the trial drug based on the symptom diary and the left-over drug. We will identify the parents who have low literacy during the informed consent process. For parents who have low literacy, we will visit their home one day after the initial visit to identify a person who lives nearby (e.g. family members, neighbours) that are able to assist the parents in completing the symptom diary, daily for two weeks. If there is noone can assist them, then the research personnel will assist them in completing the symptom diary by phone. If it is not feasible, we will visit their home daily to be able to assist them.

To assess the verification of sample size calculation for main study, we will use the CRFs.

To ensure that all the outcome data can be sufficiently collected and recorded properly according to prespecified plans, we will conduct an individual/institutional training for participating physicians prior the implementation of this pilot study. The training will include following sections: (1) introduction and summary of the study; (2) introduction of international clinical practice guidelines of AOM; (3) The diagnosis of AOM using otoscope and reporting the otoscopic results; (4) the introduction and dissemination of the principles of quality methodology clinical trial (e.g. eligibility, randomisation, blinding, outcome assessment) and good clinical practice (e.g. patient consents, confidentiality, data management); (5) practical steps of eligibility assessment, stratification, and randomisation; and (6) practical steps in completing study documentation (i.e. patient informed consents, CRFs, feedback forms, patient symptom diary).

2.4.2 Data management

The integrity and completion of data will be maintained through mechanisms such as consistency checks during data entry, and cross-checks between items after data entry. All the actions and modifications to data stored in the database will be documented and retrievable for viewing. Missing data or errors will be detected before final submission to the electronic database and will be recorded in a summary along with the descriptions for each missing and/or error data. The summary will then be notified to the co-investigators in that hospital for further investigation by checking and confirming the original forms or other resources for correction or completion for those with missing and/or erroneous data. The modification to original forms will be done by research personnel at that hospital and will be documented on paper and electronic versions. It will be annotated with the date, name, and signature of the person who is responsible for making modifications.

The central data coordinator will check the validity and completeness of study data on a regular basis. All data in the central database will be protected with a regular complete back up system.

2.4.3 Statistical methods

For the recruitment rate, we report the outcome as the proportion of children in percentages (%). For the success of the study procedures and the ability to measure planned outcomes in main study, we will report the outcomes as the proportion of clinicians in percentages based on the grading

scale of their feedback reporting on prespecified outcome measure tools. For the compliance to study and study drug, we will report the outcomes as the proportion of children in percentages who attend the follow-up visits and complete the cycle of study drug.

To assess the verification of sample size calculation for main study, we will report this outcome as the proportion of children in each stratum (mild and severe AOM group) and those with pain at Day-3 after randomisation in the control group.

For the mechanistic sub-study, we will report continuous variables (i.e. the change in MEE at various time points (mean in days standard deviation), the duration of MEE) as a mean difference (MD) with 95% confidence intervals (CI) also the difference between two groups. We also will report the correlation between ear pain and other symptoms with the changes in MEE at various time points.

2.5 Monitoring

2.5.1 Data monitoring

Data monitoring committee

Since this is a short study, we do not need data monitoring committee for this pilot study. However, independent personnel from Clinical Epidemiology and Evidence-Based Medicine (CEEBM) Unit, Dr Cipto Mangunkusumo Hospital (CMH) – Faculty of Medicine Universitas Indonesia (FMUI), who is not involved in this study, will assess the process and the quality of patient recruitment, data entry, and a compilation of research data in central database. Her feedback will be important to improve the implementation of our main study.

Interim analysis

Due to the small number of recruited patients to the trial and the duration of the trial will be lesser than one year, we will not perform an interim analysis.

2.5.2 Harms

An adverse event is defined as any untoward medical occurrence in a patient or clinical investigation subject administered a pharmaceutical product and which does not necessarily have to have a causal relationship with trial drug; whereas adverse effects or adverse drug reaction, is defined as all noxious and unintended responses to a trial drug related to any dose.

At the Visit-2 (Day-3), the appointed nurse is the first person who will identify any adverse effects (AEs). These will be identified by history taking (e.g. interview) and the symptom diary. The parents will provide information in the symptom diary regarding the adverse effects and whether they seek any treatment or medical assistance to manage AEs. The nurse then will report the adverse effects to the physicians along with the information of the intervention group which the child was allocated to. This information will be provided after the physician assess and record the primary outcome (i.e. ear pain three days after the randomization) on the 'Outcome form'. This is important to keep the concealment.

Adverse events and adverse drug reaction will be collected after they sign the written consents and being enrolled in the trial. All adverse events occurring after the enrolment into the study, during the additional treatment or hospitalization due to adverse events and/or ADR will be

recorded. A subject who experiences a serious adverse event (SAE), defined as any untoward medical occurrence at any dose that may result in-patient and/or prolonged hospitalization, persistent or significant disability, medically important events, life threatening events, and death, will receive sufficient treatment and will be recorded and reported to the Research Committee Ethics FMUI – CMH and the Bond University's Human Research Ethics Committee (BUHREC). We will responsible for any additional examinations and/or treatment that are required to manage AEs.

We will not report SAE occurring after the trial discontinuation, unless there is a temporal relationship between trial drugs or other protocol procedure to the events, as well as whether the event is unexpected or unexplained given the subject's clinical course, previous medical conditions, and concomitant medications. All the SAE will be recorded in SAE form.

2.5.3 Auditing

For the main study, we will establish an audit committee from the CRSU FMUI and CEEBM Unit CMH-FMUI which is independent from the trial investigators and the funding body. However, this will not occur separately from monitoring for the pilot study. This independent committee will conduct monitoring of source paper and electronic documents in the website system, monitor the conduct of trial in multicentre sites, interviewing the investigators and coordinators, and check the storing, distribution, and the use of trial drugs. At the start of the trial, the committee will ensure that the research staff are capable in data entry and in using the website system. Observation and quality assessment of the whole trial will be ensured to be always in accordance with the protocol and International Conference Harmonization – Good Clinical Practice (ICH-GCP) standards.

2.6 Ethics and dissemination

2.6.1 Research ethics approval

This study will be conducted according to the Declaration of Helsinki and ICH-GCP guidelines. We will seek ethics approval from: (1) the Bond University's Human Research Ethics Committee (BUHREC) Bond University, Queensland, Australia; (2) the Medical Ethics Committee of the Faculty of Medicine Universitas Indonesia – Dr. Cipto Mangunkusumo General Hospital, Jakarta, Indonesia; (3) the Directorate-General for Politics and General Government – The Ministry of Internal Affairs Republic Indonesia; (4) the Health Agency for the Province of DKI Jakarta; and (5) local research committee at each participating hospital.

2.6.2 Protocol amendments

Any modifications to the protocol which may impact on the trial process (e.g. the modification of study objectives, study design, study population, sample sizes, the procedures, and significant administrative sectors), potential benefits and harms/safety of the patients will require a formal amendment to the protocol. This amendment will be notified and approved by the funding body and the Ethics committee prior to its implementation. Notification is also applied to the health authorities in accordance with local regulations. Minor modifications that may not impact on the trial process will also be notified and approved by the funding body and will be notified to The Ethics Committee.

2.6.3 Consent

The participating physician will provide patient information sheet and obtain informed consent from the parent(s) or legal guardian of patients, before conducting the recruitment and

randomisation process. In obtaining the consent, the investigators will inform the trial process including known and potential risks from the trial. As children are considered a vulnerable population, those aged younger than 12 years old are considered not competent to give research consent and the parent or their legal guardian will make the decision. The parent can also make decision for children aged 12 years, however there should be assent from children to participate in the research. For parents who are illiterate, we will identify the literacy level before we start the informed consent session and will ensure that the language used in the oral and written information about the trial, including the written informed consent form, should be understandable to the parents and justified to their literacy level. During the process, the participating physician will confirm the participant's understanding of the process of the trial, including the risks and benefits. For particularly crucial topics, the participating physician will ask several questions related to that topic to ensure they are well-informed and understand. Gaining informed consent will be a major topic discussed during training sessions for staff.

The person who delivers the consent (i.e. participating physician, research assistant) also will provide their signatures on the consent form, stating that they have provided information and opportunity for potential participants to understand and raise relevant questions according the trial. We will ensure that the consent process is free of coercion. As the participation into the trial is voluntary, we will emphasise their rights to withdraw from the trial at any time without any consequences, particularly on the quality of their healthcare services.

2.6.4 Confidentiality

All information related to the trial will be stored securely at the study site and the research office. All participant information will be stored in locked file cabinets in areas with limited access. All data collection, including CRFs, test results, and administrative forms will be kept confidential by only using coded IDs as identifiers and will be stored separately from all forms and records that contain names or other identifiers (e.g. informed consents forms). All databases will be secured with limited access using password-protected access systems. All counselling sessions and general to specific examinations (e.g. ear, nose, throat examination, otoscopic and tympanometry examination) will be conducted in private rooms in the participating physicians' clinics or hospital. All the involved research staffs such as physicians, nurses, and audiologists will be required to sign agreements to preserve the confidentiality of all participants.

The confidentiality of every participant will be maintained and will not be distributed externally without the written permission of the participant, except as necessary for trial monitoring by national regulatory authorities related to the medical and research safety.

2.6.5 Declaration of interests

Respati W. Ranakusuma (RWR) has nothing to disclose.
Amanda McCullough (AMC) has nothing to disclose.
Elaine M. Beller (EMB) has nothing to disclose.
Christopher Del Mar (CDM) has nothing to disclose.
Eka Dian Safitri (EDS) has nothing to disclose.
Yupitri Pitoyo (YPO)has nothing to disclose.
Widyaningsih (WID) has nothing to disclose.
Arie Sulistyowati (ARS) has nothing to disclose.

Sudigdo Sastroasmoro (SSO) has nothing to disclose.

2.6.6 Access to data

The Principal Investigator will be given access to the cleaned data sets. She will also have direct access to each sites' data sets and by request. Project data sets will be secured using password. To ensure confidentiality, data dispersed to project team members will be blinded of any identifying participant information.

2.6.7 Ancillary and post-trial care

Short-term corticosteroids are very unlikely to have harm outside those we will be measuring. However, we will responsible for the adverse effects that occurring from the trial drug during the trial (immediate) and post-trial (ancillary care) related to trial drug. The compensation will include the treatment cost relevant with the trial drug, such as the consultation visit, additional examinations, and treatment (e.g. medicine, hospitalization cost). Due to other potential concurrent treatments within the drug trial, there will be robust review and analysis process to conclude the cause of adverse events. Participating physicians will explain the procedure for the management of adverse effects of trial drug during the process of consent approval before entering the trial. We will also include this information on the patient symptom diary, including the 24-hour emergency call and list of recommended healthcare providers.

2.6.8 Dissemination policy

2.6.8.1 Trial results

Trial results, either statistically significant or non-significant, and other components of the trial (literature review, survey study, pilot study, etc.) will be reported in a journal manuscript after being distributed to all the principal investigators to be reviewed.

2.6.8.2 Authorship

The authorships and contributions of this trial will be acknowledged on the protocol, manuscript, and the report. Before the publication in medical journal or paper presentation, the principal investigators (PIs) will provide written consent of their acknowledgment and contribution in the reported trial.

- Respati W Ranakusuma contributes in: (1) designing and developing the protocol; (2) conducting the trial; and (3) interpreting and reporting the trial in the final trial report and manuscript for publication.
- Amanda McCullough contributes in: (1) the protocol development (study design and methods); (2) interpreting the results; and (3) the writing process of the final trial report and manuscript for publication.
- Elaine M Beller contributes in: (1) the protocol development (study design, methods, and statistics); (2) interpreting the results; and (3) the writing process of the final trial report and manuscript for publication.
- Chris Del Mar contributes in: (1) the protocol development (study design, methods, and statistics), and (2) the writing process of the final trial report and manuscript for publication

- Sudigdo Sastroasmoro contributes in: (1) supervising the conduct of the trial in Indonesia; (2) interpreting the result; and (3) the writing process of the final trial report and manuscript for publication.
- Eka Dian Safitri contributes in: (1) developing the mechanistic study using tympanometry; (2) providing training related to using and interpreting tympanometry to physicians, nurses, and tympanometry technicians; (3) interpreting the tympanometry findings; and (4) reviewing the final study report and manuscript for publication
- Yupitri Pitoyo contributes in: (1) recruiting secondary and tertiary healthcare centres including the physicians and nurses; (2) interpreting the tympanometry findings; and (3) reviewing the final study report and manuscript for publication
- Widyaningsih contributes in: (1) submitting a research ethics application to the Medical Research Ethics Committee in Indonesia; (2) submitting clinical trial permits to Indonesian institutions; (3) supporting the data collection and management; and (4) reviewing the final study report and manuscript for publication
- Arie Sulistyowati contributes in: (1) providing advice and expertise in terms of paediatric patients; (2) supporting the data analysis; and (3) reviewing the final study report and manuscript for publication

2.6.8.3 Reproducible research

We will make the full protocol of this study to be publicly available to maintain its transparency and reproducibility. This full protocol will include detailed information regarding the study, particularly on study design and conduct that not are commonly include in the published protocol or information description in clinical trial registry. We will register the protocol into trial registry such as the Indonesia registry web portal (https://www.ina-registry.org/) and Australian New Zealand Clinical Trials Registry (http://www.anzctr.org.au/). We also will publish the results of this study in relevant medical journal as two separate papers as the following: (1) results of the pilot study and (2) results of the mechanistic sub-study. If necessary, we will include the anonymised participant-level dataset in its appendix or online. Unpublished outcomes will be reported in the full trial report that will be linked to the published study.

APPENDICES

Appendix 1. Flow chart of patient during the baseline and other visits
Appendix 2. Patient information and consent form
Appendix 3. Case report forms
Appendix 4. Study recruitment log book
Appendix 5. Patient symptom diary
Appendix 6. Product (Lupred®) information summary

REFERENCES

1. Antibiotic resistance threats in the United States. US Department of Health and Human Services: Centers for Disease Control and Prevention. 2013.
2. The World Health Organization. Global action plan on antimicrobial resistance. Geneva. 2015. ISBN 9789241509763.
3. Harris AM, Hicks LA, Qaseem A. Appropriate antibiotic use for acute respiratory tract infection in adults: Advice for high-value care from the American College of Clinicians and the Centers for Disease Control and Prevention. *Ann Intern Med*. 2016;164:425-34.
4. Pettigrew MM, Gent JF, Pyles RB, Miller AL, Nokso-Koivisto J, Chonmaitree. Viral-Bacterial Interactions and Risk of Acute Otitis Media Complicating Upper Respiratory Tract Infection. *J Clin Microbiol*. 2011;49(11):3750–5.
5. Chonmaitree T, Revai K, Grady JJ, Clos A, Patel JA, Nair S, et al. Viral upper respiratory tract infection and otitis media complication in young children. *Clin Infect Dis*. 2008;46(6): 815–23.
6. Umar S, Restuti RD, Suwento R, Priyono H, Mansyur M. The prevalence and risk factors of acute otitis media in children in the municipality of East Jakarta [Prevalensi dan faktor risiko otitis media akut pada anak–anak di kotamadya Jakarta Timur]. http://lib.ui.ac.id/naskahringkas/2015-09/SP-Sakina%20Umar. Published 2013. Accessed February 20, 2016.
7. Britt H, Miller GC, Henderson J, Bayram C, Valenti L, Harrison C, et al. A decade of Australian General Practice Activity 2005-06 to 2014-15. General practice series no. 39. Sydney: Sydney University Press; 2015.
8. Lieberthal AS, Carroll AE, Chonmaitree T, Ganiats TG, Hoberman A, Jackson MA et al. Clinical Practice Guideline: The diagnosis and management of acute otitis media. The American Academy of Pediatrics. *Pediatrics.* 2013;131:e964-e99.
9. Morris PS, Leach AJ. Managing otitis media: an evidence-based approach. *Aust Prescr.* 2009;32:155–9.
10. Le Saux N, Robinson JL, Canadian Paediatric Society Infectious Diseases and Immunization Committee. Management of acute otitis media in children six months of age and older. *Paediatr Child Health*. 2016;21(1):39-44.
11. Rovers MM, Glasziou P, Appelman CL, Burke P, McCormick DP, Damoiseaux RA, et al. Antibiotics for acute otitis media: a meta-analysis with individual patient data. *Lancet*. 2006;368:1429-35
12. Ministry of Health Republic of Indonesia. Clinical practice guidelines for clinicians in primary healthcare centres. Jakarta: Ministry of Health Republic of Indonesia;2014. Regulatory No. 5 year 2014.
13. Costelloe C, Metcalfe C, Lovering A, Mant David, Hay AD. Effect of antibiotic prescribing in primary care on antimicrobial resistance in individual patients: systematic review and meta-analysis. *BMJ.* 2010;340:c2096. doi: 10.1136/bmj.c2096
14. Venekamp RP, Sanders SL, Glasziou PP, Del Mar CB, Rovers MM. Antibiotics for acute otitis media in children. Cochrane Database of Systematic Reviews 2015, Issue 6. Art. No.: CD000219. DOI: 10.1002/14651858.CD000219.pub4.
15. Marom T, Marchisio P, Tamir SO, Torretta S, Gavriel H, Esposito S. Complementary and alternative medicine treatment options for otitis media. *Medicine*. 2016;95(6):e2695

16. Coleman C, Moore M. Decongestants and antihistamines for acute otitis media in children. Cochrane Database of Systematic Reviews 2011, Issue 3. Art. No.: CD001727. DOI: 10.1002/14651858.CD001727.pub5.
17. Juhn SK, Jung MK, Hoffman MD, Drew BR, Preciado DA, sausen NJ, et al. The role of inflammatory mediators in the pathogenesis of otitis media and sequelae. *Clin Exp Otorhinolaryngol*. 2008;1(3):117-38.
18. Chonmaitree T, Saeed K, Uchida T, Heikkinen T, Baldwin CD, Freeman DH, et al. A randomised, placebo-controlled trial of the effect of antihistamine of corticosteroid treatment in acute otitis media. *J Pediatr*. 2003;143:377-85.
19. Schimmer BP, Funder JW. ACTH, adrenal steroids, and pharmacology of the adrenal cortex. In: Brunton LL, Chabner BA, et al., eds. *Goodman & Gilman's The Pharmacological Basis of Therapeutics*. 12th Ed. New York: McGraw-Hill; 2011:1209-36.
20. Gupta P, Bhatia V. [Symposium on steroid therapy] Corticosteroid physiology and principles of therapy. *Indian J Pediatr.* 2008;75(10):1039-44.
21. Indonesian Pediatric Society. Juvenile rheumatoid arthritis. In: Pudjiadi AH, Hegar B, Handryastuti S, et.al, eds. Clinical guideline Indonesian Pediatric Society. 2nd edition. Jakarta; Indonesian Pediatric Society Publishing; 2011:1-4.
22. Indonesian Pediatric Society. Acute bronchial asthma. In: Pudjiadi AH, Hegar B, Handryastuti S, et.al, eds. Clinical guideline Indonesian Pediatric Society Jakarta; Indonesian Pediatric Society Publishing;.2009:269-73.
23. Indonesian Pediatric Society. Bacterial meningitis. In: Pudjiadi AH, Hegar B, Handryastuti S, et.al, eds. Clinical guideline Indonesian Pediatric Society Jakarta; Indonesian Pediatric Society Publishing; 2009:189-92.
24. British Thoracic Society/Scottish Intercollegiate Guidelines Network. British guideline on the management of asthma: a national clinical guideline. British Thoracic Society/Scottish Intercollegiate Guidelines Network. Revised edition; 2016:107.
25. Global Initiative for Asthma. Global Strategy for Asthma Management and Prevention, 2016. www.ginasthma.org. Updated 2016. Accessed September 2, 2016.
26. Ruohola A, Heikkinen T, Jero J, Puhakka T, Juvén T, Närkiö-Mäkelä M, et al. Oral prednisolone is an effective adjuvant therapy for acute otitis media with discharge through tympanostomy tubes. *J Pediatr*. 1999;134:459-63.
27. McCormick DP, Saeed K, Uchida T, Baldwin CD, Deskin R, Lett-Brown MA, et al. Middle ear fluid histamine and leukotriene B4 in acute otitis media: effect of antihistamine or corticosteroid treatment. *Int J Pediatr Otorhinolaryngol.* 2003;67(3):221–30.
28. Waldron CA, Thomas-Jones E, Cannings-John R, Hood K, Powell C, Roberts A, et al. Oral steroids for the resolution of otitis media with effusion (OME) in children (OSTRICH): study protocol for a randomised controlled trial. *Trials*. 2016;17:115.
29. Melhus A, Ryan AF. Expression of cytokine genes during pneumococcal and nontypeable haemophilus influenzae acute otitis media in rat. *Infection and Immunity*. 2000;68(7):4024-31.
30. Aljebab F, Choonara I, Conroy S. *Arch Dis Child* Published Online First: 3 February 2017. DOI:10.1136/archdischild-2015-309522.
31. Panickar J, Lakhanpaul M, Lambert PC, Kenia P, Stephenson T, Smyth A, et al. Oral prednisolone for preschool children with acute virus-induced wheezing. *N Engl J Med*. 2009;360(4):329-38.
32. Scarfone RJ, Fuchs SM, Nager AL, Shane SA. Controlled trial of oral prednisone in the emergency department treatment of children with acute asthma. *Pediatrics*. 1993;92(4):513-8.
33. Scarfone R J, Loiselle JM, Wiley JF II, Decker JM, Henretig FM, Joffe MD. Nebulized dexamethasone versus oral prednisone in the emergency treatment of asthmatic children. *Ann Emerg Med*. 1995;26:480-6.
34. Kayani S, Shannon DC. Adverse behavioural effects of treatment for acute exacerbation of asthma in children. A comparison of two doses of oral steroids. *Chest*. 2002;122:624-8.

35. Chang AB, Sloots TP, Petsky HL, Thearle D, Champion AA, Wheeler C, et al. A 5- versus 3-day course of oral corticosteroids for children with asthma exacerbations who are not hospitalised: a randomised controlled trial. *MJA*. 2008;189(6):306-10.
36. Ranakusuma RW, Pitoyo Y, Safitri ED, Thorning S, Beller EM, Sastroasmoro S, et al. Systemic corticosteroids for acute otitis media in children. *Cochrane Database of Systematic Reviews* 2016, Issue 7. Art. No.: CD012289. DOI:10.1002/14651858.CD012289.
37. Denhoff ER, Milliren CE, de Ferranti SD, Steltz SK, Osganian SK. Factors Associated with Clinical Research Recruitment in a Pediatric Academic Medical Center—A Web-Based Survey. PLoS ONE. 2015;10(10): e0140768. doi:10.1371/journal. pone.0140768.
38. Rosenfeld RM, Shin JJ, Schwartz SR, Coggins R, Gagnon L, Hackell JM, et al. Clinical practice guideline: Otitis media with effusion (update). *Otorlaryngology-Head and Neck Surgery.* 2016;154(1S):S1-S41.
39. Cocks K, Torgerson DJ. Sample size calculations for pilot randomized trials: a confidence interval approach. *J Clin Epidemiol.* 2013;66:197-201.
40. Nozza RJ, Bluestone CD, Kardatzke D, Bachman R. Identification of middle ear effusion by aural acoustic admittance and otoscopy. *Ear Hear*. 1994;15:310-23.
41. Huguet A, Stinson JN, McGrath PJ. Measurement of self-reported pain intensity in children and adolescents. *J Psychosom Res*. 2010;68:329-36.
42. Cohen LL, Lemanek K, Blount RL, et al. Evidence-based assessment of paediatric pain. *J Pediatr Psychol*. 2008;33(9):939-56.
43. Von Baeyer C. Children's self-report of pain intensity: What we know, where we are headed. *Pain Res Manag.* 2009;14(1):39-45.
44. Powell CV, Kelly AM, Williams A. Determining the minimum clinically significant difference in visual analogue pain score for children. *Ann Emerg Med.* 2001;37(1):28-31.
45. Shaikh N, Hoberman A, Paradise JL, et al. Responsiveness and construct validity of a symptom scale for acute otitis media. *Pediatr Infect Dis J*. 2009:28(1):9–12.
