# Supplementary material for: Oral prednisolone for acute otitis media in children: protocol of a pilot randomised, open-label, controlled study (OPAL study)
Source: Pilot Feasibility Stud. 2018 Sep 10;4:146. doi: 10.1186/s40814-018-0337-x (PMC6130070; doi:10.1186/s40814-018-0337-x)
Supplement: Supplementary file 2 — Case report forms—Pilot OPAL Study. This file includes case report forms that are used in the pilot study: CRF01. Participant information sheet and consent form; CRF02. Study registration form; CRF03. Eligibility form; CRF04. Baseline information form; CRF05. Outcome form; CRF06. Symptom diary; CRF07. Prescription of study medication; CRF08. Randomisation form; CRF09. Follow-up visit card; CRF10. Serious adverse events reporting form; CRF11. Feedback form; FORM01. Study recruitment log book; FORM02. Study medication stock book; FORM03. Study medication dispensing form; FORM04. Study medication return form; FORM05. Completed case report form; FORM06. Recapitulation of non-participating subject form; FORM07. Guideline of antibiotics for acute otitis media; FORM08. Prednisolone dose for OPAL study; FORM09. Instruction for using prednisolone for parents; FORM10. Lupred pharmaceutical brochure. This file can be accessed at https://pure.bond.edu.au/ws/portalfiles/portal/27513684/Additional_File_2._Case_report_forms_Pilot_OPAL_Study.pdf (PDF 4716 kb) [file 40814_2018_337_MOESM2_ESM.pdf]

CRF01. PARTICIPANT INFORMATION SHEET AND CONSENT FORM

Oral prednisolone for acute otitis media in children: a pilot pragmatic randomised open-label single-blind controlled study (OPAL study)
[Steroids for middle ear infection in children]

Invitation

You are invited to participate in a research study into the use of steroids (prednisolone) or an anti-inflammatory drug for middle ear infection in children.

The study is being conducted by Dr. Respati W. Ranakusuma, an otorhinolaryngologists and a researcher at the Clinical Epidemiology and Evidence-Based Medicine (CEEBM) Unit Dr. Cipto Mangunkusumo Hospital–Faculty of Medicine Universitas Indonesia. This is part of an international collaborative study between CEEBM CMH-FMUI and the Centre for Research in Evidence-Based Practice (CREBP), Faculty of Health Sciences and Medicine Bond University, Queensland, Australia.

Before you decide whether or not you wish to participate in this study, it is important for you to understand why the research is being done and what it will involve. Please take the time to read the following information carefully and discuss it with others if you wish.

1. **What is the purpose of this study?**
The purpose is to investigate whether steroids, as an alternative treatment, will reduce ear pain and other symptoms in children with acute or recent (less than 48 hours) middle ear infection. This study is part of a doctoral project at the CREBP Bond University, Queensland, Australia. As this is a pilot study, we also want to know your experience during the study. For example, the obstacles you found in giving the steroid to your child or completing the symptom diary daily.

2. **Why have my child and I been invited to participate in this study?**
Your child and you have been invited to participate in this study because your child age ranges between six months to 12 years and having symptoms and signs of acute middle ear infection, such as ear pain in the past 48 hours, or holding or tugging her/his ear more frequently, more irritable, show lack of playfulness and/sleep in a young age (baby). If visible, from the ear examination, the ear drum(s) will show redness or yellowish, bulging, or discharge.

3. **What does participation in this study involve?**
If you agree to participate in this study, your physician will ask you more questions regarding the history of your child's previous infection, allergy, and the severity of the symptoms (e.g. ear pain, fever, disruption of daily activities). As only your child and you as the parents know the best of how severe the symptoms are, we will ask you to show the severity of the symptoms using two tools. The first tool is called visual analogue scale. It is a 10-cm horizontal line, whereas the left end of the line represents 'no pain' and the right end represents 'the most painful'. We will ask you to draw a vertical line across this line at the point that represents how bad

the symptom that your child has been experiencing. The second tool is called acute otitis media – the severity of symptoms (AOM-SOS) that consisted of seven questions. You will be asked to choose one of the severity scales ('no', 'a little', or 'a lot') that corresponds to seven particular symptoms (i.e. tugging/rubbing the ears, crying more, more irritable, lack of sleep, playfulness, and appetite, and fever). Whilst you providing your best answers using these tools, your physician will also teach you to complete the symptom diary that consists similar questions that your physician has been obtained from you. This will help you in completing the symptom diary during the study which will help us to investigate the effect of the steroid in improving your child' ear pain and other symptoms due to acute middle ear infection. After that, your attending nurse and physician will examine your child's general status (i.e. body weight, height, body temperature, blood pressure) and ear-nose-throat status. From there, we will check the condition of your child's middle ear using a tool called tympanogram. This is a painless procedure to detect whether there is a fluid in your child's middle ear. From there, you will meet a nurse who will allocate your child whether she/he will receive the steroid (treatment group) or not receive the steroid (control group). Your child has 50% chance for being allocated to receive the steroid. We will do this process randomly where no one can predict in which group your child will be allocated to. This process will require 15 to 30 minutes because the nurse has to access this information from the website or calling the research team. If your child receives the steroid, she will give you a prescription for your study medication. You will give the prescription to the pharmacy at that hospital. The pharmacist will prepare your study medication by crushing the tablets, mixing it with sweeteners, and packing the study medication in a daily paper-package (you will receive five daily packages). The nurse will give an instruction to give a medication to your child every morning, once daily for 10 to 30 milligrams depends on your child's age, for five days. She will tell you what to do if your child vomits after taking a drug or experiences any effects. She also will ask you to keep the confidentiality of the treatment that your child receives from your physician and audiologist. The whole process will require 60 to 120 minutes depends on the cooperativity of your child. We will ask you to come after two and seven days after your visit. On these visits, we will investigate whether the steroid will help reducing the ear pain and other relevant symptoms and whether it give unfavorable effects. During these visits, we will ask you to bring the symptom diary and the left-over drug so we can check your child' condition. We also will ask you to come after one and three months to see whether during these time, your child experiences a new episode of acute middle ear infection. After these four additional visit after this visit, we consider that your child has completed the study.

Any information obtained in connection with this research project that can identify you child and you will remain confidential. If you agree to participate in this study, you will be asked to sign the Participant Consent Form.

4. What if I do not want to take part in this study, or if I want to withdraw later?

Participation in this study is voluntary. It is completely up to you or both of you and your child if you child aged 12 years, whether or not you participate. If you decide not to participate, it will not affect the treatment your child receive now or in the future. Whatever your decision, it will not affect your relationship with the staff caring for your child. However, it may not be possible to withdraw your data from the study results if these have already had your identifying details removed.

5. **How is this study being paid for?**
The study is being for by Dr. Respati W. Ranakusuma, ORL which is supported by self-funded.

6. **Are there risks to my child in taking part in this study?**
The foreseeable risks in taking part in this study are the bitter taste of prednisolone tablets and some potential side effects of the steroids. Pharmacist will mix the crushed tablets with sweeteners and we will also provide honey to be mixed with the medication. The common potential side effects of steroids are nausea, vomiting, abdominal pain, nervousness, mood swings, headache, increased blood sugar and blood pressure, weight gain, etc. Growth disorder could be one of the side effects however it usually occurs on the longer use of the steroids. We cannot predict whether your child will have one of these effects or not at all.

You may feel that the whole process of this study will take longer time compared to usual doctor visit due to collection of information and additional examination that will be conducted in this study. It may add some work for you to complete a symptom diary daily for the next 14 days. However, this is very important to be able to assess the day-by-day progress of your child with or without the steroids. Other potential inconveniences that your child and you may experience from this study are during the tympanometry examination and the follow-up visits (four additional visits are required in this study). Even though tympanometry is a painless procedure, we expect that your child will sit still for at least 10 minutes where she/he will hear a ringing sound and a pressure sensation during the process.

7. **What happens if my child suffers injury or complications as a result of the study?**
If you require treatment or suffer loss as a result of the negligence of any of the parties involved in the study, you may be entitled to compensation; the cost of your treatment would have to be paid out of such compensation.

8. **Will I benefit from the study?**
This study aims to further medical knowledge and may improve future treatment of acute middle ear infection (especially in mild cases where usually antibiotics are being prescribed), however, this study may not directly benefit you.

9. **Will taking part in this study cost me anything, and will I be paid?**
Participation in this study will not cost you anything, nor you will be paid. You will be reimbursed for reasonable travel expenses to the amount of $15. We also will cover the registration and consultation fees for the additional four follow-up visits to the hospital.

10. **How will my confidentiality be protected?**
Any identifiable information that is collected about your child in connection with this study will remain confidential and will be disclosed only with your permission, or except as required by law. Only the researchers named above will have access to your details and results that will be held securely at the CEEBM CMH – FMUI.

11. What happens with the results?

If you give us your permission by signing the consent document, we plan to discuss/publish the results for the monitoring and safety purposes (by the Human Research Ethics Committee, data monitoring and auditing committee, if necessary) and for publication in peer-reviewed journals or presentation at conferences or other professional forums. In any publication, information will be provided in such a way that you cannot be identified.

12. What should I do if I want to discuss this study further before I decide?

When you have read this information, your physician as one of the researchers, will discuss it with you and any queries you may have. If you would like to know more at any stage, please do not hesitate to contact Dr. Respati W. Ranakusuma, ORL by phone on +62 8111 012 185.

13. Who should I contact if I have concerns about the conduct of this study?

This study has been approved by the Medical Ethics Committee FMUI and the Bond University's Human Research Ethics Committee (BUHREC) Bond University, Queensland, Australia. Any person with concerns or complaints about the conduct of this study should contact Dr. Respati W. Ranakusuma on +62 8111 012 185, or email OPAL.study@bond.edu.au.

The conduct of this study at (please circle the answer that representing your hospital) the Dr Cipto Mangunkusumo Hospital / Persahabatan Hospital / Gatot Subroto Army Hospital / Antam Medika Hospital / Cempaka Putih Islamic Hospital / Proklamasi ENT Hospital / Hermina Bekasi Hospital, has been authorised by the the Health Agency for the Province of DKI Jakarta and the Directorate-General for Politics and General Government – The Ministry of Internal Affairs Republic Indonesia.

Thank you for taking the time to consider this study. If you wish to take part in, please sign the attached consent form. This information sheet is for you to keep

CRF01. CONSENT FORM

Oral prednisolone for acute otitis media in children: a pilot pragmatic, randomised, open-label, single-blind study (OPAL study)

[Steroids for middle ear infection in children]

1. I, ____
   of ____
   agree to participate in the study described in the participant information statement set attached to this form.
2. I acknowledge that I have read the participant information statement, which explains why my child has been selected, the aims of the study, and the nature and the possible risks of the investigation, and the statement has been explained to me to my satisfaction.
3. Before signing this consent form, I have been given the opportunity of asking any questions relating to any possible physical and mental harm my child might suffer as a result of my child participation and I have received satisfactory answers.
4. I understand that I can withdraw from the study at any time without prejudice to my relationship to my physician and the ______________________________ Hospital.
5. I agree that research data gathered from the results of the study may be published, provided that I cannot be identified.
6. I understand that I have any questions relating to my participation in this research, I may contact Dr. Respati W. Ranakusuma, ORL on telephone +62 8111 012 185, who will be happy to answer them.
7. I acknowledge receipt of a copy of this Consent Form and the Participation Information Statement.

Complaints may be directed to the OPAL Study Support Office at the Clinical Epidemiology and Evidence-Based Medicine Unit, Dr Cipto Mangunkusumo Hospital – Faculty of Medicine Universitas Indonesia, Building H Dr Cipto Mangunkusumo Hospital, Diponegoro 71, Jakarta 10430, Indonesia (phone +62 21 316 1760, email OPAL.study@bond.edu.au).

**Signature of participant or the parent**	**Name**	**Date**
______________________	______________________	______________________
**Signature of witness**	**Name**	**Date**
______________________	______________________	______________________
**Signature of investigator**	**Name**	**Date**
______________________	______________________	______________________

REVOCATION OF CONSENT

Oral prednisolone for acute otitis media in children: a pilot pragmatic, randomised, open-label, single-blind study (OPAL study)
[Steroids for middle ear infection in children]

I hereby wish to WITHDRAW my consent to participate in the study described above and understand that such withdrawal WILL NOT jeopardise any treatment or my relationship with the ______________________________ hospital or my medical attendants.

**Signature of participant or the parent**	**Name**	**Date**
____________________	____________________	____________________

The section for Revocation of Consent should be forwarded to Dr. Respati W. Ranakusuma, ORL at the Clinical Epidemiology and Evidence-Based Medicine Unit, Dr Cipto Mangunkusumo Hospital – Faculty of Medicine Universitas Indonesia.

REGISTRATION ID ____________

CRF02. STUDY REGISTRATION FORM

PATIENT'S INFORMATION

Patient's name	________________________________
Place and date of birth	______________ , ______________________
Education	O None O Pre-school O Elementary school<br>O Middle junior school
School attending hours	O 1x / week from: _____ am/pm to _____ pm/pm<br>O 2x / week from: _____ am/pm to _____ pm/pm<br>O 3x / week from: _____ am/pm to _____ pm/pm<br>O 4x / week from: _____ am/pm to _____ pm/pm<br>O ≥ 5x / week from: _____ am/pm to _____ pm/pm
Home address	________________________________<br>________________________________<br>________________________________
Home telephone number	________________________________
Home fax number	________________________________
Health service payment	O self-payment O Private insurance O Company insurance<br>O Government health coverage (BPJS) O Other: __________

Weight: ______ kg	Height: _______ cm	Temperature: _____ °C	Blood pressure: ____________ mmHg

PARENTS' INFORMATION

FATHER

Father's name	________________________________
Place and date of birth	______________
Education	O None O Elementary school O Middle junior school<br>O High school O Bachelor O Masters<br>O Doctoral
Occupation	O None O Government employee O Private employee

	O Entrepreneur O Other: ________
Home address	O Same with patient's address<br>O Different address: ________________<br>________________
Home telephone number	O Same with patient's telephone number<br>O Different number: ________________
Mobile number	________________
Email address	______ ________________
**MOTHER**	
Mother's name	________________
Place and date of birth	________________
Education	O None O Elementary school O Middle junior school<br>O High school O Bachelor O Masters<br>O Doctoral
Occupation	O None O Government employee O Private employee<br>O Entrepreneur O Other: ________
Home address	O Same with patient's address<br>O Different address: ________________<br>________________
Home telephone number	O Same with patient's telephone number<br>O Different number: ________________
Mobile number	________________
Email address	________________

Date : |___|___| – |___|___| – 201 |___|

Registration ID

Doctor ID : |___|___|___|

Hospital ID : |___|___|

CRF03 – ELIGIBILITY FORM

INCLUSION CRITERIA

OYes ONo	Definite or suspected acute otitis media (AOM)<br>OR<br>Were you able to confirm otoscopically? OYes ONo
OYes ONo	Aged 6 months to 12 years
OYes ONo	Available for follow-up visits

EXCLUSION CRITERIA

OYes ONo	Major medical conditions (e.g. heart failure, renal insufficiency, DM, peptic ulcers)
OYes ONo	Immunocompromised (e.g. cancer treatment, HIV)
OYes ONo	Congenital malformation/syndromes (e.g. cleft palate)
OYes ONo	Ventilation tube(s)
OYes ONo	Exposed to persons with varicella/active Zoster infection in the past 3 weeks with no prior history of varicella infection/ immunisation
OYes ONo	With high risk of strongyloidiasis infection
OYes ONo	Has taken oral/injection/topical steroids in the past 4 weeks
OYes ONo	Has taken antibiotics in the past 2 weeks
OYes ONo	Hypersensitive to prednisolone or other steroids

Is this child eligible for the trial?

All 'YES' at the inclusion criteria, AND
All 'NO' at the exclusion criteria

Eligible, then INCLUDE O

At least one 'NO' at the inclusion criteria, OR
At least one 'YES' at the exclusion criteria

Not eligible, then EXCLUDE O

Obtaining the CONSENT

NOT giving CONSENT → **EXCLUDE** O

Giving CONSENT → **INCLUSION** O

Do they have these following symptoms?

OYes ONo	Moderate to severe symptoms, locally or systemically (moderate to severe ear pain, fever ≥39°C, complications)
OYes ONo	Aged younger than 2 years with bilateral acute otitis media
OYes ONo	With perforation of tympanic membrane(s)
OYes ONo	If visible, otoscopic finding shows moderate to severe bulging and/or yellowish purulent tympanic membrane(s)

At least one 'YES' → **SEVERE AOM** O

All 'NO' → **MILD AOM** O

For each question, please tick (V) your answer in the circle

Registration ID [ ]

CRF04 – BASELINE INFORMATION FORM

1 Did (do) you breastfeed your child? O Yes O No

If ' YES', until the age of O ≤ 2 months O > 2 – 6 months O > 6 months

2 Does your child attend a day-care O Yes O No

How many days in a week? O ≤ 2 days O > 2 days

3 Have your child had a pneumococcus vaccine (PCV)? O Yes O No O Do not know

How many times: ___________ times

4 Have your child had an influenzae vaccine? O Yes O No O Do not know

How many times: ___________ times

5 How many episodes of recurrent acute respiratory infection (runny nose, cough, sore throat, fever) in the past year? O ≤ 3 episodes O > 3 episodes to 6 episodes O > 6 episodes

6 Did your child have a history of 3 or more episodes of ear infection (ear pain, ear discharge, diarrhoea, or vomiting) during the past 12 months? O Yes O No

7 At what age did the first episode of ear infection start?

O ≤ 6 months O > 6 to 12 months O >12 to 24 months O > 2 to 5 years O > 5 years

8 Does your child have one of the following disorders:

O Bronchial asthma

O Allergic rhinitis

O Family history of atopic disorders

O None of above

9 Number of children (including the patient) who live in the house _____________ children

10 Number of persons who smoke at home _____________ person(s)

For each question, please tick (✓) your answer on the circle or write you answer on ______

Registration ID ____________

CRF05 – OUTCOME FORM

Baseline Visit (Day-0) : |___|___| – |___|___| – 20 |___|___|

Complications (for Physician)

1	**Does your child experience discharge from the ear(s)?**	O Yes O No
2	**Does your child experience intense ear pain and pain behind the ear?**	O Yes O No
3	**Does your child experience swelling/bulging/ or redness/tenderness of the ear(s)?**	O Yes O No
4	**Does your child experience facial asymmetry (e.g. when the child smiles, cries)?**	O Yes O No

General and ENT examination (for Nurse and Physician)

5.1 **Weight** _____ kg	5.2 **Height** _______ cm	5.3 **Temp.** ________ °C	5.4 **BP** _____ / _____ mmHg

6 **Nose** O Normal O Oedema O Hyperaemic O Livid O Serous discharge O Mucoid discharge

7 **Tonsils** O Normal O Hyperaemic O Detritus O Tonsil(s) T1 O Tonsil(s) T2 O Tonsil(s) T3-4

8 **Pharynx** O Normal O Hyperaemic O Oedema O Granules O Post nasal drip (PND)

9 Otoscopic examination

O Normal O Cerumen O Erythema O Air fluid level O Complete effusion O Opacification

O Mild bulging O Moderate to severe bulging (bulging rounded) O Bulla O Perforation

10 Medicines that have been taken before the baseline visit (please circle your dose measurement)

1. ______________________ Dose : _____mg perBw kg / Teaspoon / Tablespoon ; Frequency : ____ / day
2. ______________________ Dose : _____mg perBW kg / Teaspoon / Tablespoon ; Frequency : ____ / day
3. ______________________ Dose : _____mg perBW kg / Teaspoon / Tablespoon ; Frequency : ____ / day
4. ______________________ Dose : _____mg perBW kg / Teaspoon / Tablespoon ; Frequency : ____ / day
5. ______________________ Dose : _____mg perBW kg / Teaspoon / Tablespoon ; Frequency : ____ / day

Medicines prescribed by physician (you) at the baseline visit

Antibiotic	______________________________________ Dose : _______mg / BW kg Frequency : _______ / day for _______ days

Other medicine(s)

1. ______________________ Dose : _____mg perBw kg / Teaspoon / Tablespoon ; Frequency : ____ / day
2. ______________________ Dose : _____mg perBw kg / Teaspoon / Tablespoon ; Frequency : ____ / day
3. ______________________ Dose : _____mg perBw kg / Teaspoon / Tablespoon ; Frequency : ____ / day
4. ______________________ Dose : _____mg perBw kg / Teaspoon / Tablespoon ; Frequency : ____ / day
5. ______________________ Dose : _____mg perBw kg / Teaspoon / Tablespoon ; Frequency : ____ / day

Outcome: Symptoms (for patients and the parents. Physician will help them to complete these in the symptom diary)

11 Please place a vertical line across the available horizontal line that best describes your or your child's pain during the past 24 hours?

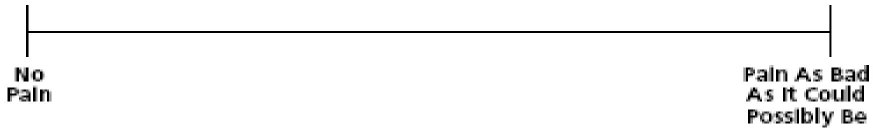

For each question, please tick (✓) your answer on the circles or write you answer on ______

12 We are interest finding out how your child has been doing. For each question, please place a checkmark (V) in the circle corresponding to your child's symptoms. Please answer all questions.			
12.1 Over the past 12 h, has your child been tugging, rubbing, or holding the ear(s) more than usual?	O No	O A little	O A lot
12.2 Over the past 12 h, has your child been crying more than usual?	O No	O A little	O A lot
12.3 Over the past 12 h, has your child been more irritable or fussy than usual?	O No	O A little	O A lot
12.4 Over the past 12 h, has your child been having more difficulty sleeping than usual?	O No	O A little	O A lot
12.5 Over the past 12 h, has your child been less playful or active than usual?	O No	O A little	O A lot
12.6 Over the past 12 h, has your child been eating less than usual?	O No	O A little	O A lot
12.7 Over the past 12 h, has your child been having fever or feeling warm to touch?	O No	O A little	O A lot

13 Tympanometry examination (for Audiologist and interpreted by Physician)

O Cannot be performed. Reason: ____

Tympanogram types (will be completed by physician) [R] Type ______ / [L] Type ______

Ear canal vol (ECV) [R] ________ mL / [L] ________ mL

Static acoustic admittance [R] _______ mL / [L] _______ mL

Compliance (SC) [R] ________ mL / [L] ________ mL

Middle Ear Pressure or TPP [R] _______ daPa / [L] ______ daPa

Gradient or TW [R] _______ daPa / [L] ______ daPa

Put the copy of tympanometry copies here

Registration ID | |

Follow-up Visit – 1 (Day – 3) : |___|___| – |___|___| – 20 |___|___|

Complications (for Physician)

1	**Does your child experience discharge from the ear(s)?**	O Yes O No
2	**Does your child experience intense ear pain and pain behind the ear?**	O Yes O No
3	**Does your child experience swelling/bulging/ or redness/tenderness of the ear(s)?**	O Yes O No
4	**Does your child experience facial asymmetry (e.g. when the child smiles, cries)?**	O Yes O No

General and ENT examination (for Nurse and Physician)

5.1 **Weight** _____ kg	5.2 **Height** _______ cm	5.3 **Temp.** ________ °C	5.4 **BP** _____ / _____ mmHg

6 **Nose** O Normal O Oedema O Hyperaemic O Livid O Serous discharge O Mucoid discharge

7 **Tonsils** O Normal O Hyperaemic O Detritus O Tonsil(s) T1 O Tonsil(s) T2 O Tonsil(s) T3-4

8 **Pharynx** O Normal O Hyperaemic O Oedema O Granules O Post nasal drip (PND)

9 Otoscopic examination

O Normal O Cerumen O Erythema O Air fluid level O Complete effusion O Opacification

O Mild bulging O Moderate to severe bulging (bulging rounded) O Bulla O Perforation

10 Medicines prescribed by you (Physician) on today visit (please circle your dose measurement)

Antibiotic	Dose : _______mg / BW kg Frequency : _______ / day for _______ days

Other medicine(s)

1. _________________________	Dose : _____mg perBw kg / Teaspoon / Tablespoon ;	Frequency :	____ / day
2. _________________________	Dose : _____mg perBw kg / Teaspoon / Tablespoon ;	Frequency :	____ / day
3. _________________________	Dose : _____mg perBw kg / Teaspoon / Tablespoon ;	Frequency :	____ / day
4. _________________________	Dose : _____mg perBw kg / Teaspoon / Tablespoon ;	Frequency :	____ / day
5. _________________________	Dose : _____mg perBw kg / Teaspoon / Tablespoon ;	Frequency :	____ / day

11 Medicines NOT prescribed by you or from over-the-counter or others (e.g. other physician, drug store)

Antibiotic	Dose : _______mg / BW kg Frequency : _______ / day for _______ days

Other medicine(s)

1. _________________________	Dose : _____mg perBw kg / Teaspoon / Tablespoon ;	Frequency :	____ / day
2. _________________________	Dose : _____mg perBw kg / Teaspoon / Tablespoon ;	Frequency :	____ / day
3. _________________________	Dose : _____mg perBw kg / Teaspoon / Tablespoon ;	Frequency :	____ / day
4. _________________________	Dose : _____mg perBw kg / Teaspoon / Tablespoon ;	Frequency :	____ / day
5. _________________________	Dose : _____mg perBw kg / Teaspoon / Tablespoon ;	Frequency :	____ / day

Outcome: Symptoms (for Patients)

12 Please place a vertical line across the available horizontal line that best describes your or your child's pain during the past 24 hours?

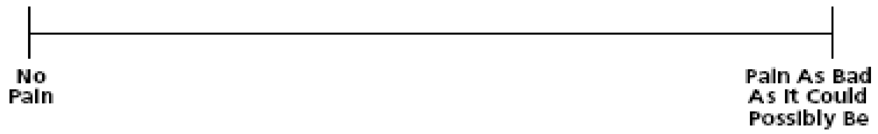

**13. We are interest finding out how your child has been doing. For each question, please place a checkmark (V) in the circle corresponding to your child's symptoms. Please answer all questions.**			
13.1 Over the past 12 h, has your child been tugging, rubbing, or holding the ear(s) more than usual?	O No	O A little	O A lot
13.2 Over the past 12 h, has your child been crying more than usual?	O No	O A little	O A lot
13.3 Over the past 12 h, has your child been more irritable or fussy than usual?	O No	O A little	O A lot
13.4 Over the past 12 h, has your child been having more difficulty sleeping than usual?	O No	O A little	O A lot
13.5 Over the past 12 h, has your child been less playful or active than usual?	O No	O A little	O A lot
13.6 Over the past 12 h, has your child been eating less than usual?	O No	O A little	O A lot
13.7 Over the past 12 h, has your child been having fever or feeling warm to touch?	O No	O A little	O A lot

14 Side effects

Does your child have these complaints after taking the medicine

14.1 Increased appetite	O Yes	O No	14.8 Drowsiness	O Yes	O No
14.2 Increased urine amount	O Yes	O No	14.9 Anxiety/distractibility/mood swing	O Yes	O No
14.3 Weight gain	O Yes	O No	14.10 Headache	O Yes	O No
14.4 Gastritis/abdominal pain	O Yes	O No	14.11 Skin rash or diaper rash	O Yes	O No
14.5 Nausea	O Yes	O No	14.12 Candidiasis	O Yes	O No
14.6 Vomiting	O Yes	O No	14.13 Dry mouth / throat irritation	O Yes	O No
14.7 Diarrhea	O Yes	O No	14.14 Sleep disturbance	O Yes	O No

Others: ____

Did you bring your child to doctor (clinic or outpatient)?	O Yes	O No	Reason: ____________________<br>Medicine prescribed: ____________________
Has your child has been admitted to hospital?	O Yes	O No	Reason: ____________________<br>Medicine prescribed: ____________________

Regarding the side effects, your action is/are (you may answer more than one):

O Discontinuation of the study drug (prednisolone)

O Continuation of the study drug

O Discontinuation of other concomitant drugs as follows:

1. ____________________ 3. ____________________
2. ____________________ 4. ____________________

The treatment you prescribed for the management of side effects

1. ____________________; Dose __________; Frequency ______ / day
2. ____________________; Dose __________; Frequency ______ / day
3. ____________________; Dose __________; Frequency ______ / day
4. ____________________; Dose __________; Frequency ______ / day

Does this child require specific or additional tests or examination?

O No

O Yes. Please specify with the results:

1. ____
2. ____
3. ____

Does this child require specific or additional treatment or medication	O No<br>O Yes. Please specify the treatment:<br>1. ____________________; Dose __________; Frequency ______ / day<br>2. ____________________; Dose __________; Frequency ______ / day<br>3. ____________________; Dose __________; Frequency ______ / day<br>4. ____________________; Dose __________; Frequency ______ / day
Does this child require a hospitalisation?	O No<br>O Yes. Please explain your reasons to hospitalise this child and the treatment will be given<br>Reason: ____<br>____<br>The treatment:<br>1. ____________________; Dose __________; Frequency ______ / day<br>2. ____________________; Dose __________; Frequency ______ / day<br>3. ____________________; Dose __________; Frequency ______ / day<br>4. ____________________; Dose __________; Frequency ______ / day

15 Tympanometry examination (for Audiologist and interpreted by Physician)

O Cannot be performed. Reason: ____

Tympanogram types (will be completed by physician) [R] Type ______ / [L] Type ______

Ear canal vol (ECV) [R] ________ mL / [L] ________ mL

Static acoustic admittance [R] _______ mL / [L] _______ mL

Compliance (SC) [R] ________ mL / [L] ________ mL

Middle Ear Pressure or TPP [R] _______ daPa / [L] ______ daPa

Gradient or TW [R] _______ daPa / [L] ______ daPa

Put the copy of tympanometry copies here

Registration ID [ ]

Follow-up Visit – 2 (Day – 7) : |___|___| – |___|___| – 20 |___|___|

Complications (for Physician)

1	Does your child experience discharge from the ear(s)?	O Yes O No
2	Does your child experience intense ear pain and pain behind the ear?	O Yes O No
3	Does your child experience swelling/bulging/ or redness/tenderness of the ear(s)?	O Yes O No
4	Does your child experience facial asymmetry (e.g. when the child smiles, cries)?	O Yes O No

General and ENT examination (for Nurse and Physician)

5.1 **Weight** _____ kg | 5.2 **Height** _______ cm | 5.3 **Temp.** _________ °C | 5.4 **BP** _____ / _____ mmHg

6 **Nose** O Normal O Oedema O Hyperaemic O Livid O Serous discharge O Mucoid discharge

7 **Tonsils** O Normal O Hyperaemic O Detritus O Tonsil(s) T1 O Tonsil(s) T2 O Tonsil(s) T3-4

8 **Pharynx** O Normal O Hyperaemic O Oedema O Granules O Post nasal drip (PND)

9 Otoscopic examination

O Normal O Cerumen O Erythema O Air fluid level O Complete effusion O Opacification

O Mild bulging O Moderate to severe bulging (bulging rounded) O Bulla O Perforation

10 Medicines prescribed by you (Physician) today visit (please circle your dose measurement)

Antibiotic	______________________ Dose : _______mg / BW kg Frequency : _______ / day for _______ days

Other medicine(s)

1. ______________________ Dose : _____mg perBw kg / Teaspoon / Tablespoon ; Frequency : ____ / day
2. ______________________ Dose : _____mg perBw kg / Teaspoon / Tablespoon ; Frequency : ____ / day
3. ______________________ Dose : _____mg perBw kg / Teaspoon / Tablespoon ; Frequency : ____ / day
4. ______________________ Dose : _____mg perBw kg / Teaspoon / Tablespoon ; Frequency : ____ / day
5. ______________________ Dose : _____mg perBw kg / Teaspoon / Tablespoon ; Frequency : ____ / day

11 Medicines NOT prescribed by you or from over-the-counter or others (e.g. other physician, drug store)

Antibiotic	______________________ Dose : _______mg / BW kg Frequency : _______ / day for _______ days

Other medicine(s)

6. ______________________ Dose : _____mg perBw kg / Teaspoon / Tablespoon ; Frequency : ____ / day
7. ______________________ Dose : _____mg perBw kg / Teaspoon / Tablespoon ; Frequency : ____ / day
8. ______________________ Dose : _____mg perBw kg / Teaspoon / Tablespoon ; Frequency : ____ / day
9. ______________________ Dose : _____mg perBw kg / Teaspoon / Tablespoon ; Frequency : ____ / day
10. ______________________ Dose : _____mg perBw kg / Teaspoon / Tablespoon ; Frequency : ____ / day

Outcome: Symptoms (for Patients)

12 Please place a vertical line across the available horizontal line that best describes your or your child's pain during the past 24 hours?

|——————————————————————————|

No Pain ... Pain As Bad As It Could Possibly Be

For each question, please tick (✓) your answer on the circles or write you answer on ______

**13 We are interest finding out how your child has been doing. For each question, please place a checkmark (V) in the circle corresponding to your child's symptoms. Please answer all questions.**			
13.1 Over the past 12 h, has your child been tugging, rubbing, or holding the ear(s) more than usual?	O No	O A little	O A lot
13.2 Over the past 12 h, has your child been crying more than usual?	O No	O A little	O A lot
13.3 Over the past 12 h, has your child been more irritable or fussy than usual?	O No	O A little	O A lot
13.4 Over the past 12 h, has your child been having more difficulty sleeping than usual?	O No	O A little	O A lot
13.5 Over the past 12 h, has your child been less playful or active than usual?	O No	O A little	O A lot
13.6 Over the past 12 h, has your child been eating less than usual?	O No	O A little	O A lot
13.7 Over the past 12 h, has your child been having fever or feeling warm to touch?	O No	O A little	O A lot

14 Side effects

Does your child have these complaints after taking the medicine

14.1 Increased appetite	O Yes	O No	14.8 Drowsiness	O Yes	O No
14.2 Increased urine amount	O Yes	O No	14.9 Anxiety/distractibility/mood swing	O Yes	O No
14.3 Weight gain	O Yes	O No	14.10 Headache	O Yes	O No
14.4 Gastritis/abdominal pain	O Yes	O No	14.11 Skin rash or diaper rash	O Yes	O No
14.5 Nausea	O Yes	O No	14.12 Candidiasis	O Yes	O No
14.6 Vomiting	O Yes	O No	14.13 Dry mouth / throat irritation	O Yes	O No
14.7 Diarrhea	O Yes	O No	14.14 Sleep disturbance	O Yes	O No

Others: ____

Did you bring your child to doctor (clinic or outpatient)?	O Yes	O No	Reason: ____________________<br>Medicine prescribed: ____________________
Has your child has been admitted to hospital?	O Yes	O No	Reason: ____________________<br>Medicine prescribed: ____________________

Regarding the side effects, your action is/are (you may answer more than one):

O Discontinuation of the study drug (prednisolone)

O Continuation of the study drug

O Discontinuation of other concomitant drugs as follows:

1. ____________________ 3. ____________________
2. ____________________ 4. ____________________

Does this child require specific or additional tests or examination?

O No

O Yes. Please specify with the results:

1. ____
____
2. ____
____
3. ____
____

Does this child require specific or additional treatment or medication

O No

O Yes. Please specify the treatment:

5. ____________________; Dose __________; Frequency ______ / day

6. ______________________; Dose ___________; Frequency ______ / day
7. ______________________; Dose ___________; Frequency ______ / day
8. ______________________; Dose ___________; Frequency ______ / day

Does this child require a hospitalisation?

O No

O Yes. Please explain your reasons to hospitalise this child and the treatment will be given

Reason: ____

____

The treatment:

5. ______________________; Dose ___________; Frequency ______ / day
6. ______________________; Dose ___________; Frequency ______ / day
7. ______________________; Dose ___________; Frequency ______ / day
8. ______________________; Dose ___________; Frequency ______ / day

15 Tympanometry examination (for Audiologist and interpreted by Physician)

O Cannot be performed. Reason: ____

Tympanogram types (will be completed by physician) [R] Type ______ / [L] Type ______

Ear canal vol (ECV) [R] ________ mL / [L] ________ mL

Static acoustic admittance [R] _______ mL / [L] _______ mL

Compliance (SC) [R] ________ mL / [L] ________ mL

Middle Ear Pressure or TPP [R] _______ daPa / [L] ______ daPa

Gradient or TW [R] _______ daPa / [L] ______ daPa

Put the copy of tympanometry copies here

Follow-up Visit – 3 (Day – 30) : |___|___| – |___|___| – 20 |___|___|

Outcome: Symptoms (for Patients)

1 Within the past one month, does your child experience a new episode of ear pain with fever or runny nose, cough, or sore throat? Please write your answer and circle the most appropriate time	O Yes O No<br>When? ________ days / weeks ago<br>How long? ________ days / weeks

2 Please place a vertical line across the available horizontal line that best describes your or your child's pain during the past 24 hours? (if applicable)

No Pain |————————————————————| Pain As Bad As It Could Possibly Be

3. We are interest finding out how your child has been doing. For each question, please place a checkmark (V) in the circle corresponding to your child's symptoms. Please answer all questions (if applicable).

3.1 Over the past 12 h, has your child been tugging, rubbing, or holding the ear(s) more than usual?	O No	O A little	O A lot
3.2 Over the past 12 h, has your child been crying more than usual?	O No	O A little	O A lot
3.3 Over the past 12 h, has your child been more irritable or fussy than usual?	O No	O A little	O A lot
3.4 Over the past 12 h, has your child been having more difficulty sleeping than usual?	O No	O A little	O A lot
3.5 Over the past 12 h, has your child been less playful or active than usual?	O No	O A little	O A lot
3.6 Over the past 12 h, has your child been eating less than usual?	O No	O A little	O A lot
3.7 Over the past 12 h, has your child been having fever or feeling warm to touch?	O No	O A little	O A lot

General and ENT examination (for Nurse and Physician)

4.1 **Weight** _____ kg	5.2 **Height** _______ cm	5.3 **Temp.** ________ °C	5.4 **BP** _____ / _____ mmHg

5 **Nose** O Normal O Oedema O Hyperaemic O Livid O Serous discharge O Mucoid discharge

6 **Tonsils** O Normal O Hyperaemic O Detritus O Tonsil(s) T1 O Tonsil(s) T2 O Tonsil(s) T3-4

7 **Pharynx** O Normal O Hyperaemic O Oedema O Granules O Post nasal drip (PND)

8 Otoscopic examination

O Normal O Cerumen O Erythema O Air fluid level O Complete effusion O Opacification

O Mild bulging O Moderate to severe bulging (bulging rounded) O Bulla O Perforation

9 Tympanometry examination (for Audiologist and interpreted by Physician)

O Cannot be performed. Reason: ____

Tympanogram types (will be completed by physician) [R] Type ______ / [L] Type ______

Ear canal vol (ECV) [R] ________ mL / [L] ________ mL

Static acoustic admittance [R] _______ mL / [L] _______ mL

Compliance (SC) [R] ________ mL / [L] ________ mL

Middle Ear Pressure or TPP [R] _______ daPa / [L] ______ daPa

Gradient or TW [R] _______ daPa / [L] ______ daPa

For each question, please tick (✓) your answer on the circles or write you answer on ______

Put the copy of tympanometry copies here

Registration ID | |

Follow-up Visit – 4 (Day – 90) : |___|___| – |___|___| – 20 |__|__|

Outcome: Symptoms (for Patients)

1 Within the past one month, does your child experience a new episode of ear pain with fever or runny nose, cough, or sore throat? Please write your answer and circle the most appropriate time	O Yes O No<br>When? ________ days / weeks ago<br>How long? ________ days / weeks

2 Please place a vertical line across the available horizontal line that best describes your or your child's pain during the past 24 hours? (if applicable)

No Pain |——————————————| Pain As Bad As It Could Possibly Be

3 We are interest finding out how your child has been doing. For each question, please place a checkmark (V) in the circle corresponding to your child's symptoms. Please answer all questions (if applicable).

3.1 Over the past 12 h, has your child been tugging, rubbing, or holding the ear(s) more than usual?	O No	O A little	O A lot
3.2 Over the past 12 h, has your child been crying more than usual?	O No	O A little	O A lot
3.3 Over the past 12 h, has your child been more irritable or fussy than usual?	O No	O A little	O A lot
3.4 Over the past 12 h, has your child been having more difficulty sleeping than usual?	O No	O A little	O A lot
3.5 Over the past 12 h, has your child been less playful or active than usual?	O No	O A little	O A lot
3.6 Over the past 12 h, has your child been eating less than usual?	O No	O A little	O A lot
3.7 Over the past 12 h, has your child been having fever or feeling warm to touch?	O No	O A little	O A lot

General and ENT examination (for Nurse and Physician)

4.1 **Weight** _____ kg	5.2 **Height** _______ cm	5.3 **Temp.** ________ °C	5.4 **BP** _____ / _____ mmHg

5 **Nose** O Normal O Oedema O Hyperaemic O Livid O Serous discharge O Mucoid discharge

6 **Tonsils** O Normal O Hyperaemic O Detritus O Tonsil(s) T1 O Tonsil(s) T2 O Tonsil(s) T3-4

7 **Pharynx** O Normal O Hyperaemic O Oedema O Granules O Post nasal drip (PND)

8 Otoscopic examination

O Normal O Cerumen O Erythema O Air fluid level O Complete effusion O Opacification

O Mild bulging O Moderate to severe bulging (bulging rounded) O Bulla O Perforation

9 Tympanometry examination (for Audiologist and interpreted by Physician)

O Cannot be performed. Reason: ____

Tympanogram types (will be completed by physician) [R] Type ______ / [L] Type ______

Ear canal vol (ECV) [R] ________ mL / [L] ________ mL

Static acoustic admittance [R] _______ mL / [L] _______ mL

Compliance (SC) [R] ________ mL / [L] ________ mL

Middle Ear Pressure or TPP [R] _______ daPa / [L] ______ daPa

Gradient or TW [R] _______ daPa / [L] ______ daPa

Put the copy of tympanometry copies here

***** End *****

Additional Visit : |___|___| – |___|___| – 20 |___|___|

Complications (for Physician)

1	Does your child experience discharge from the ear(s)?	O Yes O No
2	Does your child experience intense ear pain and pain behind the ear?	O Yes O No
3	Does your child experience swelling/bulging/ or redness/tenderness of the ear(s)?	O Yes O No
4	Does your child experience facial asymmetry (e.g. when the child smiles, cries)?	O Yes O No

General and ENT examination (for Nurse and Physician)

5.1 **Weight** _____ kg | 5.2 **Height** _______ cm | 5.3 **Temp.** ________ °C | 5.4 **BP** _____ / _____ mmHg

6 **Nose** O Normal O Oedema O Hyperaemic O Livid O Serous discharge O Mucoid discharge

7 **Tonsils** O Normal O Hyperaemic O Detritus O Tonsil(s) T1 O Tonsil(s) T2 O Tonsil(s) T3-4

8 **Pharynx** O Normal O Hyperaemic O Oedema O Granules O Post nasal drip (PND)

9 Otoscopic examination

O Normal O Cerumen O Erythema O Air fluid level O Complete effusion O Opacification

O Mild bulging O Moderate to severe bulging (bulging rounded) O Bulla O Perforation

10 Medicines prescribed by you (Physician) on today visit (please circle your dose measurement)

Antibiotic	________________________________ Dose : _______mg / BW kg Frequency : _______ / day for _______ days

Other medicine(s)

6. ________________________ Dose : _____mg perBw kg / Teaspoon / Tablespoon ; Frequency : ____ / day
7. ________________________ Dose : _____mg perBw kg / Teaspoon / Tablespoon ; Frequency : ____ / day
8. ________________________ Dose : _____mg perBw kg / Teaspoon / Tablespoon ; Frequency : ____ / day
9. ________________________ Dose : _____mg perBw kg / Teaspoon / Tablespoon ; Frequency : ____ / day
10. ________________________ Dose : _____mg perBw kg / Teaspoon / Tablespoon ; Frequency : ____ / day

11 Medicines NOT prescribed by you or from over-the-counter or others (e.g. other physician, drug store)

Antibiotic	________________________________ Dose : _______mg / BW kg Frequency : _______ / day for _______ days

Other medicine(s)

11. ________________________ Dose : _____mg perBw kg / Teaspoon / Tablespoon ; Frequency : ____ / day
12. ________________________ Dose : _____mg perBw kg / Teaspoon / Tablespoon ; Frequency : ____ / day
13. ________________________ Dose : _____mg perBw kg / Teaspoon / Tablespoon ; Frequency : ____ / day
14. ________________________ Dose : _____mg perBw kg / Teaspoon / Tablespoon ; Frequency : ____ / day
15. ________________________ Dose : _____mg perBw kg / Teaspoon / Tablespoon ; Frequency : ____ / day

Outcome: Symptoms (for Patients)

13 Please place a vertical line across the available horizontal line that best describes your or your child's pain during the past 24 hours?

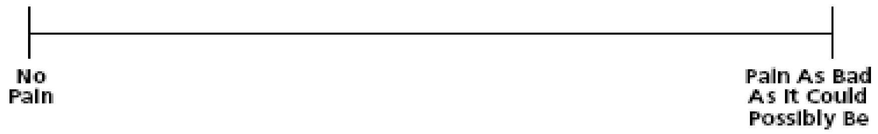

For each question, please tick (✓) your answer on the circles or write you answer on ______

13. We are interest finding out how your child has been doing. For each question, please place a checkmark (V) in the circle corresponding to your child's symptoms. Please answer all questions.

13.1 Over the past 12 h, has your child been tugging, rubbing, or holding the ear(s) more than usual?	O No	O A little	O A lot
13.2 Over the past 12 h, has your child been crying more than usual?	O No	O A little	O A lot
13.3 Over the past 12 h, has your child been more irritable or fussy than usual?	O No	O A little	O A lot
13.4 Over the past 12 h, has your child been having more difficulty sleeping than usual?	O No	O A little	O A lot
13.5 Over the past 12 h, has your child been less playful or active than usual?	O No	O A little	O A lot
13.6 Over the past 12 h, has your child been eating less than usual?	O No	O A little	O A lot
13.7 Over the past 12 h, has your child been having fever or feeling warm to touch?	O No	O A little	O A lot

14 Side effects

Does your child have these complaints after taking the medicine

14.1 Increased appetite	O Yes	O No	14.8 Drowsiness	O Yes	O No
14.2 Increased urine amount	O Yes	O No	14.9 Anxiety/distractibility/mood swing	O Yes	O No
14.3 Weight gain	O Yes	O No	14.10 Headache	O Yes	O No
14.4 Gastritis/abdominal pain	O Yes	O No	14.11 Skin rash or diaper rash	O Yes	O No
14.5 Nausea	O Yes	O No	14.12 Candidiasis	O Yes	O No
14.6 Vomiting	O Yes	O No	14.13 Dry mouth / throat irritation	O Yes	O No
14.7 Diarrhea	O Yes	O No	14.14 Sleep disturbance	O Yes	O No

Others: ____

Did you bring your child to doctor (clinic or outpatient)?	O Yes	O No	Reason: ____________________<br>Medicine prescribed: ____________________
Has your child has been admitted to hospital?	O Yes	O No	Reason: ____________________<br>Medicine prescribed: ____________________

Regarding the side effects, your action is/are (you may answer more than one):

O Discontinuation of the study drug (prednisolone)

O Continuation of the study drug

O Discontinuation of other concomitant drugs as follows:

1. ____________________ 3. ____________________
2. ____________________ 4. ____________________

The treatment you prescribed for the management of side effects

5. ____________________; Dose __________; Frequency ______ / day
6. ____________________; Dose __________; Frequency ______ / day
7. ____________________; Dose __________; Frequency ______ / day
8. ____________________; Dose __________; Frequency ______ / day

Does this child require specific or additional tests or examination?

O No

O Yes. Please specify with the results:

4. ____
5. ____
6. ____

Does this child require specific or additional treatment or medication	O No<br>O Yes. Please specify the treatment:<br>9. ________________________; Dose ____________; Frequency ______ / day<br>10. ________________________; Dose ____________; Frequency ______ / day<br>11. ________________________; Dose ____________; Frequency ______ / day<br>12. ________________________; Dose ____________; Frequency ______ / day
Does this child require a hospitalisation?	O No<br>O Yes. Please explain your reasons to hospitalise this child and the treatment will be given<br>Reason: ____<br>____<br>The treatment:<br>9. ________________________; Dose ____________; Frequency ______ / day<br>10. ________________________; Dose ____________; Frequency ______ / day<br>11. ________________________; Dose ____________; Frequency ______ / day<br>12. ________________________; Dose ____________; Frequency ______ / day

15 Tympanometry examination (for Audiologist and interpreted by Physician)

O Cannot be performed. Reason: ____

Tympanogram types (will be completed by physician) [R] Type ______ / [L] Type ______

Ear canal vol (ECV) [R] ________ mL / [L] ________ mL

Static acoustic admittance [R] _______ mL / [L] _______ mL

Compliance (SC) [R] ________ mL / [L] ________ mL

Middle Ear Pressure or TPP [R] _______ daPa / [L] ______ daPa

Gradient or TW [R] _______ daPa / [L] ______ daPa

Put the copy of tympanometry copies here

Registration ID ____________________

Additional Visit : |___|___| – |___|___| – 20 |___|___|

Complications (for Physician)

1	**Does your child experience discharge from the ear(s)?**	O Yes O No
2	**Does your child experience intense ear pain and pain behind the ear?**	O Yes O No
3	**Does your child experience swelling/bulging/ or redness/tenderness of the ear(s)?**	O Yes O No
4	**Does your child experience facial asymmetry (e.g. when the child smiles, cries)?**	O Yes O No

General and ENT examination (for Nurse and Physician)

5.1 **Weight** _____ kg | 5.2 **Height** _______ cm | 5.3 **Temp.** ________ °C | 5.4 **BP** _____ / _____ mmHg

6 **Nose** O Normal O Oedema O Hyperaemic O Livid O Serous discharge O Mucoid discharge

7 **Tonsils** O Normal O Hyperaemic O Detritus O Tonsil(s) T1 O Tonsil(s) T2 O Tonsil(s) T3-4

8 **Pharynx** O Normal O Hyperaemic O Oedema O Granules O Post nasal drip (PND)

9 Otoscopic examination

O Normal O Cerumen O Erythema O Air fluid level O Complete effusion O Opacification

O Mild bulging O Moderate to severe bulging (bulging rounded) O Bulla O Perforation

10 Medicines prescribed by you (Physician) on today visit (please circle your dose measurement)

Antibiotic ______________________________

Dose : _______mg / BW kg Frequency : _______ / day for _______ days

Other medicine(s)

1. ________________________ Dose : _____mg perBw kg / Teaspoon / Tablespoon ; Frequency : ____ / day
2. ________________________ Dose : _____mg perBw kg / Teaspoon / Tablespoon ; Frequency : ____ / day
3. ________________________ Dose : _____mg perBw kg / Teaspoon / Tablespoon ; Frequency : ____ / day
4. ________________________ Dose : _____mg perBw kg / Teaspoon / Tablespoon ; Frequency : ____ / day
5. ________________________ Dose : _____mg perBw kg / Teaspoon / Tablespoon ; Frequency : ____ / day

11 Medicines NOT prescribed by you or from over-the-counter or others (e.g. other physician, drug store)

Antibiotic ______________________________

Dose : _______mg / BW kg Frequency : _______ / day for _______ days

Other medicine(s)

1. ________________________ Dose : _____mg perBw kg / Teaspoon / Tablespoon ; Frequency : ____ / day
2. ________________________ Dose : _____mg perBw kg / Teaspoon / Tablespoon ; Frequency : ____ / day
3. ________________________ Dose : _____mg perBw kg / Teaspoon / Tablespoon ; Frequency : ____ / day
4. ________________________ Dose : _____mg perBw kg / Teaspoon / Tablespoon ; Frequency : ____ / day
5. ________________________ Dose : _____mg perBw kg / Teaspoon / Tablespoon ; Frequency : ____ / day

Outcome: Symptoms (for Patients)

12 **Please place a vertical line across the available horizontal line that best describes your or your child's pain during the past 24 hours?**

No Pain |————————————————————| Pain As Bad As It Could Possibly Be

**13 We are interest finding out how your child has been doing. For each question, please place a checkmark (V) in the circle corresponding to your child's symptoms. Please answer all questions.**			
13.1 Over the past 12 h, has your child been tugging, rubbing, or holding the ear(s) more than usual?	O No	O A little	O A lot
13.2 Over the past 12 h, has your child been crying more than usual?	O No	O A little	O A lot
13.3 Over the past 12 h, has your child been more irritable or fussy than usual?	O No	O A little	O A lot
13.4 Over the past 12 h, has your child been having more difficulty sleeping than usual?	O No	O A little	O A lot
13.5 Over the past 12 h, has your child been less playful or active than usual?	O No	O A little	O A lot
13.6 Over the past 12 h, has your child been eating less than usual?	O No	O A little	O A lot
13.7 Over the past 12 h, has your child been having fever or feeling warm to touch?	O No	O A little	O A lot

14 Side effects

14.1 Increased appetite	O Yes	O No	14.8 Drowsiness	O Yes	O No
14.2 Increased urine amount	O Yes	O No	14.9 Anxiety/distractibility/mood swing	O Yes	O No
14.3 Weight gain	O Yes	O No	14.10 Headache	O Yes	O No
14.4 Gastritis/abdominal pain	O Yes	O No	14.11 Skin rash or diaper rash	O Yes	O No
14.5 Nausea	O Yes	O No	14.12 Candidiasis	O Yes	O No
14.6 Vomiting	O Yes	O No	14.13 Dry mouth / throat irritation	O Yes	O No
14.7 Diarrhea	O Yes	O No	14.14 Sleep disturbance	O Yes	O No

Others: ____________________

Did you bring your child to doctor (clinic or outpatient)?	O Yes	O No	Reason: ____________________<br>Medicine prescribed: ____________________
Has your child has been admitted to hospital?	O Yes	O No	Reason: ____________________<br>Medicine prescribed: ____________________

Regarding the side effects, your action is/are (you may answer more than one):

O Discontinuation of the study drug (prednisolone)

O Continuation of the study drug

O Discontinuation of other concomitant drugs as follows:

1. ____________________ 3. ____________________
2. ____________________ 4. ____________________

The treatment you prescribed for the management of side effects

9. ____________________; Dose __________; Frequency ______ / day
10. ____________________; Dose __________; Frequency ______ / day
11. ____________________; Dose __________; Frequency ______ / day
12. ____________________; Dose __________; Frequency ______ / day

Does this child require specific or additional tests or examination?

O No

O Yes. Please specify with the results:

7. ____________________
____
8. ____________________
____
9. ____________________
____

Does this child require specific O No

or additional treatment or medication	O Yes. Please specify the treatment:<br>13. ______________________; Dose ___________; Frequency ______ / day<br>14. ______________________; Dose ___________; Frequency ______ / day<br>15. ______________________; Dose ___________; Frequency ______ / day<br>16. ______________________; Dose ___________; Frequency ______ / day
Does this child require a hospitalisation?	O No<br>O Yes. Please explain your reasons to hospitalise this child and the treatment will be given<br>Reason: ____<br>____<br>The treatment:<br>13. ______________________; Dose ___________; Frequency ______ / day<br>14. ______________________; Dose ___________; Frequency ______ / day<br>15. ______________________; Dose ___________; Frequency ______ / day<br>16. ______________________; Dose ___________; Frequency ______ / day

15 Tympanometry examination (for Audiologist and interpreted by Physician)

O Cannot be performed. Reason: ____

Tympanogram types (will be completed by physician) [R] Type ______ / [L] Type ______

Ear canal vol (ECV) [R] ________ mL / [L] ________ mL

Static acoustic admittance [R] _______ mL / [L] _______ mL

Compliance (SC) [R] ________ mL / [L] ________ mL

Middle Ear Pressure or TPP [R] _______ daPa / [L] ______ daPa

Gradient or TW [R] _______ daPa / [L] ______ daPa

Put the copy of tympanometry copies here

DIARY-1 (Day-0 to Day-3)

OPAL Study

Oral Prednisolone for Acute otitis media in children

Registration ID

Hello Uncle / Aunty!!

My name is________________________

I was born in__________________

On date _____ month ________ year ___________

If you find this Diary, I would be very grateful if you can return it to my Dad (mobile no. _______________) or my Mom (mobile no. _______________).

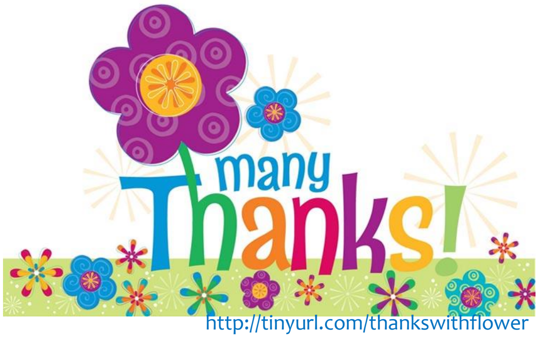

Registration ID

Day-0 (*your first visit*) : |___|___| – |___|___| – 20 |___|___|

1. Please place a vertical line across the available horizontal line that best describes your or your child's pain during the past 12 hours? Please write the time accordingly ……. (am/ pm)

No Pain ——————————————— Pain As Bad As It Could Possibly Be

2. We are interest finding out how your child has been doing. For each question, please place a check mark in O corresponding to your child's symptoms. Please answer all questions. Please write the time accordingly ……. (am/ pm)

2.1 Over the past 12 h, has your child been tugging, rubbing, or holding the ear(s) more than usual?	O No	O A little	O A lot
2.2 Over the past 12 h, has your child been crying more than usual?	O No	O A little	O A lot
2.3 Over the past 12 h, has your child been more irritable or fussy than usual?	O No	O A little	O A lot
2.4 Over the past 12 h, has your child been having more difficulty sleeping than usual?	O No	O A little	O A lot
2.5 Over the past 12 h, has your child been less playful or active than usual?	O No	O A little	O A lot
2.6 Over the past 12 h, has your child been eating less than usual?	O No	O A little	O A lot
2.7 Over the past 12 h, has your child been having fever or feeling warm to touch?	O No	O A little	O A lot

Other symptoms

3 Does your child experience discharge from the ear(s)?	O Yes	O No
4 Does your child experience intense ear pain and pain behind the ear?	O Yes	O No
5 Does your child experience swelling/bulging, redness, tenderness, or dropping behind or of the ear(s)	O Yes	O No
6 Does your child experience facial asymmetry (e.g. when the child smiles, cries)?	O Yes	O No

Medicines given (please write the name, dose, and frequency)

Medicines have been given to your child before going to the hospital (from other doctor or chemist store)	______________	Dose : _____ mg / body weight kg	Frequency : ______ / day
	______________	Dose : _____ mg / body weight kg	Frequency : ______ / day
	______________	Dose : _____ mg / body weight kg	Frequency : ______ / day
	______________	Dose : _____ mg / body weight kg	Frequency : ______ / day
	______________	Dose : _____ mg / body weight kg	Frequency : ______ / day

Please list all medicines you give to your child today by marking the circle based on the frequency and the time

______________	O ____ am/pm	O ____ am/pm	O ____ am/pm	O ____ am/pm	O ____ am/pm
______________	O ____ am/pm	O ____ am/pm	O ____ am/pm	O ____ am/pm	O ____ am/pm
______________	O ____ am/pm	O ____ am/pm	O ____ am/pm	O ____ am/pm	O ____ am/pm
______________	O ____ am/pm	O ____ am/pm	O ____ am/pm	O ____ am/pm	O ____ am/pm
______________	O ____ am/pm	O ____ am/pm	O ____ am/pm	O ____ am/pm	O ____ am/pm
______________	O ____ am/pm	O ____ am/pm	O ____ am/pm	O ____ am/pm	O ____ am/pm
______________	O ____ am/pm	O ____ am/pm	O ____ am/pm	O ____ am/pm	O ____ am/pm

For each question, please tick (✓) your answer on O or write you answer on ______

Registration ID

Thank you for filling the diary today.
Now please give your child the study medicine.

Notes:

Registration ID | |

Day – 1* : |___|___| – |___|___| – 20 |___|___|

***On the morning after your first visit to the hospital or doctor**

1. Please place a vertical line across the available horizontal line that best describes your or your child's pain during the past 12 hours? Please write the time accordingly ……. (am/ pm)

No Pain |————————————————| Pain As Bad As It Could Possibly Be

2. We are interest finding out how your child has been doing. For each question, please place a check mark in O corresponding to your child's symptoms. Please answer all questions. Please write the time accordingly ……. (am/ pm)

2.1 Over the past 12 h, has your child been tugging, rubbing, or holding the ear(s) more than usual?	O No	O A little	O A lot
2.2 Over the past 12 h, has your child been crying more than usual?	O No	O A little	O A lot
2.3 Over the past 12 h, has your child been more irritable or fussy than usual?	O No	O A little	O A lot
2.4 Over the past 12 h, has your child been having more difficulty sleeping than usual?	O No	O A little	O A lot
2.5 Over the past 12 h, has your child been less playful or active than usual?	O No	O A little	O A lot
2.6 Over the past 12 h, has your child been eating less than usual?	O No	O A little	O A lot
2.7 Over the past 12 h, has your child been having fever or feeling warm to touch?	O No	O A little	O A lot

Other symptoms

3 Does your child experience discharge from the ear(s)?	O Yes	O No
4 Does your child experience intense ear pain and pain behind the ear?	O Yes	O No
5 Does your child experience swelling/bulging, redness, tenderness, or dropping behind or of the ear(s)	O Yes	O No
6 Does your child experience facial asymmetry (e.g. when the child smiles, cries)?	O Yes	O No

7 Side effects

Does your child have these complaints after taking the medicine

7.1 Increased appetite	O Yes	O No	7.8 Drowsiness	O Yes	O No
7.2 Increased urine amount	O Yes	O No	7.9 Anxiety/distractibility/mood swing	O Yes	O No
7.3 Weight gain	O Yes	O No	7.10 Headache	O Yes	O No
7.4 Gastritis/abdominal pain	O Yes	O No	7.11 Skin rash or diaper rash	O Yes	O No
7.5 Nausea	O Yes	O No	7.12 Candidiasis	O Yes	O No
7.6 Vomiting	O Yes	O No	7.13 Dry mouth / throat irritation	O Yes	O No
7.7 Diarrhea	O Yes	O No	7.14 Sleep disturbance	O Yes	O No
Others	______________________				
Did you bring your child to doctor (clinic or outpatient)?	O Yes	O No	Reason:	______________________	
			Medicine prescribed:	______________________	

Has your child has been admitted to hospital?	O Yes O No	Reason:	________________
		Medicine prescribed:	________________

**Medicines given (please write the name, dose, and frequency)**			
Additional medicine from the chemist store or other (not precribed by your doctor)	____________	Dose : _____ mg / body weight kg	Frequency : ______ / day
	____________	Dose : _____ mg / body weight kg	Frequency : ______ / day
	____________	Dose : _____ mg / body weight kg	Frequency : ______ / day
	____________	Dose : _____ mg / body weight kg	Frequency : ______ / day
	____________	Dose : _____ mg / body weight kg	Frequency : ______ / day

**Please list all medicines you give to your child today by marking the circle based on the frequency and the time**					
____________	O ___ am/pm	O ___ am/pm	O ___ am/pm	O ___ am/pm	O ___ am/pm
____________	O ___ am/pm	O ___ am/pm	O ___ am/pm	O ___ am/pm	O ___ am/pm
____________	O ___ am/pm	O ___ am/pm	O ___ am/pm	O ___ am/pm	O ___ am/pm
____________	O ___ am/pm	O ___ am/pm	O ___ am/pm	O ___ am/pm	O ___ am/pm
____________	O ___ am/pm	O ___ am/pm	O ___ am/pm	O ___ am/pm	O ___ am/pm
____________	O ___ am/pm	O ___ am/pm	O ___ am/pm	O ___ am/pm	O ___ am/pm
____________	O ___ am/pm	O ___ am/pm	O ___ am/pm	O ___ am/pm	O ___ am/pm

Thank you for filling the diary today.
Now please give your child the study medicine.

Notes:

Day-2 : |___|___| – |___|___| – 20 |___|___|

1. Please place a vertical line across the available horizontal line that best describes your or your child's pain during the past 12 hours? Please write the time accordingly ……. (am/ pm)

No Pain ———————————————— Pain As Bad As It Could Possibly Be

2. We are interest finding out how your child has been doing. For each question, please place a check mark in O corresponding to your child's symptoms. Please answer all questions. Please write the time accordingly ……. (am/ pm)

2.1 Over the past 12 h, has your child been tugging, rubbing, or holding the ear(s) more than usual?	O No	O A little	O A lot
2.2 Over the past 12 h, has your child been crying more than usual?	O No	O A little	O A lot
2.3 Over the past 12 h, has your child been more irritable or fussy than usual?	O No	O A little	O A lot
2.4 Over the past 12 h, has your child been having more difficulty sleeping than usual?	O No	O A little	O A lot
2.5 Over the past 12 h, has your child been less playful or active than usual?	O No	O A little	O A lot
2.6 Over the past 12 h, has your child been eating less than usual?	O No	O A little	O A lot
2.7 Over the past 12 h, has your child been having fever or feeling warm to touch?	O No	O A little	O A lot

Other symptoms

3 Does your child experience discharge from the ear(s)?	O Yes	O No
4 Does your child experience intense ear pain and pain behind the ear?	O Yes	O No
5 Does your child experience swelling/bulging, redness, tenderness, or dropping behind or of the ear(s)	O Yes	O No
6 Does your child experience facial asymmetry (e.g. when the child smiles, cries)?	O Yes	O No

Medicines given (please write the name, dose, and frequency)

Medicines have been given to your child before going to the hospital (from other doctor or chemist store)	______________________	Dose : _____ mg / body weight kg	Frequency : ______ / day
	______________________	Dose : _____ mg / body weight kg	Frequency : ______ / day
	______________________	Dose : _____ mg / body weight kg	Frequency : ______ / day
	______________________	Dose : _____ mg / body weight kg	Frequency : ______ / day
	______________________	Dose : _____ mg / body weight kg	Frequency : ______ / day

7 Side effects

Does your child have these complaints after taking the medicine

7.1 Increased appetite	O Yes	O No	7.8 Drowsiness	O Yes	O No
7.2 Increased urine amount	O Yes	O No	7.9 Anxiety/distractibility/mood swing	O Yes	O No
7.3 Weight gain	O Yes	O No	7.10 Headache	O Yes	O No
7.4 Gastritis/abdominal pain	O Yes	O No	7.11 Skin rash or diaper rash	O Yes	O No
7.5 Nausea	O Yes	O No	7.12 Candidiasis	O Yes	O No
7.6 Vomiting	O Yes	O No	7.13 Dry mouth / throat irritation	O Yes	O No
7.7 Diarrhea	O Yes	O No	7.14 Sleep disturbance	O Yes	O No

For each question, please tick (✓) your answer on O or write you answer on ______

Others	______________________________			
Did you bring your child to doctor (clinic or outpatient)?	O Yes	O No	Reason:	____________ ____________
			Medicine prescribed:	____________ ____________
Has your child has been admitted to hospital?	O Yes	O No	Reason:	____________ ____________
			Medicine prescribed:	____________ ____________

Medicines given (please write the name, dose, and frequency)

Medicines have been given to your child before going to the hospital (from other doctor or chemist store)	____________	Dose : _____ mg / body weight kg	Frequency : ______ / day
	____________	Dose : _____ mg / body weight kg	Frequency : ______ / day
	____________	Dose : _____ mg / body weight kg	Frequency : ______ / day
	____________	Dose : _____ mg / body weight kg	Frequency : ______ / day
	____________	Dose : _____ mg / body weight kg	Frequency : ______ / day

Please list all medicines you give to your child today by marking the circle based on the frequency and the time

____________	O ___ am/pm	O ___ am/pm	O ___ am/pm	O ___ am/pm	O ___ am/pm
____________	O ___ am/pm	O ___ am/pm	O ___ am/pm	O ___ am/pm	O ___ am/pm
____________	O ___ am/pm	O ___ am/pm	O ___ am/pm	O ___ am/pm	O ___ am/pm
____________	O ___ am/pm	O ___ am/pm	O ___ am/pm	O ___ am/pm	O ___ am/pm
____________	O ___ am/pm	O ___ am/pm	O ___ am/pm	O ___ am/pm	O ___ am/pm
____________	O ___ am/pm	O ___ am/pm	O ___ am/pm	O ___ am/pm	O ___ am/pm
____________	O ___ am/pm	O ___ am/pm	O ___ am/pm	O ___ am/pm	O ___ am/pm

Thank you for filling the diary today.
Now please give your child the study medicine.

Notes:

Registration ID ______

Day – 3 (1st Follow-up Visit): |___|___| – |___|___| – 20 |___|___|

1. Please place a vertical line across the available horizontal line that best describes your or your child's pain during the past 12 hours? Please write the time accordingly ……. (am/ pm)

No Pain |——————————————————| Pain As Bad As It Could Possibly Be

2. We are interest finding out how your child has been doing. For each question, please place a check mark in O corresponding to your child's symptoms. Please answer all questions. Please write the time accordingly ……. (am/ pm)

2.1 Over the past 12 h, has your child been tugging, rubbing, or holding the ear(s) more than usual?	O No	O A little	O A lot
2.2 Over the past 12 h, has your child been crying more than usual?	O No	O A little	O A lot
2.3 Over the past 12 h, has your child been more irritable or fussy than usual?	O No	O A little	O A lot
2.4 Over the past 12 h, has your child been having more difficulty sleeping than usual?	O No	O A little	O A lot
2.5 Over the past 12 h, has your child been less playful or active than usual?	O No	O A little	O A lot
2.6 Over the past 12 h, has your child been eating less than usual?	O No	O A little	O A lot
2.7 Over the past 12 h, has your child been having fever or feeling warm to touch?	O No	O A little	O A lot

Other symptoms

3 Does your child experience discharge from the ear(s)?	O Yes	O No
4 Does your child experience intense ear pain and pain behind the ear?	O Yes	O No
5 Does your child experience swelling/bulging, redness, tenderness, or dropping behind or of the ear(s)	O Yes	O No
6 Does your child experience facial asymmetry (e.g. when the child smiles, cries)?	O Yes	O No

7 Side effects

Does your child have these complaints after taking the medicine

7.1 Increased appetite	O Yes	O No	7.8 Drowsiness	O Yes	O No
7.2 Increased urine amount	O Yes	O No	7.9 Anxiety/distractibility/mood swing	O Yes	O No
7.3 Weight gain	O Yes	O No	7.10 Headache	O Yes	O No
7.4 Gastritis/abdominal pain	O Yes	O No	7.11 Skin rash or diaper rash	O Yes	O No
7.5 Nausea	O Yes	O No	7.12 Candidiasis	O Yes	O No
7.6 Vomiting	O Yes	O No	7.13 Dry mouth / throat irritation	O Yes	O No
7.7 Diarrhea	O Yes	O No	7.14 Sleep disturbance	O Yes	O No
Others	______				

Did you bring your child to doctor (clinic or outpatient)?	O Yes	O No	Reason:	______
			Medicine prescribed:	______
Has your child has been admitted to hospital?	O Yes	O No	Reason:	______
			Medicine prescribed:	______

For each question, please tick (✓) your answer on O or write you answer on ______

**Medicines given (please write the name, dose, and frequency)**			
Additional medicine from the chemist store or other (not precribed by your doctor)	____________________	Dose : _____ mg / body weight kg	Frequency : ______/ day
	____________________	Dose : _____ mg / body weight kg	Frequency : ______/ day
	____________________	Dose : _____ mg / body weight kg	Frequency : ______/ day
	____________________	Dose : _____ mg / body weight kg	Frequency : ______/ day
	____________________	Dose : _____ mg / body weight kg	Frequency : ______/ day

**Please list all medicines you give to your child today by marking the circle based on the frequency and the time**					
____________________	O ____ am/pm	O ____ am/pm	O ____ am/pm	O ____ am/pm	O ____ am/pm
____________________	O ____ am/pm	O ____ am/pm	O ____ am/pm	O ____ am/pm	O ____ am/pm
____________________	O ____ am/pm	O ____ am/pm	O ____ am/pm	O ____ am/pm	O ____ am/pm
____________________	O ____ am/pm	O ____ am/pm	O ____ am/pm	O ____ am/pm	O ____ am/pm
____________________	O ____ am/pm	O ____ am/pm	O ____ am/pm	O ____ am/pm	O ____ am/pm
____________________	O ____ am/pm	O ____ am/pm	O ____ am/pm	O ____ am/pm	O ____ am/pm
____________________	O ____ am/pm	O ____ am/pm	O ____ am/pm	O ____ am/pm	O ____ am/pm

Thank you for filling the diary today.
Now please give your child the study medicine.

Notes:

Thank you for completing the first Diary.

Registration ID | |

Additional Visit : |___|___| – |___|___| – 20 |___|___|

1. Please place a vertical line across the available horizontal line that best describes your or your child's pain during the past 12 hours? Please write the time accordingly ……. (am/ pm)

No Pain |————————————————| Pain As Bad As It Could Possibly Be

2. We are interest finding out how your child has been doing. For each question, please place a check mark in O corresponding to your child's symptoms. Please answer all questions. Please write the time accordingly ……. (am/ pm)

2.1 Over the past 12 h, has your child been tugging, rubbing, or holding the ear(s) more than usual?	O No	O A little	O A lot
2.2 Over the past 12 h, has your child been crying more than usual?	O No	O A little	O A lot
2.3 Over the past 12 h, has your child been more irritable or fussy than usual?	O No	O A little	O A lot
2.4 Over the past 12 h, has your child been having more difficulty sleeping than usual?	O No	O A little	O A lot
2.5 Over the past 12 h, has your child been less playful or active than usual?	O No	O A little	O A lot
2.6 Over the past 12 h, has your child been eating less than usual?	O No	O A little	O A lot
2.7 Over the past 12 h, has your child been having fever or feeling warm to touch?	O No	O A little	O A lot

Other symptoms

3 Does your child experience discharge from the ear(s)?	O Yes	O No
4 Does your child experience intense ear pain and pain behind the ear?	O Yes	O No
5 Does your child experience swelling/bulging, redness, tenderness, or dropping behind or of the ear(s)	O Yes	O No
6 Does your child experience facial asymmetry (e.g. when the child smiles, cries)?	O Yes	O No

7 Side effects

Does your child have these complaints after taking the medicine

7.1 Increased appetite	O Yes	O No	7.8 Drowsiness	O Yes	O No
7.2 Increased urine amount	O Yes	O No	7.9 Anxiety/distractibility/mood swing	O Yes	O No
7.3 Weight gain	O Yes	O No	7.10 Headache	O Yes	O No
7.4 Gastritis/abdominal pain	O Yes	O No	7.11 Skin rash or diaper rash	O Yes	O No
7.5 Nausea	O Yes	O No	7.12 Candidiasis	O Yes	O No
7.6 Vomiting	O Yes	O No	7.13 Dry mouth / throat irritation	O Yes	O No
7.7 Diarrhea	O Yes	O No	7.14 Sleep disturbance	O Yes	O No
Others	______				

Did you bring your child to doctor (clinic or outpatient)?	O Yes	O No	Reason:	______
			Medicine prescribed:	______
Has your child has been admitted to hospital?	O Yes	O No	Reason:	______
			Medicine prescribed:	______

For each question, please tick (✓) your answer on O or write you answer on ______

**Medicines given (please write the name, dose, and frequency)**			
Additional medicine from the chemist store or other (not precribed by your doctor)	____________________	Dose : _____ mg / body weight kg	Frequency : ______ / day
	____________________	Dose : _____ mg / body weight kg	Frequency : ______ / day
	____________________	Dose : _____ mg / body weight kg	Frequency : ______ / day
	____________________	Dose : _____ mg / body weight kg	Frequency : ______ / day
	____________________	Dose : _____ mg / body weight kg	Frequency : ______ / day

**Please list all medicines you give to your child today by marking the circle based on the frequency and the time**					
____________________	O ____ am/pm	O ____ am/pm	O ____ am/pm	O ____ am/pm	O ____ am/pm
____________________	O ____ am/pm	O ____ am/pm	O ____ am/pm	O ____ am/pm	O ____ am/pm
____________________	O ____ am/pm	O ____ am/pm	O ____ am/pm	O ____ am/pm	O ____ am/pm
____________________	O ____ am/pm	O ____ am/pm	O ____ am/pm	O ____ am/pm	O ____ am/pm
____________________	O ____ am/pm	O ____ am/pm	O ____ am/pm	O ____ am/pm	O ____ am/pm
____________________	O ____ am/pm	O ____ am/pm	O ____ am/pm	O ____ am/pm	O ____ am/pm
____________________	O ____ am/pm	O ____ am/pm	O ____ am/pm	O ____ am/pm	O ____ am/pm

Notes:

DIARY – 2 (Day-4 to Day-7)

OPAL Study

Oral Prednisolone for Acute otitis media in children

Registration ID

Hello Uncle / Aunty!!

My name is______________________

I was born in_________________

On date _____ month ________ year ___________

If you find this Diary, I would be very grateful if you can return it to my Dad (mobile no. ________________) or my Mom (mobile no. ________________).

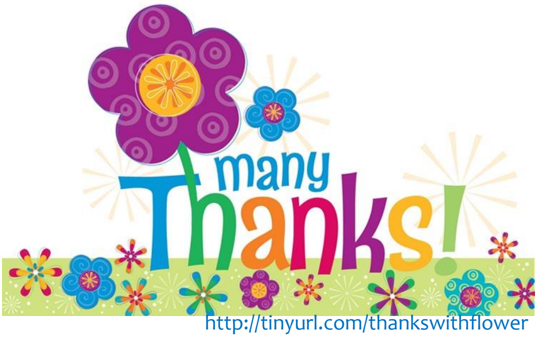

http://tinyurl.com/thankswithflower

Registration ID | |

Day-4 : |___|___| – |___|___| – 20 |___|___|

1. Please place a vertical line across the available horizontal line that best describes your or your child's pain during the past 12 hours? Please write the time accordingly ……. (am/ pm)

No Pain |————————————————| Pain As Bad As It Could Possibly Be

2. We are interest finding out how your child has been doing. For each question, please place a check mark in O corresponding to your child's symptoms. Please answer all questions. Please write the time accordingly ……. (am/ pm)

2.1 Over the past 12 h, has your child been tugging, rubbing, or holding the ear(s) more than usual?	O No	O A little	O A lot
2.2 Over the past 12 h, has your child been crying more than usual?	O No	O A little	O A lot
2.3 Over the past 12 h, has your child been more irritable or fussy than usual?	O No	O A little	O A lot
2.4 Over the past 12 h, has your child been having more difficulty sleeping than usual?	O No	O A little	O A lot
2.5 Over the past 12 h, has your child been less playful or active than usual?	O No	O A little	O A lot
2.6 Over the past 12 h, has your child been eating less than usual?	O No	O A little	O A lot
2.7 Over the past 12 h, has your child been having fever or feeling warm to touch?	O No	O A little	O A lot

Other symptoms

3 Does your child experience discharge from the ear(s)?	O Yes	O No
4 Does your child experience intense ear pain and pain behind the ear?	O Yes	O No
5 Does your child experience swelling/bulging, redness, tenderness, or dropping behind or of the ear(s)	O Yes	O No
6 Does your child experience facial asymmetry (e.g. when the child smiles, cries)?	O Yes	O No

7 Side effects

Does your child have these complaints after taking the medicine

7.1 Increased appetite	O Yes	O No	7.8 Drowsiness	O Yes	O No
7.2 Increased urine amount	O Yes	O No	7.9 Anxiety/distractibility/mood swing	O Yes	O No
7.3 Weight gain	O Yes	O No	7.10 Headache	O Yes	O No
7.4 Gastritis/abdominal pain	O Yes	O No	7.11 Skin rash or diaper rash	O Yes	O No
7.5 Nausea	O Yes	O No	7.12 Candidiasis	O Yes	O No
7.6 Vomiting	O Yes	O No	7.13 Dry mouth / throat irritation	O Yes	O No
7.7 Diarrhea	O Yes	O No	7.14 Sleep disturbance	O Yes	O No
Others	______				

Did you bring your child to doctor (clinic or outpatient)?	O Yes	O No	Reason:	______
			Medicine prescribed:	______
Has your child has been admitted to hospital?	O Yes	O No	Reason:	______
			Medicine prescribed:	______

For each question, please tick (✓) your answer on O or write you answer on ______

Registration ID ______

**Medicines given (please write the name, dose, and frequency)**			
Additional medicine from the chemist store or other (not precribed by your doctor)	______	Dose : _____ mg / body weight kg	Frequency : ______ / day
	______	Dose : _____ mg / body weight kg	Frequency : ______ / day
	______	Dose : _____ mg / body weight kg	Frequency : ______ / day
	______	Dose : _____ mg / body weight kg	Frequency : ______ / day
	______	Dose : _____ mg / body weight kg	Frequency : ______ / day

**Please list all medicines you give to your child today by marking the circle based on the frequency and the time**					
______	O ____ am/pm	O ____ am/pm	O ____ am/pm	O ____ am/pm	O ____ am/pm
______	O ____ am/pm	O ____ am/pm	O ____ am/pm	O ____ am/pm	O ____ am/pm
______	O ____ am/pm	O ____ am/pm	O ____ am/pm	O ____ am/pm	O ____ am/pm
______	O ____ am/pm	O ____ am/pm	O ____ am/pm	O ____ am/pm	O ____ am/pm
______	O ____ am/pm	O ____ am/pm	O ____ am/pm	O ____ am/pm	O ____ am/pm
______	O ____ am/pm	O ____ am/pm	O ____ am/pm	O ____ am/pm	O ____ am/pm
______	O ____ am/pm	O ____ am/pm	O ____ am/pm	O ____ am/pm	O ____ am/pm

Thank you for filling the diary today.
Now please give your child the study medicine.

Notes:

Registration ID

Day – 5 : |___|___| – |___|___| – 20 |___|___|

1. Please place a vertical line across the available horizontal line that best describes your or your child's pain during the past 12 hours? Please write the time accordingly ……. (am/ pm)

No Pain |————————————————| Pain As Bad As It Could Possibly Be

2. We are interest finding out how your child has been doing. For each question, please place a check mark in O corresponding to your child's symptoms. Please answer all questions. Please write the time accordingly …… (am/ pm)

2.1 Over the past 12 h, has your child been tugging, rubbing, or holding the ear(s) more than usual?	O No	O A little	O A lot
2.2 Over the past 12 h, has your child been crying more than usual?	O No	O A little	O A lot
2.3 Over the past 12 h, has your child been more irritable or fussy than usual?	O No	O A little	O A lot
2.4 Over the past 12 h, has your child been having more difficulty sleeping than usual?	O No	O A little	O A lot
2.5 Over the past 12 h, has your child been less playful or active than usual?	O No	O A little	O A lot
2.6 Over the past 12 h, has your child been eating less than usual?	O No	O A little	O A lot
2.7 Over the past 12 h, has your child been having fever or feeling warm to touch?	O No	O A little	O A lot

Other symptoms

3 Does your child experience discharge from the ear(s)?	O Yes	O No
4 Does your child experience intense ear pain and pain behind the ear?	O Yes	O No
5 Does your child experience swelling/bulging, redness, tenderness, or dropping behind or of the ear(s)	O Yes	O No
6 Does your child experience facial asymmetry (e.g. when the child smiles, cries)?	O Yes	O No

7 Side effects

Does your child have these complaints after taking the medicine

7.1 Increased appetite	O Yes	O No	7.8 Drowsiness	O Yes	O No
7.2 Increased urine amount	O Yes	O No	7.9 Anxiety/distractibility/mood swing	O Yes	O No
7.3 Weight gain	O Yes	O No	7.10 Headache	O Yes	O No
7.4 Gastritis/abdominal pain	O Yes	O No	7.11 Skin rash or diaper rash	O Yes	O No
7.5 Nausea	O Yes	O No	7.12 Candidiasis	O Yes	O No
7.6 Vomiting	O Yes	O No	7.13 Dry mouth / throat irritation	O Yes	O No
7.7 Diarrhea	O Yes	O No	7.14 Sleep disturbance	O Yes	O No
Others	______				

Did you bring your child to doctor (clinic or outpatient)?	O Yes	O No	Reason:	______
			Medicine prescribed:	______
Has your child has been admitted to hospital?	O Yes	O No	Reason:	______
			Medicine prescribed:	______

For each question, please tick (✓) your answer on O or write you answer on ______

Registration ID | |

**Medicines given (please write the name, dose, and frequency)**			
Additional medicine from the chemist store or other (not precribed by your doctor)	________________	Dose : _____ mg / body weight kg	Frequency : ______ / day
	________________	Dose : _____ mg / body weight kg	Frequency : ______ / day
	________________	Dose : _____ mg / body weight kg	Frequency : ______ / day
	________________	Dose : _____ mg / body weight kg	Frequency : ______ / day
	________________	Dose : _____ mg / body weight kg	Frequency : ______ / day

**Please list all medicines you give to your child today by marking the circle based on the frequency and the time**					
________________	O ____ am/pm	O ____ am/pm	O ____ am/pm	O ____ am/pm	O ____ am/pm
________________	O ____ am/pm	O ____ am/pm	O ____ am/pm	O ____ am/pm	O ____ am/pm
________________	O ____ am/pm	O ____ am/pm	O ____ am/pm	O ____ am/pm	O ____ am/pm
________________	O ____ am/pm	O ____ am/pm	O ____ am/pm	O ____ am/pm	O ____ am/pm
________________	O ____ am/pm	O ____ am/pm	O ____ am/pm	O ____ am/pm	O ____ am/pm
________________	O ____ am/pm	O ____ am/pm	O ____ am/pm	O ____ am/pm	O ____ am/pm
________________	O ____ am/pm	O ____ am/pm	O ____ am/pm	O ____ am/pm	O ____ am/pm

Thank you for filling the diary today.
Now please give your child the study medicine.

Notes:

Registration ID

Day-6 : |___|___| – |___|___| – 20 |___|___|

1. Please place a vertical line across the available horizontal line that best describes your or your child's pain during the past 12 hours? Please write the time accordingly ……. (am/ pm)

No Pain ——————————————————————— Pain As Bad As It Could Possibly Be

2. We are interest finding out how your child has been doing. For each question, please place a check mark in O corresponding to your child's symptoms. Please answer all questions. Please write the time accordingly ……. (am/ pm)

2.1 Over the past 12 h, has your child been tugging, rubbing, or holding the ear(s) more than usual?	O No	O A little	O A lot
2.2 Over the past 12 h, has your child been crying more than usual?	O No	O A little	O A lot
2.3 Over the past 12 h, has your child been more irritable or fussy than usual?	O No	O A little	O A lot
2.4 Over the past 12 h, has your child been having more difficulty sleeping than usual?	O No	O A little	O A lot
2.5 Over the past 12 h, has your child been less playful or active than usual?	O No	O A little	O A lot
2.6 Over the past 12 h, has your child been eating less than usual?	O No	O A little	O A lot
2.7 Over the past 12 h, has your child been having fever or feeling warm to touch?	O No	O A little	O A lot

Other symptoms

3 Does your child experience discharge from the ear(s)?	O Yes	O No
4 Does your child experience intense ear pain and pain behind the ear?	O Yes	O No
5 Does your child experience swelling/bulging, redness, tenderness, or dropping behind or of the ear(s)	O Yes	O No
6 Does your child experience facial asymmetry (e.g. when the child smiles, cries)?	O Yes	O No

7 Side effects

Does your child have these complaints after taking the medicine

7.1 Increased appetite	O Yes	O No	7.8 Drowsiness	O Yes	O No
7.2 Increased urine amount	O Yes	O No	7.9 Anxiety/distractibility/mood swing	O Yes	O No
7.3 Weight gain	O Yes	O No	7.10 Headache	O Yes	O No
7.4 Gastritis/abdominal pain	O Yes	O No	7.11 Skin rash or diaper rash	O Yes	O No
7.5 Nausea	O Yes	O No	7.12 Candidiasis	O Yes	O No
7.6 Vomiting	O Yes	O No	7.13 Dry mouth / throat irritation	O Yes	O No
7.7 Diarrhea	O Yes	O No	7.14 Sleep disturbance	O Yes	O No
Others	____________________				

Did you bring your child to doctor (clinic or outpatient)?	O Yes	O No	Reason:	__________
			Medicine prescribed:	__________
Has your child has been admitted to hospital?	O Yes	O No	Reason:	__________
			Medicine prescribed:	__________

For each question, please tick (✓) your answer on O or write you answer on ______

**Medicines given (please write the name, dose, and frequency)**	
Additional medicine from the chemist store or other (not precribed by your doctor)	______________ Dose : _____ mg / body weight kg Frequency : ______ / day
	______________ Dose : _____ mg / body weight kg Frequency : ______ / day
	______________ Dose : _____ mg / body weight kg Frequency : ______ / day
	______________ Dose : _____ mg / body weight kg Frequency : ______ / day
	______________ Dose : _____ mg / body weight kg Frequency : ______ / day

**Please list all medicines you give to your child today by marking the circle based on the frequency and the time**					
______________	O ____ am/pm	O ____ am/pm	O ____ am/pm	O ____ am/pm	O ____ am/pm
______________	O ____ am/pm	O ____ am/pm	O ____ am/pm	O ____ am/pm	O ____ am/pm
______________	O ____ am/pm	O ____ am/pm	O ____ am/pm	O ____ am/pm	O ____ am/pm
______________	O ____ am/pm	O ____ am/pm	O ____ am/pm	O ____ am/pm	O ____ am/pm
______________	O ____ am/pm	O ____ am/pm	O ____ am/pm	O ____ am/pm	O ____ am/pm
______________	O ____ am/pm	O ____ am/pm	O ____ am/pm	O ____ am/pm	O ____ am/pm
______________	O ____ am/pm	O ____ am/pm	O ____ am/pm	O ____ am/pm	O ____ am/pm

Notes:

Registration ID

Day – 7 (2nd Follow-up Visit): |___|___| – |___|___| – 20 |___|___|

1. Please place a vertical line across the available horizontal line that best describes your or your child's pain during the past 12 hours? Please write the time accordingly ……. (am/ pm)

No Pain |————————————————| Pain As Bad As It Could Possibly Be

2. We are interest finding out how your child has been doing. For each question, please place a check mark in O corresponding to your child's symptoms. Please answer all questions. Please write the time accordingly ……. (am/ pm)

2.1 Over the past 12 h, has your child been tugging, rubbing, or holding the ear(s) more than usual?	O No	O A little	O A lot
2.2 Over the past 12 h, has your child been crying more than usual?	O No	O A little	O A lot
2.3 Over the past 12 h, has your child been more irritable or fussy than usual?	O No	O A little	O A lot
2.4 Over the past 12 h, has your child been having more difficulty sleeping than usual?	O No	O A little	O A lot
2.5 Over the past 12 h, has your child been less playful or active than usual?	O No	O A little	O A lot
2.6 Over the past 12 h, has your child been eating less than usual?	O No	O A little	O A lot
2.7 Over the past 12 h, has your child been having fever or feeling warm to touch?	O No	O A little	O A lot

Other symptoms

3 Does your child experience discharge from the ear(s)?	O Yes	O No
4 Does your child experience intense ear pain and pain behind the ear?	O Yes	O No
5 Does your child experience swelling/bulging, redness, tenderness, or dropping behind or of the ear(s)	O Yes	O No
6 Does your child experience facial asymmetry (e.g. when the child smiles, cries)?	O Yes	O No

7 Side effects

Does your child have these complaints after taking the medicine

7.1 Increased appetite	O Yes	O No	7.8 Drowsiness	O Yes	O No
7.2 Increased urine amount	O Yes	O No	7.9 Anxiety/distractibility/mood swing	O Yes	O No
7.3 Weight gain	O Yes	O No	7.10 Headache	O Yes	O No
7.4 Gastritis/abdominal pain	O Yes	O No	7.11 Skin rash or diaper rash	O Yes	O No
7.5 Nausea	O Yes	O No	7.12 Candidiasis	O Yes	O No
7.6 Vomiting	O Yes	O No	7.13 Dry mouth / throat irritation	O Yes	O No
7.7 Diarrhea	O Yes	O No	7.14 Sleep disturbance	O Yes	O No
Others	______				

Did you bring your child to doctor (clinic or outpatient)?	O Yes	O No	Reason:	______ ______
			Medicine prescribed:	______ ______
Has your child has been admitted to hospital?	O Yes	O No	Reason:	______
			Medicine prescribed:	______ ______

For each question, please tick (✓) your answer on O or write you answer on ______

Registration ID

**Medicines given (please write the name, dose, and frequency)**			
Additional medicine from the chemist store or other (not precribed by your doctor)	______________________	Dose : _____ mg / body weight kg	Frequency : ______ / day
	______________________	Dose : _____ mg / body weight kg	Frequency : ______ / day
	______________________	Dose : _____ mg / body weight kg	Frequency : ______ / day
	______________________	Dose : _____ mg / body weight kg	Frequency : ______ / day
	______________________	Dose : _____ mg / body weight kg	Frequency : ______ / day

**Please list all medicines you give to your child today by marking the circle based on the frequency and the time**					
______________________	O ____ am/pm	O ____ am/pm	O ____ am/pm	O ____ am/pm	O ____ am/pm
______________________	O ____ am/pm	O ____ am/pm	O ____ am/pm	O ____ am/pm	O ____ am/pm
______________________	O ____ am/pm	O ____ am/pm	O ____ am/pm	O ____ am/pm	O ____ am/pm
______________________	O ____ am/pm	O ____ am/pm	O ____ am/pm	O ____ am/pm	O ____ am/pm
______________________	O ____ am/pm	O ____ am/pm	O ____ am/pm	O ____ am/pm	O ____ am/pm
______________________	O ____ am/pm	O ____ am/pm	O ____ am/pm	O ____ am/pm	O ____ am/pm
______________________	O ____ am/pm	O ____ am/pm	O ____ am/pm	O ____ am/pm	O ____ am/pm

Notes:

Thank you for completing the first Diary.

Registration ID | |

Additional Visit : |___|___| – |___|___| – 20 |___|___|

1. Please place a vertical line across the available horizontal line that best describes your or your child's pain during the past 12 hours? Please write the time accordingly ……. (am/ pm)

No Pain |————————————————| Pain As Bad As It Could Possibly Be

2. We are interest finding out how your child has been doing. For each question, please place a check mark in O corresponding to your child's symptoms. Please answer all questions. Please write the time accordingly ……. (am/ pm)

2.1 Over the past 12 h, has your child been tugging, rubbing, or holding the ear(s) more than usual?	O No	O A little	O A lot
2.2 Over the past 12 h, has your child been crying more than usual?	O No	O A little	O A lot
2.3 Over the past 12 h, has your child been more irritable or fussy than usual?	O No	O A little	O A lot
2.4 Over the past 12 h, has your child been having more difficulty sleeping than usual?	O No	O A little	O A lot
2.5 Over the past 12 h, has your child been less playful or active than usual?	O No	O A little	O A lot
2.6 Over the past 12 h, has your child been eating less than usual?	O No	O A little	O A lot
2.7 Over the past 12 h, has your child been having fever or feeling warm to touch?	O No	O A little	O A lot

Other symptoms

3 Does your child experience discharge from the ear(s)?	O Yes	O No
4 Does your child experience intense ear pain and pain behind the ear?	O Yes	O No
5 Does your child experience swelling/bulging, redness, tenderness, or dropping behind or of the ear(s)	O Yes	O No
6 Does your child experience facial asymmetry (e.g. when the child smiles, cries)?	O Yes	O No

7 Side effects

Does your child have these complaints after taking the medicine

7.1 Increased appetite	O Yes	O No	7.8 Drowsiness	O Yes	O No
7.2 Increased urine amount	O Yes	O No	7.9 Anxiety/distractibility/mood swing	O Yes	O No
7.3 Weight gain	O Yes	O No	7.10 Headache	O Yes	O No
7.4 Gastritis/abdominal pain	O Yes	O No	7.11 Skin rash or diaper rash	O Yes	O No
7.5 Nausea	O Yes	O No	7.12 Candidiasis	O Yes	O No
7.6 Vomiting	O Yes	O No	7.13 Dry mouth / throat irritation	O Yes	O No
7.7 Diarrhea	O Yes	O No	7.14 Sleep disturbance	O Yes	O No
Others	______				

Did you bring your child to doctor (clinic or outpatient)?	O Yes	O No	Reason:	______
			Medicine prescribed:	______
Has your child has been admitted to hospital?	O Yes	O No	Reason:	______
			Medicine prescribed:	______

For each question, please tick (✓) your answer on O or write you answer on ______

Registration ID

Medicines given (please write the name, dose, and frequency)			
Additional medicine from the chemist store or other (not precribed by your doctor)	______________	Dose : _____ mg / body weight kg	Frequency : ______ / day
	______________	Dose : _____ mg / body weight kg	Frequency : ______ / day
	______________	Dose : _____ mg / body weight kg	Frequency : ______ / day
	______________	Dose : _____ mg / body weight kg	Frequency : ______ / day
	______________	Dose : _____ mg / body weight kg	Frequency : ______ / day

Please list all medicines you give to your child today by marking the circle based on the frequency and the time					
______________	O ____ am/pm	O ____ am/pm	O ____ am/pm	O ____ am/pm	O ____ am/pm
______________	O ____ am/pm	O ____ am/pm	O ____ am/pm	O ____ am/pm	O ____ am/pm
______________	O ____ am/pm	O ____ am/pm	O ____ am/pm	O ____ am/pm	O ____ am/pm
______________	O ____ am/pm	O ____ am/pm	O ____ am/pm	O ____ am/pm	O ____ am/pm
______________	O ____ am/pm	O ____ am/pm	O ____ am/pm	O ____ am/pm	O ____ am/pm
______________	O ____ am/pm	O ____ am/pm	O ____ am/pm	O ____ am/pm	O ____ am/pm
______________	O ____ am/pm	O ____ am/pm	O ____ am/pm	O ____ am/pm	O ____ am/pm

Notes:

Thank you for completing the second Diary.

DIARY-3 (Day-8 to Day-14)

OPAL Study

Oral Prednisolone for Acute otitis media in children

Registration ID

Hello Uncle / Aunty!!

My name is_______________________

I was born in_________________

On date _____ month ________ year ___________

If you find this Diary, I would be very grateful if you can return it to my Dad (mobile no. _______________) or my Mom (mobile no. _______________).

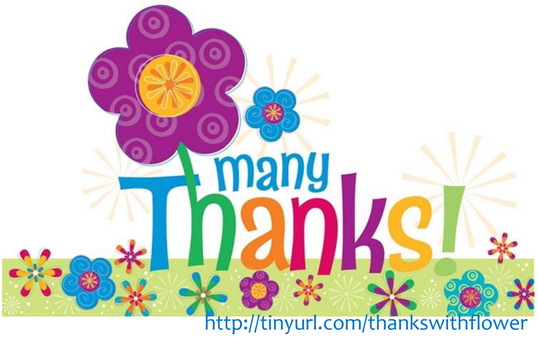

Registration ID | |

Day – 8 : |___|___| – |___|___| – 20 |___|___|

1. Please place a vertical line across the available horizontal line that best describes your or your child's pain during the past 12 hours? Please write the time accordingly ……. (am/ pm)

No Pain |————————————————| Pain As Bad As It Could Possibly Be

2. We are interest finding out how your child has been doing. For each question, please place a check mark in O corresponding to your child's symptoms. Please answer all questions. Please write the time accordingly ……. (am/ pm)

2.1 Over the past 12 h, has your child been tugging, rubbing, or holding the ear(s) more than usual?	O No	O A little	O A lot
2.2 Over the past 12 h, has your child been crying more than usual?	O No	O A little	O A lot
2.3 Over the past 12 h, has your child been more irritable or fussy than usual?	O No	O A little	O A lot
2.4 Over the past 12 h, has your child been having more difficulty sleeping than usual?	O No	O A little	O A lot
2.5 Over the past 12 h, has your child been less playful or active than usual?	O No	O A little	O A lot
2.6 Over the past 12 h, has your child been eating less than usual?	O No	O A little	O A lot
2.7 Over the past 12 h, has your child been having fever or feeling warm to touch?	O No	O A little	O A lot

Other symptoms

3 Does your child experience discharge from the ear(s)?	O Yes	O No
4 Does your child experience intense ear pain and pain behind the ear?	O Yes	O No
5 Does your child experience swelling/bulging, redness, tenderness, or dropping behind or of the ear(s)	O Yes	O No
6 Does your child experience facial asymmetry (e.g. when the child smiles, cries)?	O Yes	O No

7 Side effects

Does your child have these complaints after taking the medicine

7.1 Increased appetite	O Yes	O No	7.8 Drowsiness	O Yes	O No
7.2 Increased urine amount	O Yes	O No	7.9 Anxiety/distractibility/mood swing	O Yes	O No
7.3 Weight gain	O Yes	O No	7.10 Headache	O Yes	O No
7.4 Gastritis/abdominal pain	O Yes	O No	7.11 Skin rash or diaper rash	O Yes	O No
7.5 Nausea	O Yes	O No	7.12 Candidiasis	O Yes	O No
7.6 Vomiting	O Yes	O No	7.13 Dry mouth / throat irritation	O Yes	O No
7.7 Diarrhea	O Yes	O No	7.14 Sleep disturbance	O Yes	O No
Others	______________________				

Did you bring your child to doctor (clinic or outpatient)?	O Yes	O No	Reason:	______________________
			Medicine prescribed:	______________________
Has your child has been admitted to hospital?	O Yes	O No	Reason:	______________________
			Medicine prescribed:	______________________

For each question, please tick (✓) your answer on O or write you answer on ______

Registration ID

**Medicines given (please write the name, dose, and frequency)**		
Additional medicine from the chemist store or other (not precribed by your doctor)	____________	Dose : _____ mg / body weight kg Frequency : ______/ day
	____________	Dose : _____ mg / body weight kg Frequency : ______/ day
	____________	Dose : _____ mg / body weight kg Frequency : ______/ day
	____________	Dose : _____ mg / body weight kg Frequency : ______/ day
	____________	Dose : _____ mg / body weight kg Frequency : ______/ day

**Please list all medicines you give to your child today by marking the circle based on the frequency and the time**					
____________	O ____ am/pm	O ____ am/pm	O ____ am/pm	O ____ am/pm	O ____ am/pm
____________	O ____ am/pm	O ____ am/pm	O ____ am/pm	O ____ am/pm	O ____ am/pm
____________	O ____ am/pm	O ____ am/pm	O ____ am/pm	O ____ am/pm	O ____ am/pm
____________	O ____ am/pm	O ____ am/pm	O ____ am/pm	O ____ am/pm	O ____ am/pm
____________	O ____ am/pm	O ____ am/pm	O ____ am/pm	O ____ am/pm	O ____ am/pm
____________	O ____ am/pm	O ____ am/pm	O ____ am/pm	O ____ am/pm	O ____ am/pm
____________	O ____ am/pm	O ____ am/pm	O ____ am/pm	O ____ am/pm	O ____ am/pm

Notes:

Registration ID [ ]

Day – 9 : |___|___| – |___|___| – 20 |___|___|

1. Please place a vertical line across the available horizontal line that best describes your or your child's pain during the past 12 hours? Please write the time accordingly ……. (am/ pm)

No Pain |————————————————| Pain As Bad As It Could Possibly Be

2. We are interest finding out how your child has been doing. For each question, please place a check mark in O corresponding to your child's symptoms. Please answer all questions. Please write the time accordingly ……. (am/ pm)

2.1 Over the past 12 h, has your child been tugging, rubbing, or holding the ear(s) more than usual?	O No	O A little	O A lot
2.2 Over the past 12 h, has your child been crying more than usual?	O No	O A little	O A lot
2.3 Over the past 12 h, has your child been more irritable or fussy than usual?	O No	O A little	O A lot
2.4 Over the past 12 h, has your child been having more difficulty sleeping than usual?	O No	O A little	O A lot
2.5 Over the past 12 h, has your child been less playful or active than usual?	O No	O A little	O A lot
2.6 Over the past 12 h, has your child been eating less than usual?	O No	O A little	O A lot
2.7 Over the past 12 h, has your child been having fever or feeling warm to touch?	O No	O A little	O A lot

Other symptoms

3 Does your child experience discharge from the ear(s)?	O Yes	O No
4 Does your child experience intense ear pain and pain behind the ear?	O Yes	O No
5 Does your child experience swelling/bulging, redness, tenderness, or dropping behind or of the ear(s)	O Yes	O No
6 Does your child experience facial asymmetry (e.g. when the child smiles, cries)?	O Yes	O No

7 Side effects

Does your child have these complaints after taking the medicine

7.1 Increased appetite	O Yes	O No	7.8 Drowsiness	O Yes	O No
7.2 Increased urine amount	O Yes	O No	7.9 Anxiety/distractibility/mood swing	O Yes	O No
7.3 Weight gain	O Yes	O No	7.10 Headache	O Yes	O No
7.4 Gastritis/abdominal pain	O Yes	O No	7.11 Skin rash or diaper rash	O Yes	O No
7.5 Nausea	O Yes	O No	7.12 Candidiasis	O Yes	O No
7.6 Vomiting	O Yes	O No	7.13 Dry mouth / throat irritation	O Yes	O No
7.7 Diarrhea	O Yes	O No	7.14 Sleep disturbance	O Yes	O No
Others	________________				

Did you bring your child to doctor (clinic or outpatient)?	O Yes	O No	Reason:	________________
			Medicine prescribed:	________________
Has your child has been admitted to hospital?	O Yes	O No	Reason:	________________
			Medicine prescribed:	________________

For each question, please tick (✓) your answer on O or write you answer on ______

Registration ID | |

**Medicines given (please write the name, dose, and frequency)**			
Additional medicine from the chemist store or other (not precribed by your doctor)	______________	Dose : _____ mg / body weight kg	Frequency : ______ / day
	______________	Dose : _____ mg / body weight kg	Frequency : ______ / day
	______________	Dose : _____ mg / body weight kg	Frequency : ______ / day
	______________	Dose : _____ mg / body weight kg	Frequency : ______ / day
	______________	Dose : _____ mg / body weight kg	Frequency : ______ / day

**Please list all medicines you give to your child today by marking the circle based on the frequency and the time**					
______________	O ____ am/pm	O ____ am/pm	O ____ am/pm	O ____ am/pm	O ____ am/pm
______________	O ____ am/pm	O ____ am/pm	O ____ am/pm	O ____ am/pm	O ____ am/pm
______________	O ____ am/pm	O ____ am/pm	O ____ am/pm	O ____ am/pm	O ____ am/pm
______________	O ____ am/pm	O ____ am/pm	O ____ am/pm	O ____ am/pm	O ____ am/pm
______________	O ____ am/pm	O ____ am/pm	O ____ am/pm	O ____ am/pm	O ____ am/pm
______________	O ____ am/pm	O ____ am/pm	O ____ am/pm	O ____ am/pm	O ____ am/pm
______________	O ____ am/pm	O ____ am/pm	O ____ am/pm	O ____ am/pm	O ____ am/pm

Notes:

Registration ID | |

Day – 9 : |___|___| – |___|___| – 20 |___|___|

1. Please place a vertical line across the available horizontal line that best describes your or your child's pain during the past 12 hours? Please write the time accordingly ……. (am/ pm)

No Pain |————————————————| Pain As Bad As It Could Possibly Be

2. We are interest finding out how your child has been doing. For each question, please place a check mark in O corresponding to your child's symptoms. Please answer all questions. Please write the time accordingly ……. (am/ pm)

2.1 Over the past 12 h, has your child been tugging, rubbing, or holding the ear(s) more than usual?	O No	O A little	O A lot
2.2 Over the past 12 h, has your child been crying more than usual?	O No	O A little	O A lot
2.3 Over the past 12 h, has your child been more irritable or fussy than usual?	O No	O A little	O A lot
2.4 Over the past 12 h, has your child been having more difficulty sleeping than usual?	O No	O A little	O A lot
2.5 Over the past 12 h, has your child been less playful or active than usual?	O No	O A little	O A lot
2.6 Over the past 12 h, has your child been eating less than usual?	O No	O A little	O A lot
2.7 Over the past 12 h, has your child been having fever or feeling warm to touch?	O No	O A little	O A lot

Other symptoms

3 Does your child experience discharge from the ear(s)?	O Yes	O No
4 Does your child experience intense ear pain and pain behind the ear?	O Yes	O No
5 Does your child experience swelling/bulging, redness, tenderness, or dropping behind or of the ear(s)	O Yes	O No
6 Does your child experience facial asymmetry (e.g. when the child smiles, cries)?	O Yes	O No

7 Side effects

Does your child have these complaints after taking the medicine

7.1 Increased appetite	O Yes	O No	7.8 Drowsiness	O Yes	O No
7.2 Increased urine amount	O Yes	O No	7.9 Anxiety/distractibility/mood swing	O Yes	O No
7.3 Weight gain	O Yes	O No	7.10 Headache	O Yes	O No
7.4 Gastritis/abdominal pain	O Yes	O No	7.11 Skin rash or diaper rash	O Yes	O No
7.5 Nausea	O Yes	O No	7.12 Candidiasis	O Yes	O No
7.6 Vomiting	O Yes	O No	7.13 Dry mouth / throat irritation	O Yes	O No
7.7 Diarrhea	O Yes	O No	7.14 Sleep disturbance	O Yes	O No
Others	________________				

Did you bring your child to doctor (clinic or outpatient)?	O Yes	O No	Reason:	________________
			Medicine prescribed:	________________
Has your child has been admitted to hospital?	O Yes	O No	Reason:	________________
			Medicine prescribed:	________________

For each question, please tick (✓) your answer on O or write you answer on ______

Registration ID | |

**Medicines given (please write the name, dose, and frequency)**	
Additional medicine from the chemist store or other (not precribed by your doctor)	________________ Dose : _____ mg / body weight kg Frequency : ______ / day
	________________ Dose : _____ mg / body weight kg Frequency : ______ / day
	________________ Dose : _____ mg / body weight kg Frequency : ______ / day
	________________ Dose : _____ mg / body weight kg Frequency : ______ / day
	________________ Dose : _____ mg / body weight kg Frequency : ______ / day

**Please list all medicines you give to your child today by marking the circle based on the frequency and the time**					
________________	O ____ am/pm	O ____ am/pm	O ____ am/pm	O ____ am/pm	O ____ am/pm
________________	O ____ am/pm	O ____ am/pm	O ____ am/pm	O ____ am/pm	O ____ am/pm
________________	O ____ am/pm	O ____ am/pm	O ____ am/pm	O ____ am/pm	O ____ am/pm
________________	O ____ am/pm	O ____ am/pm	O ____ am/pm	O ____ am/pm	O ____ am/pm
________________	O ____ am/pm	O ____ am/pm	O ____ am/pm	O ____ am/pm	O ____ am/pm
________________	O ____ am/pm	O ____ am/pm	O ____ am/pm	O ____ am/pm	O ____ am/pm
________________	O ____ am/pm	O ____ am/pm	O ____ am/pm	O ____ am/pm	O ____ am/pm

Notes:

Registration ID | |

Day – 11 : |___|___| – |___|___| – 20 |___|___|

1. Please place a vertical line across the available horizontal line that best describes your or your child's pain during the past 12 hours? Please write the time accordingly ……. (am/ pm)

No Pain ———————————————— Pain As Bad As It Could Possibly Be

2. We are interest finding out how your child has been doing. For each question, please place a check mark in O corresponding to your child's symptoms. Please answer all questions. Please write the time accordingly ……. (am/ pm)

2.1 Over the past 12 h, has your child been tugging, rubbing, or holding the ear(s) more than usual?	O No	O A little	O A lot
2.2 Over the past 12 h, has your child been crying more than usual?	O No	O A little	O A lot
2.3 Over the past 12 h, has your child been more irritable or fussy than usual?	O No	O A little	O A lot
2.4 Over the past 12 h, has your child been having more difficulty sleeping than usual?	O No	O A little	O A lot
2.5 Over the past 12 h, has your child been less playful or active than usual?	O No	O A little	O A lot
2.6 Over the past 12 h, has your child been eating less than usual?	O No	O A little	O A lot
2.7 Over the past 12 h, has your child been having fever or feeling warm to touch?	O No	O A little	O A lot

Other symptoms

3 Does your child experience discharge from the ear(s)?	O Yes	O No
4 Does your child experience intense ear pain and pain behind the ear?	O Yes	O No
5 Does your child experience swelling/bulging, redness, tenderness, or dropping behind or of the ear(s)	O Yes	O No
6 Does your child experience facial asymmetry (e.g. when the child smiles, cries)?	O Yes	O No

7 Side effects

Does your child have these complaints after taking the medicine

7.1 Increased appetite	O Yes	O No	7.8 Drowsiness	O Yes	O No
7.2 Increased urine amount	O Yes	O No	7.9 Anxiety/distractibility/mood swing	O Yes	O No
7.3 Weight gain	O Yes	O No	7.10 Headache	O Yes	O No
7.4 Gastritis/abdominal pain	O Yes	O No	7.11 Skin rash or diaper rash	O Yes	O No
7.5 Nausea	O Yes	O No	7.12 Candidiasis	O Yes	O No
7.6 Vomiting	O Yes	O No	7.13 Dry mouth / throat irritation	O Yes	O No
7.7 Diarrhea	O Yes	O No	7.14 Sleep disturbance	O Yes	O No
Others	______				
Did you bring your child to doctor (clinic or outpatient)?	O Yes	O No	Reason:	______	
			Medicine prescribed:	______	
Has your child has been admitted to hospital?	O Yes	O No	Reason:	______	
			Medicine prescribed:	______	

For each question, please tick (✓) your answer on O or write you answer on ______

Registration ID

**Medicines given (please write the name, dose, and frequency)**	
Additional medicine from the chemist store or other (not precribed by your doctor)	______________ Dose : _____ mg / body weight kg Frequency : ______ / day
	______________ Dose : _____ mg / body weight kg Frequency : ______ / day
	______________ Dose : _____ mg / body weight kg Frequency : ______ / day
	______________ Dose : _____ mg / body weight kg Frequency : ______ / day
	______________ Dose : _____ mg / body weight kg Frequency : ______ / day

**Please list all medicines you give to your child today by marking the circle based on the frequency and the time**					
______________	O ____ am/pm	O ____ am/pm	O ____ am/pm	O ____ am/pm	O ____ am/pm
______________	O ____ am/pm	O ____ am/pm	O ____ am/pm	O ____ am/pm	O ____ am/pm
______________	O ____ am/pm	O ____ am/pm	O ____ am/pm	O ____ am/pm	O ____ am/pm
______________	O ____ am/pm	O ____ am/pm	O ____ am/pm	O ____ am/pm	O ____ am/pm
______________	O ____ am/pm	O ____ am/pm	O ____ am/pm	O ____ am/pm	O ____ am/pm
______________	O ____ am/pm	O ____ am/pm	O ____ am/pm	O ____ am/pm	O ____ am/pm
______________	O ____ am/pm	O ____ am/pm	O ____ am/pm	O ____ am/pm	O ____ am/pm

Notes:

Registration ID

Day – 12 : |___|___| – |___|___| – 20 |___|___|

1. Please place a vertical line across the available horizontal line that best describes your or your child's pain during the past 12 hours? Please write the time accordingly ……. (am/ pm)

No Pain |————————————| Pain As Bad As It Could Possibly Be

2. We are interest finding out how your child has been doing. For each question, please place a check mark in O corresponding to your child's symptoms. Please answer all questions. Please write the time accordingly ……. (am/ pm)

2.1 Over the past 12 h, has your child been tugging, rubbing, or holding the ear(s) more than usual?	O No	O A little	O A lot
2.2 Over the past 12 h, has your child been crying more than usual?	O No	O A little	O A lot
2.3 Over the past 12 h, has your child been more irritable or fussy than usual?	O No	O A little	O A lot
2.4 Over the past 12 h, has your child been having more difficulty sleeping than usual?	O No	O A little	O A lot
2.5 Over the past 12 h, has your child been less playful or active than usual?	O No	O A little	O A lot
2.6 Over the past 12 h, has your child been eating less than usual?	O No	O A little	O A lot
2.7 Over the past 12 h, has your child been having fever or feeling warm to touch?	O No	O A little	O A lot

Other symptoms

3 Does your child experience discharge from the ear(s)?	O Yes	O No
4 Does your child experience intense ear pain and pain behind the ear?	O Yes	O No
5 Does your child experience swelling/bulging, redness, tenderness, or dropping behind or of the ear(s)	O Yes	O No
6 Does your child experience facial asymmetry (e.g. when the child smiles, cries)?	O Yes	O No

7 Side effects

Does your child have these complaints after taking the medicine

7.1 Increased appetite	O Yes	O No	7.8 Drowsiness	O Yes	O No
7.2 Increased urine amount	O Yes	O No	7.9 Anxiety/distractibility/mood swing	O Yes	O No
7.3 Weight gain	O Yes	O No	7.10 Headache	O Yes	O No
7.4 Gastritis/abdominal pain	O Yes	O No	7.11 Skin rash or diaper rash	O Yes	O No
7.5 Nausea	O Yes	O No	7.12 Candidiasis	O Yes	O No
7.6 Vomiting	O Yes	O No	7.13 Dry mouth / throat irritation	O Yes	O No
7.7 Diarrhea	O Yes	O No	7.14 Sleep disturbance	O Yes	O No
Others	______				

Did you bring your child to doctor (clinic or outpatient)?	O Yes	O No	Reason:	______
			Medicine prescribed:	______
Has your child has been admitted to hospital?	O Yes	O No	Reason:	______
			Medicine prescribed:	______

For each question, please tick (✓) your answer on O or write you answer on ______

**Medicines given (please write the name, dose, and frequency)**			
Additional medicine from the chemist store or other (not precribed by your doctor)	____________________	Dose : _____ mg / body weight kg	Frequency : ______/ day
	____________________	Dose : _____ mg / body weight kg	Frequency : ______/ day
	____________________	Dose : _____ mg / body weight kg	Frequency : ______/ day
	____________________	Dose : _____ mg / body weight kg	Frequency : ______/ day
	____________________	Dose : _____ mg / body weight kg	Frequency : ______/ day

**Please list all medicines you give to your child today by marking the circle based on the frequency and the time**					
____________________	O ____ am/pm	O ____ am/pm	O ____ am/pm	O ____ am/pm	O ____ am/pm
____________________	O ____ am/pm	O ____ am/pm	O ____ am/pm	O ____ am/pm	O ____ am/pm
____________________	O ____ am/pm	O ____ am/pm	O ____ am/pm	O ____ am/pm	O ____ am/pm
____________________	O ____ am/pm	O ____ am/pm	O ____ am/pm	O ____ am/pm	O ____ am/pm
____________________	O ____ am/pm	O ____ am/pm	O ____ am/pm	O ____ am/pm	O ____ am/pm
____________________	O ____ am/pm	O ____ am/pm	O ____ am/pm	O ____ am/pm	O ____ am/pm
____________________	O ____ am/pm	O ____ am/pm	O ____ am/pm	O ____ am/pm	O ____ am/pm

Notes:

Registration ID | |

Day – 13 : |___|___| – |___|___| – 20 |___|___|

1. Please place a vertical line across the available horizontal line that best describes your or your child's pain during the past 12 hours? Please write the time accordingly ……. (am/ pm)

No Pain |————————————————| Pain As Bad As It Could Possibly Be

2. We are interest finding out how your child has been doing. For each question, please place a check mark in O corresponding to your child's symptoms. Please answer all questions. Please write the time accordingly ……. (am/ pm)

2.1 Over the past 12 h, has your child been tugging, rubbing, or holding the ear(s) more than usual?	O No	O A little	O A lot
2.2 Over the past 12 h, has your child been crying more than usual?	O No	O A little	O A lot
2.3 Over the past 12 h, has your child been more irritable or fussy than usual?	O No	O A little	O A lot
2.4 Over the past 12 h, has your child been having more difficulty sleeping than usual?	O No	O A little	O A lot
2.5 Over the past 12 h, has your child been less playful or active than usual?	O No	O A little	O A lot
2.6 Over the past 12 h, has your child been eating less than usual?	O No	O A little	O A lot
2.7 Over the past 12 h, has your child been having fever or feeling warm to touch?	O No	O A little	O A lot

Other symptoms

3 Does your child experience discharge from the ear(s)?	O Yes	O No
4 Does your child experience intense ear pain and pain behind the ear?	O Yes	O No
5 Does your child experience swelling/bulging, redness, tenderness, or dropping behind or of the ear(s)	O Yes	O No
6 Does your child experience facial asymmetry (e.g. when the child smiles, cries)?	O Yes	O No

7 Side effects

Does your child have these complaints after taking the medicine

7.1 Increased appetite	O Yes	O No	7.8 Drowsiness	O Yes	O No
7.2 Increased urine amount	O Yes	O No	7.9 Anxiety/distractibility/mood swing	O Yes	O No
7.3 Weight gain	O Yes	O No	7.10 Headache	O Yes	O No
7.4 Gastritis/abdominal pain	O Yes	O No	7.11 Skin rash or diaper rash	O Yes	O No
7.5 Nausea	O Yes	O No	7.12 Candidiasis	O Yes	O No
7.6 Vomiting	O Yes	O No	7.13 Dry mouth / throat irritation	O Yes	O No
7.7 Diarrhea	O Yes	O No	7.14 Sleep disturbance	O Yes	O No
Others	______				
Did you bring your child to doctor (clinic or outpatient)?	O Yes	O No	Reason:	______	
			Medicine prescribed:	______	
Has your child has been admitted to hospital?	O Yes	O No	Reason:	______	
			Medicine prescribed:	______	

For each question, please tick (✓) your answer on O or write you answer on ______

Registration ID | |

**Medicines given (please write the name, dose, and frequency)**			
Additional medicine from the chemist store or other (not precribed by your doctor)	________________	Dose : _____ mg / body weight kg	Frequency : ______ / day
	________________	Dose : _____ mg / body weight kg	Frequency : ______ / day
	________________	Dose : _____ mg / body weight kg	Frequency : ______ / day
	________________	Dose : _____ mg / body weight kg	Frequency : ______ / day
	________________	Dose : _____ mg / body weight kg	Frequency : ______ / day

**Please list all medicines you give to your child today by marking the circle based on the frequency and the time**					
________________	O ____ am/pm	O ____ am/pm	O ____ am/pm	O ____ am/pm	O ____ am/pm
________________	O ____ am/pm	O ____ am/pm	O ____ am/pm	O ____ am/pm	O ____ am/pm
________________	O ____ am/pm	O ____ am/pm	O ____ am/pm	O ____ am/pm	O ____ am/pm
________________	O ____ am/pm	O ____ am/pm	O ____ am/pm	O ____ am/pm	O ____ am/pm
________________	O ____ am/pm	O ____ am/pm	O ____ am/pm	O ____ am/pm	O ____ am/pm
________________	O ____ am/pm	O ____ am/pm	O ____ am/pm	O ____ am/pm	O ____ am/pm
________________	O ____ am/pm	O ____ am/pm	O ____ am/pm	O ____ am/pm	O ____ am/pm

Notes:

Registration ID

Day – 14 : |___|___| – |___|___| – 20 |___|___|

1. Please place a vertical line across the available horizontal line that best describes your or your child's pain during the past 12 hours? Please write the time accordingly ……. (am/ pm)

No Pain ———————————————— Pain As Bad As It Could Possibly Be

2. We are interest finding out how your child has been doing. For each question, please place a check mark in O corresponding to your child's symptoms. Please answer all questions. Please write the time accordingly ……. (am/ pm)

2.1 Over the past 12 h, has your child been tugging, rubbing, or holding the ear(s) more than usual?	O No	O A little	O A lot
2.2 Over the past 12 h, has your child been crying more than usual?	O No	O A little	O A lot
2.3 Over the past 12 h, has your child been more irritable or fussy than usual?	O No	O A little	O A lot
2.4 Over the past 12 h, has your child been having more difficulty sleeping than usual?	O No	O A little	O A lot
2.5 Over the past 12 h, has your child been less playful or active than usual?	O No	O A little	O A lot
2.6 Over the past 12 h, has your child been eating less than usual?	O No	O A little	O A lot
2.7 Over the past 12 h, has your child been having fever or feeling warm to touch?	O No	O A little	O A lot

Other symptoms

3 Does your child experience discharge from the ear(s)?	O Yes	O No
4 Does your child experience intense ear pain and pain behind the ear?	O Yes	O No
5 Does your child experience swelling/bulging, redness, tenderness, or dropping behind or of the ear(s)	O Yes	O No
6 Does your child experience facial asymmetry (e.g. when the child smiles, cries)?	O Yes	O No

7 Side effects

Does your child have these complaints after taking the medicine

7.1 Increased appetite	O Yes	O No	7.8 Drowsiness	O Yes	O No
7.2 Increased urine amount	O Yes	O No	7.9 Anxiety/distractibility/mood swing	O Yes	O No
7.3 Weight gain	O Yes	O No	7.10 Headache	O Yes	O No
7.4 Gastritis/abdominal pain	O Yes	O No	7.11 Skin rash or diaper rash	O Yes	O No
7.5 Nausea	O Yes	O No	7.12 Candidiasis	O Yes	O No
7.6 Vomiting	O Yes	O No	7.13 Dry mouth / throat irritation	O Yes	O No
7.7 Diarrhea	O Yes	O No	7.14 Sleep disturbance	O Yes	O No
Others	________				

Did you bring your child to doctor (clinic or outpatient)?	O Yes	O No	Reason:	________
			Medicine prescribed:	________
Has your child has been admitted to hospital?	O Yes	O No	Reason:	________
			Medicine prescribed:	________

For each question, please tick (✓) your answer on O or write you answer on ______

Registration ID

**Medicines given (please write the name, dose, and frequency)**			
Additional medicine from the chemist store or other (not precribed by your doctor)	______________________	Dose : _____ mg / body weight kg	Frequency : ______ / day
	______________________	Dose : _____ mg / body weight kg	Frequency : ______ / day
	______________________	Dose : _____ mg / body weight kg	Frequency : ______ / day
	______________________	Dose : _____ mg / body weight kg	Frequency : ______ / day
	______________________	Dose : _____ mg / body weight kg	Frequency : ______ / day

**Please list all medicines you give to your child today by marking the circle based on the frequency and the time**					
______________________	O ____ am/pm	O ____ am/pm	O ____ am/pm	O ____ am/pm	O ____ am/pm
______________________	O ____ am/pm	O ____ am/pm	O ____ am/pm	O ____ am/pm	O ____ am/pm
______________________	O ____ am/pm	O ____ am/pm	O ____ am/pm	O ____ am/pm	O ____ am/pm
______________________	O ____ am/pm	O ____ am/pm	O ____ am/pm	O ____ am/pm	O ____ am/pm
______________________	O ____ am/pm	O ____ am/pm	O ____ am/pm	O ____ am/pm	O ____ am/pm
______________________	O ____ am/pm	O ____ am/pm	O ____ am/pm	O ____ am/pm	O ____ am/pm
______________________	O ____ am/pm	O ____ am/pm	O ____ am/pm	O ____ am/pm	O ____ am/pm

Notes:

Thank you for completing the third Diary.

Date ____________________

CRF07. Prescription for OPAL study medication

Prednisolone doses:

- Aged 6 months to < 2 years old = 10 mg per day
- Aged 2 years to < 6 years old = 20 mg per day
- Aged 6 years to 12 years old = 30 mg per day

Registration ID : [ ]

Name : ____________________

Age : ______________ months / year(s) *[write and circle your answer]*

Study medication dose : ________ mg per day = ________ tablets per day

R/ OPAL study medication tablet ……..

Sach lact **add**

m.f. pulveres dtd **No. V**

ʃ 1 dd 1 pc (before 9 am)

____

(sign here)

REGISTRATION ID | | | | |

Nurse ID : |___|___|___| **Site ID : |___|___|___|** **Date : |___|___| – |___|___| – 201 |___|**

**CRF08 – RANDOMISATION FORM**		
**Eligibility criteria (cross-check with 'FORM01. study registration log book', and 'CRF03. Eligibility form' in the 'Case Report Form Binder' of this subject).**		
All YES for all inclusion criteria	**O** Yes	**O** No
All NO for all exclusion criteria	**O** Yes	**O** No
**Consent to the study questions (cross-check with 'CRF01. Informed consent' in the 'Case Report Form Binder' of this subject).**		
Has consent given?	**O** Yes	**O** No

**RANDOMISATION**								
**Father's mobile phone number**								
**Mother's mobile phone number**								
**Severity of AOM**	**O Mild AOM**					**O Severe AOM**		
**Subject's date of birth**	Date		Month		Year	**AGE**		Month/year

**RANDOMISATION RESULT**					
**Randomisation ID**					
**This subject is allocated to**	**O Prednisolone group**		**O Control group (no prednisolone)**		
**Prednisolone dosage (if the subject is allocated to prednisolone group)**	**O 10 mg/day**		**O 20 mg/day**		**O 30 mg/day**

Nurse's signature		Nurse's name		Date	

Follow-up Visit Card

OPAL Study
Oral Prednisolone for Acute otitis media in children

Name : ______________________________

Address : ______________________________

____

Dad/Mom's phone no : ______________________________

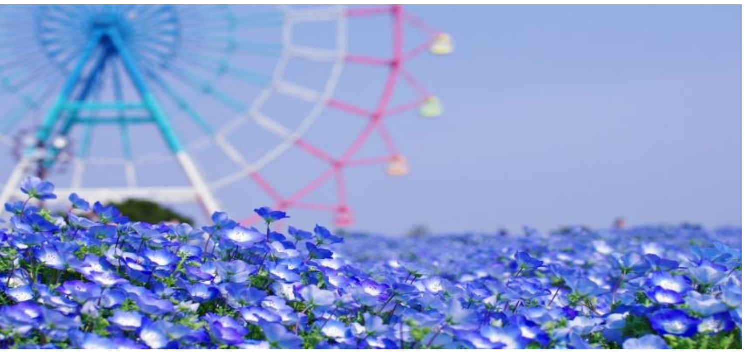

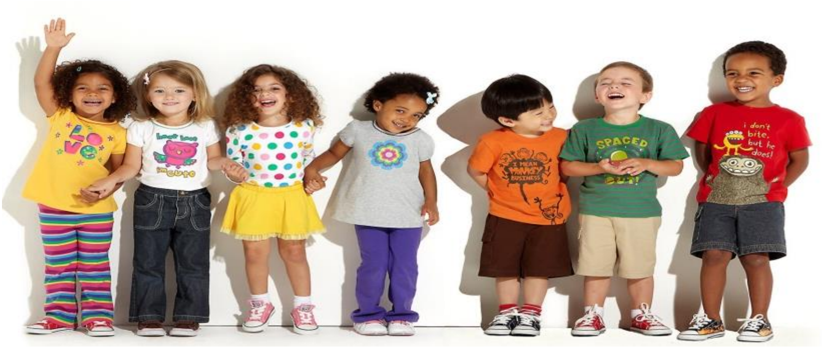

Clinical Epidemiology and Evidence-Based Medicine (CEEBM) Unit
Dr Cipto Mangunkusumo Hospital – Faculty of Medicine Universitas Indonesia
Centre for Research in Evidence-Based Practice
Faculty of Health Sciences and Medicine, Bond University, Australia

Follow-up Visit Schedule

	Initial visit date	Scheduled visit dates	Actual visit dates	Notes
Initial visit (Day–0)				
Visit – 1 (Day – 3)				
Visit – 2 (Day – 7)				
Visit – 3 (Month – 1)				
Visit – 4 (Month – 4)				

Please always bring this card to every your follow-up visit to the Hospital

Phone numbers of Hospitals and Call-centre OPAL Study

Dr. Cipto Mangunkusumo Hospital

Jl. Diponegoro No.71, Central Jakarta
Operator : 1500135

Persahabatan Hospital

Jl. Persahabatan Raya No.1, East Jakarta
Operator : 021 489 1708 Ext. 285
ENT Clinic : Ext. 230
Paediatric Clinic : Ext. 283
Emergency Instalation : Ext. 499

Gatot Soebroto Army Hospital

Jl. Dr Abdul Rahman Saleh No.24, Senen, Central Jakarta
Operator : 021 344 1008, 021 384 0702
ENT Clinic : Ext. 2057
Paediatric Clinic : Ext. 2535
Emergency Instalation : Ext. 2121

Jakarta Islamic Hospital Cempaka Putih

Jl. Cempaka Putih Tengah I No. 1, Central Jakarta
Operator : 021 425 0451, 021 428 01567 Ext. 0
Outpatient Registration : Ext. 2
Emergency Instalation : Ext. 1

Proklamasi ENT Hospital

Jl. Proklamasi No.43, Central Jakarta
Operator : 021 390 0002, 021 392 4891 Ext. 0, 101, 227, 229
ENT Clinic : Ext. 100, 236 244
Emergency Instalation : Ext. 235

Antam Medika Hospital Pulogadung

Jl. Raya Pemuda No. 1A, Pulogadung, East Jakarta
Operator : 021 806 14 888
ENT Clinic : Ext. 1027
Paediatric Clinic : Ext. 1019
Emergency Instalation : Ext. 1045

24-Call Centre OPAL Study

Dr. Respati W. Ranakusuma, Sp.THT-KL : 08111 012 185

REGISTRATION ID

CRF10. SERIOUS ADVERSE EVENTS REPORTING FORM

SUBJECT INFORMATION

Weight (kg)	|____|____| , |____|____| kg
List any relevant tests, laboratory data, history, including pre-existing medical conditions	____________
Any concomitant medication	____________

ADVERSE EVENT

Report type	☐ Initial report	☐ Follow-up	☐ Final
Reason for reporting	☐ Requires or prolongs hospitalization	☐ Congenital anomaly	
	☐ Permanently disabling or incapacitating	☐ Life threatening	
	☐ Overdose	☐ Death	
	☐ Other (please specify) ____________	Date of death ________	
		Cause of death ________	

SUSPECTED DRUG

Name of suspected drug	____________	Generic name	____________
Dose details	____________	Name of manufacturer	____________
Date of occurrence	|____|____| - |____|____| - |____|____|____|____| *(date – month – year)*		
Duration of event	|____|____| month(s) |____|____| day(s)		
Starting date of medication	|____|____| - |____|____| - |____|____|____|____| *(date – month – year)*		
Route of administration	____________	Indication	____________
Discontinuation of drug because of event	☐ No	☐ Yes	Dated (date / month / year) : ____________
If stopped/lowered dose, did the event resolve after this?	☐ Yes	☐ No	☐ N/A
If reintroduced did the event reappear?	☐ Yes	☐ No	☐ N/A
Outcomes	☐ Recovered	☐ Recovered with sequelae	☐ Continuing
	☐ Change in SAE	☐ Patient died	☐ Unknown
Severity	☐ Mild	☐ Moderate	☐ Severe
Action taken with study drug	☐ None	☐ Dose reduced	☐ Discontinued
	☐ Dose temporarily reduced	☐ Discontinued temporarily	
Other action*	☐ None	☐ Treated with medication	☐ Other

Withdrawn from the trial due to SAE	☐ No	☐ Yes
**REPORTER INFORMATION**		
Signature of reporter	______________________________	
Date of signing	|____|____| - |____|____| - |____|____|____|____| *(date – month – year)*	
Full name	______________________________	

Registration ID [ ]

CRF11 – FEEDBACK FORM (for Physician only)

<table>
<tr><th colspan="2">Questions</th><th colspan="5">Please place a checkmark (√) inthe box corresponding to your answer</th></tr>
<tr><td rowspan="2">Information Sheet and Consent Form</td><td>How do you rate the process of providing patient information and informed consent to your patient?</td><td>☐ Very easy</td><td>☐ Easy</td><td>☐ Neutral</td><td>☐ Difficult</td><td>☐ Very difficult</td></tr>
<tr><td>If your answer 'difficult' or 'very difficult', please place a checkmark (V) in the box corresponding to or write you reason(s). You may choose more than one</td><td colspan="5">☐ It was too difficult to explain this to my patient/parent
☐ Time consuming
☐ There was too much information to explain
☐ I was not sure that my patient understood
☐ Others : ____________________
____
____________________</td></tr>
<tr><td rowspan="2">Otosocopic Examination</td><td>How do rate the process of conducting an otoscopic examination to your patient?</td><td>☐ Very easy</td><td>☐ Easy</td><td>☐ Neutral</td><td>☐ Difficult</td><td>☐ Very difficult</td></tr>
<tr><td>If your answer 'difficult' or 'very difficult', please place a checkmark (V) in the box corresponding to or write you reason(s). You may choose more than one</td><td colspan="5">☐ Patient was not cooperative
☐ The ear canal was too narrow
☐ Insufficient tool (e.g. the otoscope cylidner was too large)
☐ Ear wax and it was too difficult to extract
☐ The symptoms are definitely celar showing AOM
☐ Others : ____________________
____
____________________</td></tr>
<tr><td rowspan="2">Visual Analogue Scale (VAS)</td><td>How do you rate the process of providing related information and assisting your patient/parent to complete the visual analogue scale (VAS)?</td><td>☐ Very easy</td><td>☐ Easy</td><td>☐ Neutral</td><td>☐ Difficult</td><td>☐ Very difficult</td></tr>
<tr><td>If your answer 'difficult' or 'very difficult', please place a checkmark (V) in the box corresponding to or write you reason(s). You may choose more than one</td><td colspan="5">☐ It was too difficult to explain this to my patient/parent
☐ Time consuming
☐ I was not sure that my patient/parent understood
☐ My patient/parent seem not confidence with the answer
☐ Others : ____________________
____
____________________</td></tr>
<tr><td>Acute Otitis Media –</td><td>How do you rate the process of providing related information and assisting your patient/parent to complete the acute otitis</td><td>☐ Very easy</td><td>☐ Easy</td><td>☐ Neutral</td><td>☐ Difficult</td><td>☐ Very difficult</td></tr>
</table>

<table>
<tr><td rowspan="2"></td><td>media – severity of symptom scale (AOM–SOS)?</td><td></td><td></td><td></td><td></td><td></td></tr>
<tr><td>If your answer ‘difficult’ or ‘very difficult’, please place a checkmark (V) in the box corresponding to or write you reason(s). You may choose more than one</td><td colspan="5">☐ It was too difficult to explain this to my patient/parent
☐ Time consuming
☐ I was not sure that my patient/parent understood
☐ There were several questions that difficult to explain or not suitable for my patient/parent: question no. ___ ; ___ ; ___ ;
☐ Others : ____________________</td></tr>
<tr><td rowspan="2">Symptom Diary</td><td>How do you rate the process of providing related information and assisting your patient/parent to complete the Patient/parent Diary?</td><td>☐ Very easy</td><td>☐ Easy</td><td>☐ Neutral</td><td>☐ Difficult</td><td>☐ Very difficult</td></tr>
<tr><td>If your answer ‘difficult’ or ‘very difficult’, please place a checkmark (V) in the box corresponding to or write you reason(s). You may choose more than one</td><td colspan="5">☐ It was too difficult to explain this to my patient/parent
☐ Time consuming
☐ I was not sure that my patient/parent understood
☐ The sequence of the questions was too confusing
☐ Questions in the symptom diary are too many
☐ Others : ____________________</td></tr>
<tr><td rowspan="2">Case Report Forms</td><td>How do you rate the process in completing the case report forms (CRFs)?</td><td>☐ Very easy</td><td>☐ Easy</td><td>☐ Neutral</td><td>☐ Difficult</td><td>☐ Very difficult</td></tr>
<tr><td>If your answer ‘difficult’ or ‘very difficult’, please place a checkmark (V) in the box corresponding to or write you reason(s). You may choose more than one</td><td colspan="5">☐ Time consuming
☐ Too much unnecessary information was required
☐ The sequence of the questions was too confusing
☐ Several questions in the CRF were difficult to understand
☐ Others : ____________________</td></tr>
<tr><td rowspan="2">Screening and Stratification Process</td><td>How do you rate the recruitment process, particularly in classifying the children based on their eligibility and stratification process to mild or severe AOM groups?</td><td>☐ Very easy</td><td>☐ Easy</td><td>☐ Neutral</td><td>☐ Difficult</td><td>☐ Very difficult</td></tr>
<tr><td>If your answer ‘difficult’ or ‘very difficult’, please place a checkmark (V) in the box corresponding to or write</td><td colspan="5">☐ The CRF03. Eligibility form is too complicated
☐ The form was not helping me to screen and stratify my patient/ parent
☐ Despite I was guided by the form, I was still found the process was still confusing, particularly in terms of deciding</td></tr>
</table>

	you reason(s). You may choose more than one	which group my patient/parent should go to (i.e. mild vs severe acute otitis media)<br>☐ Others : ______________________________<br>______________________________<br>______________________________

Registration ID

FEEDBACK FORM (for Nurses who conducts randomisation only)

<table>
<tr><th colspan="2">Questions</th><th colspan="5">Please place a checkmark (√) inthe box corresponding to your answer</th></tr>
<tr><td rowspan="2">Randomisation Process</td><td>How do you rate the randomisation process, in terms of obtaining the study ID and the allocation of the intervention (prednisolone group or control group)</td><td>☐ Very easy</td><td>☐ Easy</td><td>☐ Neutral</td><td>☐ Difficult</td><td>☐ Very difficult</td></tr>
<tr><td>If your answer ‘difficult’ or ‘very difficult’, please place a checkmark (V) in the box corresponding to or write you reason(s). You may choose more than one.</td><td colspan="5">☐ The CRF08. Randomisation form is too complicated
☐ The randomisation process was too confusing
☐ It was difficult to access the randomisation centre (randomisation website or by phone) to obtain the study ID and the allocation of the intervention
☐ It was difficult to explain to the patients that they were allocated to groups which reveice prednisolone or not
☐ Others ____________________
____
____________________</td></tr>
<tr><td rowspan="2">Dispensing the Study Medication Prescription</td><td>How do rate the process of dispensing the study medication prescription and keep the intervention allocation concealed from their Physician and Audiologists?</td><td>☐ Very easy</td><td>☐ Easy</td><td>☐ Neutral</td><td>☐ Difficult</td><td>☐ Very difficult</td></tr>
<tr><td>If your answer ‘difficult’ or ‘very difficult’, please place a checkmark (V) in the box corresponding to or write you reason(s). You may choose more than one</td><td colspan="5">☐ This process was too time consuming
☐ I encountered difficulties when I was providing relevant information on the intervention they received
☐ It was difficult to ask my patients/parents to keep the information of intervention allocation confidential
☐ Others: ____________________
____
____________________</td></tr>
<tr><td rowspan="2">The compilation and the Storage of Case report Forms</td><td>How do rate the process of the compilation and the storage of study documents and binders?</td><td>☐ Very easy</td><td>☐ Easy</td><td>☐ Neutral</td><td>☐ Difficult</td><td>☐ Very difficult</td></tr>
<tr><td>If your answer ‘difficult’ or ‘very difficult’, please place a checkmark (V) in the box corresponding to or write you reason(s). You may choose more than one</td><td colspan="5">☐ This process was too confusing
☐ This process was too time consuming
☐ It was difficult to find case report forms in the binder
☐ The checklist of case report forms was not helping
☐ Others: ____________________
____
____________________</td></tr>
</table>

Registration ID ☐

FEEDBACK FORM (for Audiologist/Trained Staff only)

<table>
<tr><th colspan="2">Questions</th><th colspan="5">Please place a checkmark (√) inthe box corresponding to your answer</th></tr>
<tr><td rowspan="2">Tympanomtry Examination and the Competion of Tympanometry Section in the Case report Form</td><td>How do you rate the process of tympanometry examination and completing the tympanometry section in CRF?</td><td>☐ Very easy</td><td>☐ Easy</td><td>☐ Neutral</td><td>☐ Difficult</td><td>☐ Very difficult</td></tr>
<tr><td>If your answer ‘difficult’ or ‘very difficult’, please place a checkmark (V) in the box corresponding to or write you reason(s). You may choose more than one.</td><td colspan="5">☐ The patients’ parents seem did not understand teh reason this examination being performed
☐ It was difficult to conduct this examination to my patients
☐ The ‘Tympanometry section’ in CRF05. Outcome form is confusing. The provided examination components are unfamiliar or different
☐ It was difficult to find the ‘Tympanometry section’ in the CRF05. Outcome form
☐ It was difficult to print out the copy of tympanometry result
☐ There were few components of this examination not provided in the form
☐ Others: ____________________
____
____________________</td></tr>
</table>

Registration ID | |

FEEDBACK FORM (for Pharmacists only)

	Questions	Please place a checkmark (√) inthe box corresponding to your answer				
**Preparation and Dispensing teh Study Medication**	How do rate **the preparation and dispensing process of the study medication**?	☐ Very easy	☐ Easy	☐ Neutral	☐ Difficult	☐ Very difficult
	If your answer 'difficult' or 'very difficult', please place a checkmark (V) in the box corresponding to or write you reason(s). You may choose more than one.	☐ The instruction in CRF07. Prescription was confusing<br>☐ The preparation of the study medication was too time-consuming<br>☐ I encountered difficulties when providing the information about the study medication to my patients<br>☐ Others: ________________________________<br>________________________________<br>________________________________				

Registration ID

FEEDBACK FORM (for Parents only)

Questions	Please place a checkmark (√) inthe box corresponding to your answer
How do you rate the process in **completing the pain scale** below?	

No Pain — Pain As Bad As It Could Possibly Be

	☐ Very easy	☐ Easy	☐ Neutral	☐ Difficult	☐ Very difficult

If your answer 'difficult' or 'very difficult', please place a checkmark (V) in the box corresponding to or write you reason(s). You may choose more than one.	☐ I did not understand how to complte this scale<br>☐ I need more information from my doctor<br>☐ The provided instruction in the form was unclear<br>☐ My doctor could not provide additional information that I need<br>☐ Others : ____________________<br>____________________<br>____________________

How do you rate the process in **completing the AOM-relevant symptom questionnaire** below?

12 We are interest finding out how your child has been doing. For each question, please place a checkmark (V) in the circle corresponding to your child's symptoms. Please answer all questions.			
12.1 Over the past 12 h, has your child been tugging, rubbing, or holding the ear(s) more than usual?	O No	O A little	O A lot
12.2 Over the past 12 h, has your child been crying more than usual?	O No	O A little	O A lot
12.3 Over the past 12 h, has your child been more irritable or fussy than usual?	O No	O A little	O A lot
12.4 Over the past 12 h, has your child been having more difficulty sleeping than usual?	O No	O A little	O A lot
12.5 Over the past 12 h, has your child been less playful or active than usual?	O No	O A little	O A lot
12.6 Over the past 12 h, has your child been eating less than usual?	O No	O A little	O A lot
12.7 Over the past 12 h, has your child been having fever or feeling warm to touch?	O No	O A little	O A lot

	☐ Very easy	☐ Easy	☐ Neutral	☐ Difficult	☐ Very difficult

Apabila jawaban Anda 'Difficult' atau 'Very difficult', mohon berikan tanda centang di kotak yang sesuai atau berikan alasan Anda. Anda dipersilahkan untuk memilih lebih dari satu jawaban.	☐ It was difficult to understand the question(s)<br>☐ The options of answers were confusing<br>☐ The provided instruction in the form was unclear<br>☐ The question(s) was not suitable for my child, therefore I did not know how to answer the question(s): question no ____ ; ____ : ____<br>☐ I do not know how to complete the questionnaire

	☐ Others : ____________________ ____________________ ____________________				
How do you rate the process in completing **the overall symptom diary**?	☐ Very easy	☐ Easy	☐ Neutral	☐ Difficult	☐ Very difficult
If your answer 'difficult' or 'very difficult', please place a checkmark (V) in the box corresponding to or write you reason(s). You may choose more than one.	☐ I did not understand how to complete this diary<br>☐ I need more information from my doctor in how to complete this diary<br>☐ Instuctions provided in the diary are unclear<br>☐ Time consuming<br>☐ Too many questions that I did not think they were relevant with my child's condition<br>☐ The sequence of the questions was too confusing<br>☐ Others : ____________________ ____________________ ____________________				

dr. Respati W. Ranakusuma, SpTHT-KL
Clinical Epidemiology & Evidence-Based Medicine (CEEBM) Unit Dr. Cipto Mangunkusumo Hospital – Faculty of Medicine Universitas Indonesia

FORM01 – STUDY RECRUITMENT LOG BOOK

**Nurse name/ID :**	**Study title :** Oral prednisolone for acute otitis media in children: a pilot, pragmatic, randomised, open-label, single-blind, controlled study (OPAL study)	**Hospital ID :**

Study registration ID	Patient's name	Date screened	Has your child experiencing ear pain in the past 48 hours? (YES or NO)	Has your child been tugging or rubbing her/his ear(s) and been more irritable or fussy or crying more than usual over the past 48 hours (YES or NO)	Has your child been experiencing ear discharge in the past 48 hours? (YES or NO)	Body weight (kg)	Body height (cm)	Body temperature (°C)	Blood pressure (mmHg)	Did patient go on the study? (YES or NO)	If YES, what is the Randomisation ID	If NO, please tell us reason not on the study below		
												Not eligible (YES or NO)	Did not give consent (YES or NO)	Was not approached (YES or NO). Write the reason

Please write 'Y' for 'YES' and 'N' for 'NO'.

Pharmacist ID : |___|___|___|___| **Hospital ID** : |___|___|___|

FORM02 – STUDY MEDICATION STOCK FORM

Date received	No. of tablets	Batch number	Name who received the medication	Signature	Date checked	No. of tablets	Batch number	Name who checked the medication	Signature	Notes

Pharmacy ID : |___|___|___|___| **Hospital ID** : |___|___|___|

FORM03 – STUDY MEDICATION DISPENSING FORM (FOR PHARMACY)				
**Randomisation/ Registration ID**	**Date dispensed**	**Dose (mg/day)**	**Number of doses (day)**	**Initial dose (5 days) or additional dose**

Nurse ID : |___|___|___|___|

Hospital ID : |___|___|___|

FORM04 – STUDY MEDICATION RETURN FORM (FOR NURSE)

Randomisation/ Registration ID	Date returned	Number of left-over drug	Reason for return

FORM05 – COMPLETED CASE REPORT FORM (FOR NURSE WHO PERFORM A RANDOMISATION)							
Nurse ID :		Protocol title: Oral prednisolone for acute otitis media in children: a pilot pragmatic, randomised, open-label single-blind study (OPAL study)				Hospital ID :	
No	Randomisation ID	Date enrolled to the study	Date of Visit-1* (Day-3)	Date of Visit-2* (Day-7)	Date of Visit-3* (Day-30)	Date of Visit-4* (Day-90)	Date of completion of the study
			**Please write the checkmark (V) if the study participant come to each visit*				

FORM06 - RECAPITULATION OF NON-PARTICIPATING SUBJECT FORM (FOR NURSE WHO PERFORM A RANDOMISATION)						
Nurse ID :		Protocol title: Oral prednisolone for acute otitis media in children: a pilot pragmatic, randomised, open-label single-blind study (OPAL study)				Hospital ID :
No	Registration ID	Date subject offered into the study	Date subject not participate in the study	Reasons<br>*Please write the checkmark (V) if the study participant come to each visit*		
				Not eligible	Refuse to participate in the study	Others

FORM07. GUIDELINE OF ANTIBIOTICS FOR ACUTE OTITIS MEDIA

Initial immediate or delayed antibiotic therapy		Antibiotics after 48-72 hours of failure of initial antibiotic therapy	
**Recommended first-line treatment**	**Alternative treatment (if penicillin allergy)**	**Recommended first-line treatment**	**Alternative treatment**
Amoxicillin (80-90 mg/kg per day in 2 divided doses)<br><br>OR<br><br>Amoxicillin-clavulanate[a] (90 mg/kg per day of amoxicillin, with 6.4 mg/kg per day clavulanate (amoxicillin to clavulanate ration, 14:1) in 2 divided doses)	Cefdinir (14 mg/kg per day in 1 or 2 doses)<br><br>Cefuroxime (30 mg/kg per day in 2 divided doses)<br><br>Cefpodoxime (10 mg/kg per day in 2 divided doses)<br><br>Ceftriaxone (50 mg IM or IV per day for 1 or 3 days)	Amoxicillin-clavulanate[a] (90 mg/kg per day of amoxicillin, with 6.4 mg/kg per day in 2 divided doses)<br><br>OR<br><br>Ceftriaxone (50 mg IM or IV per day for 3 days)	Ceftriaxone, 3 days Clindamycin (30-40 mg/kg per day in 3 divided doses), with or without third-generation cephalosporin (50 mg IM or IV per day for 3 days)<br>Failure of second antibiotic<br>Clindamycin (30-40 mg/kg per day in 3 divided doses) plus third-generation cephalosporin<br>Tympanocentesis[b]<br>Consult specialist[b]

[a] *may be considered in patients who have received amoxicillin in the previous 3 0 days or who have the otitis conjunctivitis syndrome;*
[b] *Perform tympanocentesis/drainage if skilled in the procedure, or seek a consultation from an otolaryngologist for tympanocentesis/drainage if the tympanocentesis reveals multidrug/resistant bacteria, seek an infection disease specialist consultation.*

Reference: Lieberthal AS, Carroll AE, Chonmaitree T, et al. Clinical Practice Guideline: The diagnosis and management of acute otitis media. The American Academy of Pediatrics. *Pediatrics.* 2013;131:e964-e99

dr. Respati W. Ranakusuma, SpTHT-KL
Clinical Epidemiology & Evidence-Based Medicine Unit, Dr. Cipto Mangunkusumo Hospital – Faculty of Medicine Universitas Indonesia
Oral Prednisolone for acute otitis media in children: a pilot pragmatic, randomised, open-label, single-blind, controlled study (OPAL Study)

FORM08 – PREDNISOLONE DOSE FOR OPAL STUDY

Aged six months to up to two years old →	Dose : 10 mg / day (2 tablets per day) →	**10 tablets** of Prednisolone for five days
Aged two years to up to six years old →	Dose : 20 mg / day (4 tablets per day) →	**20 tablets** of Prednisolone for five days
Aged six years to 12 years old →	Dose : 30 mg / day (6 tablets per day) →	**30 tablets** of Prednisolone for five days

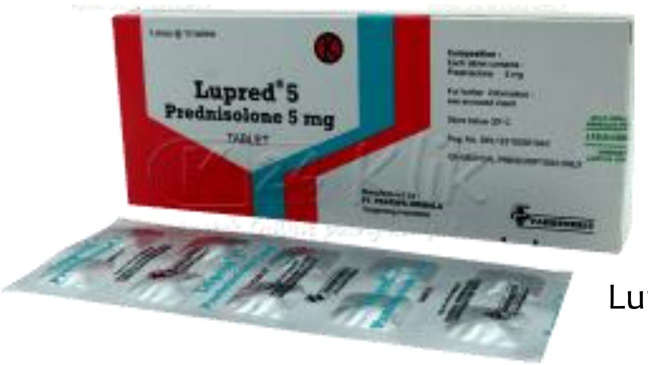

Lupred® 5 contains 5 mg prednisolone in each tablet

Instruction for using Prednisolone

We copied cited and copied the information on the leaflet from:
Medicine for children – information for parents and carers: prednisolone for asthma.
http://www.medicinesforchildren.org.uk/prednisolone-asthma

This leaflet has been written for parents and carers about how to use this medication in children. This information may differ from that provided by the pharmaceutical company, because their information is usually aimed at adult patients. Please read this leaflet carefully.

Name of drug

Lupred tablet contains of prednisolone.

When should I give prednisolone?

Prednisolone is usually given **once** each day, usually in the morning. Give the medicine at about the same time each day so that this becomes part of your child's daily routine, which will help you to remember.

How much should I give?

Your doctor will work out the amount (the dose) that is right for your child. It is important that you follow your doctor's instructions about how much to give.

How should I give it?

The pharmacist will prepare the prednisolone tablets by crushing the tablets, mixing it with the sweetener, and packing them in a daily paper-pack for your child.

You can mix it with a small amount of soft food such as yogurt, honey, or jam, or give a glass of milk or juice. Make sure your child swallows it straight away, without chewing.

When should the medicine start working?

Prednisolone usually takes 4–6 hours to have its full effect.

What if my child is sick (vomits)?

If your child is sick less than 30 minutes after having a dose of prednisolone, give them the same dose again.

If your child is sick more than 30 minutes after having a dose of prednisolone, you do not need to give them another dose. Wait until the next normal dose.

If your child is sick again, please contact us.

What if I forget to give it?

You can give your child the missed dose as soon as you remember on the same day. If you remember after they have gone to bed, do not give them the missed dose. Give the next dose in the morning as usual. Never give a double dose of prednisolone

What if I give too much?

It can be dangerous to give too much prednisolone. If you think you may have given your child too much prednisolone, contact us immediately.

Are there any possible side-effects?

We use medicines to make our children better, but sometimes they have other effects that we don't want (side-effects). It is unlikely that your child will have side-effects if they only take prednisolone for a few days. They are more likely to get side-effects if they are on a high dose, have extra doses or take prednisolone for a long time.

Side effects that you must do something about

- If your child has bad stomach pain or repeated vomiting (being sick), contact us straight away. This may be due to an ulcer or inflammation of the pancreas
- If your child develops a rash or severe/unexplained bruising, contact us straight away, as there may be a problem with your child's blood
- If your child has eye pain or changes in their vision, contact us straight away

Other side effects you need to know about

- child may have stomach ache, feel sick or be sick (vomit) or may have indigestion (heartburn). Giving the medicine with some food may help
- Your child may have an increased appetite and may gain weight while taking prednisolone. You can help by making sure your child has plenty of physical activity, and by offering fruit and vegetables and low-calorie food, rather than food that is high in calories (e.g. cakes, biscuits, sweets)
- Your child may have trouble sleeping and nightmares and may feel depressed, or their behaviour may change in other ways. Contact us for advice if you are concerned

Side effects with high doses or long courses

- Prednisolone can slow growth and affect puberty. It can also cause growth of body hair and irregular periods in girls
- Your child may be more at risk of severe infections. They should stay away from anyone with an infection (such as chicken pox, shingles, measles) if they have not had these illnesses or have not been vaccinated for measles
- If your child is unwell and you are worried about an infection, contact us straight away
- Your child's skin may become thinner, and heal more slowly than usual. Acne (spots) may become worse or your child may develop mouth ulcers or thrush (candidiasis). If you are concerned, contact us
- Your child may develop problems with their hip bones or their bones may become weaker (osteoporosis). The muscles around the hips and shoulders may also become weaker. If your child has any difficulty walking or moving around, contact us
- Occasionally, prednisolone causes diabetes. If your child seems more thirsty than normal, needs to pass urine (wee) often, or starts wetting the bed at night, contact us

There may, sometimes, be other side-effects that are not listed. above. If you notice anything unusual and are concerned, please contact us.

Can other medicines be given at the same time?

You can give your child medicines that contain paracetamol or ibuprofen, unless your doctor has told you not to. Check with us or your doctor before giving any other medicines to your child. This includes herbal or complimentary medicines.

Is there anything else I need to know about prednisolone?

For children who have been taking prednisolone in high doses or for longer than 2-3 weeks

- They must not stop taking the medicine suddenly because they may get withdrawal symptoms: they will feel unwell, dizzy and thirsty and may be sick (vomit). If this occurs, you should contact us straight away
- If your doctor decides to stop prednisolone, they will reduce the dose gradually before stopping it completely. Make sure you follow your doctor's instructions
- Make sure that you always have enough medicine.

Where should I keep this medicine?

- Keep the medicine in a cupboard, away from heat and direct sunlight. It does not need to be kept in the fridge
- Make sure that children cannot see or reach it.
- Keep the medicine in the container it came in

WHO TO CONTACT FOR MORE INFORMATION

OPAL STUDY 24-HOUR CALL CENTRE

08111 012 185

Lupred ® 5

Prednisolone 5 mg

TABLET

COMPOSITION
Each tablet contains:
Prednisolone 5 mg

PHARMACOLOGY
Prednisolone is a systemic corticosteroid with glucocorticoid and anti-inflammatory potencies. The mechanism of action of corticosteroids is thought to be by control of protein synthesis. Corticosteroids react with receptor proteins in the cytoplasm of sensitive cells in many tissues to form a steroid-receptor complex.

INDICATION
Allergic reaction, inflammation and other diseases that require glucocorticoid treatment, such as rheumatoid arthritis, collagen diseases, and dermatology disorders.

DOSAGE AND INSTRUCTION
Adults: 1 – 4 tablets per day or according to the doctor's instruction.
The dosage reduces gradually until reach the lowest effective dose.

PRECAUTION
- Avoid the abrupt discontinuation in a long-term use
- Use with caution in paediatric patients who are still in the growing process
- Not recommended for pregnant and breast-feeding women
- Prolonged use of corticosteroids may produce posterior subcapsular cataracts, glaucoma with possible damage to the optic nerves, and may enhance the establishment of secondary ocular infections due to fungi or viruses
- Risk of secondary adrenocortical insufficiency could be reduced by gradual reduction of dosage
- Use with caution in patients with diabetes mellitus because it can increase the gluconeogenesis and reduce the sensitivity to insulin
- Use with caution in patients with hypothyroidism because it can enhance the effect of corticosteroids
- Use with caution in patients with heart failure, infection diseases, chronic renal failure, and elderly

ADVERSE EFFECTS
- Water balance and electrolytes disturbance: Natrium retention, excretion of potassium, hypokalaemic alkalosis, hypertension, and congestive heart failure
- Musculoskeletal: Muscle weakness, steroid-induced myopathy, osteoporosis, vertebral compression fractures and pathologic fractures of long bones
- Gastrointestinal: Peptic ulceration with haemorrhage and perforation, pancreatitis, abdominal distension and ulcerative esophagitis
- Dermatological: Impaired wound healing, thinning of the skin, facial plethora, increased sweating
- Neurological: seizures, intracranial hypertension with papilloedema (cerebral pseudotumour), vertigo, headache

FAHRENHEIT
PT. PRATAPA NIRMALA

I001404-PG-1043

- Endocrine: Disorders of menstruation, suppression of growth in children, secondary adrenocorticoid and non-responsive pituitary (particularly in stress, trauma, surgery or illness), metabolic effects, primarily involving the carbohydrates
- Ophthalmological: Posterior subcapsular cataracts, increased intraocular pressure, glaucoma, and exophthalmos
- Metabolic: Nitrogen depletion due to protein catabolism
- Hypersensitivity: anaphylactic reaction

CONTRAINDICATION

- Patients who are known hypersensitivity to prednisone or prednisolone
- Peptic ulceration, active tuberculosis, osteoporosis, neurological disorders, renal and heart disorders
- Systemic fungal infections and ocular herpes simplex

INTERACTION WITH OTHER MEDICINES

- The use of aspirin and corticosteroid is not recommended in patients with non-specific ulcerative colitis
- Rifampicin, phenytoin, phenobarbital can increase the metabolism of corticosteroids
- Vaccination with live vaccine must be avoided

OVERDOSAGE

There is no specific antidot. Treatment is symptomatic with the dosage being reduced or the drug withdrawn.

STORAGE CONDITION

Store below 30°C.

DOCTOR's PRESCRIPTION IS A MUST

Manufactured by:
PT. PRATAPA NIRMALA
Tangerang – Indonesia

FAHRENHEIT
PT. PRATAPA NIRMALA

I001404-PG-1043
