# Supplementary material for: Oral prednisolone for acute otitis media in children: protocol of a pilot randomised, open-label, controlled study (OPAL study)
Source: Pilot Feasibility Stud. 2018 Sep 10;4:146. doi: 10.1186/s40814-018-0337-x (PMC6130070; doi:10.1186/s40814-018-0337-x)
Supplement: Supplementary file 3 — Manual of operations—Pilot OPAL Study. This file includes step-by-step manual for physicians, audiologists, nurses, and pharmacists who participate in the study. The manual of operations handbook was distributed during the trainings for participating physicians, audiologists, nurses, and pharmacists. This file can be accessed at https://pure.bond.edu.au/ws/portalfiles/portal/27513686/Additional_File_3._Manual_of_Operations_Pilot_OPAL_Study.pdf (PDF 7905 kb) [file 40814_2018_337_MOESM3_ESM.pdf]

# MANUAL OF OPERATIONS

**O**ral **P**rednisolone for **A**cute otitis media in chi**L**dren: a pilot pragmatic, randomised, open-label, single-blind study (**OPAL study**)

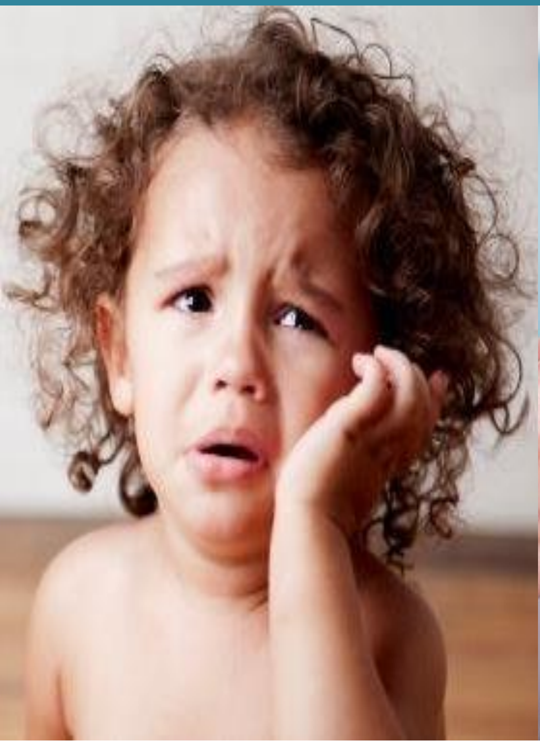
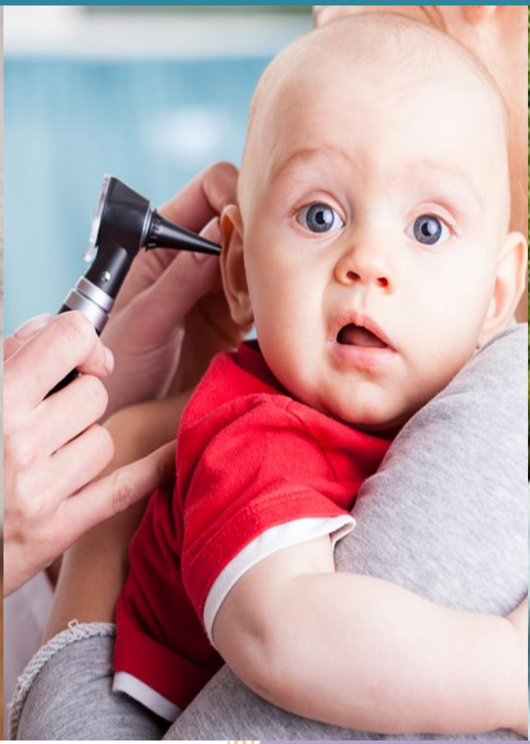
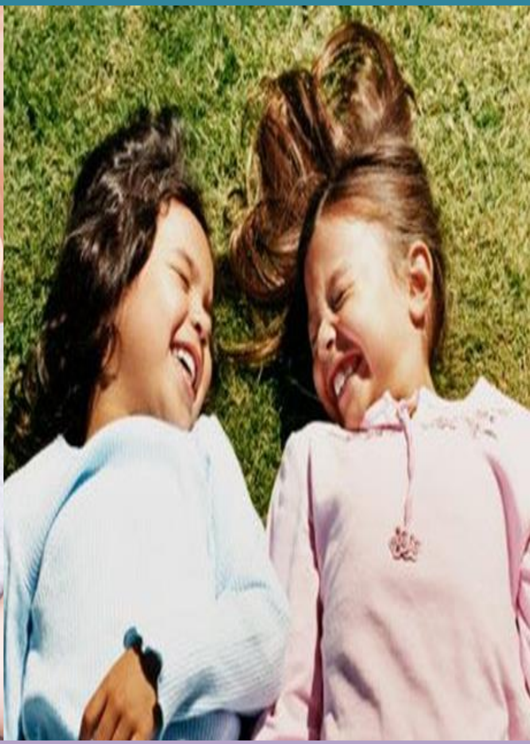

## November 2017


**Clinical Epidemiology & Evidence-Based Medicine Unit**
Dr. Cipto Mangunkusumo Hospital
Faculty of Medicine Universitas Indonesia

**Centre for Research in Evidence-Based Practice**
Faculty of Health Sciences & Medicine
Bond University, Queensland, Australia

# MANUAL OF OPERATIONS

## ORAL PREDNISOLONE FOR ACUTE OTITIS MEDIA IN CHILDREN:

## A PILOT PRAGMATIC, RANDOMISED, OPEN-LABEL, SINGLE-BLIND STUDY

## (OPAL STUDY)

# Table of Contents

Research summary ........ 3
The protocol ........ 5
Case report forms (CRFs) ........ 6
Setting up your site ........ 7
Study medication delivery and storage ........ 8
Rooms and other equipment for the study ........ 8
Step-by-step procedure ........ 11
The Nurse Station ........ 11
The Consultation Room ........ 12
Study consent, recruitment, and stratification ........ 12
Collecting baseline data and examination ........ 15
Tympanometry test ........ 19
Randomisation ........ 20
Preparing and Dispensing the study medication ........ 28
Returning the study medication ........ 29
Follow-up visits ........ 30
First Follow-up Visit (Day–3) ........ 30
Second Follow-up Visit (Day–7) ........ 31
Third Follow-up Visit (Day–30) ........ 32
Fourth Follow-up Visit (Day–90) ........ 32
Assessing adverse events ........ 34
Feedback fom ........ 35
Closing out the study ........ 36

# Research Summary

Acute otitis media (AOM) is an inflammation of the middle ear commonly found in children with symptoms of rapid onset (less than 48 hours) of ear pain, acute inflammation, and middle ear effusion (e.g. air fluid level, bulging) [1]. AOM is often self-limiting. In general, 60% of AOM cases will have clinical symptom improvement in the first 24 hours and 80% of cases in 72 hours without antibiotic treatment [2,3]. However, antibiotics are frequently prescribed regardless of the fact they do not relieve pain symptoms in most cases [3-5]. We conducted a survey study using clinical scenarios that demonstrated up to 88% of 352 physicians in Jakarta, Depok, and Bekasi (Indonesia) would prescribe antibiotics for mild AOM. However evidence shows that only one-third of cases benefit from antibiotics, generally the more severe, such as AOM with moderate-to-severe local or systemic symptoms, young children (< 2 years) with bilateral AOM, and AOM with tympanic membrane perforation [6]. In addition, frequent use of antibiotics leads to increased risk of unfavourable side effects (e.g. diarrhea, vomiting, rash) and antibiotic resistance, a serious threat to health globally [3,7].

Proposed alternatives or additions to antibiotics include various herbal preparations, decongestants, and corticosteroids [8,9]. However, the evidence for these is too weak to recommend for clinical practice. The anti-inflammatory effect of corticosteroids suggests it could be a viable treatment alternative for AOM. Our survey study demonstrated that 44% of ENT specialists would prescribe corticosteroids for children with AOM. We also conducted a systematic review of randomised placebo-controlled trials (RCTs) of steroids for AOM. Although only two small trials [10-12], indicated corticosteroids could be useful in this condition, our confidence in the results is low, due to small sample size and very low to low quality evidence. Therefore, we propose an adequately powered parallel, double-blind, stratified, randomised, placebo-controlled pragmatic trial of corticosteroids for acute otitis media in children (OPAL study), to address this uncertainty.

Prior to this, we will conduct a pilot study. This study will mimic the main study in terms of its process and procedures, but on a smaller scale. However, due to budget constraints, we will conduct a pilot study as a pragmatic, randomised, open-label, single-blind study, without using a placebo. The objectives of our pilot study are to: (1) assess the overall process and procedures of the main study; (2) verify a sample size calculation for main study; and (3) conduct a mechanistic explanatory study using tympanometry. We will include 60 children with AOM, who then, based on their AOM severity, will be stratified as mild or severe AOM, and randomly allocated to an intervention group (prednisolone) or control group (without prednisolone). An appointed nurse who performs the randomisation, patient, as well as the parents will be aware of treatment allocation, however both physicians and audiologists will remain unaware of the allocation at least until the data collection at Day-3. We will assess the following outcomes: (1) recruitment rate: (2) the success of the study procedures; (3) ability to measure planned outcomes in the main study; (4) the compliance to study and study drug; and (5) the verification of sample size calculation for main study. In the mechanistic study using tympanometry, we will assess: (1) the change in middle ear effusion at various time points; (2) the duration of middle ear effusion; and (3) the correlation between ear pain and other symptoms with the changes in middle ear effusion at various time points.

If corticosteroids prove effective in relieving pain or other relevant symptoms in children, and are safe, they could become a useful alternative to antibiotics in mild cases of AOM, and an addition to antibiotics in severe cases. The treatment is relatively cheap, easy to dispense and administer. It can

also reduce antibiotic use, particularly in mild cases of AOM. This could lead to a reduction in antibiotic resistance, thus saving precious antibiotics for severe diseases where rapid response to antibiotics is critical.

# References


1. Lieberthal AS, Carroll AE, Chonmaitree T, Ganiats TG, Hoberman A, Jackson MA et al. Clinical Practice Guideline: The diagnosis and management of acute otitis media. The American Academy of Pediatrics. Pediatrics. 2013;131:e964-e99.
2. Morris PS, Leach AM. Managing otitis media: an evidence-based approach. Aust Prescr. 2009;32:155-9.
3. Venekamp RP, Sanders SL, Glasziou PP, Del Mar CB, Rovers MM. Antibiotics for acute otitis media in children. Cochrane Database of Systematic Reviews 2015, Issue 6. Art. No.: CD000219. DOI: 10.1002/14651858.CD000219.pub4.
4. Pettigrew MM, Gent JF, Pyles RB, Miller AL, Nokso-Koivisto J, Chonmaitree. Viral-Bacterial Interactions and Risk of Acute Otitis Media Complicating Upper Respiratory Tract Infection. J Clin Microbiol. 2011;49(11):3750–5.
5. Chonmaitree T, Revai K, Grady JJ, Clos A, Patel JA, Nair S, et al. Viral upper respiratory tract infection and otitis media complication in young children. Clin Infect Dis. 2008;46(6): 815–23.
6. Rovers MM, Glasziou P, Appelman CL, Burke P, McCormick DP, et al. Antibiotics for acute otitis media: a meta-analysis with individual patient data. Lancet. 2006;368:1429-35
7. Costelloe C, Metcalfe C, Lovering A, Mant David, Hay AD. Effect of antibiotic prescribing in primary care on antimicrobial resistance in individual patients: systematic review and meta-analysis. *BMJ*. 2010;340:c2096. doi: 10.1136/bmj.c2096
8. Marom T, Marchisio P, Tamir SO, Torretta S, Gavriel H, Esposito S. Complementary and alternative medicine treatment options for otitis media. Medicine. 2016;95(6):e2695
9. Coleman C, Moore M. Decongestants and antihistamines for acute otitis media in children. Cochrane Database of Systematic Reviews 2011, Issue 3. Art. No.: CD001727. DOI: 10.1002/14651858.CD001727.pub5.
10. Ranakusuma RW, Pitoyo Y, Safitri ED, Thorning S, Beller EM, Sastroasmoro S, Del Mar CB. Systemic corticosteroids for acute otitis media in children. Cochrane Database of Systematic Reviews 2018, Issue 3. Art. No.: CD012289. DOI: 10.1002/14651858.CD012289.pub2
11. Chonmaitree T, Saeed K, Uchida T, Heikkinen T, Baldwin CD, Freeman DH, et al. A randomised, placebo-controlled trial of the effect of antihistamine of corticosteroid treatment in acute otitis media. *J Pediatr*. 2003;143:377-85.
12. McCormick DP, Saeed K, Uchida T, Baldwin CD, Deskin R, Lett-Brown MA, et al. Middle ear fluid histamine and leukotriene B4 in acute otitis media: effect of antihistamine or corticosteroid treatment. *Int J Pediatr Otorhinolaryngol.* 2003;67(3):221–30.

# The Protocol

See **Appendix 1. Protocol of Oral Prednisolone for Acute otitis media in chiLdren: a pilot pragmatic, randomised, open-label, single-blind controlled study (OPAL study).**

# Case Report Forms (CRFs)

To see **CRF01** to **CRF11** and **FORM01** to **FORM10**, please refer to **Appendix 2. Case report forms**.

# Setting Up The Study Site

Prior to the trial, the research team will set up the site for the trial. We will provide a starting kit along with an inventory checklist. The appointed nurse who works at the site will receive the starting kit. The starting kit contains:

1. Office stationery set (e.g. pen, highlighters, scissor, glue stick, stapler)
2. File binders:
    a. Confidential study document binder:
        i. FORM01: Study registration log book
        ii. CRF01: Completed consent form
        iii. CRF02: Completed study registration form
        iv. CRF08: Completed randomisation form
    b. Case report form binder:
        i. CRF01: Participant information sheet and consent form (will be separated to confidential study binder at the end of the initial visit)
        ii. CRF02: Study registration form (will be separated to confidential study binder at the end of the initial visit)
        iii. CRF03: Eligibility form
        iv. CRF04: Baseline information form
        v. CRF05: Outcome form
        vi. CRF06: Symptom diary
        vii. CRF07: Prescription of study medication
        viii. CRF08: Randomisation form (will be separated to confidential study binder at the end of the initial visit)
        ix. CRF09: Follow-up visit card
        x. CRF10: Serious adverse event reporting form
        xi. CRF11: Feedback form
        xii. FORM07: Guideline of antibiotics for acute otitis media in children
        xiii. FORM08: Prednisolone dose for OPAL study
        xiv. FORM09: Instruction of prednisolone use for parents
        xv. FORM10: Lupred pharmaceutical brochure
    c. Study medication storage binder for the pharmacists:
        i. FORM02: Study medication stock form
        ii. Form03: Study medication dispensing form
    d. Completed case report form and non-participating subject binder:
        i. FORM05: Recapitulation of completed case report form
        ii. FORM06: Recapitulation of non-participating subject form (e.g. not-eligible, not-consented individual forms)
    e. Study medication return binder:
        i. FORM04: Study medication return form
3. Medicine in a transparent storage container:
    a. Prednisolone tablets (Lupred® 5)
    b. Sirplus, a sweetener syrup
    c. FORM10: Pharmaceutical Lupred brochure

4. Study souvenirs:
    a. Initial visit (Day-0): Lunch box
    b. Visit-1 (Day-3): Water bottle
    c. Visit-2 (Day-7): Toys
    d. Visit-3 (Day-30): Mini towel
    e. Visit-4 (Day-90): Bag
5. Transportation cost reimbursement @$15
6. Inventory checklist form

The research team will visit and monitor the site at the end of the first week to check the completeness of study documentation (e.g. study document binders, case report forms), study medication stock and other supporting tools. The next monitoring visit will be conducted once every one or two weeks, depending on the recruitment flow of each site.

# Study medication delivery and storage

A research member will visit the site once every one or two weeks to deliver the study medication. When receiving the study medication, the pharmacist will record the quantity, the batch number of study medication, and the date of medicines received on **FORM02: Study medication stock form** (see Appendix 2).

The study medication will be stored at the pharmacy, in a cool dry place, protected from direct sunlight, where the temperature stays below 30°C, separately from other medication. We will provide **FORM10: Lupred pharmaceutical brochure.** The pharmacist will provide the brochure to the parents, along with the study medication and the instruction of its use.

# Rooms and other equipment for the study

In order to conduct the trial, we need several rooms and equipment which will be used for various activities, as follows:

1. Rooms:
    a. Nurse station:
        i. Identify the symptoms of acute otitis media using **FORM01: Study recruitment log boo**k.
        ii. Performing a general examination to measure body weight, height, temperature, and blood pressure.
    b. Consultation room:
        i. Delivering the information regarding the trial and obtain consent from the parents using **CRF01: Participant information sheet and consent form.**
        ii. Identifying the eligibility for the study and stratify the eligible children based on the severity of acute otitis media using **CRF03: Eligibility form.**
        iii. Obtaining baseline history information usimg **CRF04: Baseline information form**.
        iv. Identifying symptoms and signs of acute otitis media, including the severity of symptoms (using VAS and AOM-SOS), complication of acute

otitis media, medicine which have been taken prior the visit using **CRF05: Outcome form.**

v. Assessing the condition of nose, throat, as well as the ears using otoscope (if feasible)

vi. Explaining and tutor the parents in completing **CRF06: Symptom diary.**

vii. Assessing, interpreting, and recording the tympanometry results

viii. Preparing the **CRF07: Prescription of study medication** (see Appendix 10) for every study participant with doses according to their ages. The guideline for prednisolone dose is available in **FORM08: Prednisolone dose for OPAL study** and at the top of CRF07. Prescription of study medication

ix. Prescribing other medications, such as antibiotics for children with severe AOM based on physician's clinical preference or based on **FORM07: Guideline of antibiotics for AOM in children** and symptomatic medications, if necessary

c. Audiology room/corner:

  i. Conducting a tympanometry examination.

d. Private room for randomisation process:

  i. Performing and recording the randomisation of intervention allocation using **CRF08: Randomisation form**

  ii. Dispensing the prescription of study medication for study participants who are allocated to intervention (prednisolone) group.

  **iii.** Reconfirming the parent's understanding and ability in completing **CRF06: Symptom diary**

  iv. Educating the parents on the use of prednisolone, identification of potential side effects, and provide the information regarding 24-hour emergency call centre. The nurse will provide **FORM09: Instructions of prednisolone use for parents**

  v. Requesting the parent to complete **CRF02: Study registration**

  vi. Completing **CRF09: Follow-up visit card** with scheduled visits in the next three months

  vii. Providing the study souvenir and transport cost reimbursement to each study participant

  viii. Collecting and checking all the study documents, storing them according their binder, and secure the binders in a locked filing cabinet:

    1. The confidential study document binder (compilation with other study participant documents):
        a. FORM01: Study registration log book.
        b. CRF01: Completed consent form
        c. CRF02: Completed study registration form.
        c. CRF08: Completed randomisation form.
    2. The case report form binder (no name on each form and only identified by an ID registration):
        a. CRF03: Eligibility form
        b. CRF04: Baseline history form

c. CRF05: Outcome form
e. CRF10: Serious adverse events reporting form
f. CRF11: Feedback form

2. Equipment:
    a. Weight scale
    b. Measuring tape
    c. Thermometer
    d. Paediatric tensimeter
    e. Head lamp
    f. Tongue depressor
    g. Rhinoscope
    h. Otoscope
    i. Tympanometry
    j. Copy machine/scanner
    k. Office stationery
    l. Smart phone with internet connection
    m. Telephone
    n. Filing cabinet

# Step-by-step Procedure

## The nurse station

### Objective(s)

1. Initially identify of children with acute otitis media
2. Perform general examination (weight, height, body temperature, blood pressure)
3. Provide **CRF01: Participant information sheet and consent form** and **CRF02. Study registration form** for the parents
4. Prepare the study binders and case report forms

### Tool(s)

1. Weight scale
2. Measuring tape
3. Thermometer
4. Paediatric tensimeter
5. FORM01. Study recruitment log book
6. Case report form binder
7. Registration ID labels
8. OPAL study stickers

### Personnel

1. Attending nurse.

### Procedures

1. When the medical record arrives, the attending nurse will identify whether the patient has AOM symptoms using three screening questions in **FORM01: Study recruitment log book** (see Figure 1), as follows:
    a. Has your child experienced ear pain in the past 48 hours?
    b. Has your child been tugging or rubbing her/his ear(s) and been more irritable or fussy or crying more than usual over the past 48 hours?
    c. Has your child been experiencing ear discharge in the past 48 hours?

| FORM01 – STUDY RECRUITMENT LOG BOOK | | | | | | | | | | | | | | |
|---|---|---|---|---|---|---|---|---|---|---|---|---|---|---|
| Nurse name/ID : | | | | Study title : Oral prednisolone for acute otitis media in children: a pilot, pragmatic, randomised, open-label, single-blind, controlled study (OPAL study) | | | | | | | Hospital ID : | | | |
| Study registration ID | Patient's name | Date screened | Has your child experien-cing ear pain in the past 48 hours? (YES or NO) | Has your child been tugging or rubbing her/his ear(s) and been more irritable or fussy or crying more than usual over the past 48 hours (YES or NO) | Has your child been experie ncing ear discharg e in the past 48 hours? (YES or NO) | Body weight (kg) | Body height (cm) | Body tempe-rature (°C) | Blood pressure (mmHg) | Did patient go on the study? (YES or NO) | If YES, what is the Randomisation ID | If NO, please tell us reason not on the study below | | |
| | | | | | | | | | | | | Not eligible (YES or NO) | Did not give consent (YES or NO) | Was not approached (YES or NO). Write the reason |
| | | | | | | | | | | | | | | |
| | | | | | | | | | | | | | | |

Figure 1. FORM01: Study recruitment log book.

2. If the parent responds **'YES' to one of these three questions**, the attending nurse will then perform general examination (i.e. body weight and height, temperature, blood pressure) and record the results on **FORM01. Study recruitment log book**. The nurse will also prepare the case report forms which are compiled in one binder (case report form binder), as follows:
    a. CRF01: Participant information sheet and consent form.
    b. CRF02: Study registration form
    c. CRF03: Eligibility form.
    c. CRF04: Baseline information form.
    d. CRF05: Outcomes form.
    e. CRF06: Symptom diary.
    f. CRF07: Study medication prescription.
    g. CRF08: Randomisation form
    h. CRF09: Follow-up visit card
    l. CRF10: Serious adverse events reporting form
    m. CRF11: Feedback form
    m. FORM07: Guideline of antibiotics for acute otitis media in children
    i. FORM08: Prednisolone dose for OPAL study
    i. FORM09: Instruction of prednisolone use for parents
    j. FORM10: Lupred pharmaceutical brochure
3. If possible, the nurse will provide **CRF01: Participant information sheet and consent form** and **CRF02: Study registration form** for the parents so they can read the information sheet and complete the registration form while they are waiting for consultation.
4. The nurse will then report this patient to a physician as a potential study participant along with **FORM01: Study recruitment log book** and **the case report form binder** with a specific registration ID label attached on every form in the binder. The form and binder will be inserted in the medical record of that particular patient. Therefore, the physician will notify this patient as a potential study participant for OPAL study

# The consultation room

## Study consent, recruitment, and stratification

### Objective(s)

1. Identify the eligibility of potential children to be included in the trial
2. Provide sufficient information regarding the research, including the overall process and potential effects caused by the study
3. Obtain the consent from eligible patients and their parents to participate or not to participate in the study
4. Stratify the eligible patients based on their AOM severities

### Tool(s)

1. FORM01: Study recruitment log book
2. Case report form binder:
    a. CRF01: Participant information sheet and consent form
    b. CRF02: Study registration form
    c. CRF03: Eligibility form

3. Non-participating subject form binder:
    a. FORM06: Recapitulation form of non-participating subject form.

## Personnel

1. Participating clinicians

## Procedures

1. The attending nurse will notify the physician that the patient has AOM symptoms and provide the physician with **FORM01: Study recruitment log book** and **Case report form binder**.
2. The physician will re-confirm the eligibility of the patient using inclusion and exclusion criteria on the **CRF03: Eligibility form** (see Appendix 1. Protocol, section 'Eligibility criteria', page 15-16). If the parent has responded **'YES' to all inclusion criteria and 'NO' to all exclusion criteria, then the patient is eligible for the study**. However, if the parent has responded at least one 'NO' to the inclusion criteria or one 'YES' to the exclusion criteria, then the patient is not eligible for the study (see Figure 2).

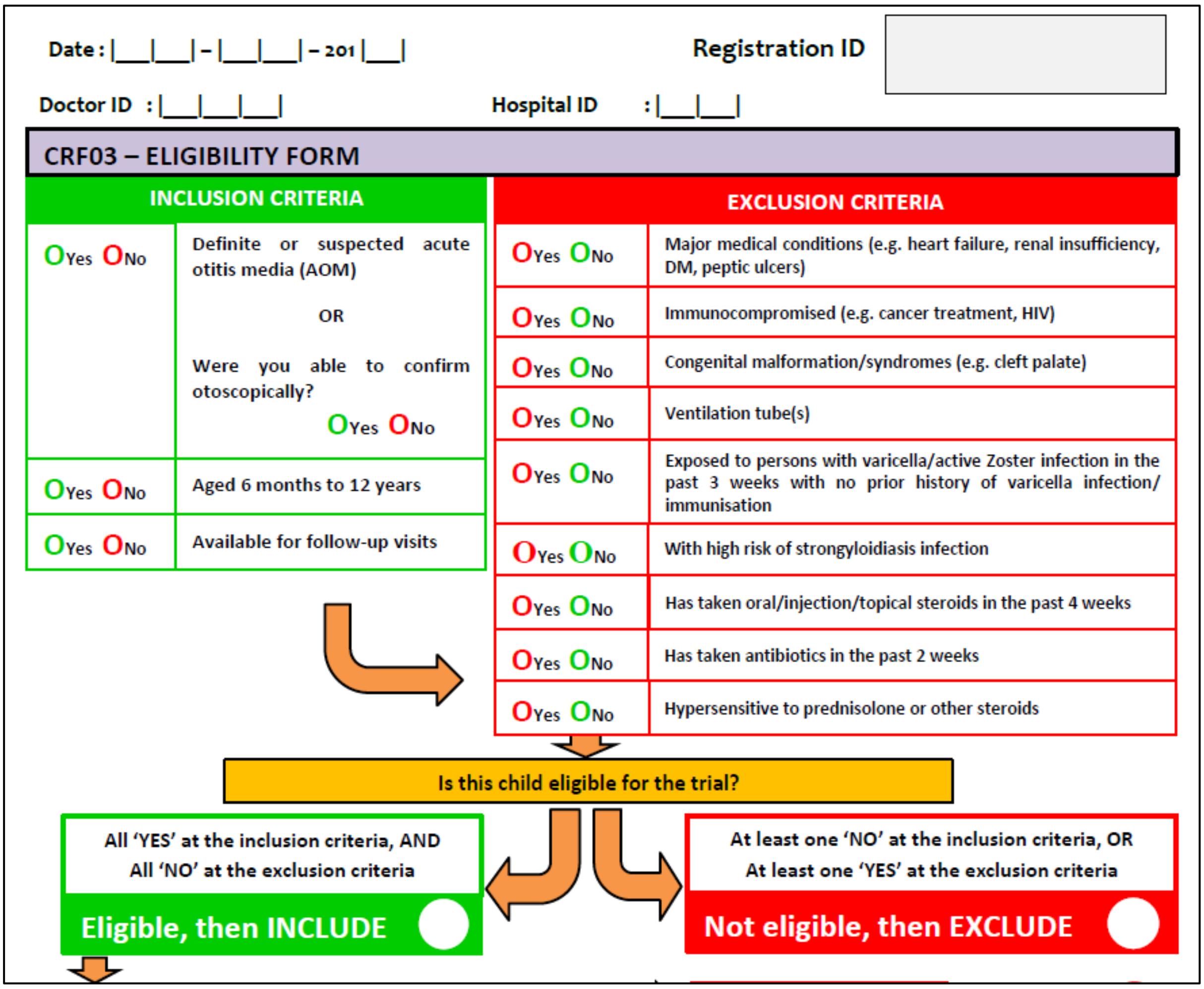

Date : |___|___| - |___|___| - 201 |___|    Registration ID

Doctor ID : |___|___|___|    Hospital ID : |___|___|

CRF03 – ELIGIBILITY FORM

| INCLUSION CRITERIA | |
|---|---|
| OYes ONo | Definite or suspected acute otitis media (AOM)<br>OR<br>Were you able to confirm otoscopically? OYes ONo |
| OYes ONo | Aged 6 months to 12 years |
| OYes ONo | Available for follow-up visits |

| EXCLUSION CRITERIA | |
|---|---|
| OYes ONo | Major medical conditions (e.g. heart failure, renal insufficiency, DM, peptic ulcers) |
| OYes ONo | Immunocompromised (e.g. cancer treatment, HIV) |
| OYes ONo | Congenital malformation/syndromes (e.g. cleft palate) |
| OYes ONo | Ventilation tube(s) |
| OYes ONo | Exposed to persons with varicella/active Zoster infection in the past 3 weeks with no prior history of varicella infection/ immunisation |
| OYes ONo | With high risk of strongyloidiasis infection |
| OYes ONo | Has taken oral/injection/topical steroids in the past 4 weeks |
| OYes ONo | Has taken antibiotics in the past 2 weeks |
| OYes ONo | Hypersensitive to prednisolone or other steroids |

Is this child eligible for the trial?

All 'YES' at the inclusion criteria, AND
All 'NO' at the exclusion criteria
Eligible, then INCLUDE

At least one 'NO' at the inclusion criteria, OR
At least one 'YES' at the exclusion criteria
Not eligible, then EXCLUDE

Figure 2. CRF03: The eligibility form – The inclusion and exclusion criteria.

3. After it has been confirmed that the child is eligible for the trial, then the physician will deliver the information regarding the trial and obtain the consent for the trial using **CRF01: Participant information sheet and consent form** (see Appendix.1 Protocol, section 'Consent', page 26-27). The physician will give the opportunity for the parents to

read the information sheet and raise questions, and will provide further information if necessary.

4. After the physician identifies the eligibility and obtains the consent for the study, the physician will then stratify the study participant based on the severity of the AOM symptoms according to the following criteria on **CRF03: Eligibility form** (see Appendix.1 Protocol, section 'Participant enrolment', page 19-20). :
    a. Moderate to severe symptoms, locally or systemically (e.g. moderate to severe ear pain, fever ≥ 39°C, complications)
    b. Aged <2 years with bilateral acute otitis media
    c. AOM with perforation of tympanic membrane(s)
    d. If visible, otoscopic finding shows moderate to severe bulging and/or yellowish purulent tympanic membrane(s)

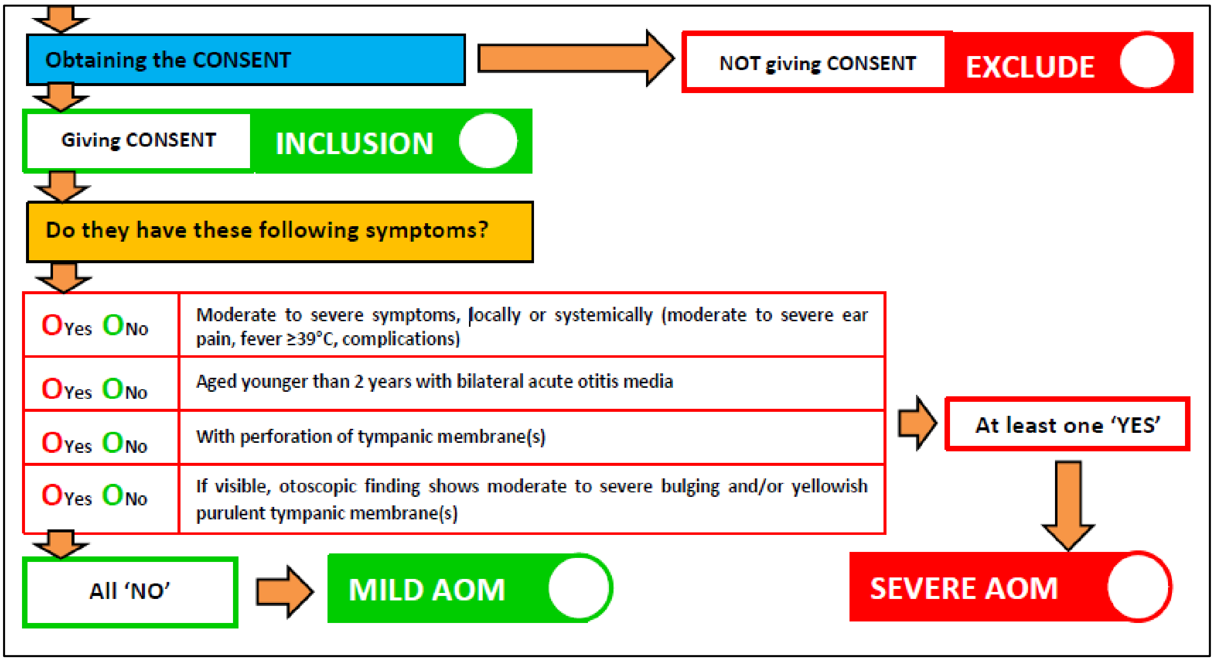


Figure 3. CRF03: The eligibility form – The consent and stratification based on severity.

5. If **the study participant has at least one 'YES' on the criteria of a to c, then the patient will be allocated to 'SEVERE AOM' group**. If the patient has **all 'NO' for a to c criteria, then the patient will be allocated to 'MILD AOM' group**. The fourth criterion (otoscopic finding) is an optional criterion because of the difficulty in identifying the tympanic membranes in small children. However, if it is visible, the physician will tick the circle corresponding to the otoscopic finding
6. For children who are stratified to 'SEVERE AOM' group, the physician will prescribe antibiotics according to their clinical preferences. However, we will provide **FORM07: Guideline of antibiotics for acute otitis media in children**.
7. The physician will then complete **FORM01: Study recruitment log book** by providing information regarding:
    a. Whether the patient participated in the study
    b. Reasons (i.e. not eligible, did not give consent, was not approached) for those who did not participate in the study
8. The nurse will complete the column 'The randomisation ID for those who went to the study' after the child has been randomised to either intervention or control group.
9. The physician will separate the case report form of children who are not eligible or do not give consent. Their eligibility forms will be removed from their case report binders and will be stored in the **Non-participating subject form binder**, after they ensure that all forms

have the registration ID label on the top right of each form. This will be recorded on **FORM06: Recapitulation of non-participating subject form.**

# Collecting baseline data and examination

## Objective(s)

1. Obtain relevant information to acute otitis media
2. Identify of AOM complications, severity of AOM symptoms, and history of medication
3. Perform ear, nose, and throat examination, as well as interpret the tympanometry examination result
4. Teach the parents of study participant to complete the symptom diary

## Tool(s)

1. Head lamp
2. Tongue depressor
3. Rhinoscope
4. Otoscope
5. Tympanometry
6. Case report form binder:
    a. CRF04: Baseline information form
    b. CRF05: Outcomes form
    c. CRF06: Symptom diary
    d. CRF07: Study medication prescription
    e. FORM07: Guideline of antibiotics for acute otitis media in children
    f. FORM08: Prednisolone dose for OPAL study

## Personnel

1. Participating physicians
2. Audiologist

## Procedures

1. The physician will obtain further baseline information using the **CRF04: Baseline information form**, such as the breastfeeding history, day-care attendance, vaccination history.
2. The physician will then obtain more detailed information relevant to acute otitis media using **CRF05: Outcome form**, such as symptoms and complications of acute otitis media: ear discharge, intense pain in and behind the ear, swelling behind the ear, or facial asymmetry
3. The physician will copy the result of general examination from **FORM01: Study recruitment log book** (i.e. body weight, height, temperature, blood pressure) measured by the attending nurse.
4. The physician will identify the condition of nose and throat and record the results by ticking the circles corresponding to the findings. (see Figure 4)
5. The physician will perform an otoscopic examination and record the result by ticking the circles corresponding to the findings: normal, erythema, air-fluid level, complete effusion, opacification and mild bulging, moderate to severe bulging, bulla, and/or perforation (see Figure 4 and Figure 5).

| CRF05 – OUTCOME FORM | | |
|---|---|---|
| **Baseline Visit (Day-0) : \|___\|___\| – \|___\|___\| – 20 \|___\|___\|** | | |
| Complications (for Physician) | | |
| 1 | Does your child experience discharge from the ear(s)? | O Yes O No |
| 2 | Does your child experience intense ear pain and pain behind the ear? | O Yes O No |
| 3 | Does your child experience swelling/bulging/ or redness/tenderness of the ear(s)? | O Yes O No |
| 4 | Does your child experience facial asymmetry (e.g. when the child smiles, cries)? | O Yes O No |
| General and ENT examination (for Nurse and Physician) | | |
| 5.1 Weight _____ kg | 5.2 Height ______ cm | 5.3 Temp. ______ °C · 5.4 BP _____/_____ mmHg |
| 6 | Nose | O Normal O Oedema O Hyperaemic O Livid O Serous discharge O Mucoid discharge |
| 7 | Tonsils | O Normal O Hyperaemic O Detritus O Tonsil(s) T1 O Tonsil(s) T2 O Tonsil(s) T3-4 |
| 8 | Pharynx | O Normal O Hyperaemic O Oedema O Granules O Post nasal drip (PND) |
| 9 Otoscopic examination | | |
| | | O Normal O Cerumen O Erythema O Air fluid level O Complete effusion O Opacification |
| | | O Mild bulging O Moderate to severe bulging (bulging rounded) O Bulla O Perforation |

Figure 4. CRF05: The outcome form – General, ear, nose, throat, and otoscopic

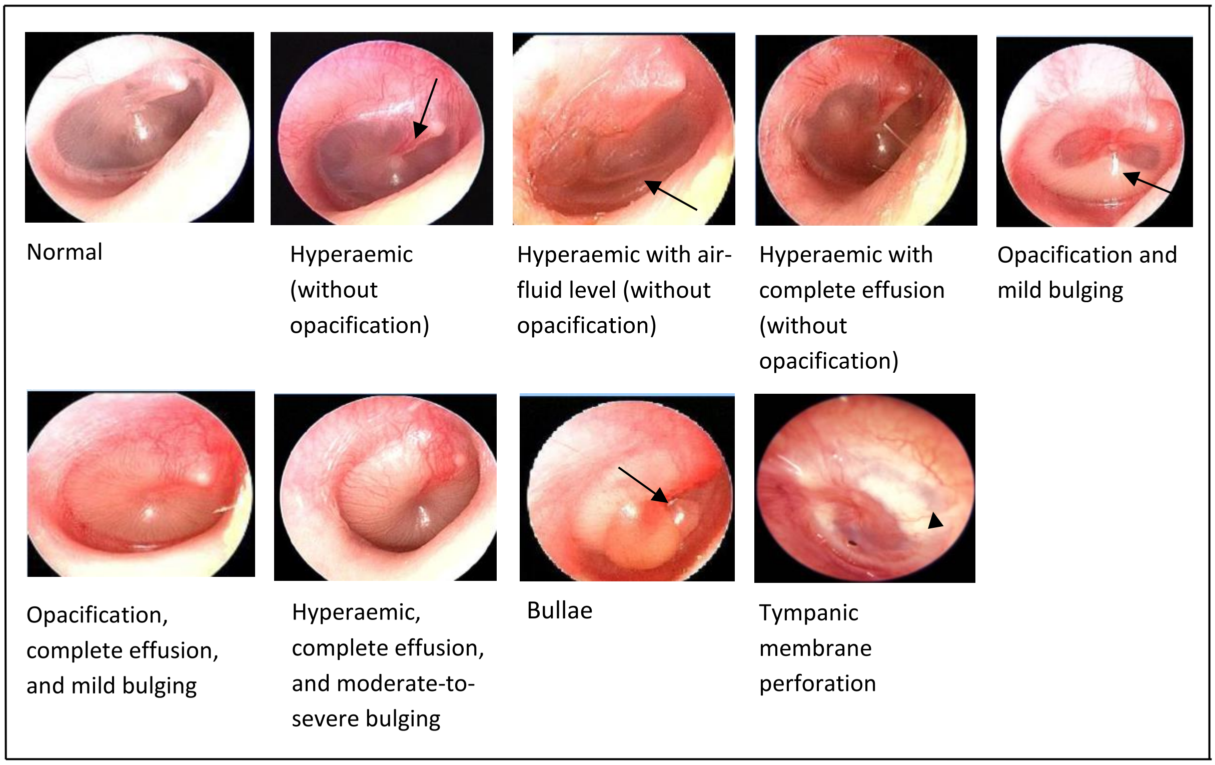


Figure 5. The otoscopic characteristics of acute otitis media.

6. The physician will then identify and record any medications that have been taken before the baseline visit prescribed by other physicians or purchased over-the-counter (e.g. antibiotics, analgesics, decongestants). The physician will also record the name and dose of medications prescribed for the study participants.
7. The physician will identify the severity of the ear pain using visual analogue scale (VAS) (see **Appendix 1. Protocol, section 'Data collection methods', page 22-23**). The VAS is a 100-mm horizontal line. The left end of the line represents 'no pain' and the right end of the line represents 'pain as bad as it could possibly be'. The physician will ask the parent to place a vertical line across the horizontal line that corresponding to the severity of ear pain during

the past 24 hours. This will also be included in **CRF06. Symptom diary**; therefore at the same time, the physician will teach the parent to complete this scale in the diary, so the parents will be able to complete this at home (see Figure 6).

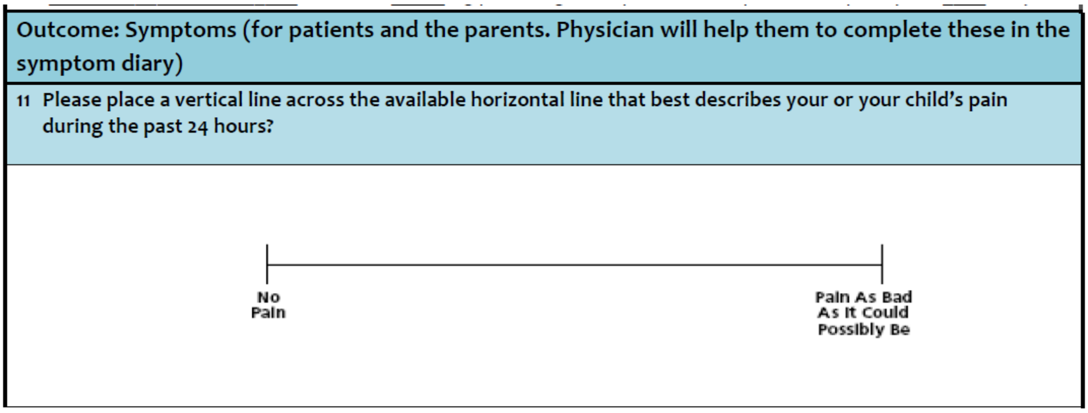
Outcome: Symptoms (for patients and the parents. Physician will help them to complete these in the symptom diary)

11 Please place a vertical line across the available horizontal line that best describes your or your child's pain during the past 24 hours?



Figure 6. CRF05: Outcome form – Visual analogue scale (VAS).

8. The physician will then identify the severity of other relevant-symptoms of acute otitis media using acute otitis media severity (AOM-SOS) (see **Appendix 1. Protocol, section 'Data collection methods', page 23-24**). This consists of seven questions identifying several symptoms as follows: has the child (1) been tugging/rubbing the ear(s)?; (2) been crying?; (3) been more irritable or fussy?; (4) been having more difficulty sleeping; (5) been less playful or active?; (6) been eating less than usual?; and (7) been having fever or feeling warm to touch over the past 12 hours?. These questions are very important for young children, particularly those who cannot express their symptoms (non-verbal children). The parent will tick the circle corresponding to the child's symptoms. This will also be included in the **CRF06: Symptom diary**; therefore, the physician will teach the parent to complete this questionnaire in the diary, so they will be able to complete this at home (see Figure 7).

| 12 We are interest finding out how your child has been doing. For each question, please place a checkmark (V) in the circle corresponding to your child's symptoms. Please answer all questions. | | | |
|---|---|---|---|
| 12.1 Over the past 12 h, has your child been tugging, rubbing, or holding the ear(s) more than usual? | O No | O A little | O A lot |
| 12.2 Over the past 12 h, has your child been crying more than usual? | O No | O A little | O A lot |
| 12.3 Over the past 12 h, has your child been more irritable or fussy than usual? | O No | O A little | O A lot |
| 12.4 Over the past 12 h, has your child been having more difficulty sleeping than usual? | O No | O A little | O A lot |
| 12.5 Over the past 12 h, has your child been less playful or active than usual? | O No | O A little | O A lot |
| 12.6 Over the past 12 h, has your child been eating less than usual? | O No | O A little | O A lot |
| 12.7 Over the past 12 h, has your child been having fever or feeling warm to touch? | O No | O A little | O A lot |

Figure 7. CRF05: Outcome form – Acute otitis media using acute otitis media severity (AOM-SOS).

9. The physician then will provide **CRF06: Symptom diary** to the parent. The symptom diary consists of three mini booklets:
    a. The first mini booklet: The parent must complete the booklet after the baseline visit to Day-3. The appointed nurse will collect this mini booklet at Visit-1 (Day-3)
    b. The second mini booklet: The parent must complete the booklet at Day-4 to Day-7. The appointed nurse will collect this mini booklet at Visit-2 (Day-7)

c. The third mini booklet: The parent must complete the booklet at Day-8 to Day-14. The research team will collect this mini booklet at Day-14 during the home visit

10. The physician will then send the study participant to the audiology room for tympanometry examination after ensuring the ear canals are free from obstructing ear wax. Prior to this, the physician will explain the purpose and the procedure of tympanometry examination to the parents
11. After the tympanometry examination, the study participant and the parent will return to the consultation room with the tympanometry result. The physician will analyse and interpret the tympanometry findings by completing the curve type of the tympanogram.
12. Before the study participant leaves the consultation room to meet the nurse who will conduct a randomisation, the physician will prepare **CRF07: Prescription of study medication** (see Figure 8) and put it back in the **case report form binder**. The prednisolone dose can be accessed in **FORM08: Predisolone dose for OPAL study** and at the top of **CRF07: Precription of study medication**. This prescription will be dispensed by the appointed nurse to children who are allocated in the intervention group.

dr. Respati W. Ranakusuma, SpTHT-KL
Clinical Epidemiology & Evidence-Based Medicine Unit, Dr. Cipto Mangunkusumo Hospital – Faculty of Medicine Universitas Indonesia
Oral Prednisolone for acute otitis media in children: a pilot pragmatic, randomised, open-label, single-blind, controlled study (OPAL Study)

Date ______________________

**CRF07. Prescription for OPAL study medication**

**Prednisolone doses:**
- Aged 6 months to < 2 years old = 10 mg per day
- Aged 2 years to < 6 years old = 20 mg per day
- Aged 6 years to 12 years old = 30 mg per day

Registration ID :
Name : ________________________
Age : ________________ months / year(s) *[write and circle your answer]*
Study medication dose : ________ mg per day = ________ tablets per day

R/ OPAL study medication tablet ........
Sach lact add
m.f. pulveres dtd No. V
∫ 1 dd 1 pc (before 9 am)

(sign here)

Figure 8. CRF07: Prescription of study medication.

13. The physician will also prescribe antibiotics for study participants with severe AOM based on the physician's clinical preference. We will provide **FORM07: Guideline of antibiotics for AOM in children** for their reference. The physician may prescribe symptomatic medication. We will ask them not to prescribe steroids
14. The physician will advise the parent to closely observe the study participant for 48 hours for persistence or the worsening of the symptoms (i.e. ear pain, fever) and potential complications (i.e. ear discharge, swollen on the back of the ear). If the parents identify any condition above, they should bring the study participant back to the hospital at any time for re-assessment and the initiation of antibiotic treatment
15. At the following visits (Day-3), the physician will identify any side effects such as abdominal pain, vomiting, diarrhea, anxiety. The physician will also identify whether the parent should

seek any medical help due to the side effects (e.g. go to other physicians, go the emergency department) and whether the parent should continue or stop the medication

16. The physician will record the additional medication or treatment or examination that is given due to the side effects or whether the study participant requires a hospitalisation.

# Tympanometry test

## Objective(s)

1. Measure the condition in the middle ear

## Tool(s)

1. Tympanometer
2. Otoscope
3. Case report form binder:
    a. CRF05. Outcome form
4. Glue stick
5. Copy machine or scanner

## Personnel

1. Audiologist or trained nurse

## Procedures

1. The audiologist will explain the procedure of tympanometry examination
2. The audiologist will ask the parent to hold the child's head firmly. Then the audiologist will identify the condition of ear canal using an otoscope to identify any ear wax and estimate the size of the ear cuffs. After connecting the ear cuffs to a probe tip, the audiologist will slowly insert the probe tip into the ear canal until the graphs appear on the screen. This procedure will be repeated on the other side of the ear. After the graphs indicate sufficient results (e.g. no air leak, no block), the result is then ready to be printed.
3. The audiologist will document the tympanometry results on **CRF05: Outcome form – Tympanometry examination** (see Figure 9), as follows: (1) ear canal volume; (2) compliance; (3) static acoustic admittance; (4) middle ear pressure for both ears. Several tympanogram machines use terminology 'compliance' for 'statistic acoustic admittance', therefore we provide both components in this section to avoid confusion. The physician will analyse and determine the type of tympanogram curve after this examination.

**13 Tympanometry examination (for Audiologist and interpreted by Physician)**

O Cannot be performed. Reason: ______________________________

Tympanogram types (will be completed by physician) [R] Type ______ / [L] Type ______

| | | |
|---|---|---|
| Ear canal vol (ECV) | [R] ________ mL | / [L] ________ mL |
| Static acoustic admittance | [R] _______ mL | / [L] _______ mL |
| Compliance (SC) | [R] ________ mL | / [L] ________ mL |
| Middle Ear Pressure or TPP | [R] _______ daPa | / [L] ______ daPa |
| Gradient or TW | [R] _______ daPa | / [L] ______ daPa |

*Put the copy of tympanometry copies here*

Figure 9. CRF05: Outcome form – Tympanometry examination.

4. The audiologist will attach the study registration ID label and write the date of examination on the printed tympanometry result paper, copy (using the copy machine or scanner), and attach the copied tympanometry result on the 'Tympanometry examination' section.
5. After the examination, the audiologist will send the study participant and the parent back to the consultation room. The physician then will analyse and interpret the tympanometry findings and conclude the consultation in his/her usual way.

# Randomisation

## Objective(s)

1. Randomly allocate the children to receive either prednisolone (intervention group) or none (control group)

## Tool(s)

1. Smart phone or computer with internet connection for accessing the randomisation website
2. Telephone to call a randomisation call-centre
3. Case report form binder:
    a. CRF01: Participant information sheet and consent form
    b. CRF02: Study registration form
    c. CRF03: Eligibility form
    d. CRF06: Symptom diary
    e. CRF07: Prescription of study medication
    f. CRF08: Randomisation form
    g. CRF09: Follow-up visit card
    h. FORM08: Prednisolone dose for OPAL study
    i. FORM09: Instruction of prednisolone use for parents
    j. FORM10: Lupred pharmaceutical brochure
4. Confidential study document binder:
    a. Completed FORM01. Study recruitment log book
    b. Completed CRF01. Completed consent form
    c. Completed CRF02. Completed study registration form
    d. Completed CRF08. Completed randomisation form
5. Completed case report form and non-participating subject binder:
    a. Non-participating subject form (e.g. not-eligible, not-consented individual forms)
6. Study souvenirs
7. Transport cost reimbursement envelope

## Personnel

1. Appointed nurse

## Procedures

1. The appointed nurse will complete **CRF0: Randomisation form** to randomise the intervention allocation for each patient (see Figure 10). The appointed nurse requires **FORM01: Study recruitment log book; CRF01: Consent form;** and **CRF02: Eligibility form** to be able to answer several questions and confirm the answers, as follows:

a. **Part 1 – Eligibility criteria**: all 'YES' for all inclusion criteria and all 'NO' for all exclusion criteria. The nurse will check the eligibility of the study participant using **FORM01: Study recruitment log book** and **CRF03: Eligibility form**
b. **Part 2 – Consent to the study**: consent to the study has been given. The nurse will check the consent to the study using **CRF01: Consent form** and **CRF03: Eligibilty form**
c. **Part 3 – Randomisation information**: Parents' mobile numbers, the severity of AOM (The nurse will check the severity using **CRF03: Eligibilty form**), and patient's date of birth and age.
d. **Part 4 – Randomisation result**: randomisation ID, intervention allocation (prednisolone or no prednisolone / control group), and prednisolone dosage for patient who is allocated to prednisolone group. The nurse will use **FORM08: Prednisolone dose for OPAL study** as a guidance for the prednisolone dose. The dosages of prednisolone will be determined based on age, as follows:
    i. Children aged 6 months to < 2 years will receive 10 mg per day (or 2 tablets per day) for 5 days
    ii. Children aged 2 years to < 6 years will receive 20 mg per day (or 4 tablets per day) for 5 days
    iii. Children aged 6 years to 12 years will receive 30 mg per day (or 6 tablets per day) for 5 days

| CRF08 – RANDOMISATION FORM | | | | | | |
|---|---|---|---|---|---|---|
| Eligibility criteria (cross-check with 'FORM01. study registration log book', and 'CRF03. Eligibility form' in the 'Case Report Form Binder' of this subject). | | | | | | |
| All YES for all inclusion criteria | | | | | O Yes | O No |
| All NO for all exclusion criteria | | | | | O Yes | O No |
| Consent to the study questions (cross-check with 'CRF01. Informed consent' in the 'Case Report Form Binder' of this subject). | | | | | | |
| Has consent given? | | | | | O Yes | O No |
| RANDOMISATION | | | | | | |
| Father's mobile phone number | | | | | | |
| Mother's mobile phone number | | | | | | |
| Severity of AOM | O Mild AOM | | | O Severe AOM | | |
| Subject's date of birth | Date | Month | Year | AGE | | Month/year |
| RANDOMISATION RESULT | | | | | | |
| Randomisation ID | | | | | | |
| This subject is allocated to | O Prednisolone group | | O Control group (no prednisolone) | | | |
| Prednisolone dosage (if the subject is allocated to prednisolone group) | O 10 mg/day | O 20 mg/day | | O 30 mg/day | | |
| Nurse's signature | | Nurse's name | | Date | | |

Figure 10. CRF08: Randomisation form.

2. The nurse will use Part 1 to Part 3 to obtain the randomisation result in Part 4 section. This can be accessed within two ways, as follows:
    a. Randomisation website:
        i. The appointed nurse will receive an invitation email from **MASCOT.org.au.** This email will notify the nurse regarding the name of the study, which is **OPAL STUDY**, and the name of institution (e.g. Cipto Mangunkusumo Hospital, etc.). Prior to the study, we obtained a list of email addresses of potential appointed nurses during the training session. For practice, we sent the invitation email from MASCOT.org.au with

the title of study of **PRACTISE STUDY**. The nurses were able to use this link to practice as much as they like until they are competent to use this tool.

ii. At the end of the email, there is a link to the **MASCOT study randomization system website** (see Figure 11).

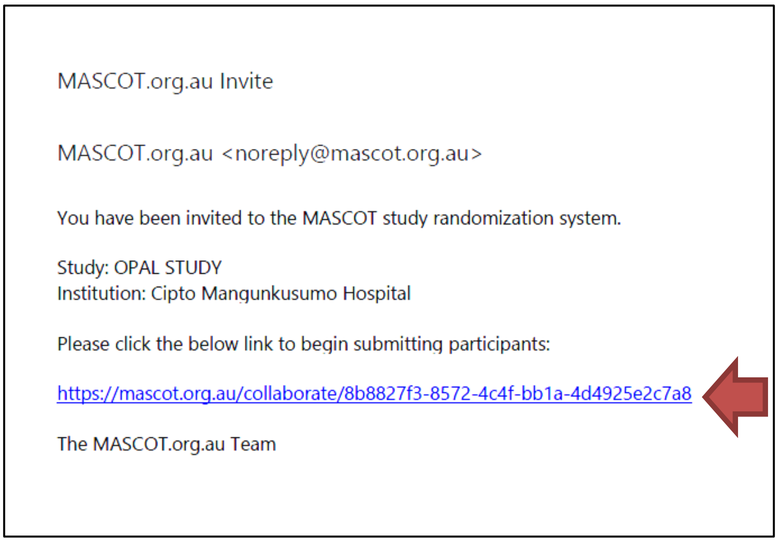


Figure 11. The MASCOT study randomization system – Invitation email for MASCOT study

iii. After clicking the link, the attending nurse will automatically open the login page of the MASCOT study randomization system website. The first page will confirm the name and institution. If the information is correct, the nurse will click **'Begin'** button (see Figure 12).

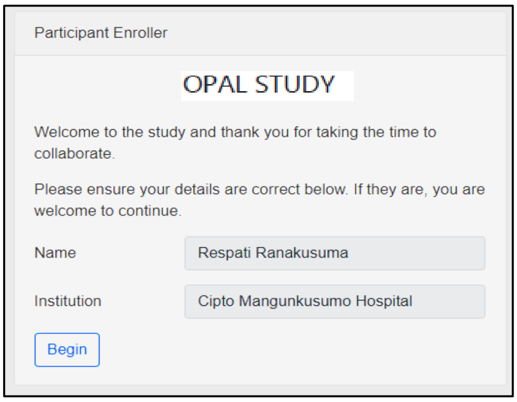


Figure 12. The MASCOT study randomization system – The enroler confirmation page.

After that, the page will ask the nurse to insert the Registration ID of the study participant (see Figure 13). The nurse will use the ID based on the Registration ID label attached on the top right of case report forms and the binder.

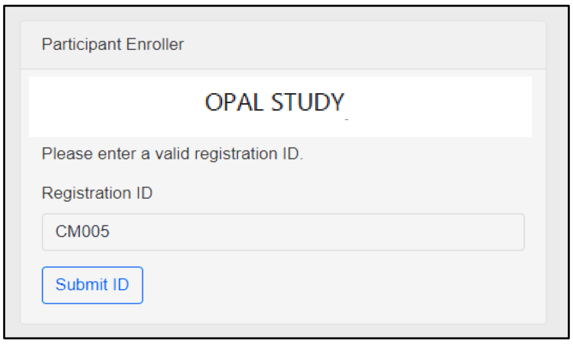


Figure 13. The MASCOT study randomization system – The study

iv. The next page presents several questions regarding the eligibility criteria (inclusion and exclusion criteria) and whether the consent the study has been given, as follows:
   1. All 'YES' for all inclusion criteria
   2. All 'NO' for all inclusion criteria
   3. Has consent been given

   The nurse will then click **'Select' → 'Yes' to all questions to be able to proceed to the next step**. To proceed, the nurse will click **'Check Eligibility'** button at the left bottom of the page (see Figure 14).

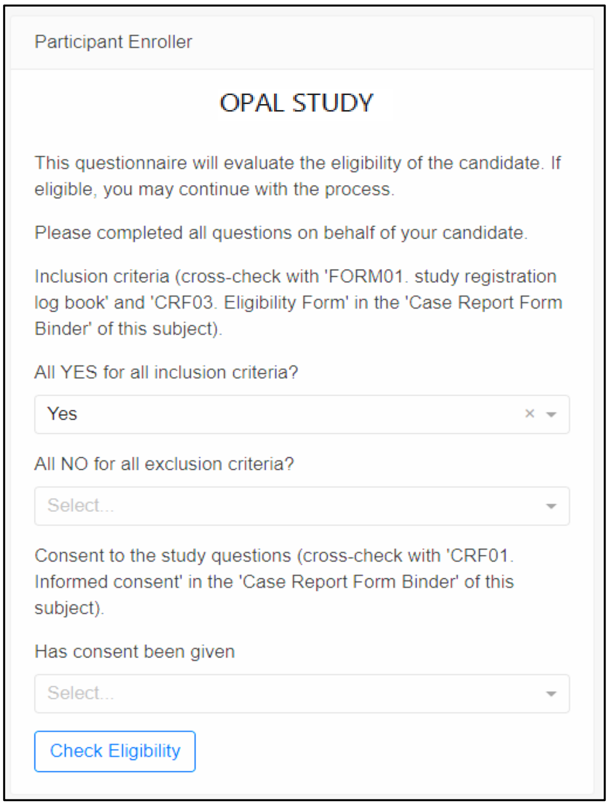


Figure 14. The MASCOT study randomization system – The eligibility page.

v. The next page will show whether the study participant is allocated to prednisolone or control (no prednisolone) group. The attending nurse will insert the date of birth using an available calendar or put the month (mm) / date (dd) / year (yyyy) manually. The nurse will check the date of birth on the available calendar. The page will automatically convert this information to age of that particular patient (please recheck the age). The nurse will select the severity of AOM based on the information in CRF03. Randomisation form, as a 'Mild AOM' or 'Severe AOM' accordingly. To proceed, the nurse will then select **'Submit Answers'** button. The nurse must ensure that the submitted answers are correct as there **will be no opportunity** to correct the answers after they have been submitted (see Figure 15).

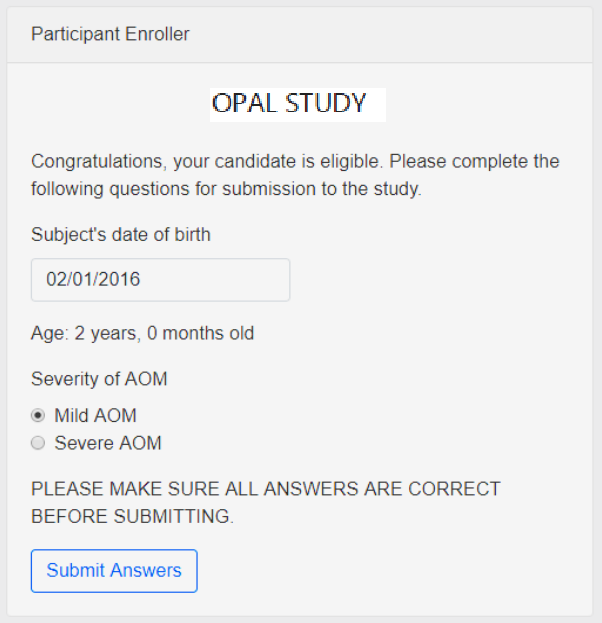


Figure 15. The MASCOT study randomization system – The date of birth and AOM severity page.

vi. If the patient is allocated to the prednisolone group, then the page will require information of the dose of prednisolone:
   1. Children aged 6 months to < 2 years will receive 10 mg per day (or 2 tablets per day) for 5 days
   2. Children aged 2 years to < 6 years will receive 20 mg per day (or 4 tablets per day) for 5 days
   3. Children aged 6 years to 12 years will receive 30 mg per day (or 6 tablets per day) for 5 days

   The nurse must ensure that the prednisolone has been prescribed according to the dose on **CRF07. Prescription of OPAL study.** This must be prepared by the physician before the randomisation process. The nurse can also check the dose using **FORM08. Prednisolone dose of OPAL study.** The nurse will then select the dose accordingly. The nurse will select **‘Submit’** button (see Figure 16).

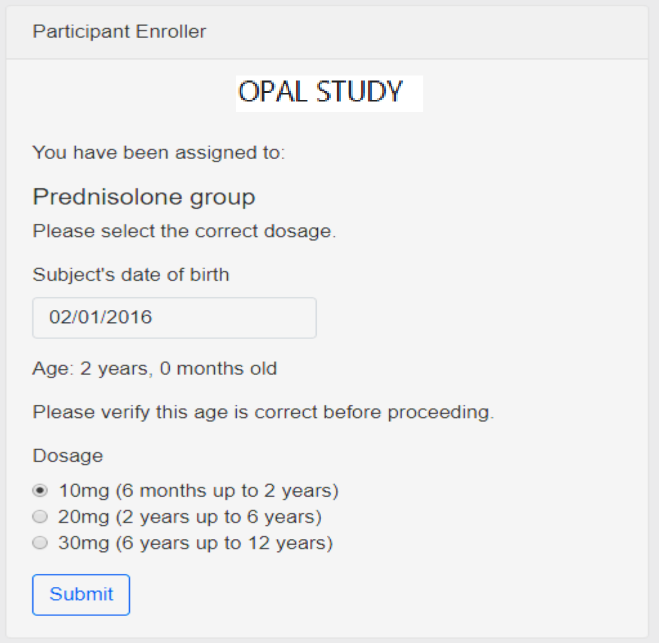


Figure 16. The MASCOT study randomization system – The prednisolone dose.

vii. The next page will provide the randomisation results (see Figure 17), as follows:
1. **Treatment group**: the prednisolone or control (no prednisolone) group
2. **Dosage of prednisolone** for patient who is allocated to the prednisolone group. This information will not be available for patient who was allocated to the control group.
3. **Registration ID**. This will confirm the Registration ID that the nurse has submitted at the start of the randomisation process
4. **Randomisation ID**. This is a new specific ID for patient that has been randomised.
5. **Study**. This will confirm the name of the study, which is 'OPAL STUDY'.

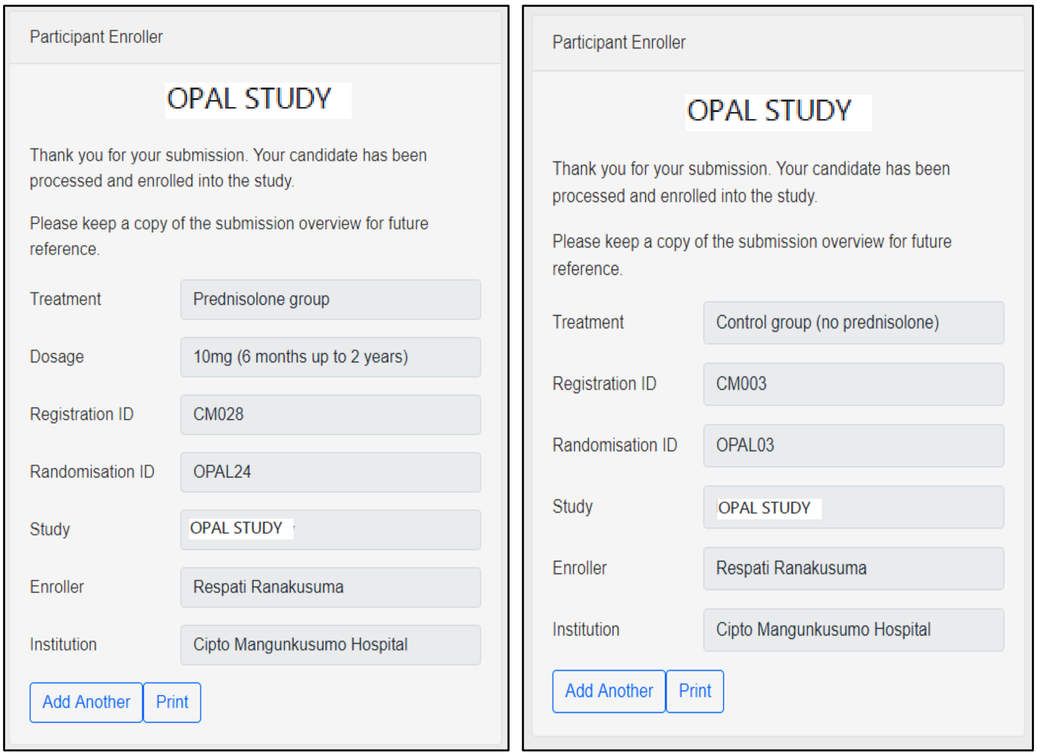


Figure 17. The MASCOT study randomization system – The randomisation result page for the prednisolone and control groups.

viii. The nurse will copy the randomisation result to **CRF08: Randomisation form.** This form will then be separated from the **case report form binder** to the **confidential study document binder**. This information will be concealed from the physicians and audiologists

ix. If the nurse is using a computer that is connected to the printer, then the nurse can directly print the randomisation result page. However, if using smart phone, then the nurse can download the page to be recalled and printed later. The nurse can click **'Add Another'** button if there is another patient to be randomised.

b. By phone:
  i. The attending nurse can contact the principal investigator (+62 8111 012 185) or the research assistant (+82 812 8799 0123) who will access the MASCOT study randomization system website.
  ii. The attending nurse has to complete **CRF08: Randomisation form** and report all information from this form to the research assistant by phone, as follows:
    1. Name of the appointed nurse and the institution
    2. Registration ID

3. All 'YES' for all inclusion criteria
4. All 'NO' for all inclusion criteria
5. Has consent been given?
6. Date of birth and age of the patient
7. Dose of the prednisolone (please confirm with **FORM08: Prednisolone dose for OPAL study** and **CRF07: Prescription of study medication**)
8. Severity of AOM

iii. The research assistant will require five minutes to access the randomisation system website and provide the randomisation allocation results back to the nurse.

iv. The research assistant will call the attending nurse and provide the information, as follows:

1. **Randomisation ID**
2. **Treatment group**: prednisolone or control (no prednisolone) group
3. **Dosage of prednisolone** for patient who is allocated to the prednisolone group. This information will not be available for patient who was allocated to the control group.

v. This information will be recorded at the bottom column of **CRF08: Randomisation form**. This form will then be separated from the **case report form binder** to the **confidential study document binder**. This information will be concealed from the physicians and audiologists.

3. For patients who are allocated to the prednisolone group, the nurse will dispense **CRF07: Prescription of study medication** to the parent(s). The nurse will provide information regarding the use of prednisolone, as follows:
   a. Taking the medication all at once, in the morning before 9 am after breakfast, everyday, for five days as
   b. Mixing the medication with sweetener syrup that has been provided by the study
   c. Mixing the medication with milk, juice, jam, or jelly
   d. Giving back one dose of medication if the child vomits less than 30 minutes after taking the medication. However, if the child vomits after 30 minutes, then the parent should not give another dose.

   The nurse will also provide **FORM09: Instruction of prednisolone use for parents**
4. For every patient, the nurse will confirm whether the parent(s) have already had **CRF06: Symptom diary** and understood how to complete it.
5. The nurse will provide **CRF09: Follow-up visit card** for each patient. The nurse will write scheduled dates for follow-up visits, based on the initial visit.
6. The nurse will ask the parent(s) to complete **CRF02: Study registration form**, if it has not been completed.
7. The nurse will remind the parent(s) to observe the patient and come to the hospital for follow-up visit after 48 hours (day-3) or if the study participant's condition worsens at any time or does not show clinical improvement within 48 hours. The nurse will inform the parent(s) that the parent(s) can contact the 24-hour call centre for any questions or assisstances at anytime.
8. The nurse will also ensure the study participant and the parent(s) will not inform their physician and audiologist regarding the intervention they received in the trial (prednisolone or without prednisolone) until at least the outcome measurement or data collection at Day-3.

9. The nurse will give Rp. 150.000,- (approx. $15) cash for **transportation cost reimbursement** in an envelope to the parent(s).
10. The nurse will provide **study souvenirs** to the patient in each visit, as follows:
    a. Initial visit: Lunch box
    b. First visit (Day-3): Water bottle
    c. Second visit (Day-7): Toys
    d. Third visit (Day-30): Mini towel
    e. Fourth visit (Day-90): Study bag
11. Before sending the patient home, the nurse will check the completeness of all study documents from the binders:
    a. Case report form:
        i. CRF01: Consent form
        ii. CF02: Study registration form
        iii. CRF03: Eligibility form
        iv. CRF04: Baseline information form
        v. CRF05: Outcome form
        vi. CRF08: Randomisation form
        vii. CRF10: Serious adverse events reporting form
        viii. CRF11: Feedback form
    b. Confidential study document binder:
        i. FORM01: Study recruitment log book, including copy the randomisation ID to the form, and reasons if patient has not been recruited to the study.
12. The nurse will apply a sticker on the patient medical record as an identification that the patient is in the OPAL study (see Figure 18), as well as on the page of consultation page in the medical record (see Figure 19).

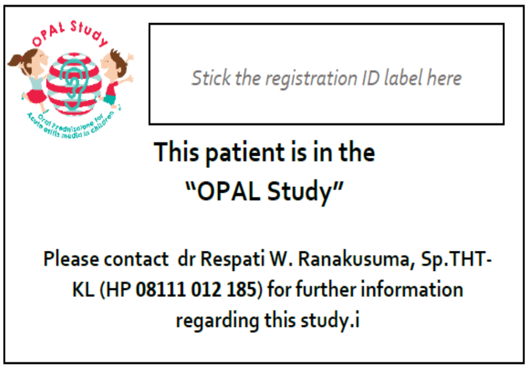


Figure 18. The OPAL study identification sticker for the cover of medical record.

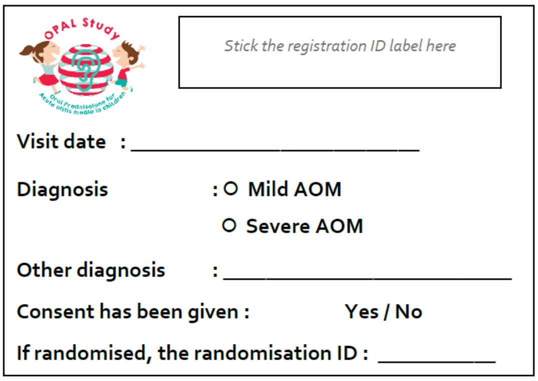


Figure 19. The OPAL study identification sticker for the medical record page.

13. When the process is over, the patient has left the room, and all documents are well completed, the nurse will then separate the forms into the binders:
    **a. Case report form:**
        i. CRF03. Eligibility form
        ii. CRF04. Baseline information form
        iii. CRF05. Outcome form
        iv. CRF10. Serious adverse events reporting form
        v. CRF11. Feedback form
    **b. Confidential study document binder:**

i. FORM01. Study recruitment log book
ii. CRF01. Consent form
iii. CRF02. Study registration form
iv. CRF08. Randomisation form

c. **Completed case report form and non-participating subject binder:**
i. FORM06. Non-participating subject form (e.g. not-eligible, not-consented individual forms).

14. The nurse will then secure all documents and binders in the locked cabinets.

# Preparing and dispensing the study medication

## Objective(s)

1. Prepare the prednisolone tablets in form of powder
2. Dispense the study medication along with the instruction for its use
3. Record the dispensing using the **FORM03: Study medication dispensing form**

## Tool(s)

1. CRF07: Prescription of study medication
2. Prednisolone tablets
3. Sweetener syrup
4. FORM03: Study medication dispensing form
5. FORM10: Lupred pharmaceutical brochure

## Personnel

1. Pharmacist

## Procedures

1. The pharmacist will receive **CRF07: Prescription of study medication** from the parents and prepare the study medication according to the prescribed dose. Prior to this, the pharmacist will recheck the dose using the prednisolone dose guideline attached on the top of the prescription.
2. The pharmacist will then prepare the prednisolone by crushing the prednisolone tablets, mixing them with sweeteners, and packing them in five daily paper-packs.
3. The pharmacist will dispense the study medication with the instruction, as follows:
    a. give the medicine in the morning, after breakfast and in the morning (before 9 am), everyday for five days
    b. mix the medicine with sweetener syrup of a ratio of 1:3
    c. give the medicine with a glass of water, milk, or juice, or mix it with a small amount of soft food such as jam, or yoghurt
    d. give the medication all at once
    e. if the child vomits in less than 30 minutes after taking the study medication, then the parent should give another dose.

    The pharmacist will also provide **FORM10. Lupred pharmaceutical brochure for the parents**
4. The pharmacist will complete **FORM03: Study medication dispensing form** by recording the registration ID, the study medication dose, and date dispensed for the study medication

# Returning the study medication

## Objective(s)

1. Identify the adherence to the study medication by collecting the left-over medication
1. Record the study medication return using **FORM04. Study medication return form**

## Tool(s)

2. FORM04. Study medication return form

## Personnel

1. The appointed nurse

## Procedures

1. At Day-7, the appointed nurse will collect the left-over study medication, including the paper-wrap, and record this on **FORM04: Study medication return form** (see Figure 20)**.**

dr. Respati W. Ranakusuma, SpTHT-KL
Clinical Epidemiology & Evidence-Based Medicine (CEEBM) Unit, Dr. Cipto Mangunkusumo Hospital – Faculty of Medicine Universitas Indonesia
Oral prednisolone for acute otitis media in children: a pilot pragmatic, randomised, open-label, single-blind, controlled study (OPAL Study)

**Nurse ID** : |___|___|___|___| **Hospital ID** : |___|___|___|

| FORM04 – STUDY MEDICATION RETURN FORM (FOR NURSE) | | | |
|---|---|---|---|
| Randomisation/ Registration ID | Date returned | Number of left-over drug | Reason for return |
| | | | |
| | | | |

Figure 20. FORM04: Study medication return form (for nurse).

# Follow-up Visits

Each study participant will have additional four follow-up visits after the baseline visit (Visit-0), as follows (see **Appendix 1. Protocol, section 'Participant timeline', page 19**):

1. First visit (Visit-1) on day-3 (48 hours after the baseline visit)
2. Second visit (Visit-2) on day-7 after the completion of 5-day cycle of study medication
3. Third visit (Visit-3) on day-30 or month-1
4. Fourth visit (Visit-4) on day-90 or month-3

Using the information of parents' mobile phone numbers provided on **CRF02: Study registration form**, a research assistant will send a daily text message as a reminder: (1) to remind the parents to give the prednisolone (for study participant in the prednisolone group) every morning for five days, (2) to visit the hospital at Day-3 and Day-7, and (3) to complete the SYMPTOM DIARY for two weeks. For the third and fourth visits, the research coordinator will send reminder text messages one week and one day before the follow-up dates (see **Appendix 1. Protocol, section 'Adherence monitoring', page 17**).

## First follow-up visit (Day–3)

The study participant and the parent(s) will have the first visit after 48 hours observation or on the third day after the baseline visit. If they miss the follow-up visit at Day-3, they can still come until the next two days (Day-4 to Day-5) after the scheduled date.

The study participant will come to the nurse station. The attending nurse will:

1. Identify the patient as a study participant by the OPAL study sticker on the front page of medical record.
2. Measure and record the weight, height, body temperature, and blood pressure
3. Report and send the study participant to the appointed nurse.

The appointed nurse will:

1. Use the registration ID label on the OPAL study sticker to find that particular **case report form binder** from the filing cabinet.
2. Check the completeness of the case report forms in the binder
3. Obtain information from the parents regarding the symptom of AOM, any complications (i.e. mastoiditis, perforation of tympanic membrane, and the adherence on the study medication use, as well as identify side effects, and complications of acute otitis media) from the first mini-booklet of the symptom diary.
4. Check the completeness and collect **the first mini booklet of CRF06: Symptom diary**. The nurse will confirm the symptoms, complication, or any side effects reported in the symptom diary. The nurse will place the **first mini booklet of symptom diary** back to the **case report form binder**. The nurse will remind the parent to continue completing the second mini booklet of symptom diary. the parent should complete the symptom diary in the next morning before administering any medicine to their children.
5. Check whether the study participant still has a sufficient number of study medication for the next two days.
6. Report the study participant to any participating physician on duty that day, along with the **case report from binder.**

7. After the study participant finish the consultation and data collection with the participating physician, the study participant again will meet the appointed nurse. The appointed nurse will check the completeness of all study documents in the **case report form binder**.
8. record the visit date on **CRF09: Follow-up visit card**
9. remind the parent to take the study medication regularly, complete the second mini booklet of the symptom diary, and to come for the next follow-up visit (Visit-2) at the Day 7.
10. hand over a study souvenir (lunch box) and transport cost reimbursement envelope for each study participant.

The participating physician will:

1. Identify any complications of AOM and perform ENT, otoscopic, and tympanometry examination.
2. Identify the severity of AOM symptoms using VAS and AOM-SOS on **CRF05: Outcome form.**
3. assess whether there is a sufficient improvement of symptoms over the past 48 hours. If there is no improvement or worsening of the symptoms of acute otitis media, based on the clinical judgement, the physician may prescribe antibiotics for the study participant in the mild AOM group or change the antibiotics for the study participant in the severe group. This will be recorded in **CRF05: Outcome form.**
4. Identify any side effects. If it is necessary to determine the treatment for the side effects, the physician can retrieve the information from the nurse whether the study participant was allocated to prednisolone or control group. However, this must be done after the physician complete assessing and recording all outcome data in **CRF05: Outcome form.**
5. prescribe the study medication accordingly, if the study participant require more medication.
6. send the participant back to the appointed nurse.

# Second follow-up visit (Day–7)

The process is similar to Visit–1. If the study participants and the parents miss the follow-up visit at Day-7, they can still come until the next two days (Day-8 to Day-9) after the scheduled date. The study participant will come to the nurse station, where the attending nurse will identify a patient as a study participant by the OPAL study sticker on the front page of the medical record. The nurse will measure the weight, height, body temperature, and blood pressure of the study participant. The nurse will then report this to the appointed nurse. Using the registration ID number attached on the OPAL study sticker on the front page of the medical record, the attending nurse will collect that particular subject's **case report form binder**.

The appointed nurse will:

1. Identify any adverse effects or complications since the last visit
2. Check the completeness and collect **the second mini booklet of CRF06: Symptom diary**
3. Remind the parents to complete **the third mini booklet of CRF06: Symptom diary** on the next morning until Day-14. The nurse will inform the parents that at Day-14, a research staff will visit their home to collect the diary. The research staff will also obtain feedback from the parents regarding their experience and obstacles during the past 14 days of the study, using **CRF11: Feedback form.**
4. Collect the left-over study medication from those in the prednisolone group. This will be recorded in **FORM04: Study medication return form.** The appointed nurse will complete

the study registration ID, returning date, and numbers of left-over study medication. She can add some comment regarding the use of prednisolone if necessary.

5. Report the study participant to any participating physician on duty that day, along with the **case report from binder**.
6. After the study participant finish the consultation and data collection with the participating physician, the study participant again will meet the appointed nurse. The appointed nurse will check the completeness of all study documents in the Case report form binder.
7. record the visit date on **CRF09: Follow-up visit card**
8. remind the parent to come for the next follow-up visit (Visit-3) at the Day 30.
9. hand over a study souvenir (toys) and transport cost reimbursement envelope for each study participant.

The participating physician will:

1. Identify any complications of AOM and perform ENT, otoscopic, and tympanometry examination.
2. Identify the severity of AOM symptoms using VAS and AOM-SOS on **CRF05: Outcome form.**
3. Identify any side effects. If it is necessary to determine the treatment for the side effects, the physician can retrieve the information from the nurse whether the study participant was allocated to prednisolone or control group.
4. Send the study participant back to the appointed nurse.

# Third follow-up visit (Day–30)

At the third visit, the patient will come on Day–30. If the study participant misses the scheduled follow-up visit, then the study participant can come on any other day up to one week after the scheduled date. Similar to previous visits, the nurse will identify the study participant using the OPAL study sticker attached on the front page of the medical record and perform a general examination. The attending nurse will report this subject to the appointed nurse. The appointed nurse will collect and prepare the **case report form binder**, and bring the study participant with the binder to the participating physician on duty.

The physician will identify whether in the past one month, the study participant experiences a new episode of ear pain or any other symptoms related to acute otitis media. The physician will also conduct a nose and throat examination, as well as an ear examination using otoscope. This information will be recorded in **CRF05: Outcome form**. The study participant will then undergo a tympanometry examination and will go back to the consultation room for the assessment of the tympanometry result. At the end of the visit, the physician will hand over the completed study documents in the **case report form binder** back to the appointed nurse where she will check the completeness of the forms and store it back in the locked filing cabinet.

# Fourth follow-up visit (Day–90)

The process is similar to Visit–3. At the end of the visit, the physician will hand over the completed study documents in the **case report form binder** to the appointed nurse where the nurse will then check the completeness of the forms. The appointed nurse will collect al the study documents form the case report form binder and clip them together, and separate the document to a plastic sleeve in the **completed case report form and non-participating subject binder.** The nurse will then record

the randomisation ID, date enrolled to the study, whether the study participant come to all follow-up visits, and date of completion of the study.

# Assessing Adverse Events

The appointed nurse will be the first person who identifies adverse events on the first visit at Day-3 (48 hours after the baseline visit). The adverse events will be identified by obtaining information of any unfavourable effects after taking the study medication in forms of: (1) interview and (2) the assessment of **the completed first mini-booklet of the symptom diary** (see **Appendix 1. Protocol, section 'Harms', page 25-26**).

The nurse will report any adverse events to the physician without acknowledging the study intervention that the patient has received. However, if it necessary to determine the treatment for the adverse events, the physician may have the information of intervention allocation. The physician will check other medications that have been prescribed (e.g. antibiotics, decongestants, mucolytic) at the baseline visit and obtain more detailed information regarding the adverse events to identify its correlation with trial drug or other concomitant drugs. The physician will then record this on **CRF05: Outcome form**.

The physician will record severe adverse event(S) on **CRF10: Serious adverse events reporting form** and inform this to the chief investigator (CI). The physician will report any adverse events that cause the modification or discontinuation on study medication to the CI. The decision to discontinue study medication will be determined by the physician based on the clinical judgment and the CI based on perspective of good clinical practice (GCP). If the patient requires other tests and/ treatments, the physician will provide these services. All the costs will be reimbursed by the study. However, this must be reported and recorded on **CRF05: Outcome form**.

# Feedback Form

As this study is a pilot study, we will obtain information from all individuals involved in the study (i.e. participating physicians, appointed nurses, audiologists, pharmacists, study participants, and the parents) to identify their experience and obstacles that they encountered during the study.

For the participating physicians, audiologists, the appointed nurses, and pharmacists, we will obtain their feedback using **CRF11: Feedback form** in an interview session. We will interview them after they have enrolled at least five children into the study.

We will obtain the feedback from the parents at Day-14 during the home visit. The home visit will be conducted to collect the **third mini-booklet of the symptom diary**, where we will also interview the parents to obtain the feedback using **CRF11: Feedback form**.

# Closing Out The Study

When closing out the study, the research team will check several documents to validate the compatibility and the completeness of the data:

1. **FORM01: Study recruitment log book**, will be checked for its compatibility with **CRF01: Consent form; CRF03: Eligibility form;** and **CRF08: Randomisation form** in the **case report form binder** of each study participant.
2. The completeness and the date of follow-up visit in **CRF05: Outcome form**, will be checked for its compatibility with the visit dates on **CRF06: Symptom diary** and **CRF09: Follow-up visit card**.
3. The number of **CRF11: Feedback form will** be checked for its compatibility with the number of participating physicians, audiologists, appointed nurses, pharmacies, and the parents who are involved in the study

In regards to study medication, the research team will check the numbers of unused medication at the pharmacy and the left-over medicine on the parents, and compare the numbers with **FORM03: Study medication dispensing form; FORM04: Study medication return form;** and the number of used paper-packs accordingly.

In regards to administrative work, the research team will:

1. Collect the left-over envelopes for the reimbursement of patients' transportation cost and check the compatibility with the numbers of follow-up visits recorded in **CRF09: Follow-up visit card.**
2. Confirm the hospital cashier regarding the study-related payment (e.g. registration fee, tympanometry examination, any additional test or treatment due to adverse events) according approved Memorandum of Agreement (MoU) with the numbers of follow-up visits recorded in **CRF09: Follow-up visit card** and **CRF05: Outcome form** for any additional tests or treatments for side effects.
3. Confirm the inventory list with the starting kit container.

At the end of study, as part of the dissemination of the study results, the research team will:

1. Provide a study summary that will include the examination results of individual study participants and the overall result of the study to each participant.
2. Provide a report document of the study to the Bond University's Human Research Ethics Committee, the Research Committee Ethics Faculty of Medicine Universitas Indonesia (FMUI) – Dr. Cipto Mangunkusumo Hospital (CMH), and all participating hospitals.

# Oral Prednisolone for Acute otitis media in chiLdren:

## A pilot pragmatic, randomised, open-label, single-blind, controlled study (OPAL Study)


**Centre for Research in Evidence-Based Practice**
Faculty of Health Sciences and Medicine Bond University
Queensland, Australia

**Clinical Epidemiology and Evidence-Based Medicine**
Dr Cipto Mangunkusumo Hospital
Faculty of Medicine Universitas Indonesia
Jakarta, Indonesia

# Administrative information

## Protocol title

**O**ral **P**rednisolone for **A**cute otitis media in chi**L**dren: a pilot pragmatic randomised open-label single-blind study (**OPAL study**)

## Authors

Respati W. Ranakusuma, MD, ORL

Dr. Amanda McCullough, PhD, PGCHET, BSc (Hons)

Associate Professor Elaine Beller, BSc, MAppStat

Professor Christopher Del Mar, FAFPHM, MA, MD, FRACGP, BSc

Professor Dr. Sudigdo Sastroasmoro, MD, PhD, Paed.

Eka Dian Safitri, MD, ORL

Yupitri Pitoyo, MD, ORL

Widyaningsih, MPH

Arie Sulistyowati, MD, MSc, Paed.

# Table of Contents

**Administrative information ... 1**
Protocol title ... 1
Authors ... 1
Trial registration ... 4
The World Health Organisation Trial Registration Data set ... 4
Protocol version ... 6
Funding ... 7
Roles and responsibilities ... 7
Protocol contributions ... 7
Contact information of trial sponsor or funder(s) ... 8
Contact information of research team and others overseeing the trial ... 8
**CHAPTER 1 – INTRODUCTION ... 10**
1.1 Background and rationale ... 10
1.1.1 Background ... 10
1.1.2 Rationale for the proposed study ... 10
1.1.3 Rationale for pilot study ... 13
1.2 Objectives ... 14
**CHAPTER 2 – METHODS ... 15**
2.1 Trial design ... 15
2.2 Participants, interventions, and outcomes ... 15
2.2.1 Study setting ... 15
2.2.2 Eligibility criteria ... 15
2.2.3 Interventions ... 16
2.2.4 Outcomes ... 17
2.2.5 Participant timeline ... 19
2.1.6 Sample size ... 21
2.1.7 Recruitment ... 21
2.3 Assignment of interventions ... 21
2.3.1 Allocation ... 21
2.3.2 Blinding (masking) ... 22
2.4 Data collection, management, and analysis ... 22
2.4.1 Data collection methods ... 22
2.4.2 Data management ... 24
2.4.3 Statistical methods ... 24
2.5 Monitoring ... 25
2.5.1 Data monitoring ... 25
2.5.2 Harms ... 25
2.5.3 Auditing ... 26
2.6 Ethics and dissemination ... 26
2.6.1 Research ethics approval ... 26
2.6.2 Protocol amendments ... 26

2.6.3 Consent ............ 26
2.6.4 Confidentiality ............ 27
2.6.5 Declaration of interests ............ 27
2.6.7 Ancillary and post-trial care ............ 28
2.6.8 Dissemination policy ............ 28

**APPENDICES ............ 29**

**REFERENCES ............ 30**

## Trial registration

Registry name: http://www.ANZCTR.org.au/ACTRN12618000049279.aspx

## The World Health Organisation Trial Registration Data set

| | |
|---|---|
| Primary Registry and Trial Identifying Number | ACTRN12618000049279 |
| Date of Registration in Primary Registry | 16 January 2018 |
| Secondary Identifying Numbers | - |
| Source(s) of Monetary or Material Support | Self-funded research |
| Primary Sponsor | Respati W. Ranakusuma, MD, ORL |
| Secondary Sponsor(s) | None |
| Contact for Public Queries | Respati W. Ranakusuma, MD, ORL<br>Centre for Research in Evidence-Based Practice<br>Faculty of Health Sciences and Medicine Bond University, QLD, Australia<br>14 University Drive, Robina 4226, Queensland<br>Phone number: +61424957129 (Australia) / +6228111012185 (Indonesia)<br>Email: rranakus@bond.edu.au |
| Contact for Scientific Queries | Respati W. Ranakusuma, MD, ORL<br>Email: rranakus@bond.edu.au<br>Phone number: +61424957129 (Australia) / +6228111012185 (Indonesia)<br>Centre for Research in Evidence-Based Practice Faculty of Health Sciences and Medicine Bond University, QLD, Australia<br>14 University Drive, Robina 4226, Queensland<br>Email: OPAL.study@bond.edu.au<br>Phone number: (+61) 7 559 51588 |
| Public Title | Oral prednisolone for acute middle ear infection in children |
| Scientific Title | Oral prednisolone for acute otitis media in children: a pilot pragmatic randomised open-label single-blind study (OPAL study) |
| Countries of recruitment | Indonesia |
| Health Condition(s) or Problem(s) Studied | Acute otitis media in children |
| Intervention(s) | Intervention Name:<br>• Active intervention: Prednisolone tablet<br>• Active comparator: None<br>Intervention Description:<br>• Prednisolone tablet with doses based on range of age for five days:<br>○ 6 months – <2 years: 10 mg/day<br>○ 2 – <6 years: 20 mg/day<br>○ 6 – 12 years: 30 mg/day<br>• None |
| Key Inclusion and Exclusion Criteria | Ages eligible for study: 6 months to 12 years<br>Sexes eligible for study: both males and females<br>Accepts healthy volunteers: no<br>Inclusion criteria:<br>• children (6 months – 12 years) with acute otitis media, defined as a current onset within 48 hours of ear-related symptoms (e.g. ear pain, ear tugging/rubbing or irritability) and if possible to assess, otoscopic findings of acute inflammation (e.g. erythema) and middle ear effusion (e.g. bulging, air-fluid level)<br><br>Exclusion criteria:<br>• children with major and severe medical conditions (e.g. heart failure, kidney failure)<br>• immunocompromised children (e.g. HIV, children receiving cancer treatment)<br>• children with congenital malformations and/or syndromes (e.g. cleft palate, Down's syndrome)<br>• children with high risk of risk of strongyloidiasis infection<br>• children with ear ventilation tube(s) |

| | |
|---|---|
| | • children who had exposed to persons with varicella (chicken pox) or active Zoster infection in the past 3 weeks without any prior varicella immunisation or infection<br>• children who have taken systemic (i.e. oral, injection) or topical steroids in the preceding four weeks<br>• children who have taken antibiotics in the preceding two weeks; a<br>• children who are hypersensitive to prednisolone or prednisone, or other corticosteroids. |
| Study Type | Type of study: interventional<br>Method of allocation: stratified, randomised<br>Masking: open-label, single-blind (outcome assessor)<br>Assignment: parallel<br>Purpose: Efficacy<br>Phase III |
| Date of first enrolment | 01 January 2018 |
| Target sample size | 60 children |
| Recruitment status | Pending (not started): participants are not yet being recruited or enrolled at any site |
| Outcome(s) | (1) Outcome Name: Recruitment rates<br>Metric/method of measurement: Informed consent form and case report forms (CRFs)<br>The timepoint(s) of interest: Baseline visit (visit-0)<br>(2) Outcome Name: The success of the study procedures<br>Metric/method of measurement: CRFs (i.e. eligibility and randomisation form, outcomes form)<br>The timepoint(s) of interest: Baseline visit (visit-0)<br>(3) Outcome Name: Ability to measure planned outcomes in main study<br>Metric/method of measurement: CRFs (i.e. eligibility and randomisation form, outcomes form) and symptom diary (e.g. Visual Analogue Scale/VAS and Acute Otitis Media – Severity of Symptoms Scale/AOM-SOS), and feedback form<br>The timepoint(s) of interest: Baseline visit, visit-1 (day-3 to-5), visit-2 (day-7 to -9), day3, visit-3 (day-3 to -40), and visit-4 (day-90 to -100)<br>(4) Outcome Name: Compliance to study and study drug<br>Metric/method of measurement: CRF (i.e. outcomes form), the symptom diary, and number of left-over drug<br>The timepoint(s) of interest: Visit-1 (day-3 to-5), visit-2 (day-7 to -9), day3, visit-3 (day-3 to -40), and visit-4 (day-90 to -100)<br>(5) Outcome Name: The verification of sample size calculation for main study<br>Metric/method of measurement: CRFs (i.e. eligibility and randomisation form)<br>The timepoint(s) of interest: Baseline visit (visit-0) |

## Protocol version

| Protocol version No. | Date issued |
|---|---|
| Protocol OPAL Study Version 1.0.0 (V1.0.0) | 27 July 2017 |
| Protocol OPAL Study Version 1.1.0 (V1.1.0) | 17 October 2017 |

**Protocol Amendment Number:** AM1.0

**Amendment history:**

| Amendment No. | Protocol version no. | Date issued | Author(s) of changes | Detail of changes made |
|---|---|---|---|---|
| Amendment No. 1.0 | Protocol V1.1.0 | 17 October 2017 | Respati W Ranakusuma | 1. The modification of the form of trial drug<br>We planned to use prednisolone liquid. Due to administrative issues where the proposed pharmaceutical manufacturer was unable to share confidential documents that were required for importing the trial drug to Indonesia, we therefore will use prednisolone tablet for this trial. The pharmacist will crush the prednisolone tablets and mix it with sweetener. The crushed tablet will be packed in daily paper-package. The parents will mix the crushed tablet with juice or honey. This method is commonly practiced in Indonesia, particularly for paediatric patients.<br>2. The duration of the trial drug use<br>We planned to give prednisolone for seven days. Although 7-day duration of corticosteroid use is considered as a short-term use, we will reduce its duration to five days to minimise the potential harms caused by corticosteroids. |

## Funding

This research is supported by self-funding of the principal investigator (Dr. Respati W. Ranakusuma, ORL).

We will purchase the trial drug, prednisolone tablets, from PT Pratapa Nirmala, Tangerang, Indonesia. This pharmaceutical manufacturer is not linked to this study and does not have authority over any procedural implementation, scientific process, or decision in the study.

## Roles and responsibilities

### Protocol contributions

| Name | Affiliation | Role of protocol contributors |
|---|---|---|
| **Dr. Respati W. Ranakusuma, ORL**<br>ENT surgeon, PhD candidate<br>Telp: +61 424 957 129 / +62 8111 012 185<br>Fax: N/A<br>Email: rranakus@bond.edu.au / respati.ranakusuma@ceebm.org | • Centre for Research in Evidence-Based Practice (CREBP) Faculty of Health Sciences and Medicine, Bond University 14 University Drive, Robina QLD 4226 Australia<br>• Clinical Epidemiology and Evidence-Based Medicine Unit, Dr. Cipto Mangunkusumo Hospital – Faculty of Medicine Universitas Indonesia Jl. Diponegoro 71 Jakarta 10430 Indonesia | Initiated the study design and methodology; developed the protocol including case report forms (CRFs), informed consent, and symptom diary |
| **Dr. Amanda McCullough, PhD, PGCHET, BSc (Hons)**<br>Epidemiologist, expert in acute respiratory infections and antibiotic resistance<br>Telp: +61 7 559 55204<br>Fax: N/A<br>Email: amccullo@bond.edu.au | Centre for Research in Evidence-Based Practice (CREBP) Faculty of Health Sciences and Medicine, Bond University, 14 University Drive, Robina QLD 4226 Australia | Initiated the study design from the clinical epidemiologic perspective; refined the protocol including case report forms (CRFs), informed consents, and symptom diary |
| **Associate Professor Elaine Beller, BSc, MAppStat**<br>Clinical trialist, biostatistician<br>Telp: +61 7 559 55523<br>Fax: N/A<br>Email: ebeller@bond.edu.au | Centre for Research in Evidence-Based Practice (CREBP) Faculty of Health Sciences and Medicine, Bond University, 14 University Drive, Robina QLD 4226 Australia | Initiated the study design and methodology from the clinical trialist and biostatistician perspectives; refined the protocol including case report forms (CRFs), informed consents, and symptom diary |
| **Professor Christopher Del Mar, FAFPHM, MBBChir, MA, MD, FRACGP, BSc**<br>Epidemiologist, evidence-based practitioner, expert in acute respiratory infection and antibiotic resistance<br>Telp: +61 7 559 52504<br>Fax: N/A<br>Email: cdelmar@bond.edu.au | Centre for Research in Evidence-Based Practice (CREBP) Faculty of Health Sciences and Medicine, Bond University, 14 University Drive, Robina QLD 4226 Australia | Initiated the study design from the clinical perspective; refined the protocol including case report forms (CRFs), informed consents, and symptom diary |
| **Professor Dr. Sudigdo Sastroasmoro, PhD, Paed**<br>Paediatrician, epidemiologist, evidence-based practitioner<br>Telp: +62 21 316 1760<br>Fax: N/A<br>Email: sudigdo1947@gmail.com | • Clinical Epidemiology and Evidence-Based Medicine Unit, Dr. Cipto Mangunkusumo Hospital – Faculty of Medicine Universitas Indonesia Jl. Diponegoro 71 Jakarta 10430 Indonesia<br>• Department of Child Health Faculty of Medicine Unit, Universitas Indonesia – Dr. Cipto Mangunkusumo Hospital DIponegoro 71 Jakarta 10430 Indonesia | Provided advice in paediatric perspectives, supervising the trial in Jakarta |

## Contact information of trial sponsor or funder(s)

| Name | Contact information | Role of study sponsor and funders |
|---|---|---|
| **Dr. Respati W. Ranakusuma, ORL** | • Centre for Research in Evidence-Based Practice (CREBP) Faculty of Health Sciences and Medicine, Bond University 14 University Drive, Robina QLD 4226 Australia<br>• Clinical Epidemiology and Evidence-Based Medicine Unit, Dr. Cipto Mangunkusumo Hospital – Faculty of Medicine Universitas Indonesia Jl. Diponegoro 71 Jakarta 10430, Indonesia | She is the principal investigator |

## Contact information of research team and others overseeing the trial

| Name | Affiliation | Roles and responsibilities |
|---|---|---|
| **Associate Investigators** | | |
| **Dr. Eka Dian Safitri, ORL**<br>ENT surgeon<br>Telp: +62 21 316 1760<br>Fax: N/A<br>Email: ekadian.safitri@ceebm.org | Clinical Epidemiology and Evidence-Based Medicine Unit, Dr. Cipto Mangunkusumo Hospital – Faculty of Medicine Universitas Indonesia<br>Jl. Diponegoro 71 Jakarta 10430 – Indonesia | (1) involved in the development of tympanometry study; (2) providing training related to using and interpreting tympanometry to physicians, nurses, and tympanometry technicians |
| **Dr. Yupitri Pitoyo, ORL**<br>ENT surgeon<br>Telp: +62 21 316 1760<br>Fax: N/A<br>Email: yupitri.pitoyo@ceebm.org | Clinical Epidemiology and Evidence-Based Medicine Unit, Dr. Cipto Mangunkusumo Hospital – Faculty of Medicine Universitas Indonesia<br>Jl. Diponegoro 71 Jakarta 10430 – Indonesia | Recruiting secondary and tertiary healthcare centres including the physicians and nurses |
| **Dr. Arie Sulistyowati, MSc, Paed.**<br>Paediatrician, epidemiologist<br>Telp: +62 21 316 1760<br>Fax: N/A<br>Email: arie.sulistyowati@ceebm.org | Clinical Epidemiology and Evidence-Based Medicine Unit, Dr. Cipto Mangunkusumo Hospital – Faculty of Medicine Universitas Indonesia<br>Jl. Diponegoro 71 Jakarta 10430 – Indonesia | Providing advice and expertise in terms of paediatric patients |
| **Widyaningsih, MPH**<br>Public Health, qualitative study expert<br>Telp: +62 21 316 1760<br>Fax: N/A<br>Email: widyaningsih.ade@ceebm.org | Clinical Epidemiology and Evidence-Based Medicine Unit, Dr. Cipto Mangunkusumo Hospital – Faculty of Medicine Universitas Indonesia<br>Jl. Diponegoro 71 Jakarta 10430 – Indonesia | (1) submitting a research ethics application to the Indonesian Medical Research Ethics Committee; (2) recruiting primary healthcare centres including the physicians and nurses |
| **Research Coordinator** | | |
| **Dr. Respati W. Ranakusuma, ORL**<br>ENT surgeon, PhD candidate<br>Telp: +62 8111 012 185<br>Fax: N/A<br>Email: rranakus@bond.edu.au / respati.ranakusuma@ceebm.org | Clinical Epidemiology and Evidence-Based Medicine Unit, Dr. Cipto Mangunkusumo Hospital – Faculty of Medicine Universitas Indonesia<br>Jl. Diponegoro 71 Jakarta 10430 – Indonesia | |
| **Co-investigators** | | |
| **Dr. Tri Juda Airlangga H, ORL**<br>ENT surgeon<br>Telp: +6221 1500 135<br>Fax: N/A<br>Email: airlanggamd@gmail.com | Department of Ear, Nose, and Throat Head and Neck Surgery<br>Dr. Cipto Mangunkusumo Hospital – Faculty of Medicine Universitas Indonesia<br>Jl. Diponegoro 71<br>Central Jakarta 10430 – Indonesia | (1) Recruiting patients<br>(2) Coordinating patient recruitment among other clinicians in the hospital |
| **Dr. Yulvina, ORL**<br>ENT surgeon<br>Telp: +6221 470 1133<br>Fax: N/A<br>Email: yulie_dj@yahoo.com | Department of Ear, Nose, and Throat Head and Neck Surgery<br>Persahabatan General Hospital<br>Jl. Persahabatan Raya 1<br>East Jakarta 13230 – Indonesia | (1) Recruiting patients<br>(2) Coordinating patient recruitment among other clinicians in the hospital |

| | | |
|---|---|---|
| **Dr. Evita Fitria Edyani, ORL**<br>ENT surgeon<br>Telp: +6221 344 1008<br>Fax: N/A<br>Email: evitafitria@yahoo.com | Department of Ear, Nose, and Throat Head and Neck Surgery<br>Gatot Subroto Army Hospital<br>Jl. Dr Abdul Rahman Saleh 24<br>Central Jakarta 10410 – Indonesia | (1) Recruiting patients<br>(2) Coordinating patient recruitment among other clinicians in the hospital |
| **Dr. Yupitri Pitoyo, ORL**<br>ENT surgeon<br>Telp: +6221 884 2121<br>Fax: N/A<br>Email: yupitri.pitoyo@ceebm.org | Department of Ear, Nose, and Throat Head and Neck Surgery<br>Hermina Bekasi Hospital<br>Jl. Kemakmuran 39, South Bekasi<br>West Java 17141 – Indonesia | (1) Recruiting patients<br>(2) Coordinating patient recruitment among other clinicians in the hospital |
| **Dr. Respati W. Ranakusuma, ORL**<br>ENT surgeon<br>Telp: +6221 2937 8939<br>Fax: N/A<br>Email: rranakus@bond.edu.au | Department of Ear, Nose, and Throat Head and Neck Surgery<br>Antam Medika Hospital<br>Jl. Pemuda 1A<br>East Jakarta 13210 – Indonesia | (1) Recruiting patients<br>(2) Coordinating patient recruitment among other clinicians in the hospital |
| **Dr. Eka Dian Safitri, ORL**<br>ENT surgeon<br>Telp: +6221 425 0451<br>Fax: N/A<br>Email: ekadian.safitri@ceebm.org | Department of Ear, Nose, and Throat Head and Neck Surgery<br>Jl. Cempaka Putih Tengah I/1<br>Central Jakarta 10510 – Indonesia | (1) Recruiting patients<br>(2) Coordinating patient recruitment among other clinicians in the hospital |
| **Dr. Hably Warganegara, ORL**<br>ENT surgeon<br>Telp: +6221 390 0002<br>Fax: N/A<br>Email: hablywarganegara@gmail.com | Proklamasi Ear, Nose, Throat (ENT) Centre<br>Jl. Proklamasi 43<br>Central Jakarta 10230 – Indonesia | (1) Recruiting patients<br>(2) Coordinating patient recruitment among other clinicians in the hospital |
| **Data Manager** | | |
| **Respati W. Ranakusuma, MD, ORL**<br>ENT surgeon, PhD candidate<br>Telp: +61 424 957 129 / +62 8111 012 185<br>Fax: N/A<br>Email: rranakus@bond.edu.au / respati.ranakusuma@ceebm.org | Clinical Epidemiology and Evidence-Based Medicine Unit, Dr. Cipto Mangunkusumo Hospital – Faculty of Medicine Universitas Indonesia<br>Jl. Diponegoro 71 Jakarta 10430<br>Indonesia | |

# CHAPTER 1 – INTRODUCTION

## 1.1 Background and rationale

### 1.1.1 Background

Antibiotic resistance, a major global threat, impacts more than two million people with illness and accounts for 23 thousand deaths annually in the United States [1]. One of the key drivers of the development of antibiotic resistance is antibiotic prescribing [2]. Antibiotics are mostly prescribed for common diseases, such as acute respiratory infections (ARIs) [3]. One of the ARIs commonly found in paediatric population with high antibiotic prescribing is acute otitis media [4,5]. In East Jakarta, Indonesia, the prevalence of AOM in children (< 18 years) was 5.4% [6]. In contrast, in Australia, there were an average of 35 new AOM cases reported by general practitioners per year (April 2010 – March 2015) [7].

Acute otitis media (AOM) is characterised by rapid onset of symptoms (e.g. earache, ear tugging/rubbing, irritability), middle ear effusion (e.g. bulging, immobile tympanic membrane, air fluid level), and acute inflammation (e.g. erythema) [8]. Almost three quarters of children have an episode of AOM in their first five years of life, with the peak incidence at the age of six to 12 months [9,10]. Guidelines recommend close monitoring for 48 hours (expectant observation) along with adequate pain management for mild AOM (e.g. mild symptoms, fever < 39°C) [8]. Children with severe symptoms, young age (< 2 years) with bilateral AOM, and AOM with tympanic membrane perforation are more likely to benefit from antibiotic treatment [11]. A high rate of antibiotic prescription for AOM is evident. Eighty-nine per cent new AOM cases were managed by antibiotics in Australia during 2010 to 2015 [7]. In Indonesia, a survey study using clinical scenarios, demonstrated that up to 88% of physicians would prescribe antibiotics for mild case of AOM. Unclear clarification of antibiotic use in the Indonesian practice guideline for AOM in the primary care may contribute to this [12]. The option of using antibiotics also must be balanced against the risks, such as adverse effects (e.g. vomiting, diarrhoea, rash) and antibiotic resistance [13,14].

### 1.1.2 Rationale for the proposed study

#### *Prednisolone*

Reducing antibiotic use is crucial to lowering the risk of antibiotic resistance. One of the methods is to use an alternative treatment that does not involve antibiotics. The current alternative treatments (i.e. ear drops, herbal products, probiotics, zinc, decongestants) demonstrate insufficient evidence on their benefits for AOM [15,16]. It is important to understand the pathophysiology of AOM, which is an inflammatory process involving both cellular and chemical inflammatory mediators (i.e. cytokines, chemokines, mast cells, prostaglandins, leukotrienes) in the middle ear. These inflammatory mediators contribute in altering the vascular permeability, increasing mucous glycoprotein secretion, as well as stimulating the chemotaxis process, the activity of epithelial secretion and other mediators [17]. An intervention suppressing this inflammatory process, could have an important role in the resolution of AOM. Corticosteroids suppress the inflammation process by inhibiting the mediators and cytokines characteristic of AOM, the recruitment of leukocytes and monocyte-macrophages into affected areas, and the synthesis and/or release of numerous inflammatory mediators and cytokine, and also reducing vascular permeability [18].

Corticosteroids are produced in the adrenal cortex. Cortisol (glucocorticoids), one of the most common corticosteroids, is responsible for anti-inflammatory effects. The production of glucocorticoids is controlled by hypothalamus, pituitary, and adrenal (HPA) axis. Corticosteroid treatment may affect the production of natural corticosteroid by suppressing the HPA axis [19,20]. We have identified prednisolone is commonly used and safe in the treatment of inflammatory and autoimmune diseases in children. Prednisolone, a synthetic intermediate-acting glucocorticoid with a biological half-life of 12 to 36 hours, is commonly used in the treatment of inflammatory and autoimmune diseases in children. Although prednisolone has a lesser anti-inflammatory potency compared to other common corticosteroids (i.e. methylprednisolone, dexamethasone), but it has lesser growth effect which is one of the concerning issues in the disease management in paediatric population [19].

We will give prednisolone at a dose of 1 mg/kg to 2 mg/kg body weight based on age category, once daily for five days. As there is a wide therapeutic dose window for prednisolone, this will enable us to operationalise the dose as 10 mg/day for children aged six months less than two years; 20 mg/day for children aged two to five years; and 30 mg/day for children aged six to 12 years, simplifying both randomisation and dosage instructions. The current treatment for AOM does not include corticosteroids in the guidelines. Therefore, we determined the dose and duration of prednisolone based on the doses regularly used in the paediatric otitis media trials and regular dose for other inflammatory and infection diseases in children based on the international and national practice guidelines, such as bronchial asthma, juvenile rheumatoid arthritis, and acute bacterial meningitis [21-25]. The duration of corticosteroid use in otitis media trials varies between three to seven days [18, 26-28]. An animal study [29] using mice infected with Streptococcus pneumoniae and non-typeable H. influenzae (NTHi) bacteria demonstrated that most of AOM-related cytokines peaks at three to six hours after the infections (interleukin-6/IL-6, interleukin-1 alpha/IL-1α, tumor necrosis factor alpha/TNF-α) an at six hours to three days (interleukin-10/IL-10). In general, these cytokines will be progressively reduced between the fourth to sixth day of infection and the acute otitis media will be resolved after the sixth day [29]. Therefore, we will give the prednisolone for five days in order to boost the natural resolution mechanism in AOM cases and to minimize the potential harms of corticosteroid use even though 7-day duration is still regarded as short-term use.

A single daily dose is preferable over divided doses to prevent the hypothalamic-pituitary-adrenal (HPA) axis suppression. Prednisolone should be given in a single dose at 6 to 8 am in the morning to mimic the normal diurnal rhythm of cortisol production [19,20] and because it is also more convenient for children and parents in the study to just take a trial drug once a day.

### *Potential harms*

Despite the favourable effect of corticosteroids for inflammation, there are still several potential adverse effects related to its short-term use. A systematic review identified side effects of short-course of corticosteroids (less than two weeks) in children, such as gastrointestinal disturbances (i.e. vomiting, gastritis, nausea), behavioural changes (i.e. mood swings, nervousness), HPA axis suppression, increased blood pressure, hyperglycaemia, weight gain, and decreased bone mineralisation [30]. Even though there were more children experiencing these side effects compared to placebo, the included studies used a diverse of corticosteroids' types and duration, as well as the results were uncertain and include both important beneficial and harmful effects of

corticosteroid. Vomiting and behavioural disturbances (i.e. anxiety, aggressive behaviour) are the common side effects [30].

Regarding vomiting, there were three studies comparing prednisolone to placebo or control (other type of corticosteroids). A good quality RCT [31] included children aged 10 to 60 months with virus-induced wheezing who received a single dose of inhaled albuterol. These children then were randomly allocated to either prednisolone (10 mg oral prednisolone for children aged 2 years and younger; 20 mg for aged >2 years) group (n=343) or placebo (n=344). No significant differences on clinical outcomes (e.g. time to hospital discharge) or adverse effects between two groups were detected. There was one child from prednisolone group who vomited that required the discontinuation of the prednisolone [odds ratio (OR) 3.02 (95% confidence interval (CI) 0.12 to 74.33; p-value=0.50; number needed to harm (NNTH; number of children who are treated with prednisolone that will result in one additional event of side effects) = 34 children)] [31]. One study [32] on children aged one to 17 years with acute asthma presenting to the emergency department (ED). The children received a dose of inhaled albuterol and either a single dose of oral prednisone 2 mg/kg (n=41) or placebo (n=40). There was no a significant difference in the incidence of vomiting after taking the prednisone between the prednisone group (n=3; 7.3%) and the placebo group (n=1; 2.5%) [OR 3.08 (95% CI 0.31 to 30.92; p-value=0.34; NNH=21)] [32]. The same author with similar inclusion criteria [33] demonstrated a significant difference on the incidence of vomiting between children who received single dose prednisone 2 mg/kg/day (n=10/66) and nebulised dexamethasone 1.5 mg/kg (n=0/62), however the confidence interval was very wide that included a high number of NNTH (if we treat more than 50 children with prednisone, then we will expect one additional event of vomiting) [OR 23.23 (95% CI 1.33 to 405.54; p-value=0.03; NNH=7)] [33].

Regarding behavioural changes, one RCT [34] randomly allocated children aged two to 16 years with acute exacerbation of mild persistent asthma to receive either oral prednisone/prednisolone high dose (2 mg/kg/day) or low dose (1 mg/kg/day) for five days. There were significant differences in observed adverse events between high-dose and low-dose groups in regard to anxiety (9/43 vs 2/43, respectively) [OR 5.43 (95% CI 1.10 to 26.83; p-value=0.04; NNTH=7)] and aggressive behaviour (9/43 vs 0/43) [OR 23.96 (95% CI 1.35 to 426.33; p-value=0.03; NNTH=5)]. The wide of intervals demonstrated a wide variance in the number of children needed to treat to expect one additional adverse event. There were no significant differences in other unfavourable effects (i.e. facial fullness and erythema, abdominal pain, diarrhea, euphoria, depression, and hyperactive) [34]. Another RCT [35] included children aged two to 15 years with acute exacerbation of asthma who were randomly allocated to receive oral prednisolone 1 mg/kg/day for three days (5-day group) vs same dose of prednisolone for three days (3-day group). There were no significant differences in regard to the incidence of rash and behavioural disturbance (e.g. angriness, aggressiveness, crankiness, irritability) between the two groups [35].

An RCT of the use of prednisolone for pediatric AOM reported no significant differences between children who received oral prednisolone 2 mg/kg/day for five days and placebo group who experienced moderate side effects (e.g. drowsiness, nervousness, diaper rash, dry mouth) [18]. This study also demonstrated that there was no correlation between the use of corticosteroid and the persistence or the emergence of viral infections [18]. Other potential side effects correlated with the use of corticosteroids are fluid retention and headache [18,30].

**Based on these trials, we consider 5-day duration of prednisolone for this study is appropriate and safe for children.**

### *Clinical trial of corticosteroids for acute otitis media in children*

Evidence has demonstrated insufficient benefits and harms of corticosteroids. An RCT demonstrated that corticosteroid reduced the duration of ear discharge in AOM children with ventilation tubes [26]. Another RCT demonstrated a temporary resolution of middle ear effusion after five days of corticosteroid treatment [18]. In a systematic review of randomised placebo-controlled trials (RCTs) of steroids for AOM, only two small trials [18,27,36] (very low to low quality) indicated corticosteroids could be useful in this condition. However, our confidence in the results is low, due to small sample size and very wide confidence intervals around the observed results. This insufficient evidence creates a research gap in the management of AOM, particularly in non-severe cases, where antibiotics are not required. Therefore, we propose an adequately powered clinical trial to address this uncertainty.

We will conduct a large, parallel, pragmatic, multicentre, stratified, double-blind, randomised, placebo-controlled trial with the allocation ratio 1:1 to test the effectiveness of corticosteroids for 760 children with AOM including 60 children for a tympanometric mechanistic sub-study and pilot study, described further in this protocol. As a comparator to prednisolone, we will use a placebo for the following advantages: (1) it is the most accurate test in assessing the efficacy of a treatment; (2) it will show the true additional benefits and/or harms of the prednisolone; and (3) it is crucial when the outcome is assessed using subjective measurements. The primary objective and outcome of this proposed trial is to assess the effectiveness of corticosteroids as a monotherapy in children with mild AOM, and as an addition to antibiotics in children with severe AOM, on ear pain at three days after randomisation using visual analogue scale (VAS). The secondary outcomes include ear pain at other time points, total duration and severity of pain, adverse effects, complications of AOM (e.g. perforation of tympanic membrane, mastoiditis), and AOM recurrence.

## 1.1.3 Rationale for pilot study

Prior to our main study, we will conduct a pilot study, described in this protocol. This study will mimic the main study in terms of its process and procedures, but on a smaller scale. However, due to budget constraints, we will conduct a pilot study as a pragmatic, randomised, open-label, single-blind study. We will blind outcome assessors (i.e. physicians and tympanometry technicians), so they will not aware of the allocation of the intervention.

The main study will involve many participating physicians and healthcare facilities across Jakarta, Bekasi, and Depok, most of whom have not been involved in a clinical trial before, will have a long follow-up period up to three months, and will utilise a symptom diary and a specific translated instrument to assess the severity of symptoms (acute otitis media – severity of symptom scale or AOM-SOS) which is not widely recognised by physicians in Indonesia. Therefore, this pilot study is crucial to test the feasibility of the main study, including the characteristics of our main study design, all the study processes and procedures (e.g. the recruitment, stratification, randomisation, outcome measurement), and other operational strategies in our proposed main study.

## 1.2 Objectives

The first objective of our pilot study is to assess the overall process and procedures of the main study, as follows: (1) the recruitment criteria; (2) stratification and randomisation processes; (3) outcome measures using validated and customised tools (e.g. visual analogue scale/VAS, Acute Otitis Media – Severity of Symptoms Scale/AOM-SOS, case report forms/CRFs, symptom diary); (4) identification of any potential practical and operational issues that may appear in the main study which will require re-structuration of the planned methods and procedures after commencing this pilot study; and (5) verification of sample size calculation for main study.

Our second objective is to conduct a mechanistic explanatory study using tympanometry. It aims to assess the efficacy of corticosteroids in improving the resolution of middle ear effusion in AOM.

# CHAPTER 2 – METHODS

## 2.1 Trial design

This study is a pilot of a parallel, pragmatic, stratified, randomised, open-label, single-blind controlled trial of corticosteroids, as monotherapy for mild AOM, and in addition to individually prescribed antibiotics for severe AOM. In the main study, we will stratify eligible children based on the clinical specialty (primary care or secondary/tertiary care) and severity of AOM (mild or severe). However, for this pilot study, we only include ear-nose-throat-specialists (ENTs) who work at tertiary centres for the convenience of the implementation of the mechanistic sub-study as tympanometry is only available at the hospitals (tertiary centres). Therefore, we will stratify the children based on their AOM severity and then will randomly allocate to corticosteroid (prednisolone) or control (usual care without prednisolone) (Figure 1) with the allocation ratio of 1:1. Because it is a n open-label study, the parents/caregivers and an appointed nurse who will perform the randomisation will be aware of treatment allocation, whilst the clinicians and tympanometry technicians will remain unaware of the treatment.

## 2.2 Participants, interventions, and outcomes

### 2.2.1 Study setting

Prior to this study, we conducted a feasibility study to survey the current management of AOM in children in three cities in Indonesia and to identify the willingness of physicians to participate in our proposed clinical trial of corticosteroids for AOM in children. Based on clinical scenarios, there were sufficient number of physicians who would prescribe corticosteroids for AOM. There were 171 physicians from 87 primary/secondary to tertiary centres (public and private) in DKI Jakarta, Depok, and Bekasi who were willing to participate in our proposed main study. However, we will only pilot this study at seven public and private hospitals in Jakarta and Bekasi: (1) Dr Cipto Mangunkusumo Hospital; (2) Persahabatan Hospital; (3) Gatot Subroto Army Hospital; (4) Antam Medika Hospital; (5) Cempaka Putih Islamic Hospital; (6) Proklamasi ENT Hospital; and (7) Hermina Bekasi Hospital.

### 2.2.2 Eligibility criteria

#### 2.2.2.1 Inclusion criteria

We will include 60 children aged six months to 12 years old with AOM, defined as current onset (48 to 72 hours) of AOM-relevant symptoms (e.g. earache, ear tugging/rubbing or irritability in non-verbal children). If it is feasible, otoscopic findings of middle ear effusion (e.g. bulged tympanic membrane, limited or absent mobility of the tympanic membrane, air fluid level, ear discharge) and acute inflammation (e.g. erythema) will confirm the diagnosis.

#### 2.2.2.2 Exclusion criteria

We will exclude children:

1. with major and severe medical conditions (e.g. heart diseases, kidney failure)
2. who are immunocompromised (e.g. HIV, children receiving cancer treatment)
3. with congenital malformations and/or syndromes (e.g. cleft palate, Down's syndrome)
4. with ear ventilation tube(s)

5. exposed to persons with varicella (chicken pox) or active Zoster infection in the past three weeks without prior varicella immunisation or infection
6. who have high risk of strongyloidiasis infections with symptoms and signs of unexplained eosinophilia, skin reaction due to larvae penetration into the skin (e.g. inflammation, oedema, petechiae, severe pruritus), particularly on the feet regions, pulmonary (e.g. dry cough, throat irritation, dyspnoea, wheezing, haemoptysis, repeated episodes of fever and mild pneumonitis), or gastrointestinal symptoms (e.g. upper abdominal pain, diarrhea, anorexia, nausea, epigastric pain, malabsorption, and vomiting)
7. who have taken systemic (i.e. oral, injection) or topical steroids in the preceeding four weeks
8. who have taken antibiotics in the preceeding two weeks
9. who are hypersensitive to prednisolone or prednisone, or other corticosteroids

## 2.2.3 Interventions

Children in the mild AOM group will be randomly allocated to receive either a single dose of prednisolone tablets daily for five days as an addition to expectant observation or expectant observation alone (without prednisolone). At the baseline visit, the participating physicians will inform the parents/caregivers to closely observe the children for 48 hours without immediate antibiotic treatment.

Children in the severe AOM group will be randomly allocated to receive either a single dose of prednisolone tablets daily for five days as an addition to antibiotics according to physicians' preferences or antibiotic alone (without prednisolone). Antibiotic treatment is commonly prescribed for AOM with severe symptoms. The information regarding the antibiotics (e.g. antibiotic type, dose, duration) will be recorded in the case report forms (CRFs).

### 2.2.3.1 Prednisolone and control group

***Prednisolone group***

Prednisolone tablets will be given in the intervention groups in both mild and severe groups. It is given at a dose of 1 mg/kg to 2 mg/kg body weight, once daily for five days. The pharmacist will crush the tablets, mix them with sweeteners, and pack them in a daily paper-pack. It should be given in a single dose at 6 to 8 am in the morning to mimic the normal diurnal rhythm of cortisol production [19,20]. Participating physicians will advise the parents to give the prednisolone at about the same time each day as a daily routine for children which will also help parents to remember.

***Control group***

Due to budget constraints, we are not able to provide a matched placebo as prespecified for the main study. Therefore, we will not give placebo for children in the control group, either in the mild or severe group. Children in the control group of the mild AOM group will still receive expectant observation and those in the severe AOM group will receive antibiotics, and other concomitant treatment based on physicians' preferences (if necessary). The only difference between the intervention and control group is solely whether they receive prednisolone (intervention group) or not (control group).

#### 2.2.3.2 Criteria for trial drug discontinuation or modification

When giving the prednisolone to a child, parent can mix the prednisolone powder with jelly or juice. If children vomit less than 30 minutes after having a dose of prednisolone, parents should give the same dose again. However, if they vomit again after 30 minutes, parents should not give another dose of prednisolone until the next dose on the next day. This should be noted in the symptom diary. If children keep vomiting after receiving prednisolone, parents should contact the research team. If the parents forget to give prednisolone to their children, they can give the missed dose as soon as they remember on the same day and they should also note this in the symptom diary.

If there are any adverse events and adverse drug reactions which have been assessed by research team that would require the discontinuation of drug trial and further assessment and treatment, then the treatment will be discontinued for this particular case, however follow-up will continue, where possible. This will be reported in the CRFs. Serious adverse events will be reported to Bond University's Human Research Ethics Committee (BUHREC) and the Research Committee Ethics Faculty of Medicine Universitas Indonesia (FMUI) – Dr. Cipto Mangunkusumo Hospital (CMH).

#### 2.2.3.3 Adherence monitoring

Participating physicians will provide information regarding the administration of the prednisolone. The researcher will send a daily text-message reminder to all the parents in both prednisolone and control groups during the intervention period of five days to take the drug regularly and to complete the symptom diary daily. The text-message will also remind all the parents to visit the clinic after the 48-hour expectant observation or visit-1 (day-3), visit-2 (day-7), visit-3 (day-30), and visit-4 (day-90) for re-assessment. At each visit-1 and visit-2, the parents will return the first and second mini-booklets of symptom diary and the left-over drug to the appointed nurse (at visit-2). The nurse will then check the symptom diaries and the left-over drug for the adherence in taking intervention drugs. We will visit the patients' homes to collect the third mini booklet of symptom diary that will record the symptoms from day-7 to day-14 after the baseline visit.

#### 2.2.3.4 Concomitant care and interventions

Physicians may give symptomatic medicine (i.e. ibuprofen, acetaminophen, decongestant, mucolytic) according to their usual practice and these will be recorded in the CRFs and symptom diary. Parents will record the use of these medicine in the symptom diary. The decision on concomitant medication will be made without knowledge of allocation to the prednisolone or control group.

### 2.2.4 Outcomes

#### 2.2.4.1. The pilot study outcomes

***Recruitment rate***

Recruitment rate is defined as the proportion of consultations with potentially eligible children who provide their consent to be included in the study. As this is recognised as a crucial aspect of conducting a clinical trial and may cause study discontinuation due to low recruitment [37], we will assess this in our pilot study. We will identify the rates and challenges during the recruitment process and determine the best strategy for the main study to overcome these challenges and obstacles.

***The success of the study procedures***

The success of the study procedures includes the following: (1) obtaining informed consent from the patients and their parents; (2) the recruitment based prespecified eligibility criteria, including the use of otoscope to diagnose AOM if feasible; and (3) the stratification and randomisation process, including stratifying eligible children based on the severity and obtaining the allocation result whether the children will be allocated to the prednisolone or control group.

***Ability to measure planned outcomes in the main study***

We will assess the ability to measure planned outcomes in main study, which are: (1) the proportion of children with pain reduced by at least the minimum clinically important amount, at day-3 after randomisation. This will be assessed using a VAS recorded in the patient symptom diary; (2) the severity of pain and other AOM-relevant symptoms at various time points using VAS, AOM-SOS, and the symptom diary; (3) duration to AOM resolution; (4) adverse effects, defined using standard clinical trials criteria, recorded in the diary, and reported to the central office and ethics committee as required.; (5) complications of AOM (e.g. perforation of tympanic membrane(s), mastoiditis); and (6) AOM recurrence, defined as a new episode of AOM at one to three months after randomisation. We will report these narratively due to a limited sample size and insufficient formal power calculation for this pilot study to be able to detect actual effects of corticosteroids to improve clinical outcomes in AOM.

***The compliance to study and study drug***

The compliance to study and study drug is defined as a proportion of children who regularly take the study drug (assessed using the symptom diary and any left-over drug) and come to follow-up visits per protocol. Participants will be followed-up closely by clinicians and research staff. Children will return for a visit at day-3 after randomisation, ensuring collection of the primary outcome.

***The verification of sample size calculation for main study***

Based on our size calculation, we plan to enrol 760 children in the main study. We estimate that there will be 35% of the total sample of children with AOM in the severe group (i.e. children with severe symptoms, fever ≥ 39°C, children aged < 2 years with bilateral AOM, AOM with perforation of tympanic membrane). Therefore, within this pilot study, we can assess the accuracy of this assumption. Also, we will check another assumption of the sample size calculation, which was the proportion in the control group with the resolution of pain at three days, which was 42.5%.

### 2.2.4.2. The mechanistic or tympanometry sub-study outcomes

### Primary outcomes

***The change in middle ear effusion (MEE) at various time points***

We will assess the change in the MEE at the following time points: baseline visit, visit-1 (day-3), visit-2 (day-7), visit-3 (day-30), and visit-4 (day-90). We will measure MEE using static acoustic admittance, defined as "the amount of energy absorbed by the tympanic membrane and middle ear, measured in mmho or mL" [38]. We will also measure the difference of this results between the intervention and control group.

### Secondary outcomes

***Duration of MEE***

We will also assess the duration to the resolution of MEE using tympanometry.

***The correlation between ear pain and other symptoms with the changes in MEE at various time points***

We will identify the correlation between ear pain and other symptoms (i.e. ear tugging, irritability, crying, lack of sleep, lack of appetite, less of playfulness, fever) with the changes in MEE at various time points.

### 2.2.5 Participant timeline

Table 1 illustrates the timeline for visits and follow-ups. We will measure the outcomes at various time points: (1) visit-1 after the 48-hour observation (day-3); (2) visit-2 (day-7); (3) visit-3 (day-30); and (4) visit-4 (day-90). Patients will visit the hospital at visit-1 and visit-2, whilst the last two visits will be a home-visit. However, children in the mechanistic sub-study have to visit the hospitals at those follow-up visits. To improve the compliance of the study, the research personnel will send a daily text-message reminder to all the parents in the study during treatment period to take the drug, complete the symptom diary, and visit the clinic after the 48-hour expectant observation and other time points. At visit-2, parents will return the symptom diary and any left-over drug.

**Table 1.** Follow-up timeline

| | **STUDY PERIOD** | | | | | |
|---|---|---|---|---|---|---|
| | **Enrolment Allocation** | **Post-allocation** | | | | **Close-out** |
| **TIMEPOINT** | **0 (Day-0)** | **t1 (Day-3)** | **Intervention ends (Day-5)** | **t2* (Day-7)** | **t3* (Day-30)** | **t4* (Day-90)** |
| **ENROLMENT:** | | | | | | |
| Eligibility screen | X | | | | | |
| Informed consent | X | | | | | |
| Allocation | X | | | | | |
| **INTERVENTIONS:** | | | | | | |
| *[Intervention A] Prednisolone (5 days)* | — | — | — | | | |
| *[Intervention B] Control (5 days)* | — | — | — | | | |
| **ASSESSMENTS:** | | | | | | |
| *Baseline examination (weight, height, BP, body temperature)* | X | X | | X | X* | X* |
| *Severity of pain and duration using VAS* | X | X | | X | | |
| *Overall symptoms and its duration using AOM-SOS* | X | X | | X | | |
| *Adherence to trial drug* | X | X | | X | | |
| *Adverse effects* | X | X | | X | | |
| *Otoscopic examination* | X | X | | X | X* | X* |
| *Tympanometry examination* | X[+] | X[+] | | X[+] | X[+] | X[+] |
| *Complication* | X | X | | X | | |
| *Recurrence of AOM* | | | | | X* | X* |

*BP=blood pressure; VAS=visual analogue scale; AOM-SOS: acute otitis media-severity of symptoms scale*

**The follow-up will be carried out by home-visit and phone, but those in the mechanistic sub-study will have their follow-up visits to the clinics; [+]These time-point assessments will be applied only for children in the mechanistic sub-study*

***Participant enrolment***

In the main study, we will stratify eligible children by the clinical specialty (primary care or secondary/tertiary care) and severity of AOM (mild or severe). However, we only include ear-nose-throat-specialists (ENTs) who work at tertiary care in this pilot study. Therefore, we will stratify the children based on their AOM severity and then will randomly allocate these eligible children to receive either a single dose prednisolone for five days or without, as an addition to expectant

observation in the mild AOM group or as an addition to antibiotics according to physicians' preferences (e.g. antibiotic type, dose, duration) in the severe AOM group (see Figure 1).

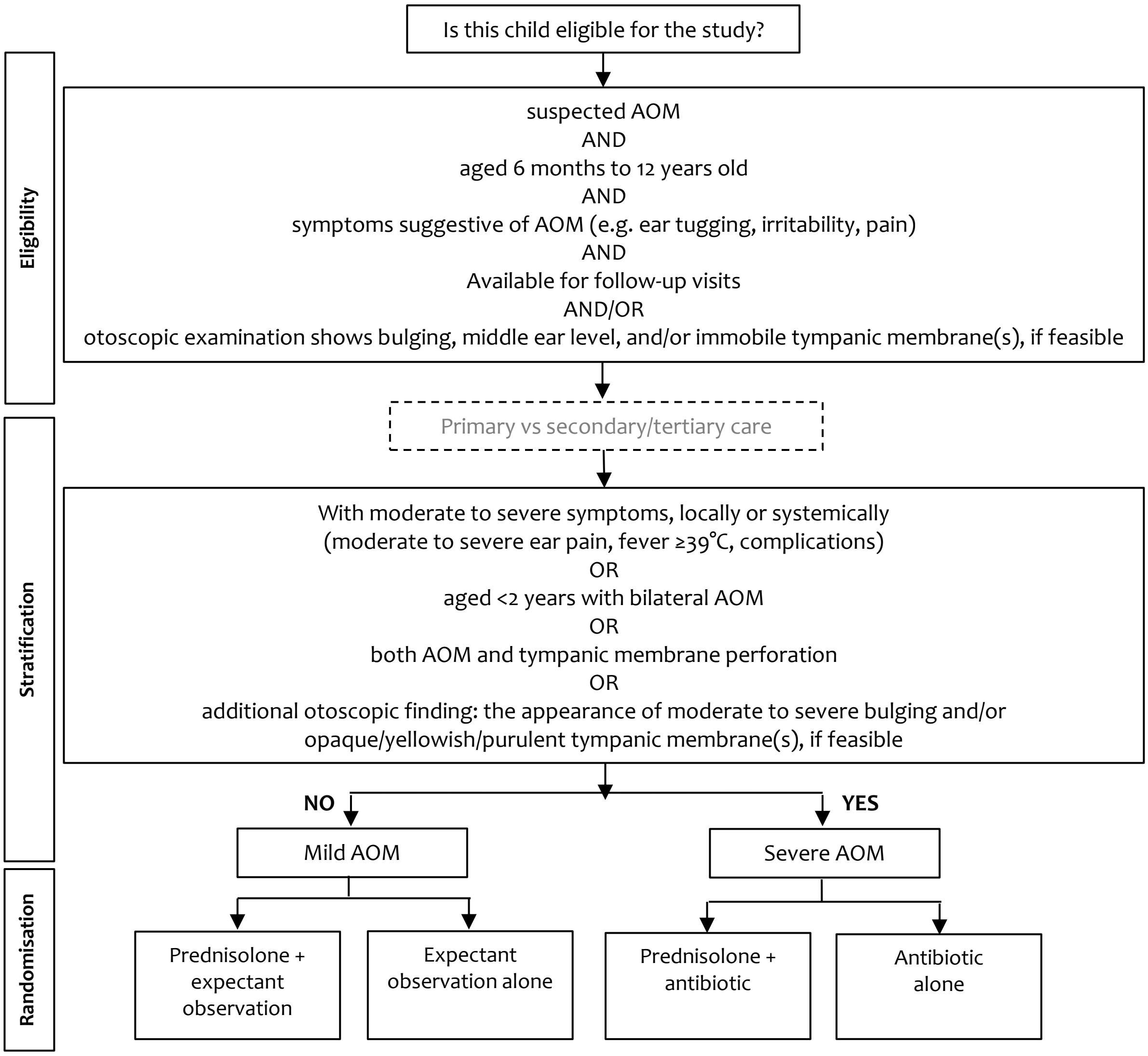


**Figure 1.** Flow chart of the stratification and randomization of the study

Participating physicians will assess the eligibility of children who come to the hospital based on their symptoms and clinical features of AOM. The eligible children will then be stratified based on the severity of AOM to either mild or severe AOM group. This process will be assisted using an eligibility and randomisation form. The participating clinicians will then obtain a clinical history and perform a baseline examination, including general, otoscopic, and tympanometry examination. The results of these procedures will be recorded at the baseline history form and outcomes form. The tympanometry examination will be conducted by an audiologist or a tympanometry technician. The physician will assess and analyse the tympanometry findings and record the results in the outcome form. After these procedures, the children will be sent to the appointed nurse who will perform the randomisation and dispense the prescription for a study medication (prednisolone) if the patient is assigned to the intervention group.

### 2.1.6 Sample size

We did not determine a sample size for the pilot study. There are several suggestions in calculating the sample size for a pilot study (e.g. at least 55 participants or at least 9% of the sample size of the main study) [39]. Since we will need 60 children in the mechanistic sub-study, we will include 60 children with AOM in our pilot study. The sample size of the mechanistic study was determined based on the main primary outcome, which is the mean value of static acoustic admittance or acoustic compliance in the tympanogram. In a previous study of children with middle ear effusion (MEE) who underwent tympanometry assessment and had a history of chronic or recurrent middle ear disease [40], the response within each subject group was normally distributed with standard deviation 0.3. If the true difference in the experimental and control means is 0.3 units, we will need to study 22 experimental subjects and 22 control subjects to be able to reject the null hypothesis that the population means of the experimental and control groups are equal with probability (power) 0.9. The Type I error probability associated with this test of this null hypothesis is 0.05. With a 20% allowance for dropouts, the total sample size becomes 56 or we will include 60 children for this pilot study.

Using the worst-scenario that of 50 physicians who work at seven appointed hospitals and have approximately 97 patients with AOM per week, we estimate that only 30% of the physicians will confirm their participation and 25% of the patients will give their consents to participate in our study. We then estimate that it will require nine months to recruit 60 children with AOM in this pilot study, including the extra months for potential slow recruitment and last follow-up (three months after the baseline visit).

### 2.1.7 Recruitment

**Recruitment of physicians**

Our feasibility survey study (April–August 2016) demonstrated there were 352 physicians (general practitioners, ear-nose-throat specialists, and paediatricians) who were willing to participate in our main study and they had 705 paediatric patients with AOM in the past seven days. For our pilot study, we identified 50 physicians from the most feasible seven hospitals located in DKI Jakarta who were willing to participate in our study and had approximately 97 paediatric patients with AOM in a week.

**Recruitment of participants**

After physicians deliver the patient information and obtain the consent from the eligible children, they will use the eligibility and randomisation form to assist them in stratifying the children based on their AOM severity. Physicians then will complete other CRFs, as the following: baseline history form and outcome form. Physician will assist the parents to complete the symptom diary. The similarity on the forms and sequences of both outcome CRF and symptom diary will help parents to be able to fill those correctly.

## 2.3 Assignment of interventions

### 2.3.1 Allocation

***Sequence generation and implementation***

All children and their parents who are eligible and consented will be enrolled, and stratified based on their AOM severities or clinical features. The randomisation process will be performed by the

appointed nurse who will randomly allocate children to either prednisolone and expectant observation or control (expectant observation alone) in the mild group and either antibiotic with prednisolone or control (antibiotic alone) in the severe group. A permuted block randomisation sequence will be computer-generated by the Centre for Research in Evidence-Based Practice Bond University, Gold Coast, Australia. Random numbers will not be disclosed to the outcome assessors (i.e. participating clinicians and audiologists/tympanometry technicians), to ensure allocation concealment. Batches of intervention packages will be dispatched to participating centres from a central pharmacy facility at the Clinical Research Supporting Unit, Faculty of Medicine Universitas Indonesia (CRSU FMUI).

***Allocation concealment mechanism and implementation***

The information of the eligibility and stratification which is provided by the participating physicians will help the appointed nurses to obtain the information from the randomisation website, whether the children will be allocated to the prednisolone or control group, identified by the 2-digit patient ID numbers. During the consultation, the physician will prescribe study medication for every subject with the dose based on the patient age and insert the prescription in the CRF folder. The nurse who performs the randomisation will give the prescription to the subjects who are allocated to the intervention group (prednisolone group). The subject then will give the prescription to the pharmacy, where the pharmacist will prepare the prednisolone by crushing the tablets, mixing them with sweeteners, packing the prednisolone mixed powder in a daily individual paper-pack for five days, and dispense these to the subjects along with instructions for preparation. The pharmacist will record the dispensing on the form provided by the study for this purpose.

### 2.3.2 Blinding (masking)

In this study, the appointed nurses and the children and their parents will know the allocation of the intervention. We will ensure that the participating physicians and the audiologists/ tympanometry technicians will be blinded to the intervention allocation during the study.

***Emergency unblinding***

The unblinding process should be done if there are serious adverse events and limited only to that particular participating physician.

## 2.4 Data collection, management, and analysis

### 2.4.1 Data collection methods

We will assess the outcomes using CRFs, patient symptom diary, and feedback forms. The CRFs consist of eligibility-and-randomisation, baseline history, outcomes, serious adverse events (if applicable), and drug dispensing and returned forms. The outcome form will record the severity of pain and overall symptoms with their durations using VAS and AOM-SOS, as well as the resolution of AOM signs using otoscopic and tympanometry examinations (for the mechanistic study).

We will identify the recruitment rate by assessing the proportion of children who provide their consents divided by the proportion of consultations with potentially eligible children during the trial. We will use the informed consent and a study recruitment log book to record the reason(s) why children were not randomised.

We will assess the success of the study procedures using feedback forms. On the feedback form, patients and their parents and participating physicians will rate their understanding and challenges

they have during the implementation of study procedures (e.g. obtaining the randomised 2-digit patient ID numbers and their allocation of the treatment, completing the informed consent forms and CRFs) using grading scale ranged from one to five (1=very easy; 2= easy; 3=moderate/neutral; 4=difficult; 5=very difficult).

To assess the ability to measure planned outcomes in main study, we will also use a feedback form to identify the understanding, the challenges, the complexity of the outcome assessment tools utilised for this study (e.g. CRFs and patient symptom diary which includes VAS and AOM-SOS) from the perspective of the patients and their parents and the participating physicians. The CRFs and symptom diary will record the clinical history and symptoms (e.g. VAS, AOM-SOS), as well as physical examination (e.g. temperature, blood pressure, otoscopic examination if feasible). This information will be obtained from the perspectives of the patients and their parents and the clinicians.

The VAS is acknowledged as a well-established and validated scale for assessing pain [41]. It has a 100-mm horizontal scale with 'no pain' anchor at the left endpoint and 'the most severe pain' at the right endpoint of the scale. The patient will mark a vertical line along the horizontal line as the representation of their pain level. The scale will be determined by measuring the distance from the left endpoint ('no pain') to the marked line [42]. A 10-mm difference has been reported to indicate a clinically significant change [43,44]. The AOM-SOS is used to assess the severity of other AOM-relevant symptoms daily. particularly in non-verbal children. Table 2 illustrates several AOM-related symptoms described as "no", "a little", and "a lot". This scale was developed as an outcome reporting tool scoping symptom and activity limitation due to AOM in the proceeding 12 to 24 hours [45]. Shaikh et al. [45] used the mean of 4.2 points as a minimal important difference. We have translated the original English) version of AOM-SOS to Indonesian version of AOM through forward and backward translation process.

**Table 2**. Acute otitis media severity of symptoms scale (AOM-SOS) [45]

| **We are interest finding out how your child has been doing. For each question, please place a check mark in the box corresponding to your child's symptoms. Please answer all questions** | | | |
|---|---|---|---|
| | No | A Little | A Lot |
| Over the past 12 h, has your child been tugging, rubbing, or holding the ear(s) more than usual? | ☐ | ☐ | ☐ |
| Over the past 12 h, has your child been crying more than usual? | ☐ | ☐ | ☐ |
| Over the past 12 h, has your child been more irritable or fussy than usual? | ☐ | ☐ | ☐ |
| Over the past 12 h, has your child been having more difficulty sleeping than usual? | ☐ | ☐ | ☐ |
| Over the past 12 h, has your child been less playful or active than usual? | ☐ | ☐ | ☐ |
| Over the past 12 h, has your child been eating less than usual? | ☐ | ☐ | ☐ |

| | | | |
|---|---|---|---|
| Over the past 12 h, has your child been having fever or feeling warm to touch? | ☐ | ☐ | ☐ |

The compliance to the study and study drug will be identified by assessing the completion of CRFs and symptom diary, particularly on the attendance of patients and the parents on their scheduled follow-up visits, the completion of the trial drug based on the symptom diary and the left-over drug. We will identify the parents who have low literacy during the informed consent process. For parents who have low literacy, we will visit their home one day after the initial visit to identify a person who lives nearby (e.g. family members, neighbours) that are able to assist the parents in completing the symptom diary, daily for two weeks. If there is noone can assist them, then the research personnel will assist them in completing the symptom diary by phone. If it is not feasible, we will visit their home daily to be able to assist them.

To assess the verification of sample size calculation for main study, we will use the CRFs.

To ensure that all the outcome data can be sufficiently collected and recorded properly according to prespecified plans, we will conduct an individual/institutional training for participating physicians prior the implementation of this pilot study. The training will include following sections: (1) introduction and summary of the study; (2) introduction of international clinical practice guidelines of AOM; (3) The diagnosis of AOM using otoscope and reporting the otoscopic results; (4) the introduction and dissemination of the principles of quality methodology clinical trial (e.g. eligibility, randomisation, blinding, outcome assessment) and good clinical practice (e.g. patient consents, confidentiality, data management); (5) practical steps of eligibility assessment, stratification, and randomisation; and (6) practical steps in completing study documentation (i.e. patient informed consents, CRFs, feedback forms, patient symptom diary).

### 2.4.2 Data management

The integrity and completion of data will be maintained through mechanisms such as consistency checks during data entry, and cross-checks between items after data entry. All the actions and modifications to data stored in the database will be documented and retrievable for viewing. Missing data or errors will be detected before final submission to the electronic database and will be recorded in a summary along with the descriptions for each missing and/or error data. The summary will then be notified to the co-investigators in that hospital for further investigation by checking and confirming the original forms or other resources for correction or completion for those with missing and/or erroneous data. The modification to original forms will be done by research personnel at that hospital and will be documented on paper and electronic versions. It will be annotated with the date, name, and signature of the person who is responsible for making modifications.

The central data coordinator will check the validity and completeness of study data on a regular basis. All data in the central database will be protected with a regular complete back up system.

### 2.4.3 Statistical methods

For the recruitment rate, we report the outcome as the proportion of children in percentages (%). For the success of the study procedures and the ability to measure planned outcomes in main study, we will report the outcomes as the proportion of clinicians in percentages based on the grading

scale of their feedback reporting on prespecified outcome measure tools. For the compliance to study and study drug, we will report the outcomes as the proportion of children in percentages who attend the follow-up visits and complete the cycle of study drug.

To assess the verification of sample size calculation for main study, we will report this outcome as the proportion of children in each stratum (mild and severe AOM group) and those with pain at Day-3 after randomisation in the control group.

For the mechanistic sub-study, we will report continuous variables (i.e. the change in MEE at various time points (mean in days standard deviation), the duration of MEE) as a mean difference (MD) with 95% confidence intervals (CI) also the difference between two groups. We also will report the correlation between ear pain and other symptoms with the changes in MEE at various time points.

## 2.5 Monitoring

### 2.5.1 Data monitoring

#### *Data monitoring committee*

Since this is a short study, we do not need data monitoring committee for this pilot study. However, independent personnel from Clinical Epidemiology and Evidence-Based Medicine (CEEBM) Unit, Dr Cipto Mangunkusumo Hospital (CMH) – Faculty of Medicine Universitas Indonesia (FMUI), who is not involved in this study, will assess the process and the quality of patient recruitment, data entry, and a compilation of research data in central database. Her feedback will be important to improve the implementation of our main study.

#### *Interim analysis*

Due to the small number of recruited patients to the trial and the duration of the trial will be lesser than one year, we will not perform an interim analysis.

### 2.5.2 Harms

An adverse event is defined as any untoward medical occurrence in a patient or clinical investigation subject administered a pharmaceutical product and which does not necessarily have to have a causal relationship with trial drug; whereas adverse effects or adverse drug reaction, is defined as all noxious and unintended responses to a trial drug related to any dose.

At the Visit-2 (Day-3), the appointed nurse is the first person who will identify any adverse effects (AEs). These will be identified by history taking (e.g. interview) and the symptom diary. The parents will provide information in the symptom diary regarding the adverse effects and whether they seek any treatment or medical assistance to manage AEs. The nurse then will report the adverse effects to the physicians along with the information of the intervention group which the child was allocated to. This information will be provided after the physician assess and record the primary outcome (i.e. ear pain three days after the randomization) on the 'Outcome form'. This is important to keep the concealment.

Adverse events and adverse drug reaction will be collected after they sign the written consents and being enrolled in the trial. All adverse events occurring after the enrolment into the study, during the additional treatment or hospitalization due to adverse events and/or ADR will be

recorded. A subject who experiences a serious adverse event (SAE), defined as any untoward medical occurrence at any dose that may result in-patient and/or prolonged hospitalization, persistent or significant disability, medically important events, life threatening events, and death, will receive sufficient treatment and will be recorded and reported to the Research Committee Ethics FMUI – CMH and the Bond University's Human Research Ethics Committee (BUHREC). We will responsible for any additional examinations and/or treatment that are required to manage AEs.

We will not report SAE occurring after the trial discontinuation, unless there is a temporal relationship between trial drugs or other protocol procedure to the events, as well as whether the event is unexpected or unexplained given the subject's clinical course, previous medical conditions, and concomitant medications. All the SAE will be recorded in SAE form.

### 2.5.3 Auditing

For the main study, we will establish an audit committee from the CRSU FMUI and CEEBM Unit CMH-FMUI which is independent from the trial investigators and the funding body. However, this will not occur separately from monitoring for the pilot study. This independent committee will conduct monitoring of source paper and electronic documents in the website system, monitor the conduct of trial in multicentre sites, interviewing the investigators and coordinators, and check the storing, distribution, and the use of trial drugs. At the start of the trial, the committee will ensure that the research staff are capable in data entry and in using the website system. Observation and quality assessment of the whole trial will be ensured to be always in accordance with the protocol and International Conference Harmonization – Good Clinical Practice (ICH-GCP) standards.

## 2.6 Ethics and dissemination

### 2.6.1 Research ethics approval

This study will be conducted according to the Declaration of Helsinki and ICH-GCP guidelines. We will seek ethics approval from: (1) the Bond University's Human Research Ethics Committee (BUHREC) Bond University, Queensland, Australia; (2) the Medical Ethics Committee of the Faculty of Medicine Universitas Indonesia – Dr. Cipto Mangunkusumo General Hospital, Jakarta, Indonesia; (3) the Directorate-General for Politics and General Government – The Ministry of Internal Affairs Republic Indonesia; (4) the Health Agency for the Province of DKI Jakarta; and (5) local research committee at each participating hospital.

### 2.6.2 Protocol amendments

Any modifications to the protocol which may impact on the trial process (e.g. the modification of study objectives, study design, study population, sample sizes, the procedures, and significant administrative sectors), potential benefits and harms/safety of the patients will require a formal amendment to the protocol. This amendment will be notified and approved by the funding body and the Ethics committee prior to its implementation. Notification is also applied to the health authorities in accordance with local regulations. Minor modifications that may not impact on the trial process will also be notified and approved by the funding body and will be notified to The Ethics Committee.

### 2.6.3 Consent

The participating physician will provide patient information sheet and obtain informed consent from the parent(s) or legal guardian of patients, before conducting the recruitment and

randomisation process. In obtaining the consent, the investigators will inform the trial process including known and potential risks from the trial. As children are considered a vulnerable population, those aged younger than 12 years old are considered not competent to give research consent and the parent or their legal guardian will make the decision. The parent can also make decision for children aged 12 years, however there should be assent from children to participate in the research. For parents who are illiterate, we will identify the literacy level before we start the informed consent session and will ensure that the language used in the oral and written information about the trial, including the written informed consent form, should be understandable to the parents and justified to their literacy level. During the process, the participating physician will confirm the participant's understanding of the process of the trial, including the risks and benefits. For particularly crucial topics, the participating physician will ask several questions related to that topic to ensure they are well-informed and understand. Gaining informed consent will be a major topic discussed during training sessions for staff.

The person who delivers the consent (i.e. participating physician, research assistant) also will provide their signatures on the consent form, stating that they have provided information and opportunity for potential participants to understand and raise relevant questions according the trial. We will ensure that the consent process is free of coercion. As the participation into the trial is voluntary, we will emphasise their rights to withdraw from the trial at any time without any consequences, particularly on the quality of their healthcare services.

### 2.6.4 Confidentiality

All information related to the trial will be stored securely at the study site and the research office. All participant information will be stored in locked file cabinets in areas with limited access. All data collection, including CRFs, test results, and administrative forms will be kept confidential by only using coded IDs as identifiers and will be stored separately from all forms and records that contain names or other identifiers (e.g. informed consents forms). All databases will be secured with limited access using password-protected access systems. All counselling sessions and general to specific examinations (e.g. ear, nose, throat examination, otoscopic and tympanometry examination) will be conducted in private rooms in the participating physicians' clinics or hospital. All the involved research staffs such as physicians, nurses, and audiologists will be required to sign agreements to preserve the confidentiality of all participants.

The confidentiality of every participant will be maintained and will not be distributed externally without the written permission of the participant, except as necessary for trial monitoring by national regulatory authorities related to the medical and research safety.

### 2.6.5 Declaration of interests

Respati W. Ranakusuma (RWR) has nothing to disclose.
Amanda McCullough (AMC) has nothing to disclose.
Elaine M. Beller (EMB) has nothing to disclose.
Christopher Del Mar (CDM) has nothing to disclose.
Eka Dian Safitri (EDS) has nothing to disclose.
Yupitri Pitoyo (YPO)has nothing to disclose.
Widyaningsih (WID) has nothing to disclose.
Arie Sulistyowati (ARS) has nothing to disclose.

Sudigdo Sastroasmoro (SSO) has nothing to disclose.

### 2.6.6 Access to data

The Principal Investigator will be given access to the cleaned data sets. She will also have direct access to each sites' data sets and by request. Project data sets will be secured using password. To ensure confidentiality, data dispersed to project team members will be blinded of any identifying participant information.

### 2.6.7 Ancillary and post-trial care

Short-term corticosteroids are very unlikely to have harm outside those we will be measuring. However, we will responsible for the adverse effects that occurring from the trial drug during the trial (immediate) and post-trial (ancillary care) related to trial drug. The compensation will include the treatment cost relevant with the trial drug, such as the consultation visit, additional examinations, and treatment (e.g. medicine, hospitalization cost). Due to other potential concurrent treatments within the drug trial, there will be robust review and analysis process to conclude the cause of adverse events. Participating physicians will explain the procedure for the management of adverse effects of trial drug during the process of consent approval before entering the trial. We will also include this information on the patient symptom diary, including the 24-hour emergency call and list of recommended healthcare providers.

### 2.6.8 Dissemination policy

#### 2.6.8.1 Trial results

Trial results, either statistically significant or non-significant, and other components of the trial (literature review, survey study, pilot study, etc.) will be reported in a journal manuscript after being distributed to all the principal investigators to be reviewed.

#### 2.6.8.2 Authorship

The authorships and contributions of this trial will be acknowledged on the protocol, manuscript, and the report. Before the publication in medical journal or paper presentation, the principal investigators (PIs) will provide written consent of their acknowledgment and contribution in the reported trial.

- Respati W Ranakusuma contributes in: (1) designing and developing the protocol; (2) conducting the trial; and (3) interpreting and reporting the trial in the final trial report and manuscript for publication.
- Amanda McCullough contributes in: (1) the protocol development (study design and methods); (2) interpreting the results; and (3) the writing process of the final trial report and manuscript for publication.
- Elaine M Beller contributes in: (1) the protocol development (study design, methods, and statistics); (2) interpreting the results; and (3) the writing process of the final trial report and manuscript for publication.
- Chris Del Mar contributes in: (1) the protocol development (study design, methods, and statistics), and (2) the writing process of the final trial report and manuscript for publication

- Sudigdo Sastroasmoro contributes in: (1) supervising the conduct of the trial in Indonesia; (2) interpreting the result; and (3) the writing process of the final trial report and manuscript for publication.
- Eka Dian Safitri contributes in: (1) developing the mechanistic study using tympanometry; (2) providing training related to using and interpreting tympanometry to physicians, nurses, and tympanometry technicians; (3) interpreting the tympanometry findings; and (4) reviewing the final study report and manuscript for publication
- Yupitri Pitoyo contributes in: (1) recruiting secondary and tertiary healthcare centres including the physicians and nurses; (2) interpreting the tympanometry findings; and (3) reviewing the final study report and manuscript for publication
- Widyaningsih contributes in: (1) submitting a research ethics application to the Medical Research Ethics Committee in Indonesia; (2) submitting clinical trial permits to Indonesian institutions; (3) supporting the data collection and management; and (4) reviewing the final study report and manuscript for publication
- Arie Sulistyowati contributes in: (1) providing advice and expertise in terms of paediatric patients; (2) supporting the data analysis; and (3) reviewing the final study report and manuscript for publication

### 2.6.8.3 Reproducible research

We will make the full protocol of this study to be publicly available to maintain its transparency and reproducibility. This full protocol will include detailed information regarding the study, particularly on study design and conduct that not are commonly include in the published protocol or information description in clinical trial registry. We will register the protocol into trial registry such as the Indonesia registry web portal (https://www.ina-registry.org/) and Australian New Zealand Clinical Trials Registry (http://www.anzctr.org.au/). We also will publish the results of this study in relevant medical journal as two separate papers as the following: (1) results of the pilot study and (2) results of the mechanistic sub-study. If necessary, we will include the anonymised participant-level dataset in its appendix or online. Unpublished outcomes will be reported in the full trial report that will be linked to the published study.

## APPENDICES

Appendix 1. Flow chart of patient during the baseline and other visits
Appendix 2. Patient information and consent form
Appendix 3. Case report forms
Appendix 4. Study recruitment log book
Appendix 5. Patient symptom diary
Appendix 6. Product (Lupred®) information summary

# REFERENCES


1. Antibiotic resistance threats in the United States. US Department of Health and Human Services: Centers for Disease Control and Prevention. 2013.
2. The World Health Organization. Global action plan on antimicrobial resistance. Geneva. 2015. ISBN 9789241509763.
3. Harris AM, Hicks LA, Qaseem A. Appropriate antibiotic use for acute respiratory tract infection in adults: Advice for high-value care from the American College of Clinicians and the Centers for Disease Control and Prevention. *Ann Intern Med*. 2016;164:425-34.
4. Pettigrew MM, Gent JF, Pyles RB, Miller AL, Nokso-Koivisto J, Chonmaitree. Viral-Bacterial Interactions and Risk of Acute Otitis Media Complicating Upper Respiratory Tract Infection. *J Clin Microbiol*. 2011;49(11):3750–5.
5. Chonmaitree T, Revai K, Grady JJ, Clos A, Patel JA, Nair S, et al. Viral upper respiratory tract infection and otitis media complication in young children. *Clin Infect Dis*. 2008;46(6): 815–23.
6. Umar S, Restuti RD, Suwento R, Priyono H, Mansyur M. The prevalence and risk factors of acute otitis media in children in the municipality of East Jakarta [Prevalensi dan faktor risiko otitis media akut pada anak–anak di kotamadya Jakarta Timur]. http://lib.ui.ac.id/naskahringkas/2015-09/SP-Sakina%20Umar. Published 2013. Accessed February 20, 2016.
7. Britt H, Miller GC, Henderson J, Bayram C, Valenti L, Harrison C, et al. A decade of Australian General Practice Activity 2005-06 to 2014-15. General practice series no. 39. Sydney: Sydney University Press; 2015.
8. Lieberthal AS, Carroll AE, Chonmaitree T, Ganiats TG, Hoberman A, Jackson MA et al. Clinical Practice Guideline: The diagnosis and management of acute otitis media. The American Academy of Pediatrics. *Pediatrics.* 2013;131:e964-e99.
9. Morris PS, Leach AJ. Managing otitis media: an evidence-based approach. *Aust Prescr.* 2009;32:155–9.
10. Le Saux N, Robinson JL, Canadian Paediatric Society Infectious Diseases and Immunization Committee. Management of acute otitis media in children six months of age and older. *Paediatr Child Health*. 2016;21(1):39-44.
11. Rovers MM, Glasziou P, Appelman CL, Burke P, McCormick DP, Damoiseaux RA, et al. Antibiotics for acute otitis media: a meta-analysis with individual patient data. *Lancet*. 2006;368:1429-35
12. Ministry of Health Republic of Indonesia. Clinical practice guidelines for clinicians in primary healthcare centres. Jakarta: Ministry of Health Republic of Indonesia;2014. Regulatory No. 5 year 2014.
13. Costelloe C, Metcalfe C, Lovering A, Mant David, Hay AD. Effect of antibiotic prescribing in primary care on antimicrobial resistance in individual patients: systematic review and meta-analysis. *BMJ.* 2010;340:c2096. doi: 10.1136/bmj.c2096
14. Venekamp RP, Sanders SL, Glasziou PP, Del Mar CB, Rovers MM. Antibiotics for acute otitis media in children. Cochrane Database of Systematic Reviews 2015, Issue 6. Art. No.: CD000219. DOI: 10.1002/14651858.CD000219.pub4.
15. Marom T, Marchisio P, Tamir SO, Torretta S, Gavriel H, Esposito S. Complementary and alternative medicine treatment options for otitis media. *Medicine*. 2016;95(6):e2695

16. Coleman C, Moore M. Decongestants and antihistamines for acute otitis media in children. Cochrane Database of Systematic Reviews 2011, Issue 3. Art. No.: CD001727. DOI: 10.1002/14651858.CD001727.pub5.
17. Juhn SK, Jung MK, Hoffman MD, Drew BR, Preciado DA, sausen NJ, et al. The role of inflammatory mediators in the pathogenesis of otitis media and sequelae. *Clin Exp Otorhinolaryngol*. 2008;1(3):117-38.
18. Chonmaitree T, Saeed K, Uchida T, Heikkinen T, Baldwin CD, Freeman DH, et al. A randomised, placebo-controlled trial of the effect of antihistamine of corticosteroid treatment in acute otitis media. *J Pediatr*. 2003;143:377-85.
19. Schimmer BP, Funder JW. ACTH, adrenal steroids, and pharmacology of the adrenal cortex. In: Brunton LL, Chabner BA, et al., eds. *Goodman & Gilman's The Pharmacological Basis of Therapeutics*. 12th Ed. New York: McGraw-Hill; 2011:1209-36.
20. Gupta P, Bhatia V. [Symposium on steroid therapy] Corticosteroid physiology and principles of therapy. *Indian J Pediatr.* 2008;75(10):1039-44.
21. Indonesian Pediatric Society. Juvenile rheumatoid arthritis. In: Pudjiadi AH, Hegar B, Handryastuti S, et.al, eds. Clinical guideline Indonesian Pediatric Society. 2nd edition. Jakarta; Indonesian Pediatric Society Publishing; 2011:1-4.
22. Indonesian Pediatric Society. Acute bronchial asthma. In: Pudjiadi AH, Hegar B, Handryastuti S, et.al, eds. Clinical guideline Indonesian Pediatric Society Jakarta; Indonesian Pediatric Society Publishing;.2009:269-73.
23. Indonesian Pediatric Society. Bacterial meningitis. In: Pudjiadi AH, Hegar B, Handryastuti S, et.al, eds. Clinical guideline Indonesian Pediatric Society Jakarta; Indonesian Pediatric Society Publishing; 2009:189-92.
24. British Thoracic Society/Scottish Intercollegiate Guidelines Network. British guideline on the management of asthma: a national clinical guideline. British Thoracic Society/Scottish Intercollegiate Guidelines Network. Revised edition; 2016:107.
25. Global Initiative for Asthma. Global Strategy for Asthma Management and Prevention, 2016. www.ginasthma.org. Updated 2016. Accessed September 2, 2016.
26. Ruohola A, Heikkinen T, Jero J, Puhakka T, Juvén T, Närkiö-Mäkelä M, et al. Oral prednisolone is an effective adjuvant therapy for acute otitis media with discharge through tympanostomy tubes. *J Pediatr*. 1999;134:459-63.
27. McCormick DP, Saeed K, Uchida T, Baldwin CD, Deskin R, Lett-Brown MA, et al. Middle ear fluid histamine and leukotriene B4 in acute otitis media: effect of antihistamine or corticosteroid treatment. *Int J Pediatr Otorhinolaryngol.* 2003;67(3):221–30.
28. Waldron CA, Thomas-Jones E, Cannings-John R, Hood K, Powell C, Roberts A, et al. Oral steroids for the resolution of otitis media with effusion (OME) in children (OSTRICH): study protocol for a randomised controlled trial. *Trials*. 2016;17:115.
29. Melhus A, Ryan AF. Expression of cytokine genes during pneumococcal and nontypeable haemophilus influenzae acute otitis media in rat. *Infection and Immunity*. 2000;68(7):4024-31.
30. Aljebab F, Choonara I, Conroy S. *Arch Dis Child* Published Online First: 3 February 2017. DOI:10.1136/archdischild-2015-309522.
31. Panickar J, Lakhanpaul M, Lambert PC, Kenia P, Stephenson T, Smyth A, et al. Oral prednisolone for preschool children with acute virus-induced wheezing. *N Engl J Med*. 2009;360(4):329-38.
32. Scarfone RJ, Fuchs SM, Nager AL, Shane SA. Controlled trial of oral prednisone in the emergency department treatment of children with acute asthma. *Pediatrics*. 1993;92(4):513-8.
33. Scarfone R J, Loiselle JM, Wiley JF II, Decker JM, Henretig FM, Joffe MD. Nebulized dexamethasone versus oral prednisone in the emergency treatment of asthmatic children. *Ann Emerg Med*. 1995;26:480-6.
34. Kayani S, Shannon DC. Adverse behavioural effects of treatment for acute exacerbation of asthma in children. A comparison of two doses of oral steroids. *Chest*. 2002;122:624-8.

35. Chang AB, Sloots TP, Petsky HL, Thearle D, Champion AA, Wheeler C, et al. A 5- versus 3-day course of oral corticosteroids for children with asthma exacerbations who are not hospitalised: a randomised controlled trial. *MJA*. 2008;189(6):306-10.
36. Ranakusuma RW, Pitoyo Y, Safitri ED, Thorning S, Beller EM, Sastroasmoro S, et al. Systemic corticosteroids for acute otitis media in children. *Cochrane Database of Systematic Reviews* 2016, Issue 7. Art. No.: CD012289. DOI:10.1002/14651858.CD012289.
37. Denhoff ER, Milliren CE, de Ferranti SD, Steltz SK, Osganian SK. Factors Associated with Clinical Research Recruitment in a Pediatric Academic Medical Center—A Web-Based Survey. PLoS ONE. 2015;10(10): e0140768. doi:10.1371/journal. pone.0140768.
38. Rosenfeld RM, Shin JJ, Schwartz SR, Coggins R, Gagnon L, Hackell JM, et al. Clinical practice guideline: Otitis media with effusion (update). *Otorlaryngology-Head and Neck Surgery.* 2016;154(1S):S1-S41.
39. Cocks K, Torgerson DJ. Sample size calculations for pilot randomized trials: a confidence interval approach. *J Clin Epidemiol.* 2013;66:197-201.
40. Nozza RJ, Bluestone CD, Kardatzke D, Bachman R. Identification of middle ear effusion by aural acoustic admittance and otoscopy. *Ear Hear*. 1994;15:310-23.
41. Huguet A, Stinson JN, McGrath PJ. Measurement of self-reported pain intensity in children and adolescents. *J Psychosom Res*. 2010;68:329-36.
42. Cohen LL, Lemanek K, Blount RL, et al. Evidence-based assessment of paediatric pain. *J Pediatr Psychol*. 2008;33(9):939-56.
43. Von Baeyer C. Children's self-report of pain intensity: What we know, where we are headed. *Pain Res Manag.* 2009;14(1):39-45.
44. Powell CV, Kelly AM, Williams A. Determining the minimum clinically significant difference in visual analogue pain score for children. *Ann Emerg Med.* 2001;37(1):28-31.
45. Shaikh N, Hoberman A, Paradise JL, et al. Responsiveness and construct validity of a symptom scale for acute otitis media. *Pediatr Infect Dis J*. 2009:28(1):9–12.

**Appendix 1. Patient flow chart**

**Baseline Visit (Visit-0)**

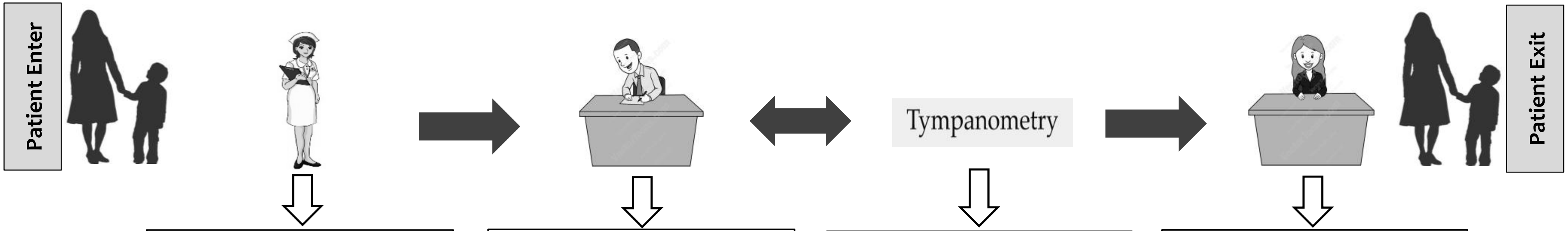


**Attending Nurse**

- Identifying potential AOM patients using the checklist on the study registration log book
- Preparing the case report form (CRFs)
- Collecting data of body weight, height, body temperature, and blood pressure
- Preparing and handing over the study document (I.e. study recruitment log book, CRFs) to the physician

**Physician**

- Eligibility assessment
- Stratification process
- Baseline history taking
- Outcomes assessment using CRF and otoscope
- Providing a symptom diary and training
- Providing study medication prescriptions
- Checking and interpreting the tympanometry findings

**Audiologist**

- Outcomes assessment using tympanometry

**Appointed nurse**

- Randomisation
- Study medication prescription dispensing
- Study document collection and storage
- List the follow-up visits on the follow-up reminder card
- Hand over the study bag
- Reimbursing the transport costs

**Other visits (Visit-1 to Visit-4)**

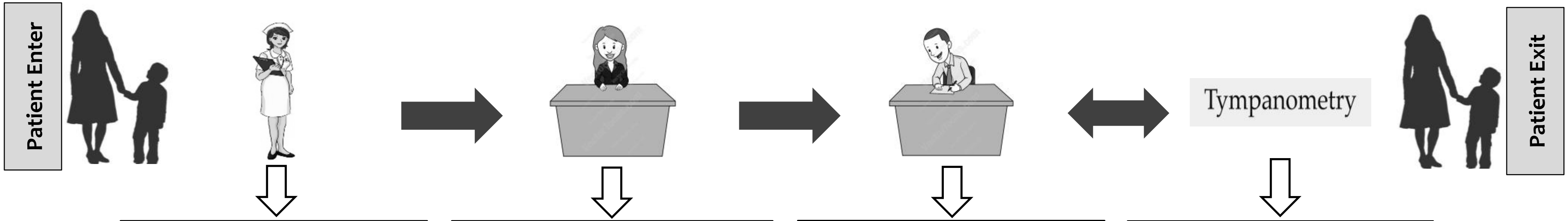


**Attending Nurse**

- Collecting data of body weight, height, body temperature, and blood pressure
- Preparing and handing over the case report form (CRFs) to the appointed nurse

**Appointed Nurse**

- Checking the completion of study document
- Identification adverse effects and complications
- Checking the symptom diary
- Checking the number of study medication, whether they will be available for the next two days
- Completing the follow-up reminder card
- Collecting the first mini-booklet of symptom diary and provide the second mini booklet at Visit-1
- Handing over the study document and reporting any adverse effects or complications to the physician
- Collecting the second mini-booklet of symptom diary and provide the third mini booklet at Visit-2
- Collecting the left-over drug (prednisolone group) at Visit–2 and completing the drug dispensing and returning form

**Physician**

- Identification adverse effects and complications
- Outcomes assessment using CRF and otoscope
- Checking the symptom diary
- Checking and interpreting the tympanometry findings
- Checking the additional medication

**Audiologist**

- Outcomes assessment using tympanometry

# PARTICIPANT INFORMATION SHEET AND CONSENT FORM

## Oral prednisolone for acute otitis media in children: a pilot pragmatic randomised open-label single-blind controlled study (OPAL study)

## [Steroids for middle ear infection in children]

## Invitation

You are invited to participate in a research study into the use of steroids (prednisolone) or an anti-inflammatory drug for middle ear infection in children.

The study is being conducted by Dr. Respati W. Ranakusuma, an otorhinolaryngologists and a researcher at the Clinical Epidemiology and Evidence-Based Medicine (CEEBM) Unit Dr. Cipto Mangunkusumo Hospital–Faculty of Medicine Universitas Indonesia. This is part of an international collaborative study between CEEBM CMH-FMUI and the Centre for Research in Evidence-Based Practice (CREBP), Faculty of Health Sciences and Medicine Bond University, Queensland, Australia.

Before you decide whether or not you wish to participate in this study, it is important for you to understand why the research is being done and what it will involve. Please take the time to read the following information carefully and discuss it with others if you wish.

1. **What is the purpose of this study?**
The purpose is to investigate whether steroids, as an alternative treatment, will reduce ear pain and other symptoms in children with acute or recent (less than 48 hours) middle ear infection. This study is part of a doctoral project at the CREBP Bond University, Queensland, Australia. As this is a pilot study, we also want to know your experience during the study. For example, the obstacles you found in giving the steroid to your child or completing the symptom diary daily.

2. **Why have my child and I been invited to participate in this study?**
Your child and you have been invited to participate in this study because your child age ranges between six months to 12 years and having symptoms and signs of acute middle ear infection, such as ear pain in the past 48 hours, or holding or tugging her/his ear more frequently, more irritable, show lack of playfulness and/sleep in a young age (baby). If visible, from the ear examination, the ear drum(s) will show redness or yellowish, bulging, or discharge.

3. **What does participation in this study involve?**
If you agree to participate in this study, your physician will ask you more questions regarding the history of your child's previous infection, allergy, and the severity of the symptoms (e.g. ear pain, fever, disruption of daily activities). As only your child and you as the parents know the best of how severe the symptoms are, we will ask you to show the severity of the symptoms using two tools. The first tool is called visual analogue scale. It is a 10-cm horizontal line, whereas the left end of the line represents 'no pain' and the right end represents 'the most painful'. We will ask you to draw a vertical line across this line at the point that represents how bad

the symptom that your child has been experiencing. The second tool is called acute otitis media – the severity of symptoms (AOM-SOS) that consisted of seven questions. You will be asked to choose one of the severity scales ('no', 'a little', or 'a lot') that corresponds to seven particular symptoms (i.e. tugging/rubbing the ears, crying more, more irritable, lack of sleep, playfulness, and appetite, and fever). Whilst you providing your best answers using these tools, your physician will also teach you to complete the symptom diary that consists similar questions that your physician has been obtained from you. This will help you in completing the symptom diary during the study which will help us to investigate the effect of the steroid in improving your child' ear pain and other symptoms due to acute middle ear infection. After that, your attending nurse and physician will examine your child's general status (i.e. body weight, height, body temperature, blood pressure) and ear-nose-throat status. From there, we will check the condition of your child's middle ear using a tool called tympanogram. This is a painless procedure to detect whether there is a fluid in your child's middle ear. From there, you will meet a nurse who will allocate your child whether she/he will receive the steroid (treatment group) or not receive the steroid (control group). Your child has 50% chance for being allocated to receive the steroid. We will do this process randomly where no one can predict in which group your child will be allocated to. This process will require 15 to 30 minutes because the nurse has to access this information from the website or calling the research team. If your child receives the steroid, she will give you a prescription for your study medication. You will give the prescription to the pharmacy at that hospital. The pharmacist will prepare your study medication by crushing the tablets, mixing it with sweeteners, and packing the study medication in a daily paper-package (you will receive five daily packages). The nurse will give an instruction to give a medication to your child every morning, once daily for 10 to 30 milligrams depends on your child's age, for five days. She will tell you what to do if your child vomits after taking a drug or experiences any effects. She also will ask you to keep the confidentiality of the treatment that your child receives from your physician and audiologist. The whole process will require 60 to 120 minutes depends on the cooperativity of your child. We will ask you to come after two and seven days after your visit. On these visits, we will investigate whether the steroid will help reducing the ear pain and other relevant symptoms and whether it give unfavorable effects. During these visits, we will ask you to bring the symptom diary and the left-over drug so we can check your child' condition. We also will ask you to come after one and three months to see whether during these time, your child experiences a new episode of acute middle ear infection. After these four additional visit after this visit, we consider that your child has completed the study.

Any information obtained in connection with this research project that can identify you child and you will remain confidential. If you agree to participate in this study, you will be asked to sign the Participant Consent Form.

**4. What if I do not want to take part in this study, or if I want to withdraw later?**

Participation in this study is voluntary. It is completely up to you or both of you and your child if you child aged 12 years, whether or not you participate. If you decide not to participate, it will not affect the treatment your child receive now or in the future. Whatever your decision, it will not affect your relationship with the staff caring for your child. However, it may not be possible to withdraw your data from the study results if these have already had your identifying details removed.

5. **How is this study being paid for?**
   The study is being for by Dr. Respati W. Ranakusuma, ORL which is supported by self-funded.

6. **Are there risks to my child in taking part in this study?**
   The foreseeable risks in taking part in this study are the bitter taste of prednisolone tablets and some potential side effects of the steroids. Pharmacist will mix the crushed tablets with sweeteners and we will also provide honey to be mixed with the medication. The common potential side effects of steroids are nausea, vomiting, abdominal pain, nervousness, mood swings, headache, increased blood sugar and blood pressure, weight gain, etc. Growth disorder could be one of the side effects however it usually occurs on the longer use of the steroids. We cannot predict whether your child will have one of these effects or not at all.

   You may feel that the whole process of this study will take longer time compared to usual doctor visit due to collection of information and additional examination that will be conducted in this study. It may add some work for you to complete a symptom diary daily for the next 14 days. However, this is very important to be able to assess the day-by-day progress of your child with or without the steroids. Other potential inconveniences that your child and you may experience from this study are during the tympanometry examination and the follow-up visits (four additional visits are required in this study). Even though tympanometry is a painless procedure, we expect that your child will sit still for at least 10 minutes where she/he will hear a ringing sound and a pressure sensation during the process.

7. **What happens if my child suffers injury or complications as a result of the study?**
   If you require treatment or suffer loss as a result of the negligence of any of the parties involved in the study, you may be entitled to compensation; the cost of your treatment would have to be paid out of such compensation.

8. **Will I benefit from the study?**
   This study aims to further medical knowledge and may improve future treatment of acute middle ear infection (especially in mild cases where usually antibiotics are being prescribed), however, this study may not directly benefit you.

9. **Will taking part in this study cost me anything, and will I be paid?**
   Participation in this study will not cost you anything, nor you will be paid. You will be reimbursed for reasonable travel expenses to the amount of $15. We also will cover the registration and consultation fees for the additional four follow-up visits to the hospital.

10. **How will my confidentiality be protected?**
    Any identifiable information that is collected about your child in connection with this study will remain confidential and will be disclosed only with your permission, or except as required by law. Only the researchers named above will have access to your details and results that will be held securely at the CEEBM CMH – FMUI.

**11. What happens with the results?**

If you give us your permission by signing the consent document, we plan to discuss/publish the results for the monitoring and safety purposes (by the Human Research Ethics Committee, data monitoring and auditing committee, if necessary) and for publication in peer-reviewed journals or presentation at conferences or other professional forums. In any publication, information will be provided in such a way that you cannot be identified.

**12. What should I do if I want to discuss this study further before I decide?**

When you have read this information, your physician as one of the researchers, will discuss it with you and any queries you may have. If you would like to know more at any stage, please do not hesitate to contact Dr. Respati W. Ranakusuma, ORL by phone on +62 8111 012 185.

**13. Who should I contact if I have concerns about the conduct of this study?**

This study has been approved by the Medical Ethics Committee FMUI and the Bond University's Human Research Ethics Committee (BUHREC) Bond University, Queensland, Australia. Any person with concerns or complaints about the conduct of this study should contact Dr. Respati W. Ranakusuma on +62 8111 012 185, or email OPAL.study@bond.edu.au.

The conduct of this study at (please circle the answer that representing your hospital) the Dr Cipto Mangunkusumo Hospital / Persahabatan Hospital / Gatot Subroto Army Hospital / Antam Medika Hospital / Cempaka Putih Islamic Hospital / Proklamasi ENT Hospital / Hermina Bekasi Hospital, has been authorised by the the Health Agency for the Province of DKI Jakarta and the Directorate-General for Politics and General Government – The Ministry of Internal Affairs Republic Indonesia.

**Thank you for taking the time to consider this study. If you wish to take part in, please sign the attached consent form. This information sheet is for you to keep**

# CONSENT FORM

## Oral prednisolone for acute otitis media in children: a pilot pragmatic, randomised, open-label, single-blind study (OPAL study)
## [Steroids for middle ear infection in children]

1. I, ______________________________________________________________________
   of ____________________________________________________________________
   agree to participate in the study described in the participant information statement set attached to this form.
2. I acknowledge that I have read the participant information statement, which explains why my child has been selected, the aims of the study, and the nature and the possible risks of the investigation, and the statement has been explained to me to my satisfaction.
3. Before signing this consent form, I have been given the opportunity of asking any questions relating to any possible physical and mental harm my child might suffer as a result of my child participation and I have received satisfactory answers.
4. I understand that I can withdraw from the study at any time without prejudice to my relationship to my physician and the ___________________________________ Hospital.
5. I agree that research data gathered from the results of the study may be published, provided that I cannot be identified.
6. I understand that I have any questions relating to my participation in this research, I may contact Dr. Respati W. Ranakusuma, ORL on telephone +62 8111 012 185, who will be happy to answer them.
7. I acknowledge receipt of a copy of this Consent Form and the Participation Information Statement.

Complaints may be directed to the OPAL Study Support Office at the Clinical Epidemiology and Evidence-Based Medicine Unit, Dr Cipto Mangunkusumo Hospital – Faculty of Medicine Universitas Indonesia, Building H Dr Cipto Mangunkusumo Hospital, Diponegoro 71, Jakarta 10430, Indonesia (phone +62 21 316 1760, email OPAL.study@bond.edu.au).

| **Signature of participant or the parent** | **Name** | **Date** |
|---|---|---|
| ______________________ | ______________________ | ______________________ |
| **Signature of witness** | **Name** | **Date** |
| ______________________ | ______________________ | ______________________ |
| **Signature of investigator** | **Name** | **Date** |
| ______________________ | ______________________ | ______________________ |

# REVOCATION OF CONSENT

## Oral prednisolone for acute otitis media in children: a pilot pragmatic, randomised, open-label, single-blind study (OPAL study)

## [Steroids for middle ear infection in children]

I hereby wish to WITHDRAW my consent to participate in the study described above and understand that such withdrawal WILL NOT jeopardise any treatment or my relationship with the ____________________________ hospital or my medical attendants.

| **Signature of participant or the parent** | **Name** | **Date** |
|---|---|---|
| ______________________ | ______________________ | ______________________ |

The section for Revocation of Consent should be forwarded to Dr. Respati W. Ranakusuma, ORL at the Clinical Epidemiology and Evidence-Based Medicine Unit, Dr Cipto Mangunkusumo Hospital – Faculty of Medicine Universitas Indonesia.

REGISTRATION ID

# CRF02. STUDY REGISTRATION FORM

## PATIENT'S INFORMATION

| | |
|---|---|
| Patient's name | ______________________ |
| Place and date of birth | __________ , ______________ |
| Education | O None O Pre-school O Elementary school<br>O Middle junior school |
| School attending hours | O 1x / week from: _____ am/pm to _____ pm/pm<br>O 2x / week from: _____ am/pm to _____ pm/pm<br>O 3x / week from: _____ am/pm to _____ pm/pm<br>O 4x / week from: _____ am/pm to _____ pm/pm<br>O ≥ 5x / week from: _____ am/pm to _____ pm/pm |
| Home address | ______________________<br>______________________<br>______________________ |
| Home telephone number | ______________________ |
| Home fax number | ______________________ |
| Health service payment | O self-payment O Private insurance O Company insurance<br>O Government health coverage (BPJS) O Other: __________ |

| | | | |
|---|---|---|---|
| Weight: ______ kg | Height: _______ cm | Temperature: _____ °C | Blood pressure: ____________ mmHg |

## PARENTS' INFORMATION

### FATHER

| | |
|---|---|
| Father's name | ______________________ |
| Place and date of birth | __________ |
| Education | O None O Elementary school O Middle junior school<br>O High school O Bachelor O Masters<br>O Doctoral |
| Occupation | O None O Government employee O Private employee |

| | |
|---|---|
| | O Entrepreneur O Other: __________ |
| Home address | O Same with patient's address<br>O Different address: ____________________<br>____________________ |
| Home telephone number | O Same with patient's telephone number<br>O Different number: ____________________ |
| Mobile number | ____________________ |
| Email address | ______ ____________________ |
| **MOTHER** | |
| Mother's name | ____________________ |
| Place and date of birth | ____________________ |
| Education | O None O Elementary school O Middle junior school<br>O High school O Bachelor O Masters<br>O Doctoral |
| Occupation | O None O Government employee O Private employee<br>O Entrepreneur O Other: __________ |
| Home address | O Same with patient's address<br>O Different address: ____________________<br>____________________ |
| Home telephone number | O Same with patient's telephone number<br>O Different number: ____________________ |
| Mobile number | ____________________ |
| Email address | ____________________ |

Date : |___|___| – |___|___| – 201 |___|

Registration ID

Doctor ID : |___|___|___|

Hospital ID : |___|___|

# CRF03 – ELIGIBILITY FORM

## INCLUSION CRITERIA

| | |
|---|---|
| OYes ONo | Definite or suspected acute otitis media (AOM)<br>OR<br>Were you able to confirm otoscopically? OYes ONo |
| OYes ONo | Aged 6 months to 12 years |
| OYes ONo | Available for follow-up visits |

## EXCLUSION CRITERIA

| | |
|---|---|
| OYes ONo | Major medical conditions (e.g. heart failure, renal insufficiency, DM, peptic ulcers) |
| OYes ONo | Immunocompromised (e.g. cancer treatment, HIV) |
| OYes ONo | Congenital malformation/syndromes (e.g. cleft palate) |
| OYes ONo | Ventilation tube(s) |
| OYes ONo | Exposed to persons with varicella/active Zoster infection in the past 3 weeks with no prior history of varicella infection/ immunisation |
| OYes ONo | With high risk of strongyloidiasis infection |
| OYes ONo | Has taken oral/injection/topical steroids in the past 4 weeks |
| OYes ONo | Has taken antibiotics in the past 2 weeks |
| OYes ONo | Hypersensitive to prednisolone or other steroids |

**Is this child eligible for the trial?**

All 'YES' at the inclusion criteria, AND
All 'NO' at the exclusion criteria

**Eligible, then INCLUDE** O

At least one 'NO' at the inclusion criteria, OR
At least one 'YES' at the exclusion criteria

**Not eligible, then EXCLUDE** O

**Obtaining the CONSENT**

NOT giving CONSENT → **EXCLUDE** O

Giving CONSENT → **INCLUSION** O

**Do they have these following symptoms?**

| | |
|---|---|
| OYes ONo | Moderate to severe symptoms, locally or systemically (moderate to severe ear pain, fever ≥39°C, complications) |
| OYes ONo | Aged younger than 2 years with bilateral acute otitis media |
| OYes ONo | With perforation of tympanic membrane(s) |
| OYes ONo | If visible, otoscopic finding shows moderate to severe bulging and/or yellowish purulent tympanic membrane(s) |

At least one 'YES' → **SEVERE AOM** O

All 'NO' → **MILD AOM** O

For each question, please tick (V) your answer in the circle

**Registration ID** | |

## CRF04 – BASELINE INFORMATION FORM

1 Did (do) you breastfeed your child? O Yes O No

If ' YES', until the age of O ≤ 2 months O > 2 – 6 months O > 6 months

2 Does your child attend a day-care O Yes O No

How many days in a week? O ≤ 2 days O > 2 days

3 Have your child had a pneumococcus vaccine (PCV)? O Yes O No O Do not know

How many times: __________ times

4 Have your child had an influenzae vaccine? O Yes O No O Do not know

How many times: __________ times

5 How many episodes of recurrent acute respiratory infection (runny nose, cough, sore throat, fever) in the past year? O ≤ 3 episodes O > 3 episodes to 6 episodes O > 6 episodes

6 Did your child have a history of 3 or more episodes of ear infection (ear pain, ear discharge, diarrhoea, or vomiting) during the past 12 months? O Yes O No

7 At what age did the first episode of ear infection start?

O ≤ 6 months O > 6 to 12 months O >12 to 24 months O > 2 to 5 years O > 5 years

8 Does your child have one of the following disorders:

O Bronchial asthma

O Allergic rhinitis

O Family history of atopic disorders

O None of above

9 Number of children (including the patient) who live in the house ___________ children

10 Number of persons who smoke at home ___________ person(s)

For each question, please tick (✓) your answer on the circle or write you answer on ______

# CRF05 – OUTCOME FORM

## Baseline Visit (Day-0) : |___|___| – |___|___| – 20 |___|___|

### Complications (for Physician)

| | | |
|---|---|---|
| 1 | **Does your child experience discharge from the ear(s)?** | O Yes O No |
| 2 | **Does your child experience intense ear pain and pain behind the ear?** | O Yes O No |
| 3 | **Does your child experience swelling/bulging/ or redness/tenderness of the ear(s)?** | O Yes O No |
| 4 | **Does your child experience facial asymmetry (e.g. when the child smiles, cries)?** | O Yes O No |

### General and ENT examination (for Nurse and Physician)

5.1 **Weight** _____ kg | 5.2 **Height** _______ cm | 5.3 **Temp.** ________ °C | 5.4 **BP** _____ / _____ mmHg

6 **Nose** O Normal O Oedema O Hyperaemic O Livid O Serous discharge O Mucoid discharge

7 **Tonsils** O Normal O Hyperaemic O Detritus O Tonsil(s) T1 O Tonsil(s) T2 O Tonsil(s) T3-4

8 **Pharynx** O Normal O Hyperaemic O Oedema O Granules O Post nasal drip (PND)

### 9 Otoscopic examination

O Normal O Cerumen O Erythema O Air fluid level O Complete effusion O Opacification

O Mild bulging O Moderate to severe bulging (bulging rounded) O Bulla O Perforation

### 10 Medicines that have been taken before the baseline visit (please circle your dose measurement)

| | | | |
|---|---|---|---|
| 1. | ______________________ | Dose : _____mg perBw kg / Teaspoon / Tablespoon ; | Frequency : ____ / day |
| 2. | ______________________ | Dose : _____mg perBW kg / Teaspoon / Tablespoon ; | Frequency : ____ / day |
| 3. | ______________________ | Dose : _____mg perBW kg / Teaspoon / Tablespoon ; | Frequency : ____ / day |
| 4. | ______________________ | Dose : _____mg perBW kg / Teaspoon / Tablespoon ; | Frequency : ____ / day |
| 5. | ______________________ | Dose : _____mg perBW kg / Teaspoon / Tablespoon ; | Frequency : ____ / day |

### Medicines prescribed by physician (you) at the baseline visit

| | |
|---|---|
| Antibiotic | ______________________<br>Dose : _______mg / BW kg Frequency : _______ / day for _______ days |

Other medicine(s)

| | | | |
|---|---|---|---|
| 1. | ______________________ | Dose : _____mg perBw kg / Teaspoon / Tablespoon ; | Frequency : ____ / day |
| 2. | ______________________ | Dose : _____mg perBw kg / Teaspoon / Tablespoon ; | Frequency : ____ / day |
| 3. | ______________________ | Dose : _____mg perBw kg / Teaspoon / Tablespoon ; | Frequency : ____ / day |
| 4. | ______________________ | Dose : _____mg perBw kg / Teaspoon / Tablespoon ; | Frequency : ____ / day |
| 5. | ______________________ | Dose : _____mg perBw kg / Teaspoon / Tablespoon ; | Frequency : ____ / day |

### Outcome: Symptoms (for patients and the parents. Physician will help them to complete these in the symptom diary)

**11 Please place a vertical line across the available horizontal line that best describes your or your child's pain during the past 24 hours?**

No Pain |————————————————| Pain As Bad As It Could Possibly Be

*For each question, please tick (✓) your answer on the circles or write you answer on ______*

| 12 We are interest finding out how your child has been doing. For each question, please place a checkmark (V) in the circle corresponding to your child's symptoms. Please answer all questions. | | | |
|---|---|---|---|
| 12.1 Over the past 12 h, has your child been tugging, rubbing, or holding the ear(s) more than usual? | O No | O A little | O A lot |
| 12.2 Over the past 12 h, has your child been crying more than usual? | O No | O A little | O A lot |
| 12.3 Over the past 12 h, has your child been more irritable or fussy than usual? | O No | O A little | O A lot |
| 12.4 Over the past 12 h, has your child been having more difficulty sleeping than usual? | O No | O A little | O A lot |
| 12.5 Over the past 12 h, has your child been less playful or active than usual? | O No | O A little | O A lot |
| 12.6 Over the past 12 h, has your child been eating less than usual? | O No | O A little | O A lot |
| 12.7 Over the past 12 h, has your child been having fever or feeling warm to touch? | O No | O A little | O A lot |

**13 Tympanometry examination (for Audiologist and interpreted by Physician)**

O Cannot be performed. Reason: ____________________

Tympanogram types (will be completed by physician) [R] Type ______ / [L] Type ______

Ear canal vol (ECV) [R] ________ mL / [L] ________ mL

Static acoustic admittance [R] _______ mL / [L] _______ mL

Compliance (SC) [R] ________ mL / [L] ________ mL

Middle Ear Pressure or TPP [R] _______ daPa / [L] ______ daPa

Gradient or TW [R] _______ daPa / [L] ______ daPa

*Put the copy of tympanometry copies here*

**Registration ID** | |

## Follow-up Visit – 1 (Day – 3) : |___|___| – |___|___| – 20 |___|___|

### Complications (for Physician)

| | | |
|---|---|---|
| 1 | **Does your child experience discharge from the ear(s)?** | O Yes O No |
| 2 | **Does your child experience intense ear pain and pain behind the ear?** | O Yes O No |
| 3 | **Does your child experience swelling/bulging/ or redness/tenderness of the ear(s)?** | O Yes O No |
| 4 | **Does your child experience facial asymmetry (e.g. when the child smiles, cries)?** | O Yes O No |

### General and ENT examination (for Nurse and Physician)

| | | | |
|---|---|---|---|
| 5.1 **Weight** _____ kg | 5.2 **Height** _______ cm | 5.3 **Temp.** _________ °C | 5.4 **BP** _____ / _____ mmHg |

6 **Nose** O Normal O Oedema O Hyperaemic O Livid O Serous discharge O Mucoid discharge

7 **Tonsils** O Normal O Hyperaemic O Detritus O Tonsil(s) T1 O Tonsil(s) T2 O Tonsil(s) T3-4

8 **Pharynx** O Normal O Hyperaemic O Oedema O Granules O Post nasal drip (PND)

### 9 Otoscopic examination

O Normal O Cerumen O Erythema O Air fluid level O Complete effusion O Opacification

O Mild bulging O Moderate to severe bulging (bulging rounded) O Bulla O Perforation

### 10 Medicines prescribed by you (Physician) on today visit (please circle your dose measurement)

| | |
|---|---|
| Antibiotic | Dose : _______mg / BW kg Frequency : _______ / day for _______ days |

Other medicine(s)

| | | |
|---|---|---|
| 1. | _______________________ | Dose : _____mg perBw kg / Teaspoon / Tablespoon ; Frequency : ____ / day |
| 2. | _______________________ | Dose : _____mg perBw kg / Teaspoon / Tablespoon ; Frequency : ____ / day |
| 3. | _______________________ | Dose : _____mg perBw kg / Teaspoon / Tablespoon ; Frequency : ____ / day |
| 4. | _______________________ | Dose : _____mg perBw kg / Teaspoon / Tablespoon ; Frequency : ____ / day |
| 5. | _______________________ | Dose : _____mg perBw kg / Teaspoon / Tablespoon ; Frequency : ____ / day |

### 11 Medicines NOT prescribed by you or from over-the-counter or others (e.g. other physician, drug store)

| | |
|---|---|
| Antibiotic | Dose : _______mg / BW kg Frequency : _______ / day for _______ days |

Other medicine(s)

| | | |
|---|---|---|
| 1. | _______________________ | Dose : _____mg perBw kg / Teaspoon / Tablespoon ; Frequency : ____ / day |
| 2. | _______________________ | Dose : _____mg perBw kg / Teaspoon / Tablespoon ; Frequency : ____ / day |
| 3. | _______________________ | Dose : _____mg perBw kg / Teaspoon / Tablespoon ; Frequency : ____ / day |
| 4. | _______________________ | Dose : _____mg perBw kg / Teaspoon / Tablespoon ; Frequency : ____ / day |
| 5. | _______________________ | Dose : _____mg perBw kg / Teaspoon / Tablespoon ; Frequency : ____ / day |

### Outcome: Symptoms (for Patients)

**12 Please place a vertical line across the available horizontal line that best describes your or your child's pain during the past 24 hours?**

|——————————————————————————————|

No Pain — Pain As Bad As It Could Possibly Be

| **13. We are interest finding out how your child has been doing. For each question, please place a checkmark (V) in the circle corresponding to your child's symptoms. Please answer all questions.** | | | |
|---|---|---|---|
| 13.1 Over the past 12 h, has your child been tugging, rubbing, or holding the ear(s) more than usual? | O No | O A little | O A lot |
| 13.2 Over the past 12 h, has your child been crying more than usual? | O No | O A little | O A lot |
| 13.3 Over the past 12 h, has your child been more irritable or fussy than usual? | O No | O A little | O A lot |
| 13.4 Over the past 12 h, has your child been having more difficulty sleeping than usual? | O No | O A little | O A lot |
| 13.5 Over the past 12 h, has your child been less playful or active than usual? | O No | O A little | O A lot |
| 13.6 Over the past 12 h, has your child been eating less than usual? | O No | O A little | O A lot |
| 13.7 Over the past 12 h, has your child been having fever or feeling warm to touch? | O No | O A little | O A lot |

**14 Side effects**

**Does your child have these complaints after taking the medicine**

| | | | | | |
|---|---|---|---|---|---|
| 14.1 Increased appetite | O Yes | O No | 14.8 Drowsiness | O Yes | O No |
| 14.2 Increased urine amount | O Yes | O No | 14.9 Anxiety/distractibility/mood swing | O Yes | O No |
| 14.3 Weight gain | O Yes | O No | 14.10 Headache | O Yes | O No |
| 14.4 Gastritis/abdominal pain | O Yes | O No | 14.11 Skin rash or diaper rash | O Yes | O No |
| 14.5 Nausea | O Yes | O No | 14.12 Candidiasis | O Yes | O No |
| 14.6 Vomiting | O Yes | O No | 14.13 Dry mouth / throat irritation | O Yes | O No |
| 14.7 Diarrhea | O Yes | O No | 14.14 Sleep disturbance | O Yes | O No |

Others: ________________________________________________

| | | | |
|---|---|---|---|
| Did you bring your child to doctor (clinic or outpatient)? | O Yes | O No | Reason: ____________________<br>Medicine prescribed: ____________________ |
| Has your child has been admitted to hospital? | O Yes | O No | Reason: ____________________<br>Medicine prescribed: ____________________ |

Regarding the side effects, your action is/are (you may answer more than one):

O Discontinuation of the study drug (prednisolone)

O Continuation of the study drug

O Discontinuation of other concomitant drugs as follows:

1. ____________________ 3. ____________________
2. ____________________ 4. ____________________

The treatment you prescribed for the management of side effects

1. ____________________; Dose __________; Frequency ______ / day
2. ____________________; Dose __________; Frequency ______ / day
3. ____________________; Dose __________; Frequency ______ / day
4. ____________________; Dose __________; Frequency ______ / day

Does this child require specific or additional tests or examination?

O No

O Yes. Please specify with the results:

1. ________________________________________
________________________________________
2. ________________________________________
________________________________________
3. ________________________________________
________________________________________

| | |
|---|---|
| Does this child require specific or additional treatment or medication | O No<br>O Yes. Please specify the treatment:<br>1. ______________________; Dose ___________; Frequency ______ / day<br>2. ______________________; Dose ___________; Frequency ______ / day<br>3. ______________________; Dose ___________; Frequency ______ / day<br>4. ______________________; Dose ___________; Frequency ______ / day |
| Does this child require a hospitalisation? | O No<br>O Yes. Please explain your reasons to hospitalise this child and the treatment will be given<br>Reason: ____________________________________________<br>____________________________________________<br>The treatment:<br>1. ______________________; Dose ___________; Frequency ______ / day<br>2. ______________________; Dose ___________; Frequency ______ / day<br>3. ______________________; Dose ___________; Frequency ______ / day<br>4. ______________________; Dose ___________; Frequency ______ / day |

**15 Tympanometry examination (for Audiologist and interpreted by Physician)**

O Cannot be performed. Reason: ____________________________________________

Tympanogram types (will be completed by physician) [R] Type ______ / [L] Type ______

Ear canal vol (ECV) [R] ________ mL / [L] ________ mL

Static acoustic admittance [R] _______ mL / [L] _______ mL

Compliance (SC) [R] ________ mL / [L] ________ mL

Middle Ear Pressure or TPP [R] _______ daPa / [L] ______ daPa

Gradient or TW [R] _______ daPa / [L] ______ daPa

*Put the copy of tympanometry copies here*

Registration ID

## Follow-up Visit – 2 (Day – 7) : |___|___| – |___|___| – 20 |___|___|

### Complications (for Physician)

| | | |
|---|---|---|
| 1 | **Does your child experience discharge from the ear(s)?** | O Yes O No |
| 2 | **Does your child experience intense ear pain and pain behind the ear?** | O Yes O No |
| 3 | **Does your child experience swelling/bulging/ or redness/tenderness of the ear(s)?** | O Yes O No |
| 4 | **Does your child experience facial asymmetry (e.g. when the child smiles, cries)?** | O Yes O No |

### General and ENT examination (for Nurse and Physician)

| | | | |
|---|---|---|---|
| 5.1 **Weight** _____ kg | 5.2 **Height** _______ cm | 5.3 **Temp.** _________ °C | 5.4 **BP** _____ / _____ mmHg |

6 **Nose** O Normal O Oedema O Hyperaemic O Livid O Serous discharge O Mucoid discharge

7 **Tonsils** O Normal O Hyperaemic O Detritus O Tonsil(s) T1 O Tonsil(s) T2 O Tonsil(s) T3-4

8 **Pharynx** O Normal O Hyperaemic O Oedema O Granules O Post nasal drip (PND)

### 9 Otoscopic examination

O Normal O Cerumen O Erythema O Air fluid level O Complete effusion O Opacification

O Mild bulging O Moderate to severe bulging (bulging rounded) O Bulla O Perforation

### 10 Medicines prescribed by you (Physician) today visit (please circle your dose measurement)

| | |
|---|---|
| Antibiotic | ________________<br>Dose : _______mg / BW kg Frequency : _______ / day for _______ days |

Other medicine(s)

1. ________________________ Dose : _____mg perBw kg / Teaspoon / Tablespoon ; Frequency : ____/ day
2. ________________________ Dose : _____mg perBw kg / Teaspoon / Tablespoon ; Frequency : ____/ day
3. ________________________ Dose : _____mg perBw kg / Teaspoon / Tablespoon ; Frequency : ____/ day
4. ________________________ Dose : _____mg perBw kg / Teaspoon / Tablespoon ; Frequency : ____/ day
5. ________________________ Dose : _____mg perBw kg / Teaspoon / Tablespoon ; Frequency : ____/ day

### 11 Medicines NOT prescribed by you or from over-the-counter or others (e.g. other physician, drug store)

| | |
|---|---|
| Antibiotic | ________________<br>Dose : _______mg / BW kg Frequency : _______ / day for _______ days |

Other medicine(s)

6. ________________________ Dose : _____mg perBw kg / Teaspoon / Tablespoon ; Frequency : ____/ day
7. ________________________ Dose : _____mg perBw kg / Teaspoon / Tablespoon ; Frequency : ____/ day
8. ________________________ Dose : _____mg perBw kg / Teaspoon / Tablespoon ; Frequency : ____/ day
9. ________________________ Dose : _____mg perBw kg / Teaspoon / Tablespoon ; Frequency : ____/ day
10. ________________________ Dose : _____mg perBw kg / Teaspoon / Tablespoon ; Frequency : ____/ day

### Outcome: Symptoms (for Patients)

**12 Please place a vertical line across the available horizontal line that best describes your or your child's pain during the past 24 hours?**

No Pain |————————————————| Pain As Bad As It Could Possibly Be

*For each question, please tick (✓) your answer on the circles or write you answer on ______*

| | | | |
|---|---|---|---|
| **13 We are interest finding out how your child has been doing. For each question, please place a checkmark (V) in the circle corresponding to your child's symptoms. Please answer all questions.** | | | |
| 13.1 Over the past 12 h, has your child been tugging, rubbing, or holding the ear(s) more than usual? | O No | O A little | O A lot |
| 13.2 Over the past 12 h, has your child been crying more than usual? | O No | O A little | O A lot |
| 13.3 Over the past 12 h, has your child been more irritable or fussy than usual? | O No | O A little | O A lot |
| 13.4 Over the past 12 h, has your child been having more difficulty sleeping than usual? | O No | O A little | O A lot |
| 13.5 Over the past 12 h, has your child been less playful or active than usual? | O No | O A little | O A lot |
| 13.6 Over the past 12 h, has your child been eating less than usual? | O No | O A little | O A lot |
| 13.7 Over the past 12 h, has your child been having fever or feeling warm to touch? | O No | O A little | O A lot |

**14 Side effects**

**Does your child have these complaints after taking the medicine**

| | | | | | |
|---|---|---|---|---|---|
| 14.1 Increased appetite | O Yes | O No | 14.8 Drowsiness | O Yes | O No |
| 14.2 Increased urine amount | O Yes | O No | 14.9 Anxiety/distractibility/mood swing | O Yes | O No |
| 14.3 Weight gain | O Yes | O No | 14.10 Headache | O Yes | O No |
| 14.4 Gastritis/abdominal pain | O Yes | O No | 14.11 Skin rash or diaper rash | O Yes | O No |
| 14.5 Nausea | O Yes | O No | 14.12 Candidiasis | O Yes | O No |
| 14.6 Vomiting | O Yes | O No | 14.13 Dry mouth / throat irritation | O Yes | O No |
| 14.7 Diarrhea | O Yes | O No | 14.14 Sleep disturbance | O Yes | O No |

Others: ______________________________________________

| | | | |
|---|---|---|---|
| Did you bring your child to doctor (clinic or outpatient)? | O Yes | O No | Reason: ____________<br>Medicine prescribed: ____________ |
| Has your child has been admitted to hospital? | O Yes | O No | Reason: ____________<br>Medicine prescribed: ____________ |

Regarding the side effects, your action is/are (you may answer more than one):

O Discontinuation of the study drug (prednisolone)

O Continuation of the study drug

O Discontinuation of other concomitant drugs as follows:

1. ____________ 3. ____________
2. ____________ 4. ____________

Does this child require specific or additional tests or examination?

O No

O Yes. Please specify with the results:

1. ______________________________
2. ______________________________
3. ______________________________

Does this child require specific or additional treatment or medication

O No

O Yes. Please specify the treatment:

5. ____________; Dose ________; Frequency ______ / day

6. ______________________; Dose ___________; Frequency ______ / day
7. ______________________; Dose ___________; Frequency ______ / day
8. ______________________; Dose ___________; Frequency ______ / day

Does this child require a hospitalisation?

O No

O Yes. Please explain your reasons to hospitalise this child and the treatment will be given

Reason: ____________________________________________________________

____________________________________________________________

The treatment:

5. ______________________; Dose ___________; Frequency ______ / day
6. ______________________; Dose ___________; Frequency ______ / day
7. ______________________; Dose ___________; Frequency ______ / day
8. ______________________; Dose ___________; Frequency ______ / day

## 15 Tympanometry examination (for Audiologist and interpreted by Physician)

O Cannot be performed. Reason: ____________________________________________________________

Tympanogram types (will be completed by physician) [R] Type ______ / [L] Type ______

Ear canal vol (ECV) [R] ________ mL / [L] ________ mL

Static acoustic admittance [R] _______ mL / [L] _______ mL

Compliance (SC) [R] ________ mL / [L] ________ mL

Middle Ear Pressure or TPP [R] _______ daPa / [L] ______ daPa

Gradient or TW [R] _______ daPa / [L] ______ daPa

*Put the copy of tympanometry copies here*

## Follow-up Visit – 3 (Day – 30) : |___|___| – |___|___| – 20 |___|___|

### Outcome: Symptoms (for Patients)

| | |
|---|---|
| 1 Within the past one month, does your child experience a new episode of ear pain with fever or runny nose, cough, or sore throat? Please write your answer and circle the most appropriate time | O Yes O No<br>When? ________ days / weeks ago<br>How long? ________ days / weeks |

**2 Please place a vertical line across the available horizontal line that best describes your or your child's pain during the past 24 hours? (if applicable)**

No Pain |————————————————| Pain As Bad As It Could Possibly Be

**3. We are interest finding out how your child has been doing. For each question, please place a checkmark (V) in the circle corresponding to your child's symptoms. Please answer all questions (if applicable).**

| | | | |
|---|---|---|---|
| 3.1 Over the past 12 h, has your child been tugging, rubbing, or holding the ear(s) more than usual? | O No | O A little | O A lot |
| 3.2 Over the past 12 h, has your child been crying more than usual? | O No | O A little | O A lot |
| 3.3 Over the past 12 h, has your child been more irritable or fussy than usual? | O No | O A little | O A lot |
| 3.4 Over the past 12 h, has your child been having more difficulty sleeping than usual? | O No | O A little | O A lot |
| 3.5 Over the past 12 h, has your child been less playful or active than usual? | O No | O A little | O A lot |
| 3.6 Over the past 12 h, has your child been eating less than usual? | O No | O A little | O A lot |
| 3.7 Over the past 12 h, has your child been having fever or feeling warm to touch? | O No | O A little | O A lot |

### General and ENT examination (for Nurse and Physician)

| | | | |
|---|---|---|---|
| 4.1 **Weight** _____ kg | 5.2 **Height** _______ cm | 5.3 **Temp.** ________ °C | 5.4 **BP** _____ / _____ mmHg |

5 **Nose** O Normal O Oedema O Hyperaemic O Livid O Serous discharge O Mucoid discharge

6 **Tonsils** O Normal O Hyperaemic O Detritus O Tonsil(s) T1 O Tonsil(s) T2 O Tonsil(s) T3-4

7 **Pharynx** O Normal O Hyperaemic O Oedema O Granules O Post nasal drip (PND)

### 8 Otoscopic examination

O Normal O Cerumen O Erythema O Air fluid level O Complete effusion O Opacification

O Mild bulging O Moderate to severe bulging (bulging rounded) O Bulla O Perforation

### 9 Tympanometry examination (for Audiologist and interpreted by Physician)

O Cannot be performed. Reason: ________________________________________

Tympanogram types (will be completed by physician) [R] Type ______ / [L] Type ______

Ear canal vol (ECV) [R] ________ mL / [L] ________ mL

Static acoustic admittance [R] _______ mL / [L] _______ mL

Compliance (SC) [R] ________ mL / [L] ________ mL

Middle Ear Pressure or TPP [R] _______ daPa / [L] ______ daPa

Gradient or TW [R] _______ daPa / [L] ______ daPa

*For each question, please tick (✓) your answer on the circles or write you answer on ______*

*Put the copy of tympanometry copies here*

## Follow-up Visit – 4 (Day – 90) : |___|___| – |___|___| – 20 |__|__|

### Outcome: Symptoms (for Patients)

| | |
|---|---|
| 1 Within the past one month, does your child experience a new episode of ear pain with fever or runny nose, cough, or sore throat? Please write your answer and circle the most appropriate time | O Yes O No<br>When? ________ days / weeks ago<br>How long? ________ days / weeks |

**2 Please place a vertical line across the available horizontal line that best describes your or your child's pain during the past 24 hours? (if applicable)**

No Pain |————————————————| Pain As Bad As It Could Possibly Be

**3 We are interest finding out how your child has been doing. For each question, please place a checkmark (V) in the circle corresponding to your child's symptoms. Please answer all questions (if applicable).**

| | | | |
|---|---|---|---|
| 3.1 Over the past 12 h, has your child been tugging, rubbing, or holding the ear(s) more than usual? | O No | O A little | O A lot |
| 3.2 Over the past 12 h, has your child been crying more than usual? | O No | O A little | O A lot |
| 3.3 Over the past 12 h, has your child been more irritable or fussy than usual? | O No | O A little | O A lot |
| 3.4 Over the past 12 h, has your child been having more difficulty sleeping than usual? | O No | O A little | O A lot |
| 3.5 Over the past 12 h, has your child been less playful or active than usual? | O No | O A little | O A lot |
| 3.6 Over the past 12 h, has your child been eating less than usual? | O No | O A little | O A lot |
| 3.7 Over the past 12 h, has your child been having fever or feeling warm to touch? | O No | O A little | O A lot |

### General and ENT examination (for Nurse and Physician)

4.1 **Weight** _____ kg | 5.2 **Height** _______ cm | 5.3 **Temp.** ________ °C | 5.4 **BP** _____ / _____ mmHg

5 **Nose** O Normal O Oedema O Hyperaemic O Livid O Serous discharge O Mucoid discharge

6 **Tonsils** O Normal O Hyperaemic O Detritus O Tonsil(s) T1 O Tonsil(s) T2 O Tonsil(s) T3-4

7 **Pharynx** O Normal O Hyperaemic O Oedema O Granules O Post nasal drip (PND)

### 8 Otoscopic examination

O Normal O Cerumen O Erythema O Air fluid level O Complete effusion O Opacification

O Mild bulging O Moderate to severe bulging (bulging rounded) O Bulla O Perforation

### 9 Tympanometry examination (for Audiologist and interpreted by Physician)

O Cannot be performed. Reason: ______________________________________________

Tympanogram types (will be completed by physician) [R] Type ______ / [L] Type ______

Ear canal vol (ECV) [R] ________ mL / [L] ________ mL

Static acoustic admittance [R] _______ mL / [L] _______ mL

Compliance (SC) [R] ________ mL / [L] ________ mL

Middle Ear Pressure or TPP [R] _______ daPa / [L] ______ daPa

Gradient or TW [R] _______ daPa / [L] ______ daPa

*For each question, please tick (✓) your answer on the circles or write you answer on* ______

*Put the copy of tympanometry copies here*

***** End *****

**Registration ID** ____________

## Additional Visit : |___|___| – |___|___| – 20 |___|___|

### Complications (for Physician)

| | | |
|---|---|---|
| 1 | **Does your child experience discharge from the ear(s)?** | O Yes O No |
| 2 | **Does your child experience intense ear pain and pain behind the ear?** | O Yes O No |
| 3 | **Does your child experience swelling/bulging/ or redness/tenderness of the ear(s)?** | O Yes O No |
| 4 | **Does your child experience facial asymmetry (e.g. when the child smiles, cries)?** | O Yes O No |

### General and ENT examination (for Nurse and Physician)

5.1 **Weight** _____ kg | 5.2 **Height** _______ cm | 5.3 **Temp.** ________ °C | 5.4 **BP** _____ / _____ mmHg

6 **Nose** O Normal O Oedema O Hyperaemic O Livid O Serous discharge O Mucoid discharge

7 **Tonsils** O Normal O Hyperaemic O Detritus O Tonsil(s) T1 O Tonsil(s) T2 O Tonsil(s) T3-4

8 **Pharynx** O Normal O Hyperaemic O Oedema O Granules O Post nasal drip (PND)

### 9 Otoscopic examination

O Normal O Cerumen O Erythema O Air fluid level O Complete effusion O Opacification

O Mild bulging O Moderate to severe bulging (bulging rounded) O Bulla O Perforation

### 10 Medicines prescribed by you (Physician) on today visit (please circle your dose measurement)

Antibiotic

Dose : _______mg / BW kg Frequency : _______ / day for _______ days

Other medicine(s)

6. ________________________ Dose : _____mg perBw kg / Teaspoon / Tablespoon ; Frequency : ____ / day
7. ________________________ Dose : _____mg perBw kg / Teaspoon / Tablespoon ; Frequency : ____ / day
8. ________________________ Dose : _____mg perBw kg / Teaspoon / Tablespoon ; Frequency : ____ / day
9. ________________________ Dose : _____mg perBw kg / Teaspoon / Tablespoon ; Frequency : ____ / day
10. ________________________ Dose : _____mg perBw kg / Teaspoon / Tablespoon ; Frequency : ____ / day

### 11 Medicines NOT prescribed by you or from over-the-counter or others (e.g. other physician, drug store)

Antibiotic

Dose : _______mg / BW kg Frequency : _______ / day for _______ days

Other medicine(s)

11. ________________________ Dose : _____mg perBw kg / Teaspoon / Tablespoon ; Frequency : ____ / day
12. ________________________ Dose : _____mg perBw kg / Teaspoon / Tablespoon ; Frequency : ____ / day
13. ________________________ Dose : _____mg perBw kg / Teaspoon / Tablespoon ; Frequency : ____ / day
14. ________________________ Dose : _____mg perBw kg / Teaspoon / Tablespoon ; Frequency : ____ / day
15. ________________________ Dose : _____mg perBw kg / Teaspoon / Tablespoon ; Frequency : ____ / day

### Outcome: Symptoms (for Patients)

13 **Please place a vertical line across the available horizontal line that best describes your or your child's pain during the past 24 hours?**

|———————————————————————|

No Pain ... Pain As Bad As It Could Possibly Be

*For each question, please tick (✓) your answer on the circles or write you answer on ______*

| **13. We are interest finding out how your child has been doing. For each question, please place a checkmark (V) in the circle corresponding to your child's symptoms. Please answer all questions.** | | | |
|---|---|---|---|
| 13.1 Over the past 12 h, has your child been tugging, rubbing, or holding the ear(s) more than usual? | O No | O A little | O A lot |
| 13.2 Over the past 12 h, has your child been crying more than usual? | O No | O A little | O A lot |
| 13.3 Over the past 12 h, has your child been more irritable or fussy than usual? | O No | O A little | O A lot |
| 13.4 Over the past 12 h, has your child been having more difficulty sleeping than usual? | O No | O A little | O A lot |
| 13.5 Over the past 12 h, has your child been less playful or active than usual? | O No | O A little | O A lot |
| 13.6 Over the past 12 h, has your child been eating less than usual? | O No | O A little | O A lot |
| 13.7 Over the past 12 h, has your child been having fever or feeling warm to touch? | O No | O A little | O A lot |

**14 Side effects**

**Does your child have these complaints after taking the medicine**

| | | | | | |
|---|---|---|---|---|---|
| 14.1 Increased appetite | O Yes | O No | 14.8 Drowsiness | O Yes | O No |
| 14.2 Increased urine amount | O Yes | O No | 14.9 Anxiety/distractibility/mood swing | O Yes | O No |
| 14.3 Weight gain | O Yes | O No | 14.10 Headache | O Yes | O No |
| 14.4 Gastritis/abdominal pain | O Yes | O No | 14.11 Skin rash or diaper rash | O Yes | O No |
| 14.5 Nausea | O Yes | O No | 14.12 Candidiasis | O Yes | O No |
| 14.6 Vomiting | O Yes | O No | 14.13 Dry mouth / throat irritation | O Yes | O No |
| 14.7 Diarrhea | O Yes | O No | 14.14 Sleep disturbance | O Yes | O No |

Others: ______________________________

| | | |
|---|---|---|
| Did you bring your child to doctor (clinic or outpatient)? | O Yes O No | Reason: ____________<br>Medicine prescribed: ____________ |
| Has your child has been admitted to hospital? | O Yes O No | Reason: ____________<br>Medicine prescribed: ____________ |
| Regarding the side effects, your action is/are (you may answer more than one): | O Discontinuation of the study drug (prednisolone)<br>O Continuation of the study drug<br>O Discontinuation of other concomitant drugs as follows:<br>1. ____________ 3. ____________<br>2. ____________ 4. ____________ | |
| The treatment you prescribed for the management of side effects | 5. ____________; Dose ________; Frequency ______ / day<br>6. ____________; Dose ________; Frequency ______ / day<br>7. ____________; Dose ________; Frequency ______ / day<br>8. ____________; Dose ________; Frequency ______ / day | |
| Does this child require specific or additional tests or examination? | O No<br>O Yes. Please specify with the results:<br>4. ____________<br>____________<br>5. ____________<br>____________<br>6. ____________<br>____________ | |

| | |
|---|---|
| Does this child require specific or additional treatment or medication | O No<br>O Yes. Please specify the treatment:<br>9. ____________________; Dose __________; Frequency _____ / day<br>10. ____________________; Dose __________; Frequency _____ / day<br>11. ____________________; Dose __________; Frequency _____ / day<br>12. ____________________; Dose __________; Frequency _____ / day |
| Does this child require a hospitalisation? | O No<br>O Yes. Please explain your reasons to hospitalise this child and the treatment will be given<br>Reason: ________________________________________<br>________________________________________<br>The treatment:<br>9. ____________________; Dose __________; Frequency _____ / day<br>10. ____________________; Dose __________; Frequency _____ / day<br>11. ____________________; Dose __________; Frequency _____ / day<br>12. ____________________; Dose __________; Frequency _____ / day |

## 15 Tympanometry examination (for Audiologist and interpreted by Physician)

O Cannot be performed. Reason: ________________________________________

Tympanogram types (will be completed by physician) [R] Type _____ / [L] Type _____

Ear canal vol (ECV) [R] _______ mL / [L] _______ mL

Static acoustic admittance [R] ______ mL / [L] ______ mL

Compliance (SC) [R] _______ mL / [L] _______ mL

Middle Ear Pressure or TPP [R] ______ daPa / [L] _____ daPa

Gradient or TW [R] ______ daPa / [L] _____ daPa

*Put the copy of tympanometry copies here*

## Additional Visit : |___|___| – |___|___| – 20 |___|___|

### Complications (for Physician)

| | | |
|---|---|---|
| 1 | **Does your child experience discharge from the ear(s)?** | O Yes O No |
| 2 | **Does your child experience intense ear pain and pain behind the ear?** | O Yes O No |
| 3 | **Does your child experience swelling/bulging/ or redness/tenderness of the ear(s)?** | O Yes O No |
| 4 | **Does your child experience facial asymmetry (e.g. when the child smiles, cries)?** | O Yes O No |

### General and ENT examination (for Nurse and Physician)

| | | | |
|---|---|---|---|
| 5.1 **Weight** _____ kg | 5.2 **Height** _______ cm | 5.3 **Temp.** _________ °C | 5.4 **BP** _____ / _____ mmHg |

6 **Nose** O Normal O Oedema O Hyperaemic O Livid O Serous discharge O Mucoid discharge

7 **Tonsils** O Normal O Hyperaemic O Detritus O Tonsil(s) T1 O Tonsil(s) T2 O Tonsil(s) T3-4

8 **Pharynx** O Normal O Hyperaemic O Oedema O Granules O Post nasal drip (PND)

### 9 Otoscopic examination

O Normal O Cerumen O Erythema O Air fluid level O Complete effusion O Opacification

O Mild bulging O Moderate to severe bulging (bulging rounded) O Bulla O Perforation

### 10 Medicines prescribed by you (Physician) on today visit (please circle your dose measurement)

| | |
|---|---|
| Antibiotic | ______________________________ <br> Dose : _______mg / BW kg Frequency : _______ / day for _______ days |

Other medicine(s)

1. ________________________ Dose : _____mg perBw kg / Teaspoon / Tablespoon ; Frequency : ____ / day
2. ________________________ Dose : _____mg perBw kg / Teaspoon / Tablespoon ; Frequency : ____ / day
3. ________________________ Dose : _____mg perBw kg / Teaspoon / Tablespoon ; Frequency : ____ / day
4. ________________________ Dose : _____mg perBw kg / Teaspoon / Tablespoon ; Frequency : ____ / day
5. ________________________ Dose : _____mg perBw kg / Teaspoon / Tablespoon ; Frequency : ____ / day

### 11 Medicines NOT prescribed by you or from over-the-counter or others (e.g. other physician, drug store)

| | |
|---|---|
| Antibiotic | ______________________________ <br> Dose : _______mg / BW kg Frequency : _______ / day for _______ days |

Other medicine(s)

1. ________________________ Dose : _____mg perBw kg / Teaspoon / Tablespoon ; Frequency : ____ / day
2. ________________________ Dose : _____mg perBw kg / Teaspoon / Tablespoon ; Frequency : ____ / day
3. ________________________ Dose : _____mg perBw kg / Teaspoon / Tablespoon ; Frequency : ____ / day
4. ________________________ Dose : _____mg perBw kg / Teaspoon / Tablespoon ; Frequency : ____ / day
5. ________________________ Dose : _____mg perBw kg / Teaspoon / Tablespoon ; Frequency : ____ / day

### Outcome: Symptoms (for Patients)

12 **Please place a vertical line across the available horizontal line that best describes your or your child's pain during the past 24 hours?**

|———————————————————————————————|

No Pain ... Pain As Bad As It Could Possibly Be

*For each question, please tick (✓) your answer on the circles or write you answer on* ______

**13 We are interest finding out how your child has been doing. For each question, please place a checkmark (V) in the circle corresponding to your child's symptoms. Please answer all questions.**

| | | | |
|---|---|---|---|
| 13.1 Over the past 12 h, has your child been tugging, rubbing, or holding the ear(s) more than usual? | O No | O A little | O A lot |
| 13.2 Over the past 12 h, has your child been crying more than usual? | O No | O A little | O A lot |
| 13.3 Over the past 12 h, has your child been more irritable or fussy than usual? | O No | O A little | O A lot |
| 13.4 Over the past 12 h, has your child been having more difficulty sleeping than usual? | O No | O A little | O A lot |
| 13.5 Over the past 12 h, has your child been less playful or active than usual? | O No | O A little | O A lot |
| 13.6 Over the past 12 h, has your child been eating less than usual? | O No | O A little | O A lot |
| 13.7 Over the past 12 h, has your child been having fever or feeling warm to touch? | O No | O A little | O A lot |

**14 Side effects**

| | | | | | |
|---|---|---|---|---|---|
| 14.1 Increased appetite | O Yes | O No | 14.8 Drowsiness | O Yes | O No |
| 14.2 Increased urine amount | O Yes | O No | 14.9 Anxiety/distractibility/mood swing | O Yes | O No |
| 14.3 Weight gain | O Yes | O No | 14.10 Headache | O Yes | O No |
| 14.4 Gastritis/abdominal pain | O Yes | O No | 14.11 Skin rash or diaper rash | O Yes | O No |
| 14.5 Nausea | O Yes | O No | 14.12 Candidiasis | O Yes | O No |
| 14.6 Vomiting | O Yes | O No | 14.13 Dry mouth / throat irritation | O Yes | O No |
| 14.7 Diarrhea | O Yes | O No | 14.14 Sleep disturbance | O Yes | O No |

Others: ____________________________________________

| | | | |
|---|---|---|---|
| Did you bring your child to doctor (clinic or outpatient)? | O Yes | O No | Reason: ______________<br>Medicine prescribed: ______________ |
| Has your child has been admitted to hospital? | O Yes | O No | Reason: ______________<br>Medicine prescribed: ______________ |

Regarding the side effects, your action is/are (you may answer more than one):

O Discontinuation of the study drug (prednisolone)

O Continuation of the study drug

O Discontinuation of other concomitant drugs as follows:

1. ____________________ 3. ____________________
2. ____________________ 4. ____________________

The treatment you prescribed for the management of side effects

9. ____________________; Dose __________; Frequency ______ / day
10. ____________________; Dose __________; Frequency ______ / day
11. ____________________; Dose __________; Frequency ______ / day
12. ____________________; Dose __________; Frequency ______ / day

Does this child require specific or additional tests or examination?

O No

O Yes. Please specify with the results:

7. ________________________________________
   ________________________________________
8. ________________________________________
   ________________________________________
9. ________________________________________
   ________________________________________

Does this child require specific O No

| | |
|---|---|
| or additional treatment or medication | O Yes. Please specify the treatment:<br>13. ____________________; Dose __________; Frequency ______ / day<br>14. ____________________; Dose __________; Frequency ______ / day<br>15. ____________________; Dose __________; Frequency ______ / day<br>16. ____________________; Dose __________; Frequency ______ / day |
| Does this child require a hospitalisation? | O No<br>O Yes. Please explain your reasons to hospitalise this child and the treatment will be given<br>Reason: ______________________________________________<br>______________________________________________<br>The treatment:<br>13. ____________________; Dose __________; Frequency ______ / day<br>14. ____________________; Dose __________; Frequency ______ / day<br>15. ____________________; Dose __________; Frequency ______ / day<br>16. ____________________; Dose __________; Frequency ______ / day |

## 15 Tympanometry examination (for Audiologist and interpreted by Physician)

O Cannot be performed. Reason: ______________________________________________

Tympanogram types (will be completed by physician) [R] Type ______ / [L] Type ______

Ear canal vol (ECV) [R] ________ mL / [L] ________ mL

Static acoustic admittance [R] _______ mL / [L] _______ mL

Compliance (SC) [R] ________ mL / [L] ________ mL

Middle Ear Pressure or TPP [R] _______ daPa / [L] ______ daPa

Gradient or TW [R] _______ daPa / [L] ______ daPa

*Put the copy of tympanometry copies here*

Date ______________________

# CRF07. Prescription for OPAL study medication

**Prednisolone doses:**

- Aged 6 months to < 2 years old = 10 mg per day
- Aged 2 years to < 6 years old = 20 mg per day
- Aged 6 years to 12 years old = 30 mg per day

**Registration ID** : [ ]

**Name** : ______________________

**Age** : ______________ months / year(s) *[write and circle your answer]*

**Study medication dose** : ________ mg per day = ________ tablets per day

R/ OPAL study medication tablet ……..

Sach lact add

m.f. pulveres dtd No. V

ʃ 1 dd 1 pc (before 9 am)

______________________________________________

(sign here)

REGISTRATION ID | | | | |

**Nurse ID : |___|___|___|** **Site ID : |___|___|___|** **Date : |___|___| – |___|___| – 201 |___|**

# CR08 – RANDOMISATION FORM

| | | |
|---|---|---|
| **Eligibility criteria (cross-check with 'FORM01. study registration log book', and 'CRF03. Eligibility form' in the 'Case Report Form Binder' of this subject).** | | |
| All YES for all inclusion criteria | **O** Yes | **O** No |
| All NO for all exclusion criteria | **O** Yes | **O** No |
| **Consent to the study questions (cross-check with 'CRF01. Informed consent' in the 'Case Report Form Binder' of this subject).** | | |
| Has consent given? | **O** Yes | **O** No |

## RANDOMISATION

| | | | | | | | | |
|---|---|---|---|---|---|---|---|---|
| **Father's mobile phone number** | | | | | | | | |
| **Mother's mobile phone number** | | | | | | | | |
| **Severity of AOM** | **O Mild AOM** | | | | | **O Severe AOM** | | |
| **Subject's date of birth** | Date | | Month | | Year | **AGE** | | Month/year |

## RANDOMISATION RESULT

| | | | |
|---|---|---|---|
| **Randomisation ID** | | | |
| **This subject is allocated to** | **O Prednisolone group** | **O Control group (no prednisolone)** | |
| **Prednisolone dosage (if the subject is allocated to prednisolone group)** | **O 10 mg/day** | **O 20 mg/day** | **O 30 mg/day** |

| | | | | | |
|---|---|---|---|---|---|
| Nurse's signature | | Nurse's name | | Date | |

For each question, please tick (✓) the circle corresponding to your answers or write you answer

# Follow-up Visit Card

OPAL Study
Oral Prednisolone for Acute otitis media in children

Name : ______________________________

Address : ______________________________

______________________________

Dad/Mom's phone no : ______________________________

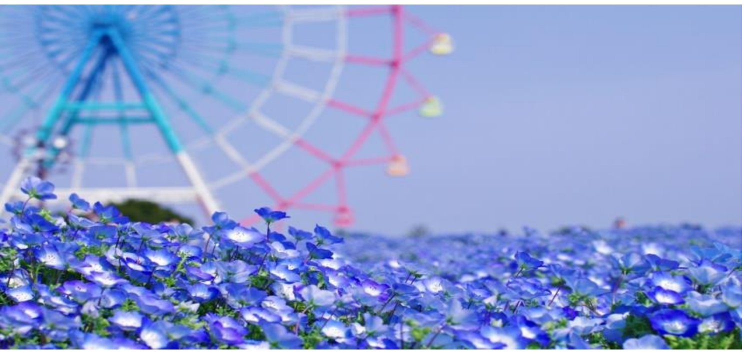

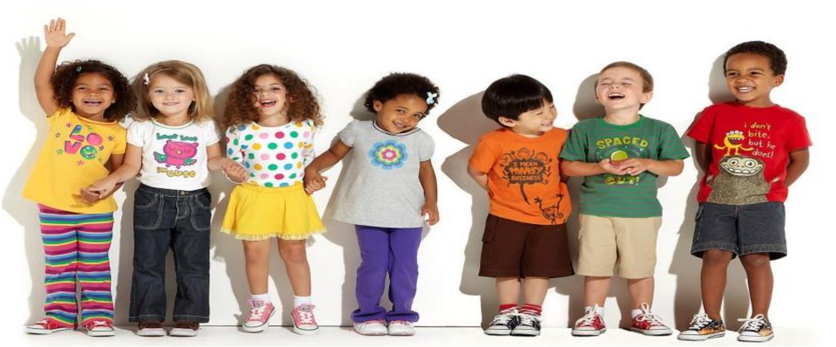


**Clinical Epidemiology and Evidence-Based Medicine (CEEBM) Unit**

**Dr Cipto Mangunkusumo Hospital – Faculty of Medicine Universitas Indonesia**

**Centre for Research in Evidence-Based Practice**

**Faculty of Health Sciences and Medicine, Bond University, Australia**

# Follow-up Visit Schedule

| | Initial visit date | Scheduled visit dates | Actual visit dates | Notes |
|---|---|---|---|---|
| Initial visit (Day–0) | | | | |
| Visit – 1 (Day – 3) | | | | |
| Visit – 2 (Day – 7) | | | | |
| Visit – 3 (Month – 1) | | | | |
| Visit – 4 (Month – 4) | | | | |

Please always bring this card to every your follow-up visit to the Hospital

# Phone numbers of Hospitals and Call-centre OPAL Study

## Dr. Cipto Mangunkusumo Hospital
**Jl. Diponegoro No.71, Central Jakarta**
**Operator : 1500135**

## Persahabatan Hospital
**Jl. Persahabatan Raya No.1, East Jakarta**
**Operator : 021 489 1708 Ext. 285**
**ENT Clinic : Ext. 230**
**Paediatric Clinic : Ext. 283**
**Emergency Instalation : Ext. 499**

## Gatot Soebroto Army Hospital
**Jl. Dr Abdul Rahman Saleh No.24, Senen, Central Jakarta**
**Operator : 021 344 1008, 021 384 0702**
**ENT Clinic : Ext. 2057**
**Paediatric Clinic : Ext. 2535**
**Emergency Instalation : Ext. 2121**

## Jakarta Islamic Hospital Cempaka Putih
**Jl. Cempaka Putih Tengah I No. 1, Central Jakarta**
**Operator : 021 425 0451, 021 428 01567 Ext. 0**
**Outpatient Registration : Ext. 2**
**Emergency Instalation : Ext. 1**

## Proklamasi ENT Hospital
**Jl. Proklamasi No.43, Central Jakarta**
**Operator : 021 390 0002, 021 392 4891 Ext. 0, 101, 227, 229**
**ENT Clinic : Ext. 100, 236 244**
**Emergency Instalation : Ext. 235**

## Antam Medika Hospital Pulogadung
**Jl. Raya Pemuda No. 1A, Pulogadung, East Jakarta**
**Operator : 021 806 14 888**
**ENT Clinic : Ext. 1027**
**Paediatric Clinic : Ext. 1019**
**Emergency Instalation : Ext. 1045**

## 24-Call Centre OPAL Study
**Dr. Respati W. Ranakusuma, Sp.THT-KL : 08111 012 185**

REGISTRATION ID

# CRF10. SERIOUS ADVERSE EVENTS REPORTING FORM

## SUBJECT INFORMATION

| | |
|---|---|
| Weight (kg) | \|____\|____\| , \|____\|____\| kg |
| List any relevant tests, laboratory data, history, including pre-existing medical conditions | |
| Any concomitant medication | |

## ADVERSE EVENT

| | | | |
|---|---|---|---|
| Report type | ☐ Initial report | ☐ Follow-up | ☐ Final |
| Reason for reporting | ☐ Requires or prolongs hospitalization | ☐ Congenital anomaly | |
| | ☐ Permanently disabling or incapacitating | ☐ Life threatening | |
| | ☐ Overdose | ☐ Death | |
| | ☐ Other (please specify) __________ | Date of death __________ | |
| | | Cause of death __________ | |

## SUSPECTED DRUG

| | | | |
|---|---|---|---|
| Name of suspected drug | __________ | Generic name | __________ |
| Dose details | __________ | Name of manufacturer | __________ |
| Date of occurrence | \|____\|____\| - \|____\|____\| - \|____\|____\|____\|____\| *(date – month – year)* | | |
| Duration of event | \|____\|____\| month(s) \|____\|____\| day(s) | | |
| Starting date of medication | \|____\|____\| - \|____\|____\| - \|____\|____\|____\|____\| *(date – month – year)* | | |
| Route of administration | __________ | Indication | __________ |
| Discontinuation of drug because of event | ☐ No | ☐ Yes | Dated (date / month / year) : __________ |
| If stopped/lowered dose, did the event resolve after this? | ☐ Yes | ☐ No | ☐ N/A |
| If reintroduced did the event reappear? | ☐ Yes | ☐ No | ☐ N/A |
| Outcomes | ☐ Recovered | ☐ Recovered with sequelae | ☐ Continuing |
| | ☐ Change in SAE | ☐ Patient died | ☐ Unknown |
| Severity | ☐ Mild | ☐ Moderate | ☐ Severe |
| Action taken with study drug | ☐ None | ☐ Dose reduced | ☐ Discontinued |
| | ☐ Dose temporarily reduced | ☐ Discontinued temporarily | |
| Other action* | ☐ None | ☐ Treated with medication | ☐ Other |

| | | |
|---|---|---|
| Withdrawn from the trial due to SAE | ☐ No | ☐ Yes |
| **REPORTER INFORMATION** | | |
| Signature of reporter | ______________________________ | |
| Date of signing | \|____\|____\| - \|____\|____\| - \|____\|____\|____\|____\| *(date – month – year)* | |
| Full name | ______________________________ | |

**Registration ID** 

# CRF11 – FEEDBACK FORM (for Physician only)

<table>
<tr><th colspan="2">Questions</th><th colspan="5">Please place a checkmark (√) inthe box corresponding to your answer</th></tr>
<tr><td rowspan="2">Information Sheet and Consent Form</td><td>How do you rate the process of providing patient information and informed consent to your patient?</td><td>☐<br>Very easy</td><td>☐<br>Easy</td><td>☐<br>Neutral</td><td>☐<br>Difficult</td><td>☐<br>Very difficult</td></tr>
<tr><td>If your answer ‘difficult’ or ‘very difficult’, please place a checkmark (V) in the box corresponding to or write you reason(s). You may choose more than one</td><td colspan="5">☐ It was too difficult to explain this to my patient/parent<br>☐ Time consuming<br>☐ There was too much information to explain<br>☐ I was not sure that my patient understood<br>☐ Others : ______________________________<br>______________________________<br>______________________________</td></tr>
<tr><td rowspan="2">Otosocopic Examination</td><td>How do rate the process of conducting an otoscopic examination to your patient?</td><td>☐<br>Very easy</td><td>☐<br>Easy</td><td>☐<br>Neutral</td><td>☐<br>Difficult</td><td>☐<br>Very difficult</td></tr>
<tr><td>If your answer ‘difficult’ or ‘very difficult’, please place a checkmark (V) in the box corresponding to or write you reason(s). You may choose more than one</td><td colspan="5">☐ Patient was not cooperative<br>☐ The ear canal was too narrow<br>☐ Insufficient tool (e.g. the otoscope cylidner was too large)<br>☐ Ear wax and it was too difficult to extract<br>☐ The symptoms are definitely celar showing AOM<br>☐ Others : ______________________________<br>______________________________<br>______________________________</td></tr>
<tr><td rowspan="2">Visual Analogue Scale (VAS)</td><td>How do you rate the process of providing related information and assisting your patient/parent to complete the visual analogue scale (VAS)?</td><td>☐<br>Very easy</td><td>☐<br>Easy</td><td>☐<br>Neutral</td><td>☐<br>Difficult</td><td>☐<br>Very difficult</td></tr>
<tr><td>If your answer ‘difficult’ or ‘very difficult’, please place a checkmark (V) in the box corresponding to or write you reason(s). You may choose more than one</td><td colspan="5">☐ It was too difficult to explain this to my patient/parent<br>☐ Time consuming<br>☐ I was not sure that my patient/parent understood<br>☐ My patient/parent seem not confidence with the answer<br>☐ Others : ______________________________<br>______________________________<br>______________________________</td></tr>
<tr><td>Acute Otitis Media –</td><td>How do you rate the process of providing related information and assisting your patient/parent to complete the acute otitis</td><td>☐<br>Very easy</td><td>☐<br>Easy</td><td>☐<br>Neutral</td><td>☐<br>Difficult</td><td>☐<br>Very difficult</td></tr>
</table>

| | | | | | | |
|---|---|---|---|---|---|---|
| | **media – severity of symptom scale (AOM–SOS)**? | | | | | |
| | If your answer 'difficult' or 'very difficult', please place a checkmark (V) in the box corresponding to or write you reason(s). You may choose more than one | ☐ It was too difficult to explain this to my patient/parent<br>☐ Time consuming<br>☐ I was not sure that my patient/parent understood<br>☐ There were several questions that difficult to explain or not suitable for my patient/parent: question no. ___ ; ___ ; ___ ;<br>☐ Others : ______________________<br>______________________<br>______________________ | | | | |
| **Symptom Diary** | How do you rate the process of providing related information and assisting your patient/parent to complete **the Patient/parent Diary**? | ☐ Very easy | ☐ Easy | ☐ Neutral | ☐ Difficult | ☐ Very difficult |
| | If your answer 'difficult' or 'very difficult', please place a checkmark (V) in the box corresponding to or write you reason(s). You may choose more than one | ☐ It was too difficult to explain this to my patient/parent<br>☐ Time consuming<br>☐ I was not sure that my patient/parent understood<br>☐ The sequence of the questions was too confusing<br>☐ Questions in the symptom diary are too many<br>☐ Others : ______________________<br>______________________<br>______________________ | | | | |
| **Case Report Forms** | How do you rate the process in completing the **case report forms (CRFs)**? | ☐ Very easy | ☐ Easy | ☐ Neutral | ☐ Difficult | ☐ Very difficult |
| | If your answer 'difficult' or 'very difficult', please place a checkmark (V) in the box corresponding to or write you reason(s). You may choose more than one | ☐ Time consuming<br>☐ Too much unnecessary information was required<br>☐ The sequence of the questions was too confusing<br>☐ Several questions in the CRF were difficult to understand<br>☐ Others : ______________________<br>______________________<br>______________________ | | | | |
| **Screening and Stratification Process** | How do you rate **the recruitment process**, particularly in classifying the children based on their eligibility and **stratification process** to mild or severe AOM groups? | ☐ Very easy | ☐ Easy | ☐ Neutral | ☐ Difficult | ☐ Very difficult |
| | If your answer 'difficult' or 'very difficult', please place a checkmark (V) in the box corresponding to or write | ☐ The CRF03. Eligibility form is too complicated<br>☐ The form was not helping me to screen and stratify my patient/ parent<br>☐ Despite I was guided by the form, I was still found the process was still confusing, particularly in terms of deciding | | | | |

| | | |
|---|---|---|
| | you reason(s). You may choose more than one | which group my patient/parent should go to (i.e. mild vs severe acute otitis media)<br>☐ Others : ____________________<br>____________________<br>____________________ |

**Registration ID** [ ]

# FEEDBACK FORM (for Nurses who conducts randomisation only)

<table>
<tr><th colspan="2">Questions</th><th colspan="5">Please place a checkmark (√) inthe box corresponding to your answer</th></tr>
<tr><td rowspan="2">Randomisation Process</td><td>How do you rate the randomisation process, in terms of obtaining the study ID and the allocation of the intervention (prednisolone group or control group)</td><td>☐ Very easy</td><td>☐ Easy</td><td>☐ Neutral</td><td>☐ Difficult</td><td>☐ Very difficult</td></tr>
<tr><td>If your answer 'difficult' or 'very difficult', please place a checkmark (V) in the box corresponding to or write you reason(s). You may choose more than one.</td><td colspan="5">☐ The CRF08. Randomisation form is too complicated<br>☐ The randomisation process was too confusing<br>☐ It was difficult to access the randomisation centre (randomisation website or by phone) to obtain the study ID and the allocation of the intervention<br>☐ It was difficult to explain to the patients that they were allocated to groups which reveice prednisolone or not<br>☐ Others ____________________<br>____________________<br>____________________</td></tr>
<tr><td rowspan="2">Dispensing the Study Medication Prescription</td><td>How do rate the process of dispensing the study medication prescription and keep the intervention allocation concealed from their Physician and Audiologists?</td><td>☐ Very easy</td><td>☐ Easy</td><td>☐ Neutral</td><td>☐ Difficult</td><td>☐ Very difficult</td></tr>
<tr><td>If your answer 'difficult' or 'very difficult', please place a checkmark (V) in the box corresponding to or write you reason(s). You may choose more than one</td><td colspan="5">☐ This process was too time consuming<br>☐ I encountered difficulties when I was providing relevant information on the intervention they received<br>☐ It was difficult to ask my patients/parents to keep the information of intervention allocation confidential<br>☐ Others: ____________________<br>____________________<br>____________________</td></tr>
<tr><td rowspan="2">The compilation and the Storage of Case report Forms</td><td>How do rate the process of the compilation and the storage of study documents and binders?</td><td>☐ Very easy</td><td>☐ Easy</td><td>☐ Neutral</td><td>☐ Difficult</td><td>☐ Very difficult</td></tr>
<tr><td>If your answer 'difficult' or 'very difficult', please place a checkmark (V) in the box corresponding to or write you reason(s). You may choose more than one</td><td colspan="5">☐ This process was too confusing<br>☐ This process was too time consuming<br>☐ It was difficult to find case report forms in the binder<br>☐ The checklist of case report forms was not helping<br>☐ Others: ____________________<br>____________________<br>____________________</td></tr>
</table>

**Registration ID** ☐

## FEEDBACK FORM (for Audiologist/Trained Staff only)

<table>
<tr><th colspan="2">Questions</th><th colspan="5">Please place a checkmark (√) inthe box corresponding to your answer</th></tr>
<tr><td rowspan="2">Tympanomtry Examination and the Competion of Tympanometry Section in the Case report Form</td><td>How do you rate the process of tympanometry examination and completing the tympanometry section in CRF?</td><td>☐ Very easy</td><td>☐ Easy</td><td>☐ Neutral</td><td>☐ Difficult</td><td>☐ Very difficult</td></tr>
<tr><td>If your answer ‘difficult’ or ‘very difficult’, please place a checkmark (V) in the box corresponding to or write you reason(s). You may choose more than one.</td><td colspan="5">☐ The patients’ parents seem did not understand teh reason this examination being performed<br>☐ It was difficult to conduct this examination to my patients<br>☐ The ‘Tympanometry section’ in CRF05. Outcome form is confusing. The provided examination components are unfamiliar or different<br>☐ It was difficult to find the ‘Tympanometry section’ in the CRF05. Outcome form<br>☐ It was difficult to print out the copy of tympanometry result<br>☐ There were few components of this examination not provided in the form<br>☐ Others: ____________________<br>____________________<br>____________________</td></tr>
</table>

Registration ID

## FEEDBACK FORM (for Pharmacists only)

<table>
<tr><th colspan="2">Questions</th><th colspan="5">Please place a checkmark (√) inthe box corresponding to your answer</th></tr>
<tr><td rowspan="2">Preparation and Dispensing teh Study Medication</td><td>How do rate the preparation and dispensing process of the study medication?</td><td>☐ Very easy</td><td>☐ Easy</td><td>☐ Neutral</td><td>☐ Difficult</td><td>☐ Very difficult</td></tr>
<tr><td>If your answer ‘difficult’ or ‘very difficult’, please place a checkmark (V) in the box corresponding to or write you reason(s). You may choose more than one.</td><td colspan="5">☐ The instruction in CRF07. Prescription was confusing<br>☐ The preparation of the study medication was too time-consuming<br>☐ I encountered difficulties when providing the information about the study medication to my patients<br>☐ Others: ____________________<br>____________________<br>____________________</td></tr>
</table>

Registration ID

# FEEDBACK FORM (for Parents only)

| Questions | Please place a checkmark (√) inthe box corresponding to your answer |
|---|---|
| How do you rate the process in **completing the pain scale** below? | |

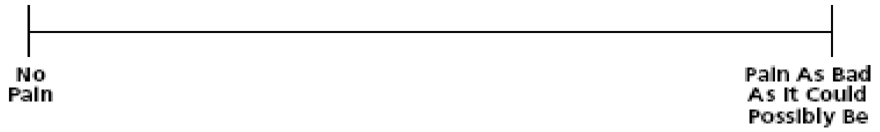


| | ☐ Very easy | ☐ Easy | ☐ Neutral | ☐ Difficult | ☐ Very difficult |
|---|---|---|---|---|---|
| If your answer 'difficult' or 'very difficult', please place a checkmark (V) in the box corresponding to or write you reason(s). You may choose more than one. | ☐ I did not understand how to complte this scale<br>☐ I need more information from my doctor<br>☐ The provided instruction in the form was unclear<br>☐ My doctor could not provide additional information that I need<br>☐ Others : ______________________ | | | | |

How do you rate the process in **completing the AOM-relevant symptom questionnaire** below?

| 12 | We are interest finding out how your child has been doing. For each question, please place a checkmark (V) in the circle corresponding to your child's symptoms. Please answer all questions. | | | |
|---|---|---|---|---|
| 12.1 | Over the past 12 h, has your child been tugging, rubbing, or holding the ear(s) more than usual? | O No | O A little | O A lot |
| 12.2 | Over the past 12 h, has your child been crying more than usual? | O No | O A little | O A lot |
| 12.3 | Over the past 12 h, has your child been more irritable or fussy than usual? | O No | O A little | O A lot |
| 12.4 | Over the past 12 h, has your child been having more difficulty sleeping than usual? | O No | O A little | O A lot |
| 12.5 | Over the past 12 h, has your child been less playful or active than usual? | O No | O A little | O A lot |
| 12.6 | Over the past 12 h, has your child been eating less than usual? | O No | O A little | O A lot |
| 12.7 | Over the past 12 h, has your child been having fever or feeling warm to touch? | O No | O A little | O A lot |

| | ☐ Very easy | ☐ Easy | ☐ Neutral | ☐ Difficult | ☐ Very difficult |
|---|---|---|---|---|---|
| Apabila jawaban Anda 'Difficult' atau 'Very difficult', mohon berikan tanda centang di kotak yang sesuai atau berikan alasan Anda. Anda dipersilahkan untuk memilih lebih dari satu jawaban. | ☐ It was difficult to understand the question(s)<br>☐ The options of answers were confusing<br>☐ The provided instruction in the form was unclear<br>☐ The question(s) was not suitable for my child, therefore I did not know how to answer the question(s): question no ____ ; ____ : ____<br>☐ I do not know how to complete the questionnaire | | | | |

| | | | | | |
|---|---|---|---|---|---|
| | ☐ Others : ______ | | | | |
| How do you rate the process in completing **the overall symptom diary**? | ☐ Very easy | ☐ Easy | ☐ Neutral | ☐ Difficult | ☐ Very difficult |
| If your answer 'difficult' or 'very difficult', please place a checkmark (V) in the box corresponding to or write you reason(s). You may choose more than one. | ☐ I did not understand how to complete this diary<br>☐ I need more information from my doctor in how to complete this diary<br>☐ Instuctions provided in the diary are unclear<br>☐ Time consuming<br>☐ Too many questions that I did not think they were relevant with my child's condition<br>☐ The sequence of the questions was too confusing<br>☐ Others : ______ | | | | |

**Pharmacist ID** : |___|___|___|___| **Hospital ID** : |___|___|___|

# FORM02 – STUDY MEDICATION STOCK FORM

| Date received | No. of tablets | Batch number | Name who received the medication | Signature | Date checked | No. of tablets | Batch number | Name who checked the medication | Signature | Notes |
|---|---|---|---|---|---|---|---|---|---|---|
| | | | | | | | | | | |
| | | | | | | | | | | |
| | | | | | | | | | | |
| | | | | | | | | | | |
| | | | | | | | | | | |
| | | | | | | | | | | |
| | | | | | | | | | | |
| | | | | | | | | | | |
| | | | | | | | | | | |
| | | | | | | | | | | |
| | | | | | | | | | | |
| | | | | | | | | | | |
| | | | | | | | | | | |
| | | | | | | | | | | |
| | | | | | | | | | | |

**Pharmacy ID** : |___|___|___|___| **Hospital ID** : |___|___|___|

## FORM03 – STUDY MEDICATION DISPENSING FORM (FOR PHARMACY)

| Randomisation/ Registration ID | Date dispensed | Dose (mg/day) | Number of doses (day) | Initial dose (5 days) or additional dose |
|---|---|---|---|---|
| | | | | |
| | | | | |
| | | | | |
| | | | | |
| | | | | |
| | | | | |
| | | | | |
| | | | | |
| | | | | |
| | | | | |
| | | | | |
| | | | | |
| | | | | |
| | | | | |
| | | | | |
| | | | | |
| | | | | |
| | | | | |
| | | | | |
| | | | | |
| | | | | |
| | | | | |
| | | | | |
| | | | | |
| | | | | |
| | | | | |
| | | | | |

**Nurse ID** : |___|___|___|___| **Hospital ID** : |___|___|___|

| FORM04 – STUDY MEDICATION RETUN FORM (FOR NURSE) | | | |
|---|---|---|---|
| **Randomisation/ Registration ID** | **Date returned** | **Number of left-over drug** | **Reason for return** |
| | | | |
| | | | |
| | | | |
| | | | |
| | | | |
| | | | |
| | | | |
| | | | |
| | | | |
| | | | |
| | | | |
| | | | |
| | | | |
| | | | |
| | | | |
| | | | |
| | | | |
| | | | |
| | | | |
| | | | |
| | | | |
| | | | |
| | | | |
| | | | |
| | | | |
| | | | |

# FORM05 – COMPLETED CASE REPORT FORM (FOR NURSE WHO PERFORM A RANDOMISATION)

| Nurse ID : | Protocol title: Oral prednisolone for acute otitis media in children: a pilot pragmatic, randomised, open-label single-blind study (OPAL study) | Hospital ID : |
|---|---|---|

| No | Randomisation ID | Date enrolled to the study | Date of Visit-1* (Day-3) | Date of Visit-2* (Day-7) | Date of Visit-3* (Day-30) | Date of Visit-4* (Day-90) | Date of completion of the study |
|---|---|---|---|---|---|---|---|
| | | | *Please write the checkmark (V) if the study participant come to each visit | | | | |
| | | | | | | | |
| | | | | | | | |
| | | | | | | | |
| | | | | | | | |
| | | | | | | | |
| | | | | | | | |
| | | | | | | | |
| | | | | | | | |
| | | | | | | | |
| | | | | | | | |
| | | | | | | | |
| | | | | | | | |

# FORM06 - RECAPITULATION OF NON-PARTICIPATING SUBJECT FORM (FOR NURSE WHO PERFORM A RANDOMISATION)

| Nurse ID : | Protocol title: Oral prednisolone for acute otitis media in children: a pilot pragmatic, randomised, open-label single-blind study (OPAL study) | Hospital ID : |
|---|---|---|

| No | Registration ID | Date subject offered into the study | Date subject not participate in the study | Reasons<br>*Please write the checkmark (V) if the study participant come to each visit* | | |
|---|---|---|---|---|---|---|
| | | | | Not eligible | Refuse to participate in the study | Others |
| | | | | | | |
| | | | | | | |
| | | | | | | |
| | | | | | | |
| | | | | | | |
| | | | | | | |
| | | | | | | |
| | | | | | | |
| | | | | | | |
| | | | | | | |
| | | | | | | |
| | | | | | | |

# FORM07. GUIDELINE OF ANTIBIOTICS FOR ACUTE OTITIS MEDIA

| Initial immediate or delayed antibiotic therapy | | Antibiotics after 48-72 hours of failure of initial antibiotic therapy | |
|---|---|---|---|
| **Recommended first-line treatment** | **Alternative treatment (if penicillin allergy)** | **Recommended first-line treatment** | **Alternative treatment** |
| Amoxicillin (80-90 mg/kg per day in 2 divided doses)<br><br>OR<br><br>Amoxicillin-clavulanate[a] (90 mg/kg per day of amoxicillin, with 6.4 mg/kg per day clavulanate (amoxicillin to clavulanate ration, 14:1) in 2 divided doses) | Cefdinir (14 mg/kg per day in 1 or 2 doses)<br><br>Cefuroxime (30 mg/kg per day in 2 divided doses)<br><br>Cefpodoxime (10 mg/kg per day in 2 divided doses)<br><br>Ceftriaxone (50 mg IM or IV per day for 1 or 3 days) | Amoxicillin-clavulanate[a] (90 mg/kg per day of amoxicillin, with 6.4 mg/kg per day in 2 divided doses)<br><br>OR<br><br>Ceftriaxone (50 mg IM or IV per day for 3 days) | Ceftriaxone, 3 days Clindamycin (30-40 mg/kg per day in 3 divided doses), with or without third-generation cephalosporin (50 mg IM or IV per day for 3 days)<br>Failure of second antibiotic<br>Clindamycin (30-40 mg/kg per day in 3 divided doses) plus third-generation cephalosporin<br>Tympanocentesis[b]<br>Consult specialist[b] |

[a] *may be considered in patients who have received amoxicillin in the previous 3 0 days or who have the otitis conjunctivitis syndrome;*
[b] *Perform tympanocentesis/drainage if skilled in the procedure, or seek a consultation from an otolaryngologist for tympanocentesis/drainage if the tympanocentesis reveals multidrug/resistant bacteria, seek an infection disease specialist consultation.*

Reference: Lieberthal AS, Carroll AE, Chonmaitree T, et al. Clinical Practice Guideline: The diagnosis and management of acute otitis media. The American Academy of Pediatrics. *Pediatrics.* 2013;131:e964-e99

dr. Respati W. Ranakusuma, SpTHT-KL
Clinical Epidemiology & Evidence-Based Medicine Unit, Dr. Cipto Mangunkusumo Hospital – Faculty of Medicine Universitas Indonesia


# FORM08 – PREDNISOLONE DOSE FOR OPAL STUDY

| Age | Dose | Tablets |
|---|---|---|
| Aged six months to up to two years old | Dose : 10 mg / day (2 tablets per day) | **10 tablets** of Prednisolone for five days |
| Aged two years to up to six years old | Dose : 20 mg / day (4 tablets per day) | **20 tablets** of Prednisolone for five days |
| Aged six years to 12 years old | Dose : 30 mg / day (6 tablets per day) | **30 tablets** of Prednisolone for five days |

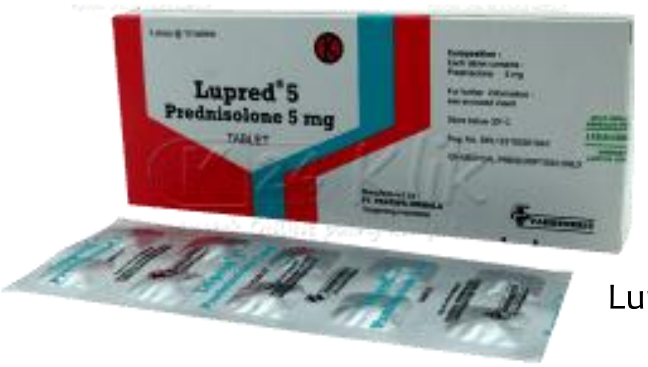


Lupred® 5 contains 5 mg prednisolone in each tablet

# Instruction for using Prednisolone

We copied cited and copied the information on the leaflet from:
Medicine for children – information for parents and carers: prednisolone for asthma.
http://www.medicinesforchildren.org.uk/prednisolone-asthma

This leaflet has been written for parents and carers about how to use this medication in children. This information may differ from that provided by the pharmaceutical company, because their information is usually aimed at adult patients. Please read this leaflet carefully.

## Name of drug

Lupred tablet contains of prednisolone.

## When should I give prednisolone?

Prednisolone is usually given **once** each day, usually in the morning. Give the medicine at about the same time each day so that this becomes part of your child's daily routine, which will help you to remember.

## How much should I give?

Your doctor will work out the amount (the dose) that is right for your child. It is important that you follow your doctor's instructions about how much to give.

## How should I give it?

The pharmacist will prepare the prednisolone tablets by crushing the tablets, mixing it with the sweetener, and packing them in a daily paper-pack for your child.

You can mix it with a small amount of soft food such as yogurt, honey, or jam, or give a glass of milk or juice. Make sure your child swallows it straight away, without chewing.

## When should the medicine start working?

Prednisolone usually takes 4–6 hours to have its full effect.

## What if my child is sick (vomits)?

If your child is sick less than 30 minutes after having a dose of prednisolone, give them the same dose again.

If your child is sick more than 30 minutes after having a dose of prednisolone, you do not need to give them another dose. Wait until the next normal dose.

If your child is sick again, please contact us.

## What if I forget to give it?

You can give your child the missed dose as soon as you remember on the same day. If you remember after they have gone to bed, do not give them the missed dose. Give the next dose in the morning as usual. Never give a double dose of prednisolone

## What if I give too much?

It can be dangerous to give too much prednisolone. If you think you may have given your child too much prednisolone, contact us immediately.

## Are there any possible side-effects?

We use medicines to make our children better, but sometimes they have other effects that we don't want (side-effects). It is unlikely that your child will have side-effects if they only take prednisolone for a few days. They are more likely to get side-effects if they are on a high dose, have extra doses or take prednisolone for a long time.

Side effects that you must do something about

- If your child has bad stomach pain or repeated vomiting (being sick), contact us straight away. This may be due to an ulcer or inflammation of the pancreas
- If your child develops a rash or severe/unexplained bruising, contact us straight away, as there may be a problem with your child's blood
- If your child has eye pain or changes in their vision, contact us straight away

Other side effects you need to know about

- child may have stomach ache, feel sick or be sick (vomit) or may have indigestion (heartburn). Giving the medicine with some food may help
- Your child may have an increased appetite and may gain weight while taking prednisolone. You can help by making sure your child has plenty of physical activity, and by offering fruit and vegetables and low-calorie food, rather than food that is high in calories (e.g. cakes, biscuits, sweets)
- Your child may have trouble sleeping and nightmares and may feel depressed, or their behaviour may change in other ways. Contact us for advice if you are concerned

Side effects with high doses or long courses

- Prednisolone can slow growth and affect puberty. It can also cause growth of body hair and irregular periods in girls
- Your child may be more at risk of severe infections. They should stay away from anyone with an infection (such as chicken pox, shingles, measles) if they have not had these illnesses or have not been vaccinated for measles
- If your child is unwell and you are worried about an infection, contact us straight away
- Your child's skin may become thinner, and heal more slowly than usual. Acne (spots) may become worse or your child may develop mouth ulcers or thrush (candidiasis). If you are concerned, contact us
- Your child may develop problems with their hip bones or their bones may become weaker (osteoporosis). The muscles around the hips and shoulders may also become weaker. If your child has any difficulty walking or moving around, contact us
- Occasionally, prednisolone causes diabetes. If your child seems more thirsty than normal, needs to pass urine (wee) often, or starts wetting the bed at night, contact us

There may, sometimes, be other side-effects that are not listed. above. If you notice anything unusual and are concerned, please contact us.

## Can other medicines be given at the same time?

You can give your child medicines that contain paracetamol or ibuprofen, unless your doctor has told you not to. Check with us or your doctor before giving any other medicines to your child. This includes herbal or complimentary medicines.

## Is there anything else I need to know about prednisolone?

For children who have been taking prednisolone in high doses or for longer than 2-3 weeks

- They must not stop taking the medicine suddenly because they may get withdrawal symptoms: they will feel unwell, dizzy and thirsty and may be sick (vomit). If this occurs, you should contact us straight away
- If your doctor decides to stop prednisolone, they will reduce the dose gradually before stopping it completely. Make sure you follow your doctor's instructions
- Make sure that you always have enough medicine.

## Where should I keep this medicine?

- Keep the medicine in a cupboard, away from heat and direct sunlight. It does not need to be kept in the fridge
- Make sure that children cannot see or reach it.
- Keep the medicine in the container it came in

| WHO TO CONTACT FOR MORE INFORMATION |
| --- |
| OPAL STUDY 24-HOUR CALL CENTRE<br>08111 012 185 |

# FORM01 – STUDY RECRUITMENT LOG BOOK

| **Nurse name/ID :** | **Study title :** Oral prednisolone for acute otitis media in children: a pilot, pragmatic, randomised, open-label, single-blind, controlled study (OPAL study) | **Hospital ID :** |
|---|---|---|

| Study registration ID | Patient's name | Date screened | Has your child experien-cing ear pain in the past 48 hours? (YES or NO) | Has your child been tugging or rubbing her/his ear(s) and been more irritable or fussy or crying more than usual over the past 48 hours (YES or NO) | Has your child been experie ncing ear discharg e in the past 48 hours? (YES or NO) | Body weight (kg) | Body height (cm) | Body tempe-rature (°C) | Blood pressure (mmHg) | Did patient go on the study? (YES or NO) | If YES, what is the Randomisation ID | If NO, please tell us reason not on the study below | | |
|---|---|---|---|---|---|---|---|---|---|---|---|---|---|---|
| | | | | | | | | | | | | Not eligible (YES or NO) | Did not give consent (YES or NO) | Was not approached (YES or NO). Write the reason |
| | | | | | | | | | | | | | | |
| | | | | | | | | | | | | | | |
| | | | | | | | | | | | | | | |
| | | | | | | | | | | | | | | |
| | | | | | | | | | | | | | | |

Please write 'Y' for 'YES' and 'N' for 'NO'.

# DIARY-1 (Day-0 to Day-3)

OPAL Study

Oral Prednisolone for Acute otitis media in children

Registration ID

Hello Uncle / Aunty!!

My name is______________________

I was born in_________________

On date _____ month ________ year ___________

If you find this Diary, I would be very grateful if you can return it to my Dad (mobile no. _______________) or my Mom (mobile no. _______________).

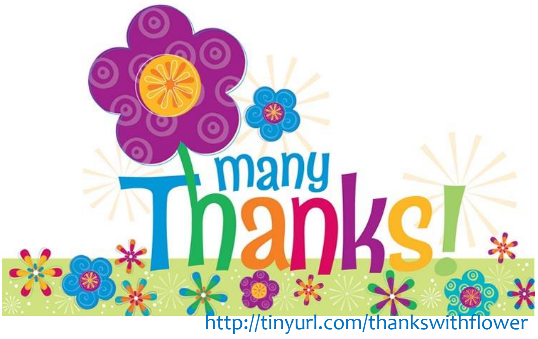


http://tinyurl.com/thankswithflower

Registration ID

# Day-0 (*your first visit*) : |___|___| – |___|___| – 20 |___|___|

**1. Please place a vertical line across the available horizontal line that best describes your or your child's pain during the past 12 hours? Please write the time accordingly ……. (am/ pm)**

No Pain ———————————————— Pain As Bad As It Could Possibly Be

**2. We are interest finding out how your child has been doing. For each question, please place a check mark in O corresponding to your child's symptoms. Please answer all questions. Please write the time accordingly ……. (am/ pm)**

| | | | |
|---|---|---|---|
| 2.1 Over the past 12 h, has your child been tugging, rubbing, or holding the ear(s) more than usual? | O No | O A little | O A lot |
| 2.2 Over the past 12 h, has your child been crying more than usual? | O No | O A little | O A lot |
| 2.3 Over the past 12 h, has your child been more irritable or fussy than usual? | O No | O A little | O A lot |
| 2.4 Over the past 12 h, has your child been having more difficulty sleeping than usual? | O No | O A little | O A lot |
| 2.5 Over the past 12 h, has your child been less playful or active than usual? | O No | O A little | O A lot |
| 2.6 Over the past 12 h, has your child been eating less than usual? | O No | O A little | O A lot |
| 2.7 Over the past 12 h, has your child been having fever or feeling warm to touch? | O No | O A little | O A lot |

**Other symptoms**

| | | |
|---|---|---|
| 3 Does your child experience discharge from the ear(s)? | O Yes | O No |
| 4 Does your child experience intense ear pain and pain behind the ear? | O Yes | O No |
| 5 Does your child experience swelling/bulging, redness, tenderness, or dropping behind or of the ear(s) | O Yes | O No |
| 6 Does your child experience facial asymmetry (e.g. when the child smiles, cries)? | O Yes | O No |

**Medicines given (please write the name, dose, and frequency)**

| | | | |
|---|---|---|---|
| Medicines have been given to your child before going to the hospital (from other doctor or chemist store) | ______________ | Dose : _____ mg / body weight kg | Frequency : ______ / day |
| | ______________ | Dose : _____ mg / body weight kg | Frequency : ______ / day |
| | ______________ | Dose : _____ mg / body weight kg | Frequency : ______ / day |
| | ______________ | Dose : _____ mg / body weight kg | Frequency : ______ / day |
| | ______________ | Dose : _____ mg / body weight kg | Frequency : ______ / day |

**Please list all medicines you give to your child today by marking the circle based on the frequency and the time**

| | | | | | |
|---|---|---|---|---|---|
| ______________ | O ___ am/pm | O ___ am/pm | O ___ am/pm | O ___ am/pm | O ___ am/pm |
| ______________ | O ___ am/pm | O ___ am/pm | O ___ am/pm | O ___ am/pm | O ___ am/pm |
| ______________ | O ___ am/pm | O ___ am/pm | O ___ am/pm | O ___ am/pm | O ___ am/pm |
| ______________ | O ___ am/pm | O ___ am/pm | O ___ am/pm | O ___ am/pm | O ___ am/pm |
| ______________ | O ___ am/pm | O ___ am/pm | O ___ am/pm | O ___ am/pm | O ___ am/pm |
| ______________ | O ___ am/pm | O ___ am/pm | O ___ am/pm | O ___ am/pm | O ___ am/pm |
| ______________ | O ___ am/pm | O ___ am/pm | O ___ am/pm | O ___ am/pm | O ___ am/pm |

*For each question, please tick (✓) your answer on O or write you answer on ______*

*Thank you for filling the diary today.*
*Now please give your child the study medicine.*

**Notes:**

Registration ID

## Day – 1* : |___|___| – |___|___| – 20 |___|___|

## ***On the morning after your first visit to the hospital or doctor**

**1. Please place a vertical line across the available horizontal line that best describes your or your child's pain during the past 12 hours? Please write the time accordingly ……. (am/ pm)**

No Pain ———————————————— Pain As Bad As It Could Possibly Be

**2. We are interest finding out how your child has been doing. For each question, please place a check mark in O corresponding to your child's symptoms. Please answer all questions. Please write the time accordingly ……. (am/ pm)**

| | | | |
|---|---|---|---|
| 2.1 Over the past 12 h, has your child been tugging, rubbing, or holding the ear(s) more than usual? | O No | O A little | O A lot |
| 2.2 Over the past 12 h, has your child been crying more than usual? | O No | O A little | O A lot |
| 2.3 Over the past 12 h, has your child been more irritable or fussy than usual? | O No | O A little | O A lot |
| 2.4 Over the past 12 h, has your child been having more difficulty sleeping than usual? | O No | O A little | O A lot |
| 2.5 Over the past 12 h, has your child been less playful or active than usual? | O No | O A little | O A lot |
| 2.6 Over the past 12 h, has your child been eating less than usual? | O No | O A little | O A lot |
| 2.7 Over the past 12 h, has your child been having fever or feeling warm to touch? | O No | O A little | O A lot |

**Other symptoms**

| | | |
|---|---|---|
| 3 Does your child experience discharge from the ear(s)? | O Yes | O No |
| 4 Does your child experience intense ear pain and pain behind the ear? | O Yes | O No |
| 5 Does your child experience swelling/bulging, redness, tenderness, or dropping behind or of the ear(s) | O Yes | O No |
| 6 Does your child experience facial asymmetry (e.g. when the child smiles, cries)? | O Yes | O No |

**7 Side effects**

Does your child have these complaints after taking the medicine

| | | | | | |
|---|---|---|---|---|---|
| 7.1 Increased appetite | O Yes | O No | 7.8 Drowsiness | O Yes | O No |
| 7.2 Increased urine amount | O Yes | O No | 7.9 Anxiety/distractibility/mood swing | O Yes | O No |
| 7.3 Weight gain | O Yes | O No | 7.10 Headache | O Yes | O No |
| 7.4 Gastritis/abdominal pain | O Yes | O No | 7.11 Skin rash or diaper rash | O Yes | O No |
| 7.5 Nausea | O Yes | O No | 7.12 Candidiasis | O Yes | O No |
| 7.6 Vomiting | O Yes | O No | 7.13 Dry mouth / throat irritation | O Yes | O No |
| 7.7 Diarrhea | O Yes | O No | 7.14 Sleep disturbance | O Yes | O No |
| Others | ______ | | | | |
| Did you bring your child to doctor (clinic or outpatient)? | O Yes | O No | Reason: | ______ | |
| | | | Medicine prescribed: | ______ | |

*For each question, please tick (✓) your answer on O or write you answer on ______*

| | | | |
|---|---|---|---|
| Has your child has been admitted to hospital? | O Yes O No | Reason: | ______________ |
| | | Medicine prescribed: | ______________ |

| **Medicines given (please write the name, dose, and frequency)** | | | |
|---|---|---|---|
| Additional medicine from the chemist store or other (not precribed by your doctor) | ______________ | Dose : _____ mg / body weight kg | Frequency : ______ / day |
| | ______________ | Dose : _____ mg / body weight kg | Frequency : ______ / day |
| | ______________ | Dose : _____ mg / body weight kg | Frequency : ______ / day |
| | ______________ | Dose : _____ mg / body weight kg | Frequency : ______ / day |
| | ______________ | Dose : _____ mg / body weight kg | Frequency : ______ / day |

| **Please list all medicines you give to your child today by marking the circle based on the frequency and the time** | | | | | |
|---|---|---|---|---|---|
| ______________ | O ____ am/pm | O ____ am/pm | O ____ am/pm | O ____ am/pm | O ____ am/pm |
| ______________ | O ____ am/pm | O ____ am/pm | O ____ am/pm | O ____ am/pm | O ____ am/pm |
| ______________ | O ____ am/pm | O ____ am/pm | O ____ am/pm | O ____ am/pm | O ____ am/pm |
| ______________ | O ____ am/pm | O ____ am/pm | O ____ am/pm | O ____ am/pm | O ____ am/pm |
| ______________ | O ____ am/pm | O ____ am/pm | O ____ am/pm | O ____ am/pm | O ____ am/pm |
| ______________ | O ____ am/pm | O ____ am/pm | O ____ am/pm | O ____ am/pm | O ____ am/pm |
| ______________ | O ____ am/pm | O ____ am/pm | O ____ am/pm | O ____ am/pm | O ____ am/pm |

*Thank you for filling the diary today.*
*Now please give your child the study medicine.*

**Notes:**

# Day-2 : |___|___| – |___|___| – 20 |___|___|

**1. Please place a vertical line across the available horizontal line that best describes your or your child's pain during the past 12 hours? Please write the time accordingly ……. (am/ pm)**

No Pain |————————————————| Pain As Bad As It Could Possibly Be

**2. We are interest finding out how your child has been doing. For each question, please place a check mark in O corresponding to your child's symptoms. Please answer all questions. Please write the time accordingly ……. (am/ pm)**

| | | | |
|---|---|---|---|
| 2.1 Over the past 12 h, has your child been tugging, rubbing, or holding the ear(s) more than usual? | O No | O A little | O A lot |
| 2.2 Over the past 12 h, has your child been crying more than usual? | O No | O A little | O A lot |
| 2.3 Over the past 12 h, has your child been more irritable or fussy than usual? | O No | O A little | O A lot |
| 2.4 Over the past 12 h, has your child been having more difficulty sleeping than usual? | O No | O A little | O A lot |
| 2.5 Over the past 12 h, has your child been less playful or active than usual? | O No | O A little | O A lot |
| 2.6 Over the past 12 h, has your child been eating less than usual? | O No | O A little | O A lot |
| 2.7 Over the past 12 h, has your child been having fever or feeling warm to touch? | O No | O A little | O A lot |

**Other symptoms**

| | | |
|---|---|---|
| 3 Does your child experience discharge from the ear(s)? | O Yes | O No |
| 4 Does your child experience intense ear pain and pain behind the ear? | O Yes | O No |
| 5 Does your child experience swelling/bulging, redness, tenderness, or dropping behind or of the ear(s) | O Yes | O No |
| 6 Does your child experience facial asymmetry (e.g. when the child smiles, cries)? | O Yes | O No |

**Medicines given (please write the name, dose, and frequency)**

| | | | |
|---|---|---|---|
| Medicines have been given to your child before going to the hospital (from other doctor or chemist store) | ________________ | Dose : _____ mg / body weight kg | Frequency : ______ / day |
| | ________________ | Dose : _____ mg / body weight kg | Frequency : ______ / day |
| | ________________ | Dose : _____ mg / body weight kg | Frequency : ______ / day |
| | ________________ | Dose : _____ mg / body weight kg | Frequency : ______ / day |
| | ________________ | Dose : _____ mg / body weight kg | Frequency : ______ / day |

**7 Side effects**

Does your child have these complaints after taking the medicine

| | | | | | |
|---|---|---|---|---|---|
| 7.1 Increased appetite | O Yes | O No | 7.8 Drowsiness | O Yes | O No |
| 7.2 Increased urine amount | O Yes | O No | 7.9 Anxiety/distractibility/mood swing | O Yes | O No |
| 7.3 Weight gain | O Yes | O No | 7.10 Headache | O Yes | O No |
| 7.4 Gastritis/abdominal pain | O Yes | O No | 7.11 Skin rash or diaper rash | O Yes | O No |
| 7.5 Nausea | O Yes | O No | 7.12 Candidiasis | O Yes | O No |
| 7.6 Vomiting | O Yes | O No | 7.13 Dry mouth / throat irritation | O Yes | O No |
| 7.7 Diarrhea | O Yes | O No | 7.14 Sleep disturbance | O Yes | O No |

*For each question, please tick (✓) your answer on O or write you answer on ______*

| | | | |
|---|---|---|---|
| Others | ____________________ | | |
| Did you bring your child to doctor (clinic or outpatient)? | O Yes O No | Reason: | ____________________ |
| | | Medicine prescribed: | ____________________ |
| Has your child has been admitted to hospital? | O Yes O No | Reason: | ____________________ |
| | | Medicine prescribed: | ____________________ |

**Medicines given (please write the name, dose, and frequency)**

| | | | |
|---|---|---|---|
| Medicines have been given to your child before going to the hospital (from other doctor or chemist store) | ____________________ | Dose : _____ mg / body weight kg | Frequency : ______ / day |
| | ____________________ | Dose : _____ mg / body weight kg | Frequency : ______ / day |
| | ____________________ | Dose : _____ mg / body weight kg | Frequency : ______ / day |
| | ____________________ | Dose : _____ mg / body weight kg | Frequency : ______ / day |
| | ____________________ | Dose : _____ mg / body weight kg | Frequency : ______ / day |

**Please list all medicines you give to your child today by marking the circle based on the frequency and the time**

| | | | | | |
|---|---|---|---|---|---|
| ____________________ | O ___ am/pm | O ___ am/pm | O ___ am/pm | O ___ am/pm | O ___ am/pm |
| ____________________ | O ___ am/pm | O ___ am/pm | O ___ am/pm | O ___ am/pm | O ___ am/pm |
| ____________________ | O ___ am/pm | O ___ am/pm | O ___ am/pm | O ___ am/pm | O ___ am/pm |
| ____________________ | O ___ am/pm | O ___ am/pm | O ___ am/pm | O ___ am/pm | O ___ am/pm |
| ____________________ | O ___ am/pm | O ___ am/pm | O ___ am/pm | O ___ am/pm | O ___ am/pm |
| ____________________ | O ___ am/pm | O ___ am/pm | O ___ am/pm | O ___ am/pm | O ___ am/pm |
| ____________________ | O ___ am/pm | O ___ am/pm | O ___ am/pm | O ___ am/pm | O ___ am/pm |

*Thank you for filling the diary today.*
*Now please give your child the study medicine.*

**Notes:**

# Day – 3 (1st Follow-up Visit): |___|___| – |___|___| – 20 |___|___|

**1. Please place a vertical line across the available horizontal line that best describes your or your child's pain during the past 12 hours? Please write the time accordingly ……. (am/ pm)**

No Pain ———————————————— Pain As Bad As It Could Possibly Be

**2. We are interest finding out how your child has been doing. For each question, please place a check mark in O corresponding to your child's symptoms. Please answer all questions. Please write the time accordingly ……. (am/ pm)**

| | | | |
|---|---|---|---|
| 2.1 Over the past 12 h, has your child been tugging, rubbing, or holding the ear(s) more than usual? | O No | O A little | O A lot |
| 2.2 Over the past 12 h, has your child been crying more than usual? | O No | O A little | O A lot |
| 2.3 Over the past 12 h, has your child been more irritable or fussy than usual? | O No | O A little | O A lot |
| 2.4 Over the past 12 h, has your child been having more difficulty sleeping than usual? | O No | O A little | O A lot |
| 2.5 Over the past 12 h, has your child been less playful or active than usual? | O No | O A little | O A lot |
| 2.6 Over the past 12 h, has your child been eating less than usual? | O No | O A little | O A lot |
| 2.7 Over the past 12 h, has your child been having fever or feeling warm to touch? | O No | O A little | O A lot |

**Other symptoms**

| | | |
|---|---|---|
| 3 Does your child experience discharge from the ear(s)? | O Yes | O No |
| 4 Does your child experience intense ear pain and pain behind the ear? | O Yes | O No |
| 5 Does your child experience swelling/bulging, redness, tenderness, or dropping behind or of the ear(s) | O Yes | O No |
| 6 Does your child experience facial asymmetry (e.g. when the child smiles, cries)? | O Yes | O No |

**7 Side effects**

Does your child have these complaints after taking the medicine

| | | | | | |
|---|---|---|---|---|---|
| 7.1 Increased appetite | O Yes | O No | 7.8 Drowsiness | O Yes | O No |
| 7.2 Increased urine amount | O Yes | O No | 7.9 Anxiety/distractibility/mood swing | O Yes | O No |
| 7.3 Weight gain | O Yes | O No | 7.10 Headache | O Yes | O No |
| 7.4 Gastritis/abdominal pain | O Yes | O No | 7.11 Skin rash or diaper rash | O Yes | O No |
| 7.5 Nausea | O Yes | O No | 7.12 Candidiasis | O Yes | O No |
| 7.6 Vomiting | O Yes | O No | 7.13 Dry mouth / throat irritation | O Yes | O No |
| 7.7 Diarrhea | O Yes | O No | 7.14 Sleep disturbance | O Yes | O No |

Others ________________________________________

| | | | | |
|---|---|---|---|---|
| Did you bring your child to doctor (clinic or outpatient)? | O Yes | O No | Reason: | ____________ |
| | | | Medicine prescribed: | ____________ |
| Has your child has been admitted to hospital? | O Yes | O No | Reason: | ____________ |
| | | | Medicine prescribed: | ____________ |

*For each question, please tick (✓) your answer on O or write you answer on ______*

**Registration ID** | |

| **Medicines given (please write the name, dose, and frequency)** | | | |
|---|---|---|---|
| Additional medicine from the chemist store or other (not precribed by your doctor) | ________________ | Dose : _____ mg / body weight kg | Frequency : ______ / day |
| | ________________ | Dose : _____ mg / body weight kg | Frequency : ______ / day |
| | ________________ | Dose : _____ mg / body weight kg | Frequency : ______ / day |
| | ________________ | Dose : _____ mg / body weight kg | Frequency : ______ / day |
| | ________________ | Dose : _____ mg / body weight kg | Frequency : ______ / day |

| **Please list all medicines you give to your child today by marking the circle based on the frequency and the time** | | | | | |
|---|---|---|---|---|---|
| ________________ | O ____ am/pm | O ____ am/pm | O ____ am/pm | O ____ am/pm | O ____ am/pm |
| ________________ | O ____ am/pm | O ____ am/pm | O ____ am/pm | O ____ am/pm | O ____ am/pm |
| ________________ | O ____ am/pm | O ____ am/pm | O ____ am/pm | O ____ am/pm | O ____ am/pm |
| ________________ | O ____ am/pm | O ____ am/pm | O ____ am/pm | O ____ am/pm | O ____ am/pm |
| ________________ | O ____ am/pm | O ____ am/pm | O ____ am/pm | O ____ am/pm | O ____ am/pm |
| ________________ | O ____ am/pm | O ____ am/pm | O ____ am/pm | O ____ am/pm | O ____ am/pm |
| ________________ | O ____ am/pm | O ____ am/pm | O ____ am/pm | O ____ am/pm | O ____ am/pm |

*Thank you for filling the diary today.*
*Now please give your child the study medicine.*

**Notes:**

*Thank you for completing the first Diary.*

**Registration ID** | |

# Additional Visit : |___|___| – |___|___| – 20 |___|___|

**1. Please place a vertical line across the available horizontal line that best describes your or your child's pain during the past 12 hours? Please write the time accordingly ……. (am/ pm)**

No Pain |————————————————| Pain As Bad As It Could Possibly Be

**2. We are interest finding out how your child has been doing. For each question, please place a check mark in O corresponding to your child's symptoms. Please answer all questions. Please write the time accordingly ……. (am/ pm)**

| | | | |
|---|---|---|---|
| 2.1 Over the past 12 h, has your child been tugging, rubbing, or holding the ear(s) more than usual? | O No | O A little | O A lot |
| 2.2 Over the past 12 h, has your child been crying more than usual? | O No | O A little | O A lot |
| 2.3 Over the past 12 h, has your child been more irritable or fussy than usual? | O No | O A little | O A lot |
| 2.4 Over the past 12 h, has your child been having more difficulty sleeping than usual? | O No | O A little | O A lot |
| 2.5 Over the past 12 h, has your child been less playful or active than usual? | O No | O A little | O A lot |
| 2.6 Over the past 12 h, has your child been eating less than usual? | O No | O A little | O A lot |
| 2.7 Over the past 12 h, has your child been having fever or feeling warm to touch? | O No | O A little | O A lot |

**Other symptoms**

| | | |
|---|---|---|
| 3 Does your child experience discharge from the ear(s)? | O Yes | O No |
| 4 Does your child experience intense ear pain and pain behind the ear? | O Yes | O No |
| 5 Does your child experience swelling/bulging, redness, tenderness, or dropping behind or of the ear(s) | O Yes | O No |
| 6 Does your child experience facial asymmetry (e.g. when the child smiles, cries)? | O Yes | O No |

**7 Side effects**

Does your child have these complaints after taking the medicine

| | | | | | |
|---|---|---|---|---|---|
| 7.1 Increased appetite | O Yes | O No | 7.8 Drowsiness | O Yes | O No |
| 7.2 Increased urine amount | O Yes | O No | 7.9 Anxiety/distractibility/mood swing | O Yes | O No |
| 7.3 Weight gain | O Yes | O No | 7.10 Headache | O Yes | O No |
| 7.4 Gastritis/abdominal pain | O Yes | O No | 7.11 Skin rash or diaper rash | O Yes | O No |
| 7.5 Nausea | O Yes | O No | 7.12 Candidiasis | O Yes | O No |
| 7.6 Vomiting | O Yes | O No | 7.13 Dry mouth / throat irritation | O Yes | O No |
| 7.7 Diarrhea | O Yes | O No | 7.14 Sleep disturbance | O Yes | O No |
| Others | ______________________ | | | | |

| | | | | |
|---|---|---|---|---|
| Did you bring your child to doctor (clinic or outpatient)? | O Yes | O No | Reason:<br>Medicine prescribed: | ______________<br>______________ |
| Has your child has been admitted to hospital? | O Yes | O No | Reason:<br>Medicine prescribed: | ______________<br>______________ |

*For each question, please tick (✓) your answer on O or write you answer on ______*

| **Medicines given (please write the name, dose, and frequency)** | | | |
|---|---|---|---|
| Additional medicine from the chemist store or other (not precribed by your doctor) | ______________________ | Dose : _____ mg / body weight kg | Frequency : ______ / day |
| | ______________________ | Dose : _____ mg / body weight kg | Frequency : ______ / day |
| | ______________________ | Dose : _____ mg / body weight kg | Frequency : ______ / day |
| | ______________________ | Dose : _____ mg / body weight kg | Frequency : ______ / day |
| | ______________________ | Dose : _____ mg / body weight kg | Frequency : ______ / day |

| **Please list all medicines you give to your child today by marking the circle based on the frequency and the time** | | | | | |
|---|---|---|---|---|---|
| ______________________ | O ____ am/pm | O ____ am/pm | O ____ am/pm | O ____ am/pm | O ____ am/pm |
| ______________________ | O ____ am/pm | O ____ am/pm | O ____ am/pm | O ____ am/pm | O ____ am/pm |
| ______________________ | O ____ am/pm | O ____ am/pm | O ____ am/pm | O ____ am/pm | O ____ am/pm |
| ______________________ | O ____ am/pm | O ____ am/pm | O ____ am/pm | O ____ am/pm | O ____ am/pm |
| ______________________ | O ____ am/pm | O ____ am/pm | O ____ am/pm | O ____ am/pm | O ____ am/pm |
| ______________________ | O ____ am/pm | O ____ am/pm | O ____ am/pm | O ____ am/pm | O ____ am/pm |
| ______________________ | O ____ am/pm | O ____ am/pm | O ____ am/pm | O ____ am/pm | O ____ am/pm |

**Notes:**

# DIARY – 2 (Day-4 to Day-7)

OPAL Study

Oral Prednisolone for Acute otitis media in children

Registration ID

Hello Uncle / Aunty!!

My name is______________________

I was born in________________

On date _____ month ________ year ___________

If you find this Diary, I would be very grateful if you can return it to my Dad (mobile no. _______________) or my Mom (mobile no. _______________).

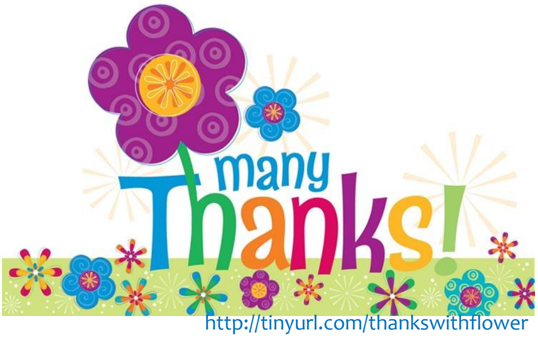


http://tinyurl.com/thankswithflower

# Day-4 : |___|___| – |___|___| – 20 |___|___|

**1. Please place a vertical line across the available horizontal line that best describes your or your child's pain during the past 12 hours? Please write the time accordingly ……. (am/ pm)**

No Pain |——————————————| Pain As Bad As It Could Possibly Be

**2. We are interest finding out how your child has been doing. For each question, please place a check mark in O corresponding to your child's symptoms. Please answer all questions. Please write the time accordingly ……. (am/ pm)**

| | | | |
|---|---|---|---|
| 2.1 Over the past 12 h, has your child been tugging, rubbing, or holding the ear(s) more than usual? | O No | O A little | O A lot |
| 2.2 Over the past 12 h, has your child been crying more than usual? | O No | O A little | O A lot |
| 2.3 Over the past 12 h, has your child been more irritable or fussy than usual? | O No | O A little | O A lot |
| 2.4 Over the past 12 h, has your child been having more difficulty sleeping than usual? | O No | O A little | O A lot |
| 2.5 Over the past 12 h, has your child been less playful or active than usual? | O No | O A little | O A lot |
| 2.6 Over the past 12 h, has your child been eating less than usual? | O No | O A little | O A lot |
| 2.7 Over the past 12 h, has your child been having fever or feeling warm to touch? | O No | O A little | O A lot |

**Other symptoms**

| | | |
|---|---|---|
| 3 Does your child experience discharge from the ear(s)? | O Yes | O No |
| 4 Does your child experience intense ear pain and pain behind the ear? | O Yes | O No |
| 5 Does your child experience swelling/bulging, redness, tenderness, or dropping behind or of the ear(s) | O Yes | O No |
| 6 Does your child experience facial asymmetry (e.g. when the child smiles, cries)? | O Yes | O No |

**7 Side effects**

Does your child have these complaints after taking the medicine

| | | | | | |
|---|---|---|---|---|---|
| 7.1 Increased appetite | O Yes | O No | 7.8 Drowsiness | O Yes | O No |
| 7.2 Increased urine amount | O Yes | O No | 7.9 Anxiety/distractibility/mood swing | O Yes | O No |
| 7.3 Weight gain | O Yes | O No | 7.10 Headache | O Yes | O No |
| 7.4 Gastritis/abdominal pain | O Yes | O No | 7.11 Skin rash or diaper rash | O Yes | O No |
| 7.5 Nausea | O Yes | O No | 7.12 Candidiasis | O Yes | O No |
| 7.6 Vomiting | O Yes | O No | 7.13 Dry mouth / throat irritation | O Yes | O No |
| 7.7 Diarrhea | O Yes | O No | 7.14 Sleep disturbance | O Yes | O No |
| Others | ______ | | | | |

| | | | | |
|---|---|---|---|---|
| Did you bring your child to doctor (clinic or outpatient)? | O Yes | O No | Reason: | ______ |
| | | | Medicine prescribed: | ______ |
| Has your child has been admitted to hospital? | O Yes | O No | Reason: | ______ |
| | | | Medicine prescribed: | ______ |

*For each question, please tick (✓) your answer on O or write you answer on ______*

| **Medicines given (please write the name, dose, and frequency)** | | | |
|---|---|---|---|
| Additional medicine from the chemist store or other (not precribed by your doctor) | ______________ | Dose : _____ mg / body weight kg | Frequency : ______ / day |
| | ______________ | Dose : _____ mg / body weight kg | Frequency : ______ / day |
| | ______________ | Dose : _____ mg / body weight kg | Frequency : ______ / day |
| | ______________ | Dose : _____ mg / body weight kg | Frequency : ______ / day |
| | ______________ | Dose : _____ mg / body weight kg | Frequency : ______ / day |

| **Please list all medicines you give to your child today by marking the circle based on the frequency and the time** | | | | | |
|---|---|---|---|---|---|
| ______________ | O ____ am/pm | O ____ am/pm | O ____ am/pm | O ____ am/pm | O ____ am/pm |
| ______________ | O ____ am/pm | O ____ am/pm | O ____ am/pm | O ____ am/pm | O ____ am/pm |
| ______________ | O ____ am/pm | O ____ am/pm | O ____ am/pm | O ____ am/pm | O ____ am/pm |
| ______________ | O ____ am/pm | O ____ am/pm | O ____ am/pm | O ____ am/pm | O ____ am/pm |
| ______________ | O ____ am/pm | O ____ am/pm | O ____ am/pm | O ____ am/pm | O ____ am/pm |
| ______________ | O ____ am/pm | O ____ am/pm | O ____ am/pm | O ____ am/pm | O ____ am/pm |
| ______________ | O ____ am/pm | O ____ am/pm | O ____ am/pm | O ____ am/pm | O ____ am/pm |

*Thank you for filling the diary today.*
*Now please give your child the study medicine.*

**Notes:**

# Day – 5 : |___|___| – |___|___| – 20 |___|___|

**1. Please place a vertical line across the available horizontal line that best describes your or your child's pain during the past 12 hours? Please write the time accordingly ……. (am/ pm)**

| | |
|---|---|
| No Pain | Pain As Bad As It Could Possibly Be |

**2. We are interest finding out how your child has been doing. For each question, please place a check mark in O corresponding to your child's symptoms. Please answer all questions. Please write the time accordingly …… (am/ pm)**

| | | | |
|---|---|---|---|
| 2.1 Over the past 12 h, has your child been tugging, rubbing, or holding the ear(s) more than usual? | O No | O A little | O A lot |
| 2.2 Over the past 12 h, has your child been crying more than usual? | O No | O A little | O A lot |
| 2.3 Over the past 12 h, has your child been more irritable or fussy than usual? | O No | O A little | O A lot |
| 2.4 Over the past 12 h, has your child been having more difficulty sleeping than usual? | O No | O A little | O A lot |
| 2.5 Over the past 12 h, has your child been less playful or active than usual? | O No | O A little | O A lot |
| 2.6 Over the past 12 h, has your child been eating less than usual? | O No | O A little | O A lot |
| 2.7 Over the past 12 h, has your child been having fever or feeling warm to touch? | O No | O A little | O A lot |

**Other symptoms**

| | | |
|---|---|---|
| 3 Does your child experience discharge from the ear(s)? | O Yes | O No |
| 4 Does your child experience intense ear pain and pain behind the ear? | O Yes | O No |
| 5 Does your child experience swelling/bulging, redness, tenderness, or dropping behind or of the ear(s) | O Yes | O No |
| 6 Does your child experience facial asymmetry (e.g. when the child smiles, cries)? | O Yes | O No |

**7 Side effects**

Does your child have these complaints after taking the medicine

| | | | | | |
|---|---|---|---|---|---|
| 7.1 Increased appetite | O Yes | O No | 7.8 Drowsiness | O Yes | O No |
| 7.2 Increased urine amount | O Yes | O No | 7.9 Anxiety/distractibility/mood swing | O Yes | O No |
| 7.3 Weight gain | O Yes | O No | 7.10 Headache | O Yes | O No |
| 7.4 Gastritis/abdominal pain | O Yes | O No | 7.11 Skin rash or diaper rash | O Yes | O No |
| 7.5 Nausea | O Yes | O No | 7.12 Candidiasis | O Yes | O No |
| 7.6 Vomiting | O Yes | O No | 7.13 Dry mouth / throat irritation | O Yes | O No |
| 7.7 Diarrhea | O Yes | O No | 7.14 Sleep disturbance | O Yes | O No |
| Others | ____________________ | | | | |

| | | | | |
|---|---|---|---|---|
| Did you bring your child to doctor (clinic or outpatient)? | O Yes | O No | Reason: | __________ |
| | | | Medicine prescribed: | __________ |
| Has your child has been admitted to hospital? | O Yes | O No | Reason: | __________ |
| | | | Medicine prescribed: | __________ |

*For each question, please tick (✓) your answer on O or write you answer on ______*

| **Medicines given (please write the name, dose, and frequency)** | | | |
|---|---|---|---|
| Additional medicine from the chemist store or other (not precribed by your doctor) | ____________________ | Dose : _____ mg / body weight kg | Frequency : ______ / day |
| | ____________________ | Dose : _____ mg / body weight kg | Frequency : ______ / day |
| | ____________________ | Dose : _____ mg / body weight kg | Frequency : ______ / day |
| | ____________________ | Dose : _____ mg / body weight kg | Frequency : ______ / day |
| | ____________________ | Dose : _____ mg / body weight kg | Frequency : ______ / day |

| **Please list all medicines you give to your child today by marking the circle based on the frequency and the time** | | | | | |
|---|---|---|---|---|---|
| ____________________ | O ____ am/pm | O ____ am/pm | O ____ am/pm | O ____ am/pm | O ____ am/pm |
| ____________________ | O ____ am/pm | O ____ am/pm | O ____ am/pm | O ____ am/pm | O ____ am/pm |
| ____________________ | O ____ am/pm | O ____ am/pm | O ____ am/pm | O ____ am/pm | O ____ am/pm |
| ____________________ | O ____ am/pm | O ____ am/pm | O ____ am/pm | O ____ am/pm | O ____ am/pm |
| ____________________ | O ____ am/pm | O ____ am/pm | O ____ am/pm | O ____ am/pm | O ____ am/pm |
| ____________________ | O ____ am/pm | O ____ am/pm | O ____ am/pm | O ____ am/pm | O ____ am/pm |
| ____________________ | O ____ am/pm | O ____ am/pm | O ____ am/pm | O ____ am/pm | O ____ am/pm |

*Thank you for filling the diary today.*
*Now please give your child the study medicine.*

**Notes:**

Registration ID

## Day-6 : |___|___| – |___|___| – 20 |___|___|

**1. Please place a vertical line across the available horizontal line that best describes your or your child's pain during the past 12 hours? Please write the time accordingly ……. (am/ pm)**

No Pain ———————————————— Pain As Bad As It Could Possibly Be

**2. We are interest finding out how your child has been doing. For each question, please place a check mark in O corresponding to your child's symptoms. Please answer all questions. Please write the time accordingly ……. (am/ pm)**

| | | | |
|---|---|---|---|
| 2.1 Over the past 12 h, has your child been tugging, rubbing, or holding the ear(s) more than usual? | O No | O A little | O A lot |
| 2.2 Over the past 12 h, has your child been crying more than usual? | O No | O A little | O A lot |
| 2.3 Over the past 12 h, has your child been more irritable or fussy than usual? | O No | O A little | O A lot |
| 2.4 Over the past 12 h, has your child been having more difficulty sleeping than usual? | O No | O A little | O A lot |
| 2.5 Over the past 12 h, has your child been less playful or active than usual? | O No | O A little | O A lot |
| 2.6 Over the past 12 h, has your child been eating less than usual? | O No | O A little | O A lot |
| 2.7 Over the past 12 h, has your child been having fever or feeling warm to touch? | O No | O A little | O A lot |

**Other symptoms**

| | | |
|---|---|---|
| 3 Does your child experience discharge from the ear(s)? | O Yes | O No |
| 4 Does your child experience intense ear pain and pain behind the ear? | O Yes | O No |
| 5 Does your child experience swelling/bulging, redness, tenderness, or dropping behind or of the ear(s) | O Yes | O No |
| 6 Does your child experience facial asymmetry (e.g. when the child smiles, cries)? | O Yes | O No |

**7 Side effects**

Does your child have these complaints after taking the medicine

| | | | | | |
|---|---|---|---|---|---|
| 7.1 Increased appetite | O Yes | O No | 7.8 Drowsiness | O Yes | O No |
| 7.2 Increased urine amount | O Yes | O No | 7.9 Anxiety/distractibility/mood swing | O Yes | O No |
| 7.3 Weight gain | O Yes | O No | 7.10 Headache | O Yes | O No |
| 7.4 Gastritis/abdominal pain | O Yes | O No | 7.11 Skin rash or diaper rash | O Yes | O No |
| 7.5 Nausea | O Yes | O No | 7.12 Candidiasis | O Yes | O No |
| 7.6 Vomiting | O Yes | O No | 7.13 Dry mouth / throat irritation | O Yes | O No |
| 7.7 Diarrhea | O Yes | O No | 7.14 Sleep disturbance | O Yes | O No |
| Others | ______ | | | | |

| | | | | |
|---|---|---|---|---|
| Did you bring your child to doctor (clinic or outpatient)? | O Yes | O No | Reason: | ______ |
| | | | Medicine prescribed: | ______ |
| Has your child has been admitted to hospital? | O Yes | O No | Reason: | ______ |
| | | | Medicine prescribed: | ______ |

*For each question, please tick (✓) your answer on O or write you answer on ______*

**Registration ID** ____________

| Medicines given (please write the name, dose, and frequency) | | | |
|---|---|---|---|
| Additional medicine from the chemist store or other (not precribed by your doctor) | ______________________ | Dose : _____ mg / body weight kg | Frequency : ______ / day |
| | ______________________ | Dose : _____ mg / body weight kg | Frequency : ______ / day |
| | ______________________ | Dose : _____ mg / body weight kg | Frequency : ______ / day |
| | ______________________ | Dose : _____ mg / body weight kg | Frequency : ______ / day |
| | ______________________ | Dose : _____ mg / body weight kg | Frequency : ______ / day |

| Please list all medicines you give to your child today by marking the circle based on the frequency and the time | | | | | |
|---|---|---|---|---|---|
| ______________________ | O ____ am/pm | O ____ am/pm | O ____ am/pm | O ____ am/pm | O ____ am/pm |
| ______________________ | O ____ am/pm | O ____ am/pm | O ____ am/pm | O ____ am/pm | O ____ am/pm |
| ______________________ | O ____ am/pm | O ____ am/pm | O ____ am/pm | O ____ am/pm | O ____ am/pm |
| ______________________ | O ____ am/pm | O ____ am/pm | O ____ am/pm | O ____ am/pm | O ____ am/pm |
| ______________________ | O ____ am/pm | O ____ am/pm | O ____ am/pm | O ____ am/pm | O ____ am/pm |
| ______________________ | O ____ am/pm | O ____ am/pm | O ____ am/pm | O ____ am/pm | O ____ am/pm |
| ______________________ | O ____ am/pm | O ____ am/pm | O ____ am/pm | O ____ am/pm | O ____ am/pm |

**Notes:**

**Registration ID** 

# Day – 7 (2nd Follow-up Visit): |___|___| – |___|___| – 20 |___|___|

**1. Please place a vertical line across the available horizontal line that best describes your or your child's pain during the past 12 hours? Please write the time accordingly ……. (am/ pm)**

No Pain —————————————— Pain As Bad As It Could Possibly Be

**2. We are interest finding out how your child has been doing. For each question, please place a check mark in O corresponding to your child's symptoms. Please answer all questions. Please write the time accordingly ……. (am/ pm)**

| | | | |
|---|---|---|---|
| 2.1 Over the past 12 h, has your child been tugging, rubbing, or holding the ear(s) more than usual? | O No | O A little | O A lot |
| 2.2 Over the past 12 h, has your child been crying more than usual? | O No | O A little | O A lot |
| 2.3 Over the past 12 h, has your child been more irritable or fussy than usual? | O No | O A little | O A lot |
| 2.4 Over the past 12 h, has your child been having more difficulty sleeping than usual? | O No | O A little | O A lot |
| 2.5 Over the past 12 h, has your child been less playful or active than usual? | O No | O A little | O A lot |
| 2.6 Over the past 12 h, has your child been eating less than usual? | O No | O A little | O A lot |
| 2.7 Over the past 12 h, has your child been having fever or feeling warm to touch? | O No | O A little | O A lot |

**Other symptoms**

| | | |
|---|---|---|
| 3 Does your child experience discharge from the ear(s)? | O Yes | O No |
| 4 Does your child experience intense ear pain and pain behind the ear? | O Yes | O No |
| 5 Does your child experience swelling/bulging, redness, tenderness, or dropping behind or of the ear(s) | O Yes | O No |
| 6 Does your child experience facial asymmetry (e.g. when the child smiles, cries)? | O Yes | O No |

**7 Side effects**

Does your child have these complaints after taking the medicine

| | | | | | |
|---|---|---|---|---|---|
| 7.1 Increased appetite | O Yes | O No | 7.8 Drowsiness | O Yes | O No |
| 7.2 Increased urine amount | O Yes | O No | 7.9 Anxiety/distractibility/mood swing | O Yes | O No |
| 7.3 Weight gain | O Yes | O No | 7.10 Headache | O Yes | O No |
| 7.4 Gastritis/abdominal pain | O Yes | O No | 7.11 Skin rash or diaper rash | O Yes | O No |
| 7.5 Nausea | O Yes | O No | 7.12 Candidiasis | O Yes | O No |
| 7.6 Vomiting | O Yes | O No | 7.13 Dry mouth / throat irritation | O Yes | O No |
| 7.7 Diarrhea | O Yes | O No | 7.14 Sleep disturbance | O Yes | O No |
| Others | ______ | | | | |

| | | | | |
|---|---|---|---|---|
| Did you bring your child to doctor (clinic or outpatient)? | O Yes | O No | Reason: | ______ |
| | | | Medicine prescribed: | ______ |
| Has your child has been admitted to hospital? | O Yes | O No | Reason: | ______ |
| | | | Medicine prescribed: | ______ |

*For each question, please tick (✓) your answer on O or write you answer on ______*

**Registration ID**

| **Medicines given (please write the name, dose, and frequency)** | | | |
|---|---|---|---|
| Additional medicine from the chemist store or other (not precribed by your doctor) | ______________ | Dose : _____ mg / body weight kg | Frequency : ______ / day |
| | ______________ | Dose : _____ mg / body weight kg | Frequency : ______ / day |
| | ______________ | Dose : _____ mg / body weight kg | Frequency : ______ / day |
| | ______________ | Dose : _____ mg / body weight kg | Frequency : ______ / day |
| | ______________ | Dose : _____ mg / body weight kg | Frequency : ______ / day |

| **Please list all medicines you give to your child today by marking the circle based on the frequency and the time** | | | | | |
|---|---|---|---|---|---|
| ______________ | O ____ am/pm | O ____ am/pm | O ____ am/pm | O ____ am/pm | O ____ am/pm |
| ______________ | O ____ am/pm | O ____ am/pm | O ____ am/pm | O ____ am/pm | O ____ am/pm |
| ______________ | O ____ am/pm | O ____ am/pm | O ____ am/pm | O ____ am/pm | O ____ am/pm |
| ______________ | O ____ am/pm | O ____ am/pm | O ____ am/pm | O ____ am/pm | O ____ am/pm |
| ______________ | O ____ am/pm | O ____ am/pm | O ____ am/pm | O ____ am/pm | O ____ am/pm |
| ______________ | O ____ am/pm | O ____ am/pm | O ____ am/pm | O ____ am/pm | O ____ am/pm |
| ______________ | O ____ am/pm | O ____ am/pm | O ____ am/pm | O ____ am/pm | O ____ am/pm |

**Notes:**

*Thank you for completing the first Diary.*

Registration ID

# Additional Visit : |___|___| – |___|___| – 20 |___|___|

**1. Please place a vertical line across the available horizontal line that best describes your or your child's pain during the past 12 hours? Please write the time accordingly ……. (am/ pm)**

No Pain |————————————| Pain As Bad As It Could Possibly Be

**2. We are interest finding out how your child has been doing. For each question, please place a check mark in O corresponding to your child's symptoms. Please answer all questions. Please write the time accordingly ……. (am/ pm)**

| | | | |
|---|---|---|---|
| 2.1 Over the past 12 h, has your child been tugging, rubbing, or holding the ear(s) more than usual? | O No | O A little | O A lot |
| 2.2 Over the past 12 h, has your child been crying more than usual? | O No | O A little | O A lot |
| 2.3 Over the past 12 h, has your child been more irritable or fussy than usual? | O No | O A little | O A lot |
| 2.4 Over the past 12 h, has your child been having more difficulty sleeping than usual? | O No | O A little | O A lot |
| 2.5 Over the past 12 h, has your child been less playful or active than usual? | O No | O A little | O A lot |
| 2.6 Over the past 12 h, has your child been eating less than usual? | O No | O A little | O A lot |
| 2.7 Over the past 12 h, has your child been having fever or feeling warm to touch? | O No | O A little | O A lot |

**Other symptoms**

| | | |
|---|---|---|
| 3 Does your child experience discharge from the ear(s)? | O Yes | O No |
| 4 Does your child experience intense ear pain and pain behind the ear? | O Yes | O No |
| 5 Does your child experience swelling/bulging, redness, tenderness, or dropping behind or of the ear(s) | O Yes | O No |
| 6 Does your child experience facial asymmetry (e.g. when the child smiles, cries)? | O Yes | O No |

**7 Side effects**

Does your child have these complaints after taking the medicine

| | | | | | |
|---|---|---|---|---|---|
| 7.1 Increased appetite | O Yes | O No | 7.8 Drowsiness | O Yes | O No |
| 7.2 Increased urine amount | O Yes | O No | 7.9 Anxiety/distractibility/mood swing | O Yes | O No |
| 7.3 Weight gain | O Yes | O No | 7.10 Headache | O Yes | O No |
| 7.4 Gastritis/abdominal pain | O Yes | O No | 7.11 Skin rash or diaper rash | O Yes | O No |
| 7.5 Nausea | O Yes | O No | 7.12 Candidiasis | O Yes | O No |
| 7.6 Vomiting | O Yes | O No | 7.13 Dry mouth / throat irritation | O Yes | O No |
| 7.7 Diarrhea | O Yes | O No | 7.14 Sleep disturbance | O Yes | O No |
| Others | ______ | | | | |

| | | | | |
|---|---|---|---|---|
| Did you bring your child to doctor (clinic or outpatient)? | O Yes | O No | Reason: | ______ |
| | | | Medicine prescribed: | ______ |
| Has your child has been admitted to hospital? | O Yes | O No | Reason: | ______ |
| | | | Medicine prescribed: | ______ |

*For each question, please tick (✓) your answer on O or write you answer on ______*

**Registration ID**

| **Medicines given (please write the name, dose, and frequency)** | | | |
|---|---|---|---|
| Additional medicine from the chemist store or other (not precribed by your doctor) | ________________ | Dose : _____ mg / body weight kg | Frequency : ______ / day |
| | ________________ | Dose : _____ mg / body weight kg | Frequency : ______ / day |
| | ________________ | Dose : _____ mg / body weight kg | Frequency : ______ / day |
| | ________________ | Dose : _____ mg / body weight kg | Frequency : ______ / day |
| | ________________ | Dose : _____ mg / body weight kg | Frequency : ______ / day |

| **Please list all medicines you give to your child today by marking the circle based on the frequency and the time** | | | | | |
|---|---|---|---|---|---|
| ________________ | O ____ am/pm | O ____ am/pm | O ____ am/pm | O ____ am/pm | O ____ am/pm |
| ________________ | O ____ am/pm | O ____ am/pm | O ____ am/pm | O ____ am/pm | O ____ am/pm |
| ________________ | O ____ am/pm | O ____ am/pm | O ____ am/pm | O ____ am/pm | O ____ am/pm |
| ________________ | O ____ am/pm | O ____ am/pm | O ____ am/pm | O ____ am/pm | O ____ am/pm |
| ________________ | O ____ am/pm | O ____ am/pm | O ____ am/pm | O ____ am/pm | O ____ am/pm |
| ________________ | O ____ am/pm | O ____ am/pm | O ____ am/pm | O ____ am/pm | O ____ am/pm |
| ________________ | O ____ am/pm | O ____ am/pm | O ____ am/pm | O ____ am/pm | O ____ am/pm |

**Notes:**

*Thank you for completing the second Diary.*

# DIARY-3 (Day-8 to Day-14)

OPAL Study

Oral Prednisolone for Acute otitis media in children

Registration ID

**Registration ID** | |

Hello Uncle / Aunty!!

My name is_______________________

I was born in_________________

On date _____ month ________ year ___________

If you find this Diary, I would be very grateful if you can return it to my Dad (mobile no. _______________) or my Mom (mobile no. _______________).

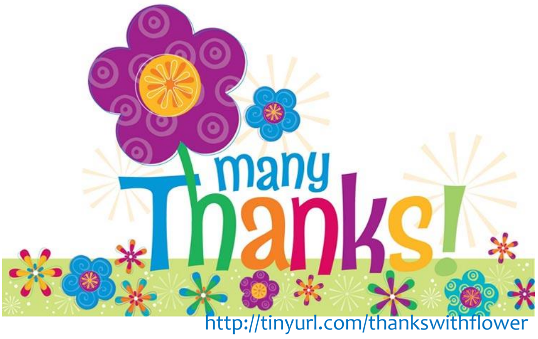


http://tinyurl.com/thankswithflower

**Registration ID** | |

## Day – 8 : |___|___| – |___|___| – 20 |___|___|

**1. Please place a vertical line across the available horizontal line that best describes your or your child's pain during the past 12 hours? Please write the time accordingly ……. (am/ pm)**

No Pain |——————————————————| Pain As Bad As It Could Possibly Be

**2. We are interest finding out how your child has been doing. For each question, please place a check mark in O corresponding to your child's symptoms. Please answer all questions. Please write the time accordingly ……. (am/ pm)**

| | | | |
|---|---|---|---|
| 2.1 Over the past 12 h, has your child been tugging, rubbing, or holding the ear(s) more than usual? | O No | O A little | O A lot |
| 2.2 Over the past 12 h, has your child been crying more than usual? | O No | O A little | O A lot |
| 2.3 Over the past 12 h, has your child been more irritable or fussy than usual? | O No | O A little | O A lot |
| 2.4 Over the past 12 h, has your child been having more difficulty sleeping than usual? | O No | O A little | O A lot |
| 2.5 Over the past 12 h, has your child been less playful or active than usual? | O No | O A little | O A lot |
| 2.6 Over the past 12 h, has your child been eating less than usual? | O No | O A little | O A lot |
| 2.7 Over the past 12 h, has your child been having fever or feeling warm to touch? | O No | O A little | O A lot |

**Other symptoms**

| | | |
|---|---|---|
| 3 Does your child experience discharge from the ear(s)? | O Yes | O No |
| 4 Does your child experience intense ear pain and pain behind the ear? | O Yes | O No |
| 5 Does your child experience swelling/bulging, redness, tenderness, or dropping behind or of the ear(s) | O Yes | O No |
| 6 Does your child experience facial asymmetry (e.g. when the child smiles, cries)? | O Yes | O No |

**7 Side effects**

Does your child have these complaints after taking the medicine

| | | | | | |
|---|---|---|---|---|---|
| 7.1 Increased appetite | O Yes | O No | 7.8 Drowsiness | O Yes | O No |
| 7.2 Increased urine amount | O Yes | O No | 7.9 Anxiety/distractibility/mood swing | O Yes | O No |
| 7.3 Weight gain | O Yes | O No | 7.10 Headache | O Yes | O No |
| 7.4 Gastritis/abdominal pain | O Yes | O No | 7.11 Skin rash or diaper rash | O Yes | O No |
| 7.5 Nausea | O Yes | O No | 7.12 Candidiasis | O Yes | O No |
| 7.6 Vomiting | O Yes | O No | 7.13 Dry mouth / throat irritation | O Yes | O No |
| 7.7 Diarrhea | O Yes | O No | 7.14 Sleep disturbance | O Yes | O No |

| | | | | |
|---|---|---|---|---|
| Others | ______________________________ | | | |
| Did you bring your child to doctor (clinic or outpatient)? | O Yes | O No | Reason: | ____________ |
| | | | Medicine prescribed: | ____________ |
| Has your child has been admitted to hospital? | O Yes | O No | Reason: | ____________ |
| | | | Medicine prescribed: | ____________ |

*For each question, please tick (✓) your answer on O or write you answer on ______*

| **Medicines given (please write the name, dose, and frequency)** | |
|---|---|
| Additional medicine from the chemist store or other (not precribed by your doctor) | ________________ Dose : _____ mg / body weight kg Frequency : ______ / day |
| | ________________ Dose : _____ mg / body weight kg Frequency : ______ / day |
| | ________________ Dose : _____ mg / body weight kg Frequency : ______ / day |
| | ________________ Dose : _____ mg / body weight kg Frequency : ______ / day |
| | ________________ Dose : _____ mg / body weight kg Frequency : ______ / day |

| **Please list all medicines you give to your child today by marking the circle based on the frequency and the time** | | | | | |
|---|---|---|---|---|---|
| ________________ | O ____ am/pm | O ____ am/pm | O ____ am/pm | O ____ am/pm | O ____ am/pm |
| ________________ | O ____ am/pm | O ____ am/pm | O ____ am/pm | O ____ am/pm | O ____ am/pm |
| ________________ | O ____ am/pm | O ____ am/pm | O ____ am/pm | O ____ am/pm | O ____ am/pm |
| ________________ | O ____ am/pm | O ____ am/pm | O ____ am/pm | O ____ am/pm | O ____ am/pm |
| ________________ | O ____ am/pm | O ____ am/pm | O ____ am/pm | O ____ am/pm | O ____ am/pm |
| ________________ | O ____ am/pm | O ____ am/pm | O ____ am/pm | O ____ am/pm | O ____ am/pm |
| ________________ | O ____ am/pm | O ____ am/pm | O ____ am/pm | O ____ am/pm | O ____ am/pm |

**Notes:**

**Registration ID** | |

# Day – 9 : |___|___| – |___|___| – 20 |___|___|

**1. Please place a vertical line across the available horizontal line that best describes your or your child's pain during the past 12 hours? Please write the time accordingly ……. (am/ pm)**

No Pain |——————————————————| Pain As Bad As It Could Possibly Be

**2. We are interest finding out how your child has been doing. For each question, please place a check mark in O corresponding to your child's symptoms. Please answer all questions. Please write the time accordingly ……. (am/ pm)**

| | | | |
|---|---|---|---|
| 2.1 Over the past 12 h, has your child been tugging, rubbing, or holding the ear(s) more than usual? | O No | O A little | O A lot |
| 2.2 Over the past 12 h, has your child been crying more than usual? | O No | O A little | O A lot |
| 2.3 Over the past 12 h, has your child been more irritable or fussy than usual? | O No | O A little | O A lot |
| 2.4 Over the past 12 h, has your child been having more difficulty sleeping than usual? | O No | O A little | O A lot |
| 2.5 Over the past 12 h, has your child been less playful or active than usual? | O No | O A little | O A lot |
| 2.6 Over the past 12 h, has your child been eating less than usual? | O No | O A little | O A lot |
| 2.7 Over the past 12 h, has your child been having fever or feeling warm to touch? | O No | O A little | O A lot |

**Other symptoms**

| | | |
|---|---|---|
| 3 Does your child experience discharge from the ear(s)? | O Yes | O No |
| 4 Does your child experience intense ear pain and pain behind the ear? | O Yes | O No |
| 5 Does your child experience swelling/bulging, redness, tenderness, or dropping behind or of the ear(s) | O Yes | O No |
| 6 Does your child experience facial asymmetry (e.g. when the child smiles, cries)? | O Yes | O No |

**7 Side effects**

Does your child have these complaints after taking the medicine

| | | | | | |
|---|---|---|---|---|---|
| 7.1 Increased appetite | O Yes | O No | 7.8 Drowsiness | O Yes | O No |
| 7.2 Increased urine amount | O Yes | O No | 7.9 Anxiety/distractibility/mood swing | O Yes | O No |
| 7.3 Weight gain | O Yes | O No | 7.10 Headache | O Yes | O No |
| 7.4 Gastritis/abdominal pain | O Yes | O No | 7.11 Skin rash or diaper rash | O Yes | O No |
| 7.5 Nausea | O Yes | O No | 7.12 Candidiasis | O Yes | O No |
| 7.6 Vomiting | O Yes | O No | 7.13 Dry mouth / throat irritation | O Yes | O No |
| 7.7 Diarrhea | O Yes | O No | 7.14 Sleep disturbance | O Yes | O No |
| Others | ____________________ | | | | |

| | | | | |
|---|---|---|---|---|
| Did you bring your child to doctor (clinic or outpatient)? | O Yes | O No | Reason: | ____________ |
| | | | Medicine prescribed: | ____________ |
| Has your child has been admitted to hospital? | O Yes | O No | Reason: | ____________ |
| | | | Medicine prescribed: | ____________ |

*For each question, please tick (✓) your answer on O or write you answer on ______*

| **Medicines given (please write the name, dose, and frequency)** | | | |
|---|---|---|---|
| Additional medicine from the chemist store or other (not precribed by your doctor) | ______________ | Dose : _____ mg / body weight kg | Frequency : ______ / day |
| | ______________ | Dose : _____ mg / body weight kg | Frequency : ______ / day |
| | ______________ | Dose : _____ mg / body weight kg | Frequency : ______ / day |
| | ______________ | Dose : _____ mg / body weight kg | Frequency : ______ / day |
| | ______________ | Dose : _____ mg / body weight kg | Frequency : ______ / day |

| **Please list all medicines you give to your child today by marking the circle based on the frequency and the time** | | | | | |
|---|---|---|---|---|---|
| ______________ | O ____ am/pm | O ____ am/pm | O ____ am/pm | O ____ am/pm | O ____ am/pm |
| ______________ | O ____ am/pm | O ____ am/pm | O ____ am/pm | O ____ am/pm | O ____ am/pm |
| ______________ | O ____ am/pm | O ____ am/pm | O ____ am/pm | O ____ am/pm | O ____ am/pm |
| ______________ | O ____ am/pm | O ____ am/pm | O ____ am/pm | O ____ am/pm | O ____ am/pm |
| ______________ | O ____ am/pm | O ____ am/pm | O ____ am/pm | O ____ am/pm | O ____ am/pm |
| ______________ | O ____ am/pm | O ____ am/pm | O ____ am/pm | O ____ am/pm | O ____ am/pm |
| ______________ | O ____ am/pm | O ____ am/pm | O ____ am/pm | O ____ am/pm | O ____ am/pm |

**Notes:**

Registration ID

# Day – 9 : |___|___| – |___|___| – 20 |___|___|

**1. Please place a vertical line across the available horizontal line that best describes your or your child's pain during the past 12 hours? Please write the time accordingly ……. (am/ pm)**

No Pain |———————————————| Pain As Bad As It Could Possibly Be

**2. We are interest finding out how your child has been doing. For each question, please place a check mark in O corresponding to your child's symptoms. Please answer all questions. Please write the time accordingly ……. (am/ pm)**

| | | | |
|---|---|---|---|
| 2.1 Over the past 12 h, has your child been tugging, rubbing, or holding the ear(s) more than usual? | O No | O A little | O A lot |
| 2.2 Over the past 12 h, has your child been crying more than usual? | O No | O A little | O A lot |
| 2.3 Over the past 12 h, has your child been more irritable or fussy than usual? | O No | O A little | O A lot |
| 2.4 Over the past 12 h, has your child been having more difficulty sleeping than usual? | O No | O A little | O A lot |
| 2.5 Over the past 12 h, has your child been less playful or active than usual? | O No | O A little | O A lot |
| 2.6 Over the past 12 h, has your child been eating less than usual? | O No | O A little | O A lot |
| 2.7 Over the past 12 h, has your child been having fever or feeling warm to touch? | O No | O A little | O A lot |

**Other symptoms**

| | | |
|---|---|---|
| 3 Does your child experience discharge from the ear(s)? | O Yes | O No |
| 4 Does your child experience intense ear pain and pain behind the ear? | O Yes | O No |
| 5 Does your child experience swelling/bulging, redness, tenderness, or dropping behind or of the ear(s) | O Yes | O No |
| 6 Does your child experience facial asymmetry (e.g. when the child smiles, cries)? | O Yes | O No |

**7 Side effects**

Does your child have these complaints after taking the medicine

| | | | | | |
|---|---|---|---|---|---|
| 7.1 Increased appetite | O Yes | O No | 7.8 Drowsiness | O Yes | O No |
| 7.2 Increased urine amount | O Yes | O No | 7.9 Anxiety/distractibility/mood swing | O Yes | O No |
| 7.3 Weight gain | O Yes | O No | 7.10 Headache | O Yes | O No |
| 7.4 Gastritis/abdominal pain | O Yes | O No | 7.11 Skin rash or diaper rash | O Yes | O No |
| 7.5 Nausea | O Yes | O No | 7.12 Candidiasis | O Yes | O No |
| 7.6 Vomiting | O Yes | O No | 7.13 Dry mouth / throat irritation | O Yes | O No |
| 7.7 Diarrhea | O Yes | O No | 7.14 Sleep disturbance | O Yes | O No |
| Others | ______________________ | | | | |

| | | | | |
|---|---|---|---|---|
| Did you bring your child to doctor (clinic or outpatient)? | O Yes | O No | Reason: | ______________ |
| | | | Medicine prescribed: | ______________ |
| Has your child has been admitted to hospital? | O Yes | O No | Reason: | ______________ |
| | | | Medicine prescribed: | ______________ |

*For each question, please tick (✓) your answer on O or write you answer on ______*

**Registration ID** [ ]

| **Medicines given (please write the name, dose, and frequency)** | | | |
|---|---|---|---|
| Additional medicine from the chemist store or other (not precribed by your doctor) | ______________ | Dose : _____ mg / body weight kg | Frequency : ______ / day |
| | ______________ | Dose : _____ mg / body weight kg | Frequency : ______ / day |
| | ______________ | Dose : _____ mg / body weight kg | Frequency : ______ / day |
| | ______________ | Dose : _____ mg / body weight kg | Frequency : ______ / day |
| | ______________ | Dose : _____ mg / body weight kg | Frequency : ______ / day |

| **Please list all medicines you give to your child today by marking the circle based on the frequency and the time** | | | | | |
|---|---|---|---|---|---|
| ______________ | O ____ am/pm | O ____ am/pm | O ____ am/pm | O ____ am/pm | O ____ am/pm |
| ______________ | O ____ am/pm | O ____ am/pm | O ____ am/pm | O ____ am/pm | O ____ am/pm |
| ______________ | O ____ am/pm | O ____ am/pm | O ____ am/pm | O ____ am/pm | O ____ am/pm |
| ______________ | O ____ am/pm | O ____ am/pm | O ____ am/pm | O ____ am/pm | O ____ am/pm |
| ______________ | O ____ am/pm | O ____ am/pm | O ____ am/pm | O ____ am/pm | O ____ am/pm |
| ______________ | O ____ am/pm | O ____ am/pm | O ____ am/pm | O ____ am/pm | O ____ am/pm |
| ______________ | O ____ am/pm | O ____ am/pm | O ____ am/pm | O ____ am/pm | O ____ am/pm |

**Notes:**

# Day – 11 : |___|___| – |___|___| – 20 |___|___|

**1. Please place a vertical line across the available horizontal line that best describes your or your child's pain during the past 12 hours? Please write the time accordingly ……. (am/ pm)**

No Pain ———————————————— Pain As Bad As It Could Possibly Be

**2. We are interest finding out how your child has been doing. For each question, please place a check mark in O corresponding to your child's symptoms. Please answer all questions. Please write the time accordingly ……. (am/ pm)**

| | | | |
|---|---|---|---|
| 2.1 Over the past 12 h, has your child been tugging, rubbing, or holding the ear(s) more than usual? | O No | O A little | O A lot |
| 2.2 Over the past 12 h, has your child been crying more than usual? | O No | O A little | O A lot |
| 2.3 Over the past 12 h, has your child been more irritable or fussy than usual? | O No | O A little | O A lot |
| 2.4 Over the past 12 h, has your child been having more difficulty sleeping than usual? | O No | O A little | O A lot |
| 2.5 Over the past 12 h, has your child been less playful or active than usual? | O No | O A little | O A lot |
| 2.6 Over the past 12 h, has your child been eating less than usual? | O No | O A little | O A lot |
| 2.7 Over the past 12 h, has your child been having fever or feeling warm to touch? | O No | O A little | O A lot |

**Other symptoms**

| | | |
|---|---|---|
| 3 Does your child experience discharge from the ear(s)? | O Yes | O No |
| 4 Does your child experience intense ear pain and pain behind the ear? | O Yes | O No |
| 5 Does your child experience swelling/bulging, redness, tenderness, or dropping behind or of the ear(s) | O Yes | O No |
| 6 Does your child experience facial asymmetry (e.g. when the child smiles, cries)? | O Yes | O No |

**7 Side effects**

Does your child have these complaints after taking the medicine

| | | | | | |
|---|---|---|---|---|---|
| 7.1 Increased appetite | O Yes | O No | 7.8 Drowsiness | O Yes | O No |
| 7.2 Increased urine amount | O Yes | O No | 7.9 Anxiety/distractibility/mood swing | O Yes | O No |
| 7.3 Weight gain | O Yes | O No | 7.10 Headache | O Yes | O No |
| 7.4 Gastritis/abdominal pain | O Yes | O No | 7.11 Skin rash or diaper rash | O Yes | O No |
| 7.5 Nausea | O Yes | O No | 7.12 Candidiasis | O Yes | O No |
| 7.6 Vomiting | O Yes | O No | 7.13 Dry mouth / throat irritation | O Yes | O No |
| 7.7 Diarrhea | O Yes | O No | 7.14 Sleep disturbance | O Yes | O No |
| Others | ______ | | | | |
| Did you bring your child to doctor (clinic or outpatient)? | O Yes | O No | Reason: | ______ | |
| | | | Medicine prescribed: | ______ | |
| Has your child has been admitted to hospital? | O Yes | O No | Reason: | ______ | |
| | | | Medicine prescribed: | ______ | |

*For each question, please tick (✓) your answer on O or write you answer on ______*

**Registration ID** 

| **Medicines given (please write the name, dose, and frequency)** | | | |
|---|---|---|---|
| Additional medicine from the chemist store or other (not precribed by your doctor) | ______________ | Dose : _____ mg / body weight kg | Frequency : ______ / day |
| | ______________ | Dose : _____ mg / body weight kg | Frequency : ______ / day |
| | ______________ | Dose : _____ mg / body weight kg | Frequency : ______ / day |
| | ______________ | Dose : _____ mg / body weight kg | Frequency : ______ / day |
| | ______________ | Dose : _____ mg / body weight kg | Frequency : ______ / day |

| **Please list all medicines you give to your child today by marking the circle based on the frequency and the time** | | | | | |
|---|---|---|---|---|---|
| ______________ | O ____ am/pm | O ____ am/pm | O ____ am/pm | O ____ am/pm | O ____ am/pm |
| ______________ | O ____ am/pm | O ____ am/pm | O ____ am/pm | O ____ am/pm | O ____ am/pm |
| ______________ | O ____ am/pm | O ____ am/pm | O ____ am/pm | O ____ am/pm | O ____ am/pm |
| ______________ | O ____ am/pm | O ____ am/pm | O ____ am/pm | O ____ am/pm | O ____ am/pm |
| ______________ | O ____ am/pm | O ____ am/pm | O ____ am/pm | O ____ am/pm | O ____ am/pm |
| ______________ | O ____ am/pm | O ____ am/pm | O ____ am/pm | O ____ am/pm | O ____ am/pm |
| ______________ | O ____ am/pm | O ____ am/pm | O ____ am/pm | O ____ am/pm | O ____ am/pm |

**Notes:**

**Registration ID** | |

# Day – 12 : |___|___| – |___|___| – 20 |___|___|

**1. Please place a vertical line across the available horizontal line that best describes your or your child's pain during the past 12 hours? Please write the time accordingly ……. (am/ pm)**

No Pain ———————————————— Pain As Bad As It Could Possibly Be

**2. We are interest finding out how your child has been doing. For each question, please place a check mark in O corresponding to your child's symptoms. Please answer all questions. Please write the time accordingly ……. (am/ pm)**

| | | | |
|---|---|---|---|
| 2.1 Over the past 12 h, has your child been tugging, rubbing, or holding the ear(s) more than usual? | O No | O A little | O A lot |
| 2.2 Over the past 12 h, has your child been crying more than usual? | O No | O A little | O A lot |
| 2.3 Over the past 12 h, has your child been more irritable or fussy than usual? | O No | O A little | O A lot |
| 2.4 Over the past 12 h, has your child been having more difficulty sleeping than usual? | O No | O A little | O A lot |
| 2.5 Over the past 12 h, has your child been less playful or active than usual? | O No | O A little | O A lot |
| 2.6 Over the past 12 h, has your child been eating less than usual? | O No | O A little | O A lot |
| 2.7 Over the past 12 h, has your child been having fever or feeling warm to touch? | O No | O A little | O A lot |

**Other symptoms**

| | | |
|---|---|---|
| 3 Does your child experience discharge from the ear(s)? | O Yes | O No |
| 4 Does your child experience intense ear pain and pain behind the ear? | O Yes | O No |
| 5 Does your child experience swelling/bulging, redness, tenderness, or dropping behind or of the ear(s) | O Yes | O No |
| 6 Does your child experience facial asymmetry (e.g. when the child smiles, cries)? | O Yes | O No |

**7 Side effects**

Does your child have these complaints after taking the medicine

| | | | | | |
|---|---|---|---|---|---|
| 7.1 Increased appetite | O Yes | O No | 7.8 Drowsiness | O Yes | O No |
| 7.2 Increased urine amount | O Yes | O No | 7.9 Anxiety/distractibility/mood swing | O Yes | O No |
| 7.3 Weight gain | O Yes | O No | 7.10 Headache | O Yes | O No |
| 7.4 Gastritis/abdominal pain | O Yes | O No | 7.11 Skin rash or diaper rash | O Yes | O No |
| 7.5 Nausea | O Yes | O No | 7.12 Candidiasis | O Yes | O No |
| 7.6 Vomiting | O Yes | O No | 7.13 Dry mouth / throat irritation | O Yes | O No |
| 7.7 Diarrhea | O Yes | O No | 7.14 Sleep disturbance | O Yes | O No |
| Others | ____________________ | | | | |

| | | | | |
|---|---|---|---|---|
| Did you bring your child to doctor (clinic or outpatient)? | O Yes | O No | Reason: | ____________________ |
| | | | Medicine prescribed: | ____________________ |
| Has your child has been admitted to hospital? | O Yes | O No | Reason: | ____________________ |
| | | | Medicine prescribed: | ____________________ |

*For each question, please tick (✓) your answer on O or write you answer on ______*

**Registration ID** | |

| **Medicines given (please write the name, dose, and frequency)** | | | |
|---|---|---|---|
| Additional medicine from the chemist store or other (not precribed by your doctor) | ____________________ | Dose : _____ mg / body weight kg | Frequency : ______ / day |
| | ____________________ | Dose : _____ mg / body weight kg | Frequency : ______ / day |
| | ____________________ | Dose : _____ mg / body weight kg | Frequency : ______ / day |
| | ____________________ | Dose : _____ mg / body weight kg | Frequency : ______ / day |
| | ____________________ | Dose : _____ mg / body weight kg | Frequency : ______ / day |

| **Please list all medicines you give to your child today by marking the circle based on the frequency and the time** | | | | | |
|---|---|---|---|---|---|
| ____________________ | O ____ am/pm | O ____ am/pm | O ____ am/pm | O ____ am/pm | O ____ am/pm |
| ____________________ | O ____ am/pm | O ____ am/pm | O ____ am/pm | O ____ am/pm | O ____ am/pm |
| ____________________ | O ____ am/pm | O ____ am/pm | O ____ am/pm | O ____ am/pm | O ____ am/pm |
| ____________________ | O ____ am/pm | O ____ am/pm | O ____ am/pm | O ____ am/pm | O ____ am/pm |
| ____________________ | O ____ am/pm | O ____ am/pm | O ____ am/pm | O ____ am/pm | O ____ am/pm |
| ____________________ | O ____ am/pm | O ____ am/pm | O ____ am/pm | O ____ am/pm | O ____ am/pm |
| ____________________ | O ____ am/pm | O ____ am/pm | O ____ am/pm | O ____ am/pm | O ____ am/pm |

**Notes:**

**Registration ID** | |

# Day – 13 : |___|___| – |___|___| – 20 |___|___|

**1. Please place a vertical line across the available horizontal line that best describes your or your child's pain during the past 12 hours? Please write the time accordingly ……. (am/ pm)**

No Pain |————————————————| Pain As Bad As It Could Possibly Be

**2. We are interest finding out how your child has been doing. For each question, please place a check mark in O corresponding to your child's symptoms. Please answer all questions. Please write the time accordingly ……. (am/ pm)**

| | | | |
|---|---|---|---|
| 2.1 Over the past 12 h, has your child been tugging, rubbing, or holding the ear(s) more than usual? | O No | O A little | O A lot |
| 2.2 Over the past 12 h, has your child been crying more than usual? | O No | O A little | O A lot |
| 2.3 Over the past 12 h, has your child been more irritable or fussy than usual? | O No | O A little | O A lot |
| 2.4 Over the past 12 h, has your child been having more difficulty sleeping than usual? | O No | O A little | O A lot |
| 2.5 Over the past 12 h, has your child been less playful or active than usual? | O No | O A little | O A lot |
| 2.6 Over the past 12 h, has your child been eating less than usual? | O No | O A little | O A lot |
| 2.7 Over the past 12 h, has your child been having fever or feeling warm to touch? | O No | O A little | O A lot |

**Other symptoms**

| | | |
|---|---|---|
| 3 Does your child experience discharge from the ear(s)? | O Yes | O No |
| 4 Does your child experience intense ear pain and pain behind the ear? | O Yes | O No |
| 5 Does your child experience swelling/bulging, redness, tenderness, or dropping behind or of the ear(s) | O Yes | O No |
| 6 Does your child experience facial asymmetry (e.g. when the child smiles, cries)? | O Yes | O No |

**7 Side effects**

Does your child have these complaints after taking the medicine

| | | | | | |
|---|---|---|---|---|---|
| 7.1 Increased appetite | O Yes | O No | 7.8 Drowsiness | O Yes | O No |
| 7.2 Increased urine amount | O Yes | O No | 7.9 Anxiety/distractibility/mood swing | O Yes | O No |
| 7.3 Weight gain | O Yes | O No | 7.10 Headache | O Yes | O No |
| 7.4 Gastritis/abdominal pain | O Yes | O No | 7.11 Skin rash or diaper rash | O Yes | O No |
| 7.5 Nausea | O Yes | O No | 7.12 Candidiasis | O Yes | O No |
| 7.6 Vomiting | O Yes | O No | 7.13 Dry mouth / throat irritation | O Yes | O No |
| 7.7 Diarrhea | O Yes | O No | 7.14 Sleep disturbance | O Yes | O No |
| Others | __________ | | | | |

| | | | | |
|---|---|---|---|---|
| Did you bring your child to doctor (clinic or outpatient)? | O Yes | O No | Reason: | __________ |
| | | | Medicine prescribed: | __________ |
| Has your child has been admitted to hospital? | O Yes | O No | Reason: | __________ |
| | | | Medicine prescribed: | __________ |

*For each question, please tick (✓) your answer on O or write you answer on ______*

**Registration ID** | |

| **Medicines given (please write the name, dose, and frequency)** | | | |
|---|---|---|---|
| Additional medicine from the chemist store or other (not precribed by your doctor) | ____________ | Dose : _____ mg / body weight kg | Frequency : ______ / day |
| | ____________ | Dose : _____ mg / body weight kg | Frequency : ______ / day |
| | ____________ | Dose : _____ mg / body weight kg | Frequency : ______ / day |
| | ____________ | Dose : _____ mg / body weight kg | Frequency : ______ / day |
| | ____________ | Dose : _____ mg / body weight kg | Frequency : ______ / day |

| **Please list all medicines you give to your child today by marking the circle based on the frequency and the time** | | | | | |
|---|---|---|---|---|---|
| ____________ | O ____ am/pm | O ____ am/pm | O ____ am/pm | O ____ am/pm | O ____ am/pm |
| ____________ | O ____ am/pm | O ____ am/pm | O ____ am/pm | O ____ am/pm | O ____ am/pm |
| ____________ | O ____ am/pm | O ____ am/pm | O ____ am/pm | O ____ am/pm | O ____ am/pm |
| ____________ | O ____ am/pm | O ____ am/pm | O ____ am/pm | O ____ am/pm | O ____ am/pm |
| ____________ | O ____ am/pm | O ____ am/pm | O ____ am/pm | O ____ am/pm | O ____ am/pm |
| ____________ | O ____ am/pm | O ____ am/pm | O ____ am/pm | O ____ am/pm | O ____ am/pm |
| ____________ | O ____ am/pm | O ____ am/pm | O ____ am/pm | O ____ am/pm | O ____ am/pm |

**Notes:**

# Day – 14 : |___|___| – |___|___| – 20 |___|___|

**1. Please place a vertical line across the available horizontal line that best describes your or your child's pain during the past 12 hours? Please write the time accordingly ……. (am/ pm)**

No Pain |————————————————| Pain As Bad As It Could Possibly Be

**2. We are interest finding out how your child has been doing. For each question, please place a check mark in O corresponding to your child's symptoms. Please answer all questions. Please write the time accordingly ……. (am/ pm)**

| | | | |
|---|---|---|---|
| 2.1 Over the past 12 h, has your child been tugging, rubbing, or holding the ear(s) more than usual? | O No | O A little | O A lot |
| 2.2 Over the past 12 h, has your child been crying more than usual? | O No | O A little | O A lot |
| 2.3 Over the past 12 h, has your child been more irritable or fussy than usual? | O No | O A little | O A lot |
| 2.4 Over the past 12 h, has your child been having more difficulty sleeping than usual? | O No | O A little | O A lot |
| 2.5 Over the past 12 h, has your child been less playful or active than usual? | O No | O A little | O A lot |
| 2.6 Over the past 12 h, has your child been eating less than usual? | O No | O A little | O A lot |
| 2.7 Over the past 12 h, has your child been having fever or feeling warm to touch? | O No | O A little | O A lot |

**Other symptoms**

| | | |
|---|---|---|
| 3 Does your child experience discharge from the ear(s)? | O Yes | O No |
| 4 Does your child experience intense ear pain and pain behind the ear? | O Yes | O No |
| 5 Does your child experience swelling/bulging, redness, tenderness, or dropping behind or of the ear(s) | O Yes | O No |
| 6 Does your child experience facial asymmetry (e.g. when the child smiles, cries)? | O Yes | O No |

**7 Side effects**

Does your child have these complaints after taking the medicine

| | | | | | |
|---|---|---|---|---|---|
| 7.1 Increased appetite | O Yes | O No | 7.8 Drowsiness | O Yes | O No |
| 7.2 Increased urine amount | O Yes | O No | 7.9 Anxiety/distractibility/mood swing | O Yes | O No |
| 7.3 Weight gain | O Yes | O No | 7.10 Headache | O Yes | O No |
| 7.4 Gastritis/abdominal pain | O Yes | O No | 7.11 Skin rash or diaper rash | O Yes | O No |
| 7.5 Nausea | O Yes | O No | 7.12 Candidiasis | O Yes | O No |
| 7.6 Vomiting | O Yes | O No | 7.13 Dry mouth / throat irritation | O Yes | O No |
| 7.7 Diarrhea | O Yes | O No | 7.14 Sleep disturbance | O Yes | O No |

Others ______________________________

| | | | | |
|---|---|---|---|---|
| Did you bring your child to doctor (clinic or outpatient)? | O Yes | O No | Reason: | ______________ |
| | | | Medicine prescribed: | ______________ |
| Has your child has been admitted to hospital? | O Yes | O No | Reason: | ______________ |
| | | | Medicine prescribed: | ______________ |

**Registration ID** [ ]

| **Medicines given (please write the name, dose, and frequency)** | |
|---|---|
| Additional medicine from the chemist store or other (not precribed by your doctor) | ______________ Dose : _____ mg / body weight kg Frequency : ______ / day |
| | ______________ Dose : _____ mg / body weight kg Frequency : ______ / day |
| | ______________ Dose : _____ mg / body weight kg Frequency : ______ / day |
| | ______________ Dose : _____ mg / body weight kg Frequency : ______ / day |
| | ______________ Dose : _____ mg / body weight kg Frequency : ______ / day |

| **Please list all medicines you give to your child today by marking the circle based on the frequency and the time** | | | | | |
|---|---|---|---|---|---|
| ______________ | O ____ am/pm | O ____ am/pm | O ____ am/pm | O ____ am/pm | O ____ am/pm |
| ______________ | O ____ am/pm | O ____ am/pm | O ____ am/pm | O ____ am/pm | O ____ am/pm |
| ______________ | O ____ am/pm | O ____ am/pm | O ____ am/pm | O ____ am/pm | O ____ am/pm |
| ______________ | O ____ am/pm | O ____ am/pm | O ____ am/pm | O ____ am/pm | O ____ am/pm |
| ______________ | O ____ am/pm | O ____ am/pm | O ____ am/pm | O ____ am/pm | O ____ am/pm |
| ______________ | O ____ am/pm | O ____ am/pm | O ____ am/pm | O ____ am/pm | O ____ am/pm |
| ______________ | O ____ am/pm | O ____ am/pm | O ____ am/pm | O ____ am/pm | O ____ am/pm |

**Notes:**

*Thank you for completing the third Diary.*

# Lupred ® 5
## Prednisolone 5 mg
TABLET

**COMPOSITION**
Each tablet contains:
Prednisolone 5 mg

**PHARMACOLOGY**
Prednisolone is a systemic corticosteroid with glucocorticoid and anti-inflammatory potencies. The mechanism of action of corticosteroids is thought to be by control of protein synthesis. Corticosteroids react with receptor proteins in the cytoplasm of sensitive cells in many tissues to form a steroid-receptor complex.

**INDICATION**
Allergic reaction, inflammation and other diseases that require glucocorticoid treatment, such as rheumatoid arthritis, collagen diseases, and dermatology disorders.

**DOSAGE AND INSTRUCTION**
Adults: 1 – 4 tablets per day or according to the doctor's instruction.
The dosage reduces gradually until reach the lowest effective dose.

**PRECAUTION**
- Avoid the abrupt discontinuation in a long-term use
- Use with caution in paediatric patients who are still in the growing process
- Not recommended for pregnant and breast-feeding women
- Prolonged use of corticosteroids may produce posterior subcapsular cataracts, glaucoma with possible damage to the optic nerves, and may enhance the establishment of secondary ocular infections due to fungi or viruses
- Risk of secondary adrenocortical insufficiency could be reduced by gradual reduction of dosage
- Use with caution in patients with diabetes mellitus because it can increase the gluconeogenesis and reduce the sensitivity to insulin
- Use with caution in patients with hypothyroidism because it can enhance the effect of corticosteroids
- Use with caution in patients with heart failure, infection diseases, chronic renal failure, and elderly

**ADVERSE EFFECTS**
- Water balance and electrolytes disturbance: Natrium retention, excretion of potassium, hypokalaemic alkalosis, hypertension, and congestive heart failure
- Musculoskeletal: Muscle weakness, steroid-induced myopathy, osteoporosis, vertebral compression fractures and pathologic fractures of long bones
- Gastrointestinal: Peptic ulceration with haemorrhage and perforation, pancreatitis, abdominal distension and ulcerative esophagitis
- Dermatological: Impaired wound healing, thinning of the skin, facial plethora, increased sweating
- Neurological: seizures, intracranial hypertension with papilloedema (cerebral pseudotumour), vertigo, headache

FAHRENHEIT
PT. PRATAPA NIRMALA

**I001404-PG-1043**

- Endocrine: Disorders of menstruation, suppression of growth in children, secondary adrenocorticoid and non-responsive pituitary (particularly in stress, trauma, surgery or illness), metabolic effects, primarily involving the carbohydrates
- Ophthalmological: Posterior subcapsular cataracts, increased intraocular pressure, glaucoma, and exophthalmos
- Metabolic: Nitrogen depletion due to protein catabolism
- Hypersensitivity: anaphylactic reaction

**CONTRAINDICATION**

- Patients who are known hypersensitivity to prednisone or prednisolone
- Peptic ulceration, active tuberculosis, osteoporosis, neurological disorders, renal and heart disorders
- Systemic fungal infections and ocular herpes simplex

**INTERACTION WITH OTHER MEDICINES**

- The use of aspirin and corticosteroid is not recommended in patients with non-specific ulcerative colitis
- Rifampicin, phenytoin, phenobarbital can increase the metabolism of corticosteroids
- Vaccination with live vaccine must be avoided

**OVERDOSAGE**

There is no specific antidot. Treatment is symptomatic with the dosage being reduced or the drug withdrawn.

**STORAGE CONDITION**

Store below 30°C.

**DOCTOR's PRESCRIPTION IS A MUST**

Manufactured by:
**PT. PRATAPA NIRMALA**
Tangerang – Indonesia

FAHRENHEIT
PT. PRATAPA NIRMALA
