# Supplementary material for: Oral prednisolone for acute otitis media in children: protocol of a pilot randomised, open-label, controlled study (OPAL study)
Source: Pilot Feasibility Stud. 2018 Sep 10;4:146. doi: 10.1186/s40814-018-0337-x (PMC6130070; doi:10.1186/s40814-018-0337-x)
Supplement: Supplementary file 4 — Training slides—Pilot OPAL Study. The training slide was presented during the training for participating physicians, audiologists, nurses, and pharmacists. The training was conducted prior to the study commencement. This file can be accessed at https://pure.bond.edu.au/ws/portalfiles/portal/27513688/Additional_File_4._Training_Slides_Pilot_OPAL_Study.pdf (PDF 5528 kb) [file 40814_2018_337_MOESM4_ESM.pdf]

Oral Prednisolone for Acute otitis media in chiLdren: a pilot pragmatic, randomised, open-label, single-blind study (OPAL Study)

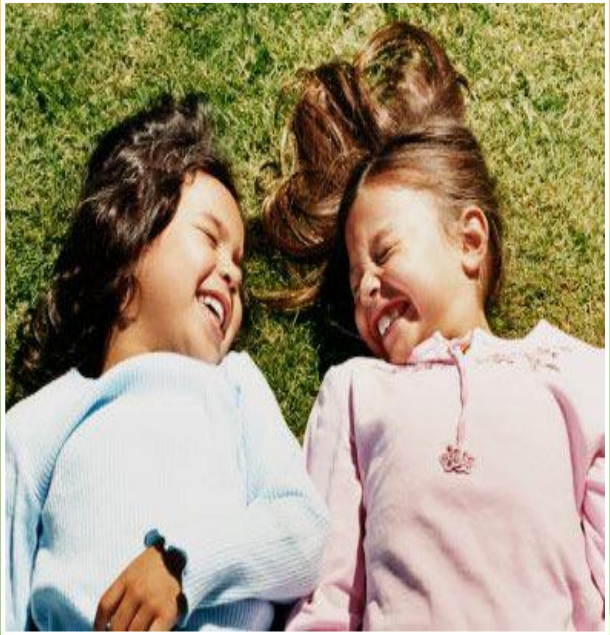

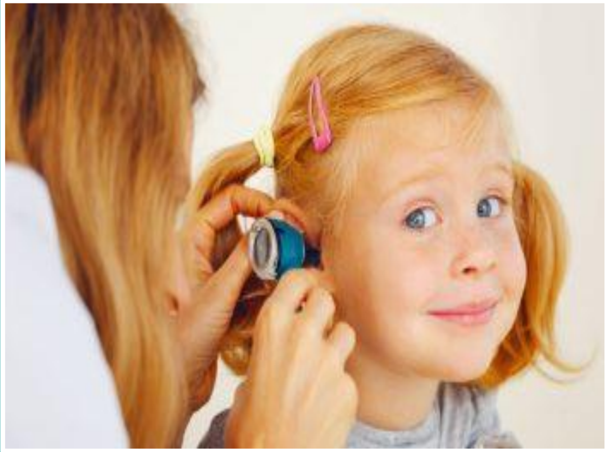

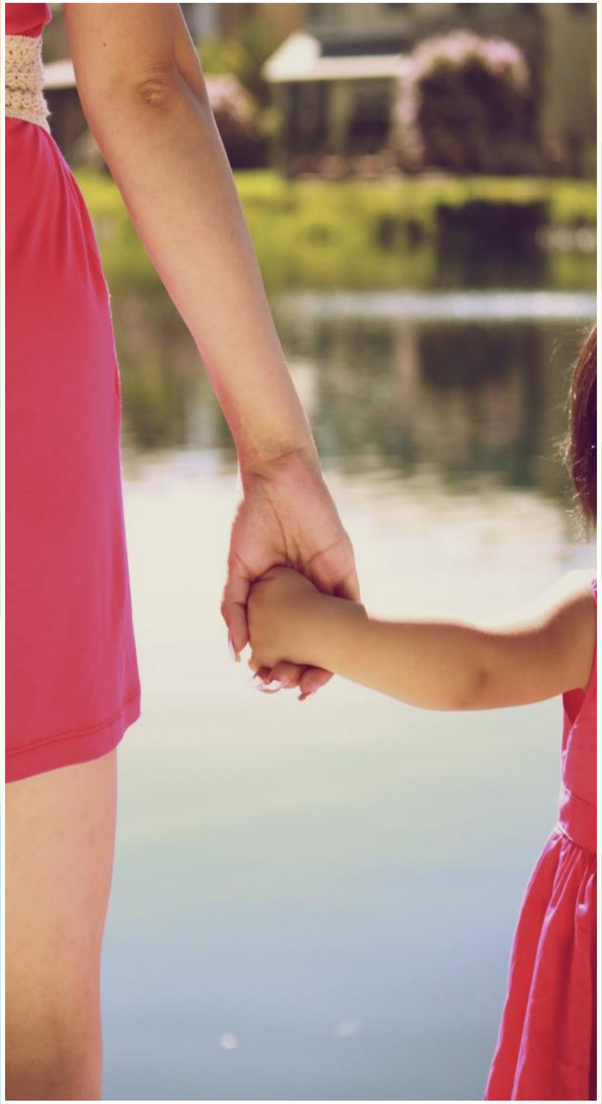

Clinical Epidemiology and Evidence-Based Medicine Unit
Dr Cipto Mangunkusumo Hospital – Faculty of Medicine Universitas Indonesia

Centre for Research in Evidence-Based Practice
Faculty of Health Sciences and Medicine Bond University

STUDY SUMMARY

1

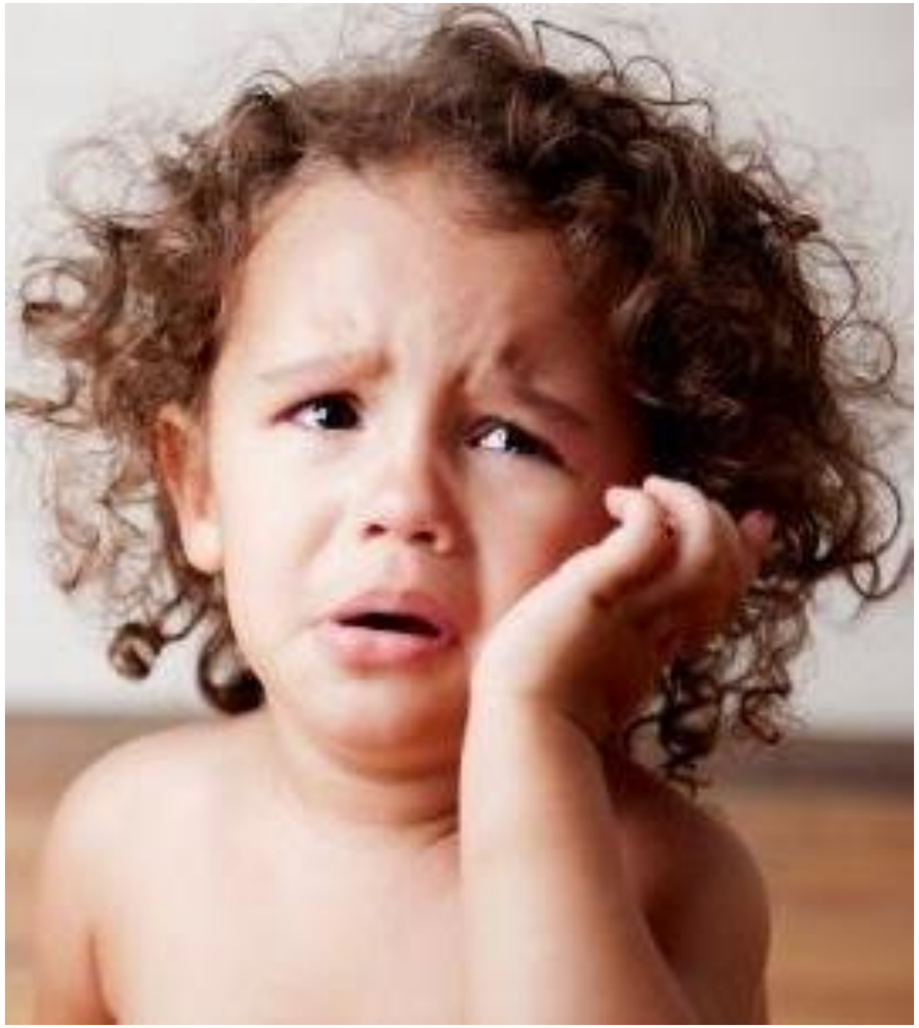

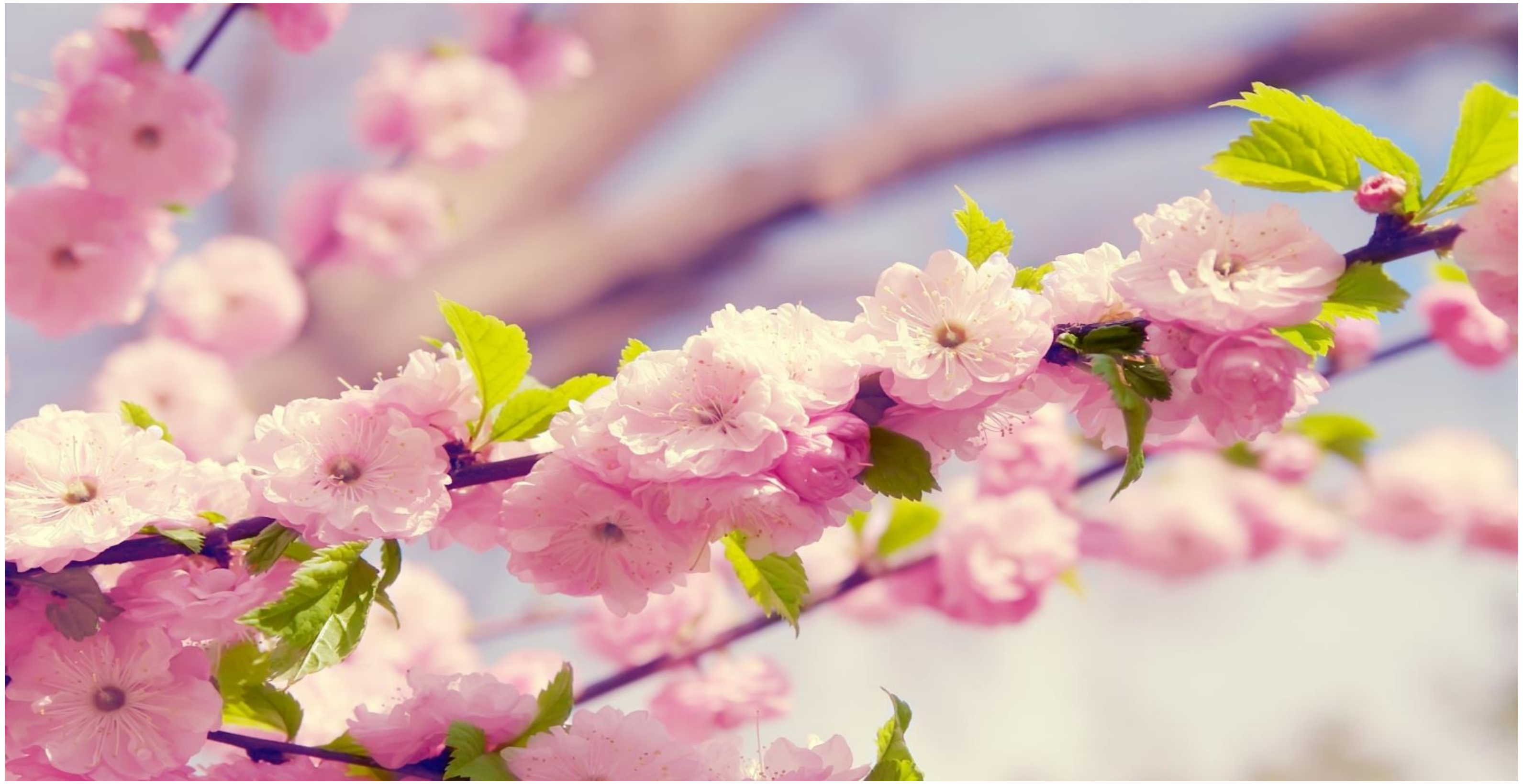

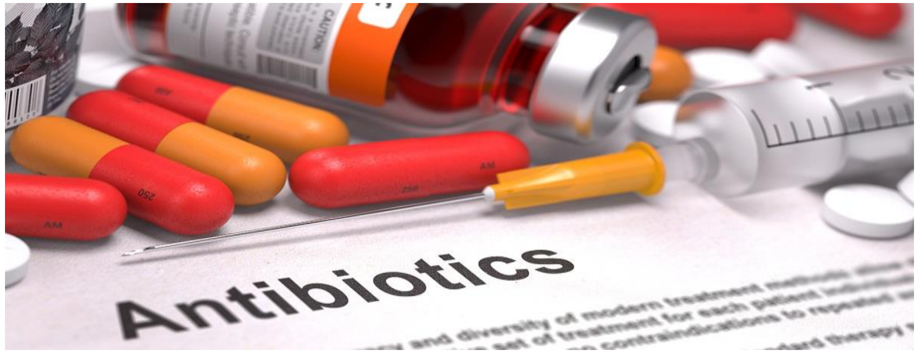

Background (1)

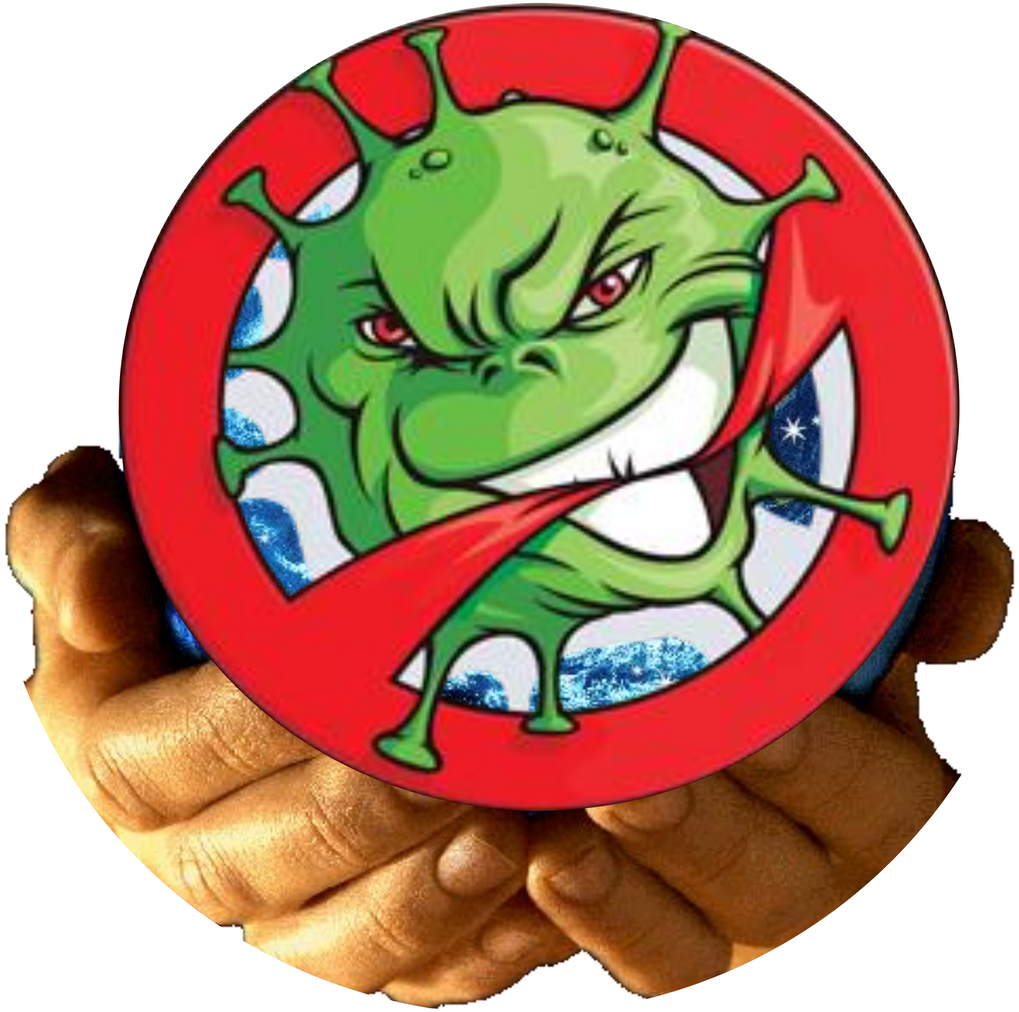

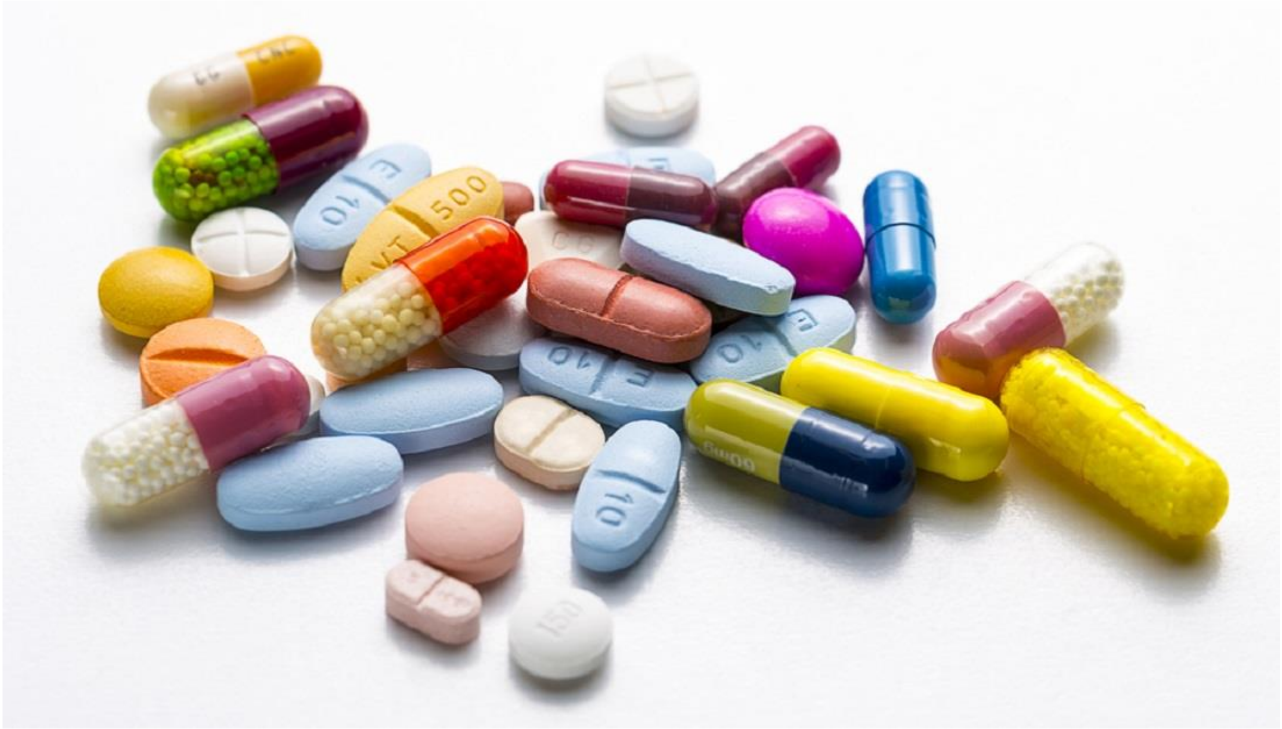

Background (2)

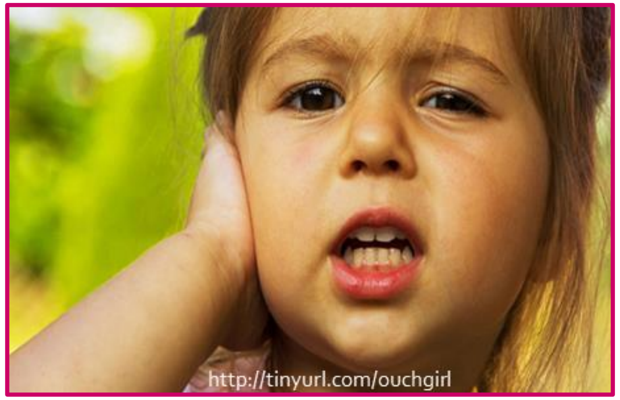

60% of AOM cases will be resolved in 24 hours

Treatment

Pain management

Observation

Antibiotics

80% of AOM cases will be resolved in 72 hours

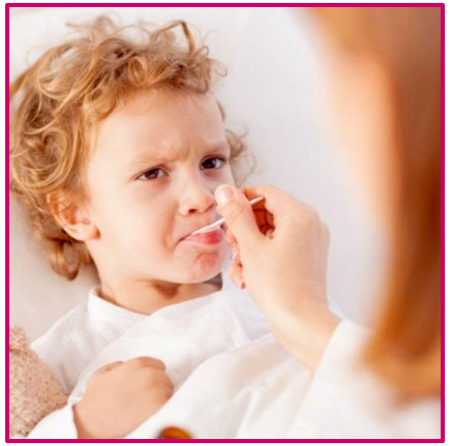

Australia: 89% of new AOM cases were treated with antibiotics (2010 – 2015).
Our survey study (2016) demonstrated that 88% of physicians would prescribe antibiotics for mild AOM.

Alternative treatment for AOM

Corticosteroids

Clinical practice guidelines
versus
Daily clinical practice

Clinical trials of corticosteroids for AOM

Cochrane Library

Cochrane Database of Systematic Reviews

Systemic corticosteroids for acute otitis media in children (Protocol)

Ranakusuma RW, Pitoyo Y, Safitri ED, Thorning S, Beller EM, Sastroasmoro S, Del Mar CB

Ranakusuma RW, Pitoyo Y, Safitri ED, Thorning S, Beller EM, Sastroasmoro S, Del Mar CB.
Systemic corticosteroids for acute otitis media in children.
Cochrane Database of Systematic Reviews 2016, Issue 7. Art. No.: CD012289.
DOI: 10.1002/14651858.CD012289.

www.cochranelibrary.com

Oral prednisolone is an effective adjuvant therapy for acute otitis media with discharge through tympanostomy tubes

Aino Ruohola, MD, Terho Heikkinen, MD, Jussi Jero, MD, Tuomo Puhakka, MD, Taina Juvén, MD, Mervi Närkiö-Mäkelä, MD, Harri Saxén, MD, and Olli Ruuskanen, MD

Objective: To determine the efficacy of a short course of oral prednisolone as an adjuvant therapy for acute otitis media draining through tympanostomy tubes.

Study design: In a randomized, double-blind, placebo-controlled study, children with acute discharge (<48 hours) through tympanostomy tubes received either prednisolone (2 mg/kg/d; n = 23) or placebo (n = 27) for 3 days. All children received amoxicillin/clavulanate (40/10 mg/kg/d) for 7 days. The children were examined daily at the study clinic until the drainage ceased.

Results: The median duration of otorrhea in the prednisolone group was 1.0 days (25% to 75% range, 1.0 to 2.0 days), compared with 3.0 days (25% to 75% range, 2.0 to 4.0 days) in the children receiving placebo ($P < .001$). The duration of otorrhea was ≤2 days in 21 (91%) children in the prednisolone group, compared with 8 (30%) children in the placebo group ($P < .001$).

Conclusions: Oral prednisolone appears to be modestly effective adjuvant therapy for acute otitis media with discharge through tympanostomy tubes in children. Further studies seem warranted to determine whether short-term use of steroids early during the course of acute otitis media would also reduce the duration of middle ear effusion in children with intact tympanic membranes. (J Pediatr 1999;134:459-63)

Research question

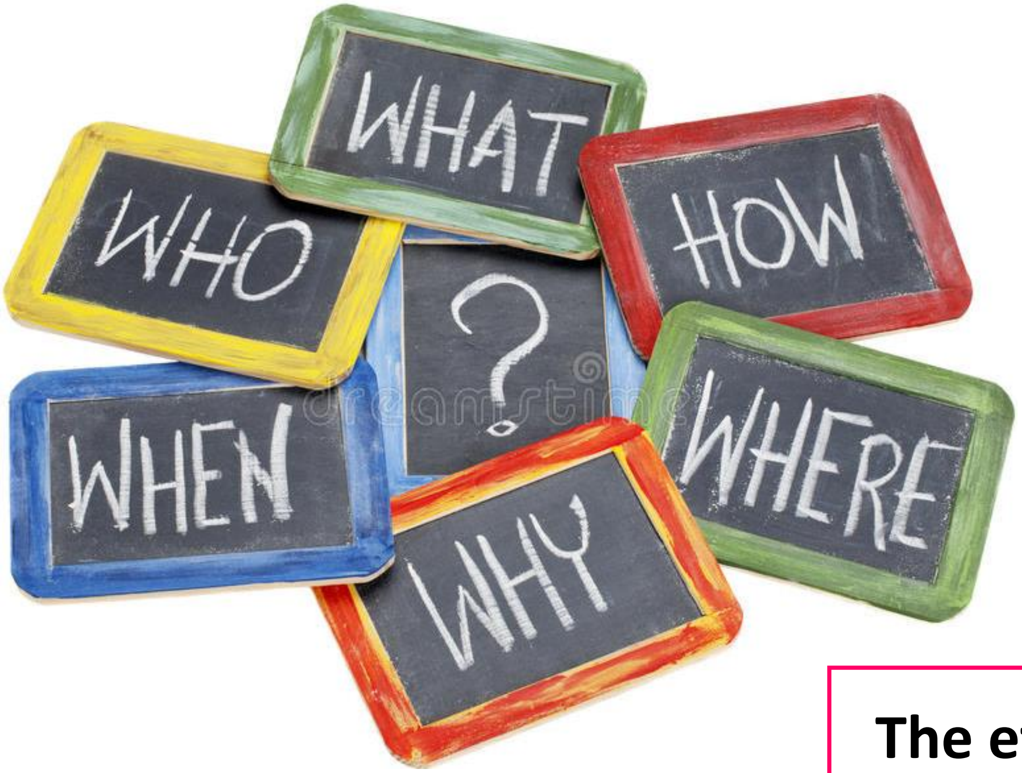

Research Gap

↓

A quality clinical trial

↓

The effectiveness of corticosteroids as a monotherapy or as an addition to antibiotics for AOM

A pragmatic, parallel, randomized, placebo-controlled, double blind study of corticosteroids for AOM in children

PREDNISOLONE

Doses

10 mg/day: aged 6 months to < 2 years
20 mg/day: aged 2 years to < 6 years
30 mg/day: →aged 6 years to 12 years

Otitis media clinical trial and national/international guidelines of paediatric infectious and inflammation disorders.

Range of prednisolone dose:
0.25 – 2 mg/kg body weight/day

Frequency

Once a day, in the morning

Single dose to prevent the suppression of hypothalamic-pituitary-adrenal (HPA) axis

Duration

5 (five) days

Ranged 3 to 7 days

AOM cytokines peaked: 3 hours to 3 days.
Progressively decreased: 4 to 6 days
Normalization: > 6 days

Side effects?

Objectives of the study

**Primary objectives**	To assess the overall process and procedures of a large main study, including (1) the recruitment criteria; (2) the process of stratification and randomisation; (3) clinical outcomes measures using validated and customized tools.
	To identify any practical and operational issues that potentially occur in the large main study.
	To verify a sample size calculation for the large main study.
**Secondary objectives**	To identify and explain the mechanism of corticosteroids in improving middle ear effusion and other clinical symptoms of AOM in a mechanistic sub-study using tympanometry.

A Pilot study and a mechanistic sub-study

P	Children aged 6 month to 12 years with acute otitis media (AOM) [N=60].
I	Oral prednisolone plus expectant observation (mild AOM)<br>OR<br>Oral prednisolone plus antibiotics (severe AOM)
C	Without prednisolone plus expectant observation (mild AOM)<br>OR<br>Without prednisolone plus antibiotics (severe AOM)
O	Recruitment rates<br>The success of study procedures<br>Ability to measure planned outcomes in main study<br>Compliance to study and study drug<br>The verification of sample size calculation for main study
	The change in middle ear effusion at various time points<br>Duration of middle ear effusion<br>The correlation between ear pain and other symptoms with the changes in MEE at various time points
T	9 months<br>[Dr Cipto Mangunkusumo Hospital, Persahabatan Hospital, Gatot Soebroto Army Hospital, Antam Medika Hospital, Islamic Hospital, Proklamasi ENT Hospital]

Study timeline

	STUDY PERIOD				
	Enrolment Allocation	Post-allocation			Close-out
**TIMEPOINT**	**0**	t_1 (day-3 to -5)	t_2^* (day-7 to -9)	t_3^* (day-30 to -40)	t_4^* (day-90 to -100)
**ENROLMENT:**					
Eligibility screen	X				
Informed consent	X				
Allocation	X				
**INTERVENTIONS:**					
[Intervention A] Prednisolone	——	——	——		
[Intervention B] Control	——	——	——		
**ASSESSMENTS:**					
Baseline examination (weight, height, BP, temperature)	X	X	X	X*	X*
Severity of pain and duration using VAS	X	X	X		
Overall symptoms and its duration using AOM-SOS	X	X	X		
Adherence to trial drug	X	X	X		
Adverse effects	X	X	X		
Otoscopic examination	X	X	X	X**	X**
Tympanometry examination	X+	X+	X+	X+	X+
Complication	X	X	X		
Recurrence of AOM				X*	X*

Recruitment process

Initial screening
[Attending nurse & Phycisian]

FORM01. Study recruitment log book

Ear pain in the past 48 hours
OR
Holding/pulling out the ear, irritable in the past 48 hours
OR
Experiencing ear discharge in the past 48 hours

Consent to study
[Physician]

CRF01. Information sheet and consent form

The research summary
The overall procedures in the study, including the follow-up visit
Potential side effects
Compensation
Voluntary participation

Recruitment based on eligibility criteria
[Physician]

CRF03. Eligibility form

Inclusion criteria	Exclusion criteria
children (6 months – 12 years) with AOM, defined as a current onset within 48 hours of ear-related symptoms (e.g. ear pain, ear tugging/rubbing or irritability) and if possible to assess, otoscopic findings of acute inflammation (e.g. erythema) and middle ear effusion (e.g. bulging, air-fluid level)	Children with or who:<br>1. major and severe medical conditions (e.g. heart/kidney failure)<br>2. immunocompromised children (e.g. HIV, in cancer treatment)<br>3. congenital malformations and/or syndromes (e.g. cleft palate)<br>4. high risk of strongyloidiasis infection<br>5. ear ventilation tube(s)<br>6. had exposed to persons with varicella or active Zoster infection in the past 3 weeks without any prior varicella immunization/ infection<br>7. have taken oral or topical steroids in the preceding four weeks<br>8. have taken antibiotics in the preceding two weeks<br>9. are hypersensitive to prednisolone other corticosteroids.

Stratification
[Physician]

CRF03. Eligibility form

Mild AOM	Severe AOM
• Mild ear pain<br>• fever < 39°C<br>• Otoscopy: mild bulging<br>• Complication (-)	• Moderate to severe ear pain<br>• Fever ≥ 39°C<br>• Otoscopy: moderate-to-severe bulging, suppurative appearance<br>• Children aged < 2 years with bilateral AOM<br>• AOM with tympanic membrane perforation

Data collection

Outcome identification [Physician]

CRF04. Baseline information form
CRF05. Outcome form
CRF06. Symptom diary
CRF07. Prescription of study medication
FORM07. Guideline of antibiotics for AOM
FORM08. Prednisolone dose for OPAL study

Baseline information and history
General examination
ENT examination, including otoscopy
Tympanometry examination
Severity of AOM symptoms using VAS and AOM-SOS
Complications of AOM
Previous and current treatment
Prescribe the study medication (and antibiotics for severe AOM) and other symptomatic medications
Teach how to complete a symptom diary

Tympanometry examination [Audiologist & Physician]

CRF05. Outcome form

Tympanometry examination
Interpret the tympanometry results

Randomisation [An appointed nurse for randomosation]

CRF08. Randomisation form

Mild AOM		Severe AOM	
Observation + Prednisolone	Observation alone	Antibiotics + Prednisolone	Antibiotics alone

CRF07. Prescription of study medication
CRF06. Symptom diary
CRF09. Follow-up visit card
FORM09. Instruction of prednisolone use for parents
FORM10. Lupred pharmaceutical brochure
CRF02. Study registration form

Dispense the prescription of study medication (prednisolone group), including provide the Information of prednisolone use
Confirm the completion of symptom diary
Complete the study registration form
Observation and identification of complication and side effects
Follow-up visits and the card
Keep the intervention allocation confidential
Provide study souvenirs and transport reimbursement

Case report form binder / Confidential study document binder

Check the completeness all study documents and separate the confidential ones to the confidential study document binder, as follows:
Confidential study document binder: (1) FORM01. Study recruitment log book; (2) CRF01. Consent form; (3) CRF02. Study registration form; and (4) CRF08. Randomisation form.
Case report form: (1) CRF03. Eligibility form; (2) CRF04. Baseline information form; (3) CRF05. Outcome form; (4) CRF10. Serious adverse event reporting form; (5) CRF11. Feedback form

dr. Respati W. Ranakusuma, SpTHT-KL
Clinical Epidemiology & Evidence-Based Medicine (CEEBM) Unit Dr. Cipto Mangunkusumo Hospital – Faculty of Medicine Universitas Indonesia
Oral prednisolone for acute otitis media in children: a pilot, pragmatic, randomised, open-label, single-blind study (OPAL Study)

FORM01 – STUDY RECRUITMENT LOG BOOK

Nurse name/ID :

Study title :
Oral prednisolone for acute otitis media in children: a pilot, pragmatic, randomised, open-label, single-blind, controlled study (OPAL study)

Hospital ID :

Study registration ID	Patient's name	Date screened	Has your child experien-cing ear pain in the past 48 hours? (YES or NO)	Has your child been tugging or rubbing her/his ear(s) and been more irritable or fussy or crying more than usual over the past 48 hours (YES or NO)	Has your child been experiencing ear discharge in the past 48 hours? (YES or NO)	Body weight (kg)	Body height (cm)	Tempe-rature (°C)	Blood pressure (mmHg)	Did patient go on the study? (YES or NO)	If YES, what is the Randomisation ID	If NO, please tell us reason not on the study below		
												Not eligible (YES or NO)	Did not give consent (YES or NO)	Was not approached (YES or NO). Write the reason

CONSENT TO THE STUDY (1)

REGISTRATION ID

dr. Respati W. Ranakusuma, SpTHT-KL
Clinical Epidemiology & Evidence-Based Medicine Unit, Dr. Cipto Mangunkusumo Hospital – Faculty of Medicine Universitas Indonesia
Oral Prednisolone for acute otitis media in children: a pilot pragmatic, randomised, open-label, single-blind study (OPAL Study)

CEEBM RSCM KEDOKTERAN CREBP BOND UNIVERSITY

PARTICIPANT INFORMATION SHEET AND CONSENT FORM

Oral prednisolone for acute otitis media in children: a pilot pragmatic randomised open-label single-blind controlled study (OPAL study)

[Steroids for middle ear infection in children]

Invitation

You are invited to participate in a research study into the use of steroids (prednisolone) or an anti-inflammatory drug for middle ear infection in children.

The study is being conducted by Dr. Respati W. Ranakusuma, an otorhinolaryngologists and a researcher at the Clinical Epidemiology and Evidence-Based Medicine (CEEBM) Unit Dr. Cipto Mangunkusumo Hospital–Faculty of Medicine Universitas Indonesia. This is part of an international collaborative study between CEEBM CMH-FMUI and the Centre for Research in Evidence-Based Practice (CREBP), Faculty of Health Sciences and Medicine Bond University, Queensland, Australia.

Before you decide whether or not you wish to participate in this study, it is important for you to understand why the research is being done and what it will involve. Please take the time to read the following information carefully and discuss it with others if you wish.

1. What is the purpose of this study?
   The purpose is to investigate whether steroids, as an alternative treatment, will reduce ear pain and other symptoms in children with acute or recent (less than 48 hours) middle ear infection. This study is part of a doctoral project at the CREBP Bond University, Queensland, Australia. As this is a pilot study, we also want to know your experience during the study. For example, the obstacles you found in giving the steroid to your child or completing the symptom diary daily.

2. Why have my child and I been invited to participate in this study?
   Your child and you have been invited to participate in this study because your child age ranges between six months to 12 years and having symptoms and signs of acute middle ear infection, such as ear pain in the past 48 hours, or holding or tugging her/his ear more frequently, more irritable, show lack of playfulness and/sleep in a young age (baby). If visible, from the ear examination using an otoscope, the ear drum(s) will show redness or yellowish, bulging, or discharge. Otoscope is a tool consisted of a lamp, magnifying glass, and a silicone/plastic probe that will be inserted into the ear canal to identify the condition of the ear canal and ear drum. This examination is painless.

3. What does participation in this study involve?
   If you agree to participate in this study, your physician will ask you more questions regarding the history of your

CONSENT FORM

Oral prednisolone for acute otitis media in children: a pilot pragmatic, randomised, open-label, single-blind study (OPAL study)

[Steroids for middle ear infection in children]

1. I, ______________________________
   of ______________________________
   agree to participate in the study described in the participant information statement set attached to this form.
2. I acknowledge that I have read the participant information statement, which explains why my child has been selected, the aims of the study, and the nature and the possible risks of the investigation, and the statement has been explained to me to my satisfaction.
3. Before signing this consent form, I have been given the opportunity of asking any questions relating to any possible physical and mental harm my child might suffer as a result of my child participation and I have received satisfactory answers.
4. I understand that I can withdraw from the study at any time without prejudice to my relationship to my physician and the ______________________ Hospital.
5. I agree that research data gathered from the results of the study may be published, provided that I cannot be identified.
6. I understand that I have any questions relating to my participation in this research, I may contact Dr. Respati W. Ranakusuma, ORL on telephone +62 8111 012 185, who will be happy to answer them.
7. I acknowledge receipt of a copy of this Consent Form and the Participation Information Statement.

Complaints may be directed to the OPAL Study Support Office at the Clinical Epidemiology and Evidence-Based Medicine Unit, Dr Cipto Mangunkusumo Hospital – Faculty of Medicine Universitas Indonesia, Building H Dr Cipto Mangunkusumo Hospital, Diponegoro 71, Jakarta 10430, Indonesia (phone +62 21 316 1760, email OPAL.study@bond.edu.au).

Signature of participant aged 12 years old	Name	Date
______________________	______________________	______________________
Signature of the parent	Name	Date
______________________	______________________	______________________
Signature of witness	Name	Date
______________________	______________________	______________________

Statement by the researcher/person taking consent

I have accurately read out the information sheet to the potential participant, and to the best of my ability made sure that the participant understands that the following will be done:

1. I will collect all clinical information throughout clinical history taking, general examination, and ear, nose, and throat assessment (using otoscope, tympanometry in some cases), questionnaires/forms, feedback forms, and symptom diary
2. The patient has to come to the hospital for four times following the first visit for three months
3. The patient will be located to treatment with antibiotic or 48-hours observation with re-assessment at the end of the observation. Either way, the patient will receive a research medicine (prednisolone) or without
4. The patient or patient's parent(s) or the parents are aware of any potential unwanted effects that may occur during the research
5. The patient or patient's parent(s) should record the patient's condition, the adherence of trial drug intake, and unwanted effects by completing a symptom diary that will provided at the first visit

I confirm that the participant (patient and/or patient's parent(s)) was given an opportunity to ask questions about the study, and all the questions asked by the participant have been answered correctly and to the best of my ability. I confirm that the individual has not been coerced into giving consent, and the consent has been given freely and voluntarily.

Signature of investigator	Name	Date
______________________	______________________	______________________

REVOCATION OF CONSENT

Oral prednisolone for acute otitis media in children: a pilot pragmatic, randomised, open-label, single-blind study (OPAL study)

[Steroids for middle ear infection in children]

I hereby wish to WITHDRAW my consent to participate in the study described above and understand that such withdrawal WILL NOT jeopardise any treatment or my relationship with the ________________________ hospital or my medical attendants.

Signature of participant or the parent	Name	Date
______________________	______________________	______________________

The section for Revocation of Consent should be forwarded to Dr. Respati W. Ranakusuma, ORL at the Clinical Epidemiology and Evidence-Based Medicine Unit, Dr Cipto Mangunkusumo Hospital – Faculty of Medicine Universitas Indonesia.

Obtaining the consent to the study

In obtaining and documenting IC, the investigator should comply with the applicable regulatory requirement(s), GCP and to the ethical principles that have their origin in the Declaration of Helsinki.

The subject or the subject’s legally acceptable representative should be informed in a timely manner if new relevant information becomes available. The communication of this information should be documented.

Neither the investigator, nor the trial staff, should coerce or unduly influence a subject to participate or to continue to participate in a trial.

None of the oral and written information concerning the trial, including the written IC form, should contain any language that causes the subject or the subject's legally acceptable representative to waive or to appear to waive any legal rights, or that releases or appears to release the investigator / institution for negligence.

The investigator, or a person designated by the investigator, should fully inform the subject or, if the subject is unable to provide IC, the subject's legally acceptable representative, of all pertinent aspects of the trial including the written information and the approval/ favourable opinion by the IRB/IEC.

Information in the consent form

- The trial involves research
- Purpose of the trial
- The trial treatment(s) & the probability for random assignment to each treatment.
- The trial procedures to be followed
- The subject's responsibilities.
- Those aspects of the trial that are experimental
- The reasonably foreseeable risks or inconveniences to the subject
- The reasonably expected or no intended benefits, including the risks
- The alternative procedure(s) or course(s) of treatment that may be available to the subject, including important potential benefits and risks.
- The compensation and/or treatment available to the subject in the event of trial related injury.
- The anticipated prorated payment and anticipated expenses (if any)
- Voluntary participation and the subject may refuse to participate or withdraw from the trial, at any time
- The auditor(s) or IRB/IEC will be granted direct access to the subject's medical records for verification of clinical trial procedures
- The records identifying the subject will be kept confidential
- the subject or the subject's legally representative will be informed if relevant information becomes available
- The person(s) to contact for further information regarding the trial , including trial-related injury events
- The foreseeable circumstances and/or reasons under which the subject's participation in the trial may be terminated.
- The expected duration of the trial, including the approximate number of subjects involved in the trial.

CRF03 – ELIGIBILITY FORM

INCLUSION CRITERIA	
O Yes O No	Definite or suspected acute otitis media (AOM)<br>OR<br>Were you able to confirm otoscopically? O Yes O No
O Yes O No	Aged 6 months to 12 years
O Yes O No	Available for follow-up visits

EXCLUSION CRITERIA	
O Yes O No	Major medical conditions (e.g. heart failure, renal insufficiency, DM, peptic ulcers)
O Yes O No	Immunocompromised (e.g. cancer treatment, HIV)
O Yes O No	Congenital malformation/syndromes (e.g. cleft palate)
O Yes O No	Ventilation tube(s)
O Yes O No	Exposed to persons with varicella/active Zoster infection in the past 3 weeks with no prior history of varicella infection/ immunisation
O Yes O No	With high risk of strongyloidiasis infection
O Yes O No	Has taken oral/injection/topical steroids in the past 4 weeks
O Yes O No	Has taken antibiotics in the past 2 weeks
O Yes O No	Hypersensitive to prednisolone or other steroids

Is this child eligible for the trial?

All 'YES' at the inclusion criteria, AND
All 'NO' at the exclusion criteria

Eligible, then INCLUDE O

At least one 'NO' at the inclusion criteria, OR
At least one 'YES' at the exclusion criteria

Not eligible, then EXCLUDE O

Obtaining the CONSENT

NOT giving CONSENT → **EXCLUDE** O

Giving CONSENT → **INCLUSION** O

Do they have these following symptoms?

O Yes O No	Moderate to severe symptoms, locally or systemically (moderate to severe ear pain, fever ≥39°C, complications)
O Yes O No	Aged younger than 2 years with bilateral acute otitis media
O Yes O No	With perforation of tympanic membrane(s)
O Yes O No	If visible, otoscopic finding shows moderate to severe bulging and/or yellowish purulent tympanic membrane(s)

All 'NO' → **MILD AOM** O

At least one 'YES' → **SEVERE AOM** O

CRF03 – ELIGIBILITY FORM

INCLUSION CRITERIA	
O Yes O No	Definite or suspected acute otitis media (AOM)<br>OR<br>Were you able to confirm otoscopically?<br>O Yes O No
O Yes O No	Aged 6 months to 12 years
O Yes O No	Available for follow-up visits

EXCLUSION CRITERIA	
O Yes O No	Major medical conditions (e.g. heart failure, renal insufficiency, DM, peptic ulcers)
O Yes O No	Immunocompromised (e.g. cancer treatment, HIV)
O Yes O No	Congenital malformation/syndromes (e.g. cleft palate)
O Yes O No	Ventilation tube(s)
O Yes O No	Exposed to persons with varicella/active Zoster infection in the past 3 weeks with no prior history of varicella infection/ immunisation
O Yes O No	With high risk of strongyloidiasis infection
O Yes O No	Has taken oral/injection/topical steroids in the past 4 weeks
O Yes O No	Has taken antibiotics in the past 2 weeks
O Yes O No	Hypersensitive to prednisolone or other steroids

Is this child eligible for the trial?

All 'YES' at the inclusion criteria, AND
All 'NO' at the exclusion criteria

Eligible, then INCLUDE O

At least one 'NO' at the inclusion criteria, OR
At least one 'YES' at the exclusion criteria

Not eligible, then EXCLUDE O

Obtaining the CONSENT

NOT giving CONSENT — **EXCLUDE** O

Giving CONSENT — **INCLUSION** O

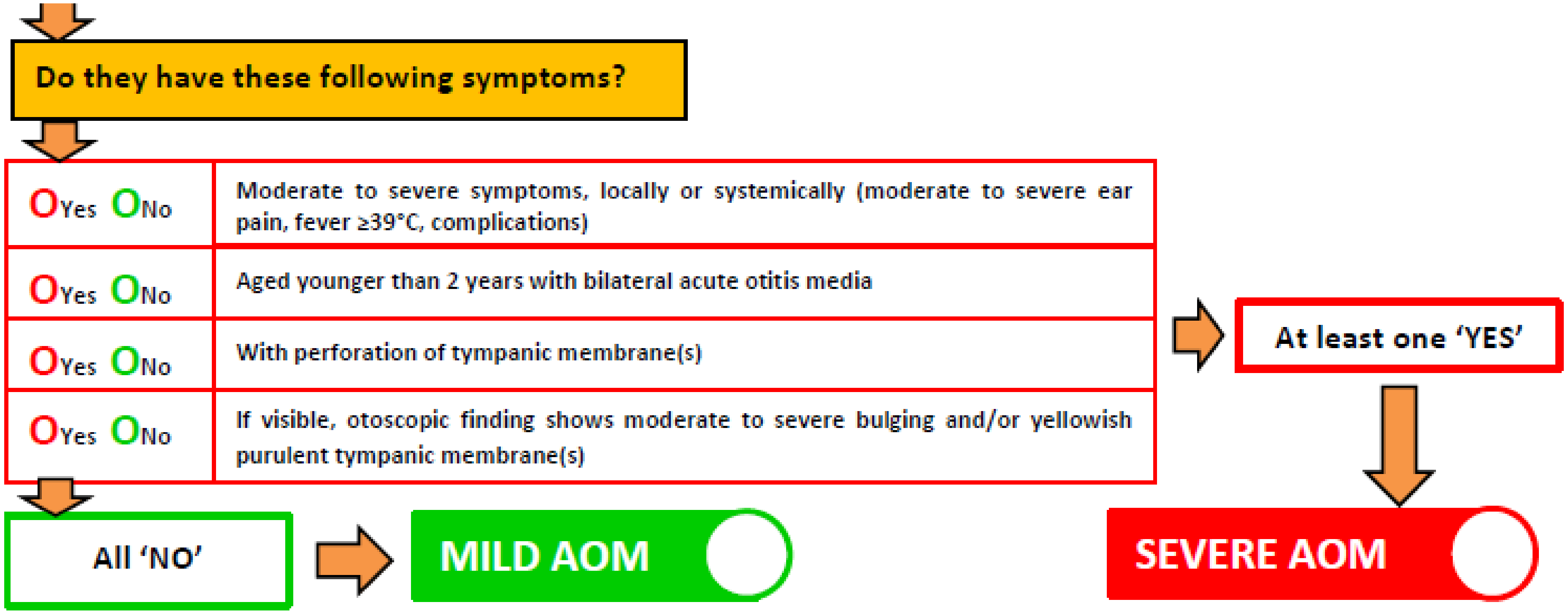
Do they have these following symptoms?
OYes ONo Moderate to severe symptoms, locally or systemically (moderate to severe ear pain, fever ≥39°C, complications)
OYes ONo Aged younger than 2 years with bilateral acute otitis media
OYes ONo With perforation of tympanic membrane(s)
OYes ONo If visible, otoscopic finding shows moderate to severe bulging and/or yellowish purulent tympanic membrane(s)
All 'NO'
MILD AOM
At least one 'YES'
SEVERE AOM

ELIGIBILITY RESULT (by Physician)

dr. Respati W. Ranakusuma, SpTHT-KL
Clinical Epidemiology & Evidence-Based Medicine (CEEBM) Unit Dr. Cipto Mangunkusumo Hospital – Faculty of Medicine Universitas Indonesia
Oral prednisolone for acute otitis media in children: a pilot, pragmatic, randomised, open-label, single-blind study (OPAL Study)

FORM01 – STUDY RECRUITMENT LOG BOOK

Nurse name/ID :	Study title : Oral prednisolone for acute otitis media in children: a pilot, pragmatic, randomised, open-label, single-blind, controlled study (OPAL study)	Hospital ID :

Study registration ID	Patient's name	Date screened	Has your child experien-cing ear pain in the past 48 hours? (YES or NO)	Has your child been tugging or rubbing her/his ear(s) and been more irritable or fussy or crying more than usual over the past 48 hours (YES or NO)	Has your child been experiencing ear discharge in the past 48 hours? (YES or NO)	Body weight (kg)	Body height (cm)	Body tempe-rature (ºC)	Blood pressure (mmHg)	Did patient go on the study? (YES or NO)	If YES, what is the Randomisation ID	If NO, please tell us reason not on the study below		
												Not eligible (YES or NO)	Did not give consent (YES or NO)	Was not approached (YES or NO). Write the reason

CRF04 – BASELINE INFORMATION FORM

1 Did (do) you breastfeed your child? O Yes O No

If ' YES', until the age of O ≤ 2 months O > 2 – 6 months O > 6 months

2 Does your child attend a day-care O Yes O No

How many days in a week? O ≤ 2 days O > 2 days

3 Have your child had a pneumococcus vaccine (PCV)? O Yes O No O Do not know

How many times: __________ times

4 Have your child had an influenzae vaccine? O Yes O No O Do not know

How many times: __________ times

5 How many episodes of recurrent acute respiratory infection (runny nose, cough, sore throat, fever) in the past year? O ≤ 3 episodes O > 3 episodes to 6 episodes O > 6 episodes

6 Did your child have a history of 3 or more episodes of ear infection (ear pain, ear discharge, diarrhoea, or vomiting) during the past 12 months? O Yes O No

7 At what age did the first episode of ear infection start?

O ≤ 6 months O > 6 to 12 months O >12 to 24 months O > 2 to 5 years O > 5 years

8 Does your child have one of the following disorders:

O Bronchial asthma

O Allergic rhinitis

O Family history of atopic disorders

O None of above

9 Number of children (including the patient) who live in the house ___________ children

10 Number of persons who smoke at home ___________ person(s)

CRF05 – OUTCOME FORM

Baseline Visit (Day-0) : |___|___| – |___|___| – 20 |___|___|

Complications (for Physician)

1	Does your child experience discharge from the ear(s)?	O Yes O No
2	Does your child experience intense ear pain and pain behind the ear?	O Yes O No
3	Does your child experience swelling/bulging/ or redness/tenderness of the ear(s)?	O Yes O No
4	Does your child experience facial asymmetry (e.g. when the child smiles, cries)?	O Yes O No

General and ENT examination (for Nurse and Physician)

5.1 Weight _____ kg | 5.2 Height _______ cm | 5.3 Temp. _________ °C | 5.4 BP _____ / _____ mmHg

6 Nose O Normal O Oedema O Hyperaemic O Livid O Serous discharge O Mucoid discharge

7 Tonsils O Normal O Hyperaemic O Detritus O Tonsil(s) T1 O Tonsil(s) T2 O Tonsil(s) T3-4

8 Pharynx O Normal O Hyperaemic O Oedema O Granules O Post nasal drip (PND)

9 Otoscopic examination

O Normal O Cerumen O Erythema O Air fluid level O Complete effusion O Opacification

O Mild bulging O Moderate to severe bulging (bulging rounded) O Bulla O Perforation

10 Medicines that have been taken before the baseline visit (please circle your dose measurement)

1. ____________________ Dose : _____mg perBw kg / Teaspoon / Tablespoon ; Frequency : ____/ day
2. ____________________ Dose : _____mg perBW kg / Teaspoon / Tablespoon ; Frequency : ____/ day
3. ____________________ Dose : _____mg perBw kg / Teaspoon / Tablespoon ; Frequency : ____/ day
4. ____________________ Dose : _____mg perBw kg / Teaspoon / Tablespoon ; Frequency : ____/ day
5. ____________________ Dose : _____mg perBW kg / Teaspoon / Tablespoon ; Frequency : ____/ day

Medicines prescribed by physician (you) at the baseline visit

Antibiotic	______________________________<br>Dose : _______mg / BW kg Frequency : _______ / day for _______ days

Other medicine(s)

1. ____________________ Dose : _____mg perBw kg / Teaspoon / Tablespoon ; Frequency : ____/ day
2. ____________________ Dose : _____mg perBw kg / Teaspoon / Tablespoon ; Frequency : ____/ day
3. ____________________ Dose : _____mg perBw kg / Teaspoon / Tablespoon ; Frequency : ____/ day
4. ____________________ Dose : _____mg perBw kg / Teaspoon / Tablespoon ; Frequency : ____/ day
5. ____________________ Dose : _____mg perBw kg / Teaspoon / Tablespoon ; Frequency : ____/ day

Outcome: Symptoms (for patients and the parents. Physician will help them to complete these in the symptom diary)

11 Please place a vertical line across the available horizontal line that best describes your or your child's pain during the past 24 hours?

No Pain |————————————————| Pain As Bad As It Could Possibly Be

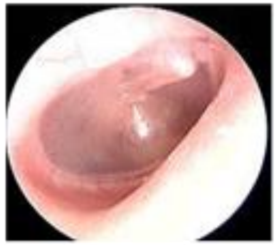

Normal appearance

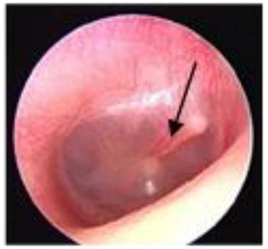

Erythema only (without effusion)

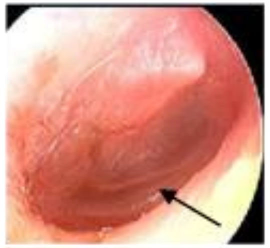

Erythema with air-fluid level (no opacification)

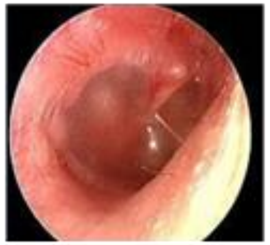

Erythema with complete effusion (no opacification)

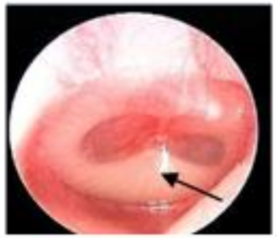

Opacification and mild bulging

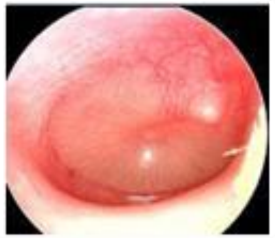

Opacification, complete effusion, mild bulging

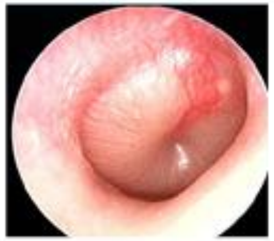

Erythema, complete effusion, bulging (moderate to severe)

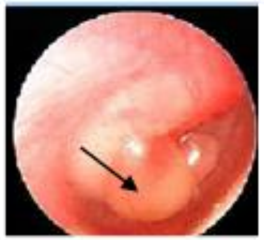

Bulla

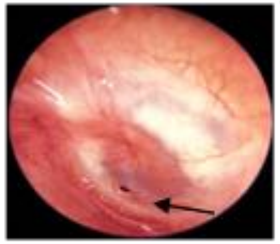

Perforated tympanic membrane

2 Please place a vertical line across the available horizontal line that best describes your or your child's pain during the past 24 hours? (if applicable)

No Pain ———————————————— Pain As Bad As It Could Possibly Be

3. We are interest finding out how your child has been doing. For each question, please place a checkmark (V) in the circle corresponding to your child's symptoms. Please answer all questions (if applicable).

3.1 Over the past 12 h, has your child been tugging, rubbing, or holding the ear(s) more than usual?	O No	O A little	O A lot
3.2 Over the past 12 h, has your child been crying more than usual?	O No	O A little	O A lot
3.3 Over the past 12 h, has your child been more irritable or fussy than usual?	O No	O A little	O A lot
3.4 Over the past 12 h, has your child been having more difficulty sleeping than usual?	O No	O A little	O A lot
3.5 Over the past 12 h, has your child been less playful or active than usual?	O No	O A little	O A lot
3.6 Over the past 12 h, has your child been eating less than usual?	O No	O A little	O A lot
3.7 Over the past 12 h, has your child been having fever or feeling warm to touch?	O No	O A little	O A lot

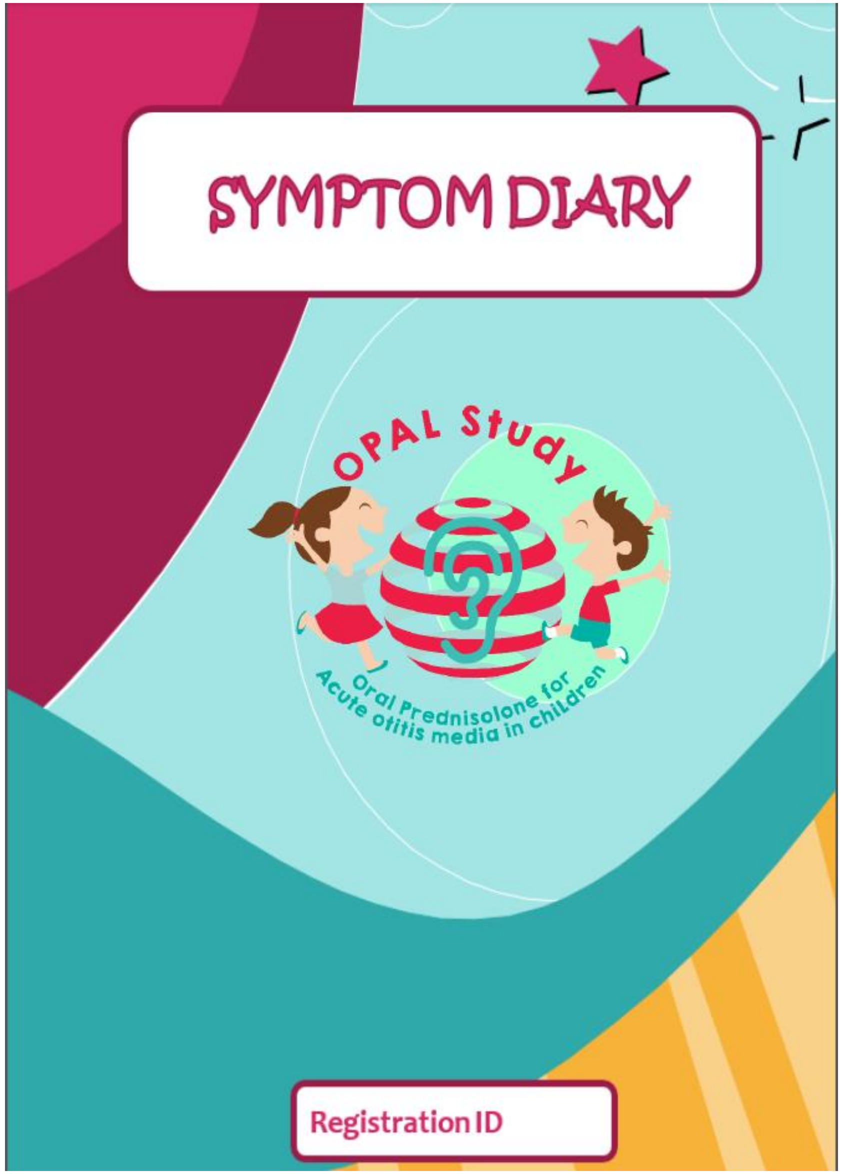

dr. Respati W. Ranakusuma, SpTHT-KL
Clinical Epidemiology & Evidence-Based Medicine Unit, Dr. Cipto Mangunkusumo Hospital – Faculty of Medicine Universitas Indonesia
Oral Prednisolone for acute otitis media in children: a pilot pragmati, randomised, open-label, single-blind, controlled study (OPAL Study)

FORM07. GUIDELINE OF ANTIBIOTICS FOR ACUTE OTITIS MEDIA

Initial immediate or delayed antibiotic therapy		Antibiotics after 48-72 hours of failure of initial antibiotic therapy	
Recommended first-line treatment	Alternative treatment (if penicillin allergy)	Recommended first-line treatment	Alternative treatment
Amoxicillin (80-90 mg/kg per day in 2 divided doses)<br><br>OR<br><br>Amoxicillin-clavulanate[a] (90 mg/kg per day of amoxicillin, with 6.4 mg/kg per day clavulanate (amoxicillin to clavulanate ration, 14:1) in 2 divided doses)	Cefdinir (14 mg/kg per day in 1 or 2 doses)<br><br>Cefuroxime (30 mg/kg per day in 2 divided doses)<br><br>Cefpodoxime (10 mg/kg per day in 2 divided doses)<br><br>Ceftriaxone (50 mg IM or IV per day for 1 or 3 days)	Amoxicillin-clavulanate[a] (90 mg/kg per day of amoxicillin, with 6.4 mg/kg per day in 2 divided doses)<br><br>OR<br><br>Ceftriaxone (50 mg IM or IV per day for 3 days)	Ceftriaxone, 3 days Clindamycin (30-40 mg/kg per day in 3 divided doses), with or without third-generation cephalosporin (50 mg IM or IV per day for 3 days)<br>Failure of second antibiotic<br>Clindamycin (30-40 mg/kg per day in 3 divided doses) plus third-generation cephalosporin<br>Tympanocentesis[b]<br>Consult specialist[b]

[a] *may be considered in patients who have received amoxicillin in the previous 3 0 days or who have the otitis conjunctivitis syndrome;*
[b] *Perform tympanocentesis/drainage if skilled in the procedure, or seek a consultation from an otolaryngologist for tympanocentesis/drainage if the tympanocentesis reveals multidrug/resistant bacteria, seek an infection disease specialist consultation.*

Reference: Lieberthal AS, Carroll AE, Chonmaitree T, et al. Clinical Practice Guideline: The diagnosis and management of acute otitis media. The American Academy of Pediatrics. *Pediatrics.* 2013;131:e964-e99

GUIDELINE OF PRDNISOLONE DOSE FOR OPAL STUDY

dr. Respati W. Ranakusuma, SpTHT-KL
Clinical Epidemiology & Evidence-Based Medicine Unit, Dr. Cipto Mangunkusumo Hospital – Faculty of Medicine Universitas Indonesia
Oral Prednisolone for acute otitis media in children: a pilot pragmatic, randomised, open-label, single-blind, controlled study (OPAL Study)

FORM08 – PREDNISOLONE DOSE FOR OPAL STUDY

Aged six months to up to two years old →	Dose : 10 mg / day (2 tablets per day) →	10 tablets of Prednisolone for five days
Aged two years to up to six years old →	Dose : 20 mg / day (4 tablets per day) →	20 tablets of Prednisolone for five days
Aged six years to 12 years old →	Dose : 30 mg / day (6 tablets per day) →	30 tablets of Prednisolone for five days

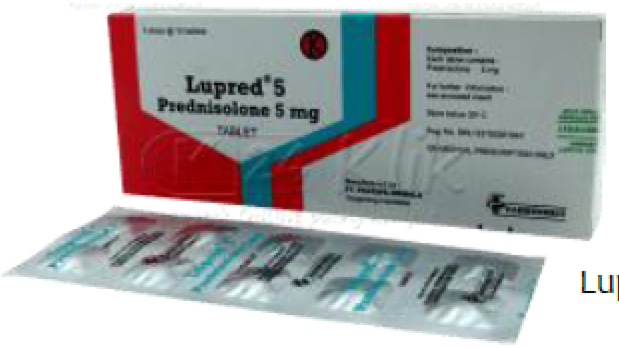

Lupred® 5 contains 5 mg prednisolone in each tablet

PRESCRIPTION OF STUDY MEDICATION (by Physician)

dr. Respati W. Ranakusuma, SpTHT-KL
Clinical Epidemiology & Evidence-Based Medicine Unit, Dr. Cipto Mangunkusumo Hospital – Faculty of Medicine Universitas Indonesia
Oral Prednisolone for acute otitis media in children: a pilot pragmatic, randomised, open-label, single-blind, controlled study (OPAL Study)

Date ____________________

CRF07. Prescription for OPAL study medication

Prednisolone doses:

- Aged 6 months to < 2 years old = 10 mg per day
- Aged 2 years to < 6 years old = 20 mg per day
- Aged 6 years to 12 years old = 30 mg per day

Registration ID : [ ]

Name : ______________________

Age : ______________ months / year(s) *[write and circle your answer]*

Study medication dose : _______ mg per day = _______ tablets per day

R/ OPAL study medication tablet ……..

Sach lact add

m.f. pulveres dtd No. V

∫ 1 dd 1 pc (before 9 am)

____

(sign here)

REGISTRATION ID | | | | |

Nurse ID : |___|___|___| Site ID : |___|___|___| Date : |___|___| – |___|___| – 201 |___|

CR08 – RANDOMISATION FORM

**Eligibility criteria (cross-check with 'FORM01. study registration log book', and 'CRF03. Eligibility form' in the 'Case Report Form Binder' of this subject).**									
All YES for all inclusion criteria						O Yes		O No	
All NO for all exclusion criteria						O Yes		O No	
**Consent to the study questions (cross-check with 'CRF01. Informed consent' in the 'Case Report Form Binder' of this subject).**									
Has consent given?						O Yes		O No	
**RANDOMISATION**									
**Father's mobile phone number**									
**Mother's mobile phone number**									
**Severity of AOM**	O Mild AOM					O Severe AOM			
**Subject's date of birth**	Date		Month		Year		**AGE**		Month/year
**RANDOMISATION RESULT**									
**Randomisation ID**									
**This subject is allocated to**	O Prednisolone group					O Control group (no prednisolone)			
**Prednisolone dosage (if the subject is allocated to prednisolone group)**	O 10 mg/day			O 20 mg/day			O 30 mg/day		
Nurse's signature		Nurse's name				Date			

RANDOMISATION BY RANDOMISATION SYSTEM WEBSITE

MASCOT.org.au Invite

MASCOT.org.au <noreply@mascot.org.au>

You have been invited to the MASCOT study randomization system.

Study: OPAL STUDY
Institution: Cipto Mangunkusumo Hospital

Please click the below link to begin submitting participants:

https://mascot.org.au/collaborate/8b8827f3-8572-4c4f-bb1a-4d4925e2c7a8

The MASCOT.org.au Team

Participant Enroller

OPAL STUDY

Welcome to the study and thank you for taking the time to collaborate.

Please ensure your details are correct below. If they are, you are welcome to continue.

Name: Respati Ranakusuma

Institution: Cipto Mangunkusumo Hospital

Begin

Participant Enroller

OPAL STUDY

Please enter a valid registration ID.

Registration ID: CM005

Submit ID

Participant Enroller

OPAL STUDY

This questionnaire will evaluate the eligibility of the candidate. If eligible, you may continue with the process.

Please completed all questions on behalf of your candidate.

Inclusion criteria (cross-check with 'FORM01. study registration log book' and 'CRF03. Eligibility Form' in the 'Case Report Form Binder' of this subject).

All YES for all inclusion criteria?

Yes ×

All NO for all exclusion criteria?

Select...

Consent to the study questions (cross-check with 'CRF01. Informed consent' in the 'Case Report Form Binder' of this subject).

Has consent been given

Select...

Check Eligibility

Participant Enroller

OPAL STUDY

Congratulations, your candidate is eligible. Please complete the following questions for submission to the study.

Subject's date of birth: 02/01/2016

Severity of AOM

- (●) Mild AOM
- ( ) Severe AOM

Submit Answers

Participant Enroller

OPAL STUDY

You have been assigned to:

Prednisolone group

Please select the correct dosage.

Subject's date of birth: 02/01/2016

Age: 2 years, 0 months old

Please verify this age is correct before proceeding.

Dosage

- ( ) 10mg (6 months up to 2 years)
- (●) 20mg (2 years up to 5 years)
- ( ) 30mg (5 years or older)

Submit

RANDOMISATION BY RANDOMISATION SYSTEM WEBSITE

PREDNISOLONE GROUP

Participant Enroller

OPAL STUDY

Thank you for your submission. Your candidate has been processed and enrolled into the study.

Please keep a copy of the submission overview for future reference.

Treatment	Prednisolone group
Dosage	20mg (2 years up to 5 years)
Registration ID	CM002
Randomisation ID	OPAL02
Study	OPAL STUDY
Enroller	Respati Ranakusuma
Institution	Cipto Mangunkusumo Hospital

Add Another Print

CONTROL GROUP

Participant Enroller

OPAL STUDY

Thank you for your submission. Your candidate has been processed and enrolled into the study.

Please keep a copy of the submission overview for future reference.

Treatment	Control group (no prednisolone)
Registration ID	CM003
Randomisation ID	OPAL03
Study	OPAL STUDY
Enroller	Respati Ranakusuma
Institution	Cipto Mangunkusumo Hospital

Add Another Print

RANDOMISATION STARTS → CALL RANDOMISATION CALL-CENTRE → PROVIDE RANDOMISATION INFORMATION

Registration ID label	Registration ID
CRF03. Eligibility form	All 'YES' for all inclusion criteria
CRF03. Eligibility form	All 'NO' for all inclusion criteria
CRF01. Consent form<br>CRF03. Eligibility form	Has consent been given?
CRF08. Randomisation form	Date of birth and age of the patient
CRF07. Prescription<br>FORM08. Prednisolone dose for OPAL study	Dose of the prednisolone
CRF03. Eligibility form	Severity of AOM

→ RANDOMISATION CALL-CENTRE WILL ACCESS THE RANDOMISATION SYSTEM → RANDOMISATION CALL-CENTRE PROVIDE RANDOMISATION RESULTS

Randomisation ID
Treatment group
Prednisolone dose for those allocated in the prednisolone group

↓

RECORD THE RANDOMISATION RESULT TO CRF08. RANDOMISATION FORM

32

CLOSING THE INIATIAL VISIT (by Appointed Nurse)

Dispensing CRF07.Prescription of study medication for children allocated to Prednisolone group

Provide FORM09. Instruction of prednisolone use for parents whose children were allocated to the Prednisolone group

Reimbursement for transportation cost

Study souvenirs

Follow-up visit card

Confirming the ability of parents to complete CRF05. Symptom diary

Completion of CRF02. Study registration form by the parents

Follow-up Visit Card

Name :
Address :
Dad/Mom's phone no :

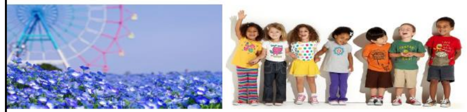

Clinical Epidemiology and Evidence-Based Medicine (CEEBM) Unit
Dr Cipto Mangunkusumo Hospital – Faculty of Medicine Universitas Indonesia
Centre for Research in Evidence-Based Practice
Faculty of Health Sciences and Medicine, Bond University, Australia

Follow-up Visit Schedule

	Initial visit date	Scheduled visit dates	Actual visit dates	Notes
Initial visit (Day–0)				
Visit – 1 (Day – 3)				
Visit – 2 (Day – 7)				
Visit – 3 (Month – 1)				
Visit – 4 (Month – 4)				

Please always bring this card to every your follow-up visit to the Hospital

Phone numbers of Hospitals and Call-centre OPAL Study

Dr. Cipto Mangunkusumo Hospital
Jl. Diponegoro No.71, Central Jakarta
Operator : 1500135

Persahabatan Hospital
Jl. Persahabatan Raya No.1, East Jakarta
Operator : 021 489 1708 Ext. 285
ENT Clinic : Ext. 230
Paediatric Clinic : Ext. 283
Emergency Instalation : Ext. 499

Gatot Soebroto Army Hospital
Jl. Dr Abdul Rahman Saleh No.24, Senen, Central Jakarta
Operator : 021 344 1008, 021 384 0702
ENT Clinic : Ext. 2057
Paediatric Clinic : Ext. 2535
Emergency Instalation : Ext. 2121

Jakarta Islamic Hospital Cempaka Putih
Jl. Cempaka Putih Tengah I No. 1, Central Jakarta
Operator : 021 425 0451, 021 428 01567 Ext. 0
Outpatient Registration : Ext. 2
Emergency Instalation : Ext. 1

Proklamasi ENT Hospital
Jl. Proklamasi No.43, Central Jakarta
Operator : 021 390 0002, 021 392 4891 Ext. 0, 101, 227, 229
ENT Clinic : Ext. 100, 236 244
Emergency Instalation : Ext. 235

Antam Medika Hospital Pulogadung
Jl. Raya Pemuda No. 1A, Pulogadung, East Jakarta
Operator : 021 806 14 888
ENT Clinic : Ext. 1027
Paediatric Clinic : Ext. 1019
Emergency Instalation : Ext. 1045

24-Call Centre OPAL Study
Dr. Respati W. Ranakusuma, Sp.THT-KL : 08111 012 185

ORAL PREDNISOLONE FOR ACUTE OTITIS MEDIA IN CHILDREN (OPAL STUDY)

Instruction for using Prednisolone

We copied cited and copied the information on the leaflet from:
Medicine for children – information for parents and carers: prednisolone for asthma.
http://www.medicinesforchildren.org.uk/prednisolone-asthma

This leaflet has been written for parents and carers about how to use this medication in children. This information may differ from that provided by the pharmaceutical company, because their information is usually aimed at adult patients. Please read this leaflet carefully.

Name of drug

Lupred tablet contains of prednisolone.

When should I give prednisolone?

Prednisolone is usually given **once** each day, usually in the morning. Give the medicine at about the same time each day so that this becomes part of your child's daily routine, which will help you to remember.

How much should I give?

Your doctor will work out the amount (the dose) that is right for your child. It is important that you follow your doctor's instructions about how much to give.

How should I give it?

The pharmacist will prepare the prednisolone tablets by crushing the tablets, mixing it with the sweetener, and packing them in a daily paper-pack for your child.

You can mix it with a small amount of soft food such as yogurt, honey, or jam, or give a glass of milk or juice. Make sure your child swallows it straight away, without chewing.

When should the medicine start working?

Prednisolone usually takes 4–6 hours to have its full effect.

What if my child is sick (vomits)?

If your child is sick less than 30 minutes after having a dose of prednisolone, give them the same dose again.

If your child is sick more than 30 minutes after having a dose of prednisolone, you do not need to give them another dose. Wait until the next normal dose.

If your child is sick again, please contact us.

What if I forget to give it?

You can give your child the missed dose as soon as you remember on the same day. If you remember after they have gone to bed, do not give them the missed dose. Give the next dose in the morning as usual. Never give a double dose of prednisolone

What if I give too much?

It can be dangerous to give too much prednisolone. If you think you may have given your child too much prednisolone, contact us immediately.

Are there any possible side-effects?

We use medicines to make our children better, but sometimes they have other effects that we don't want (side-effects). It is unlikely that your child will have side-effects if they only take prednisolone for a few days. They are more likely to get side-effects if they are on a high dose, have extra doses or take prednisolone for a long time.

Side effects that you must do something about

- If your child has bad stomach pain or repeated vomiting (being sick), contact us straight away. This may be due to an ulcer or inflammation of the pancreas
- If your child develops a rash or severe/unexplained bruising, contact us straight away, as there may be a problem with your child's blood
- If your child has eye pain or changes in their vision, contact us straight away

CHECK THE COMPLETION OF STUDY DOCUMENTS
(by Appointed Nurse)

CONFIDENTIAL STUDY DOCUMENT BINDER

- FORM01. Study Recruitment Log Book
- CRF01. Consent Form
- CRF02. Study Registration Form
- CRF08. Randomisation Form

CASE REPORT FORM BINDER

- CRF03. Eligibility Form
- CRF04. Baseline Information Form
- CRF05. Outcome Form
- CRF10. Serious Adverse Event Reporting Form
- CRF11. Feedback Form
- FORM7. Guideline of Antibiotics for AOM
- FORM08. Prednisolone Dose for OPAL Study

COMPLETED AND NON-PARTICIPATING SUBJECT BINDER

- FORM05. Recapitulation of Completed Case Report Form
- FORM06. Recapitulation of Non-Participating Subject Form

PHARMACY

PREPARATION OF STUDY MEDICATION

dr. Respati W. Ranakusuma, SpTHT-KL
Clinical Epidemiology & Evidence-Based Medicine Unit, Dr. Cipto Mangunkusumo Hospital – Faculty of Medicine Universitas Indonesia
Oral Prednisolone for acute otitis media in children: a pilot pragmatic, randomised, open-label, single-blind, controlled study (OPAL Study)

Date ____________________

CRF07. Prescription for OPAL study medication

Prednisolone doses:

- Aged 6 months to < 2 years old = 10 mg per day
- Aged 2 years to < 6 years old = 20 mg per day
- Aged 6 years to 12 years old = 30 mg per day

Registration ID :

Name : ____________________

Age : ______________ months / year(s) *[write and circle your answer]*

Study medication dose : ________ mg per day = ________ tablets per day

R/ OPAL study medication tablet ……..

Sach lact add

m.f. pulveres dtd No. V

ʃ 1 dd 1 pc (before 9 am)

____

(sign here)

DISPENSING THE STUDY MEDICATION

dr. Respati W. Ranakusuma, SpTHT-KL
Clinical Epidemiology & Evidence-Based Medicine (CEEBM) Unit, Dr. Cipto Mangunkusumo Hospital – Faculty of Medicine Universitas Indonesia
Oral prednisolone for acute otitis media in children: a pilot pragmatic, randomised, open-label, single-blind, controlled study (OPAL Study)

Pharmacy ID : |___|___|___|___| Hospital ID : |___|___|___|

FORM03 – STUDY MEDICATION DISPENSING FORM (FOR PHARMACY)

Randomisation/ Registration ID	Date dispensed	Dose (mg/day)	Number of doses (day)	Initial dose (5 days) or additional dose

Lupred ® 5

Prednisolone 5 mg

TABLET

COMPOSITION
Each tablet contains:
Prednisolone 5 mg

PHARMACOLOGY
Prednisolone is a systemic corticosteroid with glucocorticoid and anti-inflammatory potencies. The mechanism of action of corticosteroids is thought to be by control of protein synthesis. Corticosteroids react with receptor proteins in the cytoplasm of sensitive cells in many tissues to form a steroid-receptor complex.

INDICATION
Allergic reaction, inflammation and other diseases that require glucocorticoid treatment, such as rheumatoid arthritis, collagen diseases, and dermatology disorders.

DOSAGE AND INSTRUCTION
Adults: 1 – 4 tablets per day or according to the doctor's instruction.
The dosage reduces gradually until reach the lowest effective dose.

PRECAUTION
- Avoid the abrupt discontinuation in a long-term use
- Use with caution in paediatric patients who are still in the growing process
- Not recommended for pregnant and breast-feeding women
- Prolonged use of corticosteroids may produce posterior subcapsular cataracts, glaucoma with possible damage to the optic nerves, and may enhance the establishment of secondary ocular infections due to fungi or viruses
- Risk of secondary adrenocortical insufficiency could be reduced by gradual reduction of dosage
- Use with caution in patients with diabetes mellitus because it can increase the gluconeogenesis and reduce the sensitivity to insulin
- Use with caution in patients with hypothyroidism because it can enhance the effect of corticosteroids
- Use with caution in patients with heart failure, infection diseases, chronic renal failure, and elderly

ADVERSE EFFECTS
- Water balance and electrolytes disturbance: Natrium retention, excretion of potassium, hypokalaemic alkalosis, hypertension, and congestive heart failure
- Musculoskeletal: Muscle weakness, steroid-induced myopathy, osteoporosis, vertebral compression fractures and pathologic fractures of long bones
- Gastrointestinal: Peptic ulceration with haemorrhage and perforation, pancreatitis, abdominal distension and ulcerative esophagitis
- Dermatological: Impaired wound healing, thinning of the skin, facial plethora, increased sweating
- Neurological: seizures, intracranial hypertension with papilloedema (cerebral pseudotumour), vertigo, headache

FAHRENHEIT
PT. PRATAPA NIRMALA
I001404-PG-1043

FOLLOW-UP VISITS

35

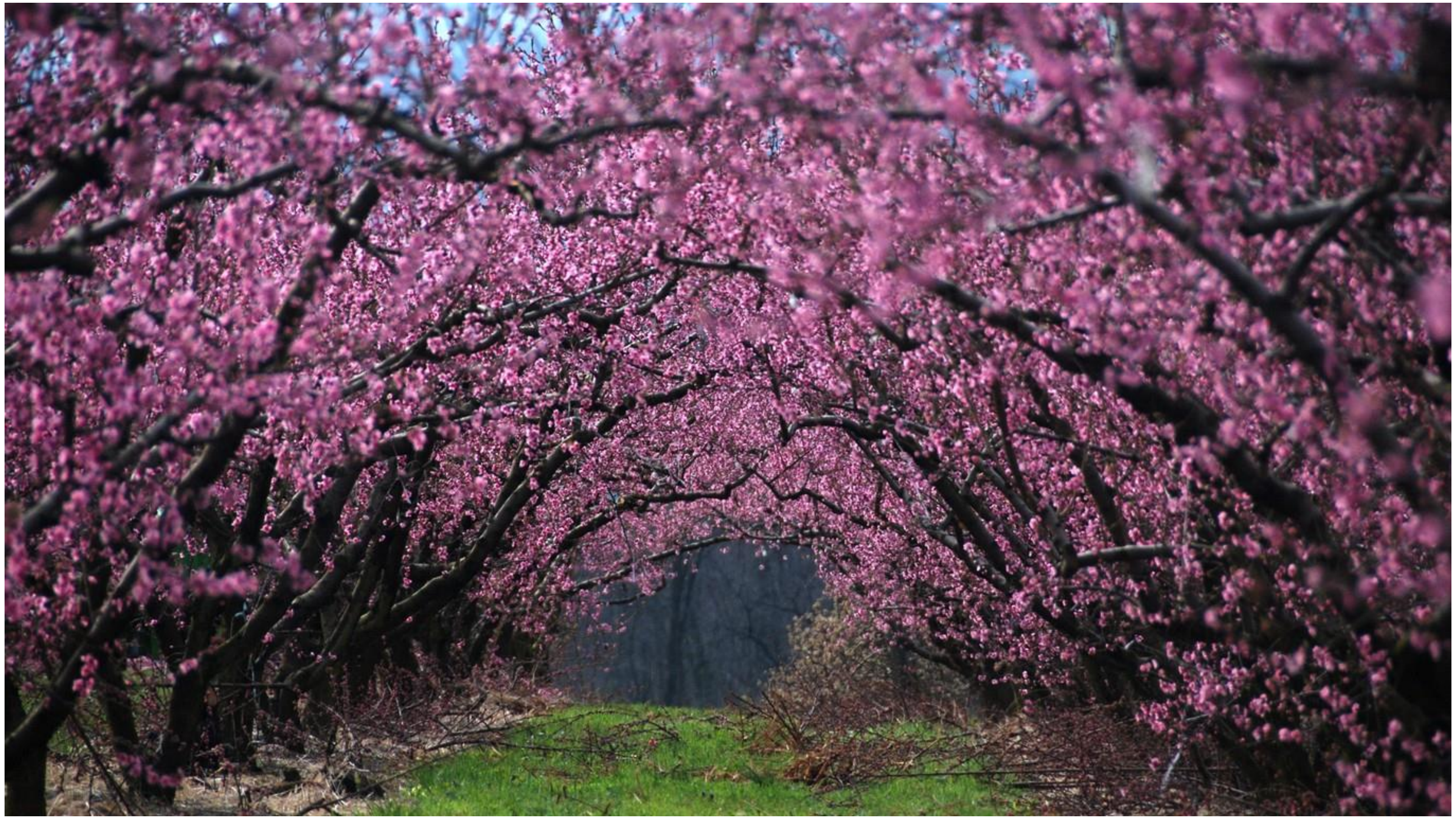

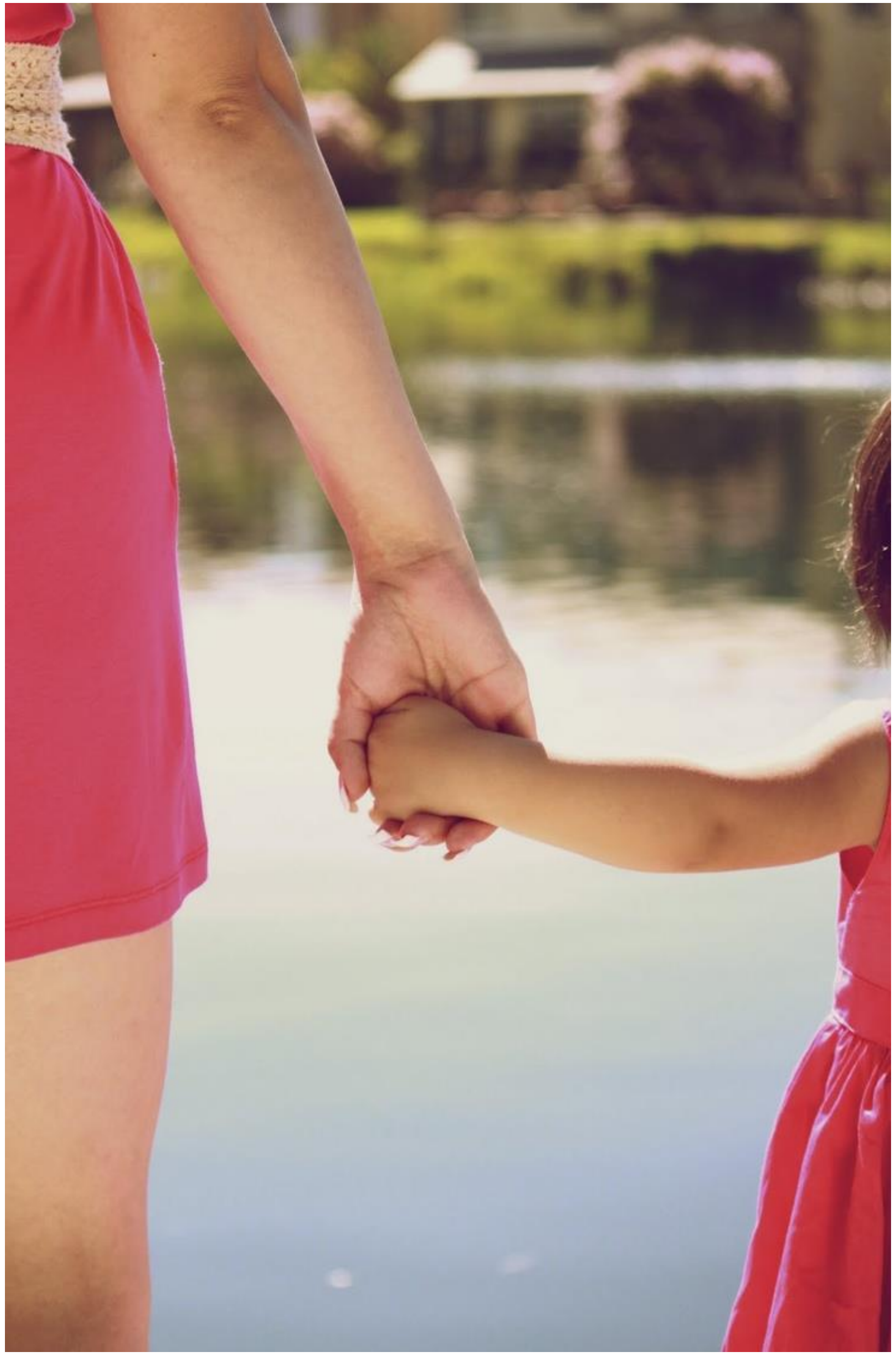

Follow-up timeline

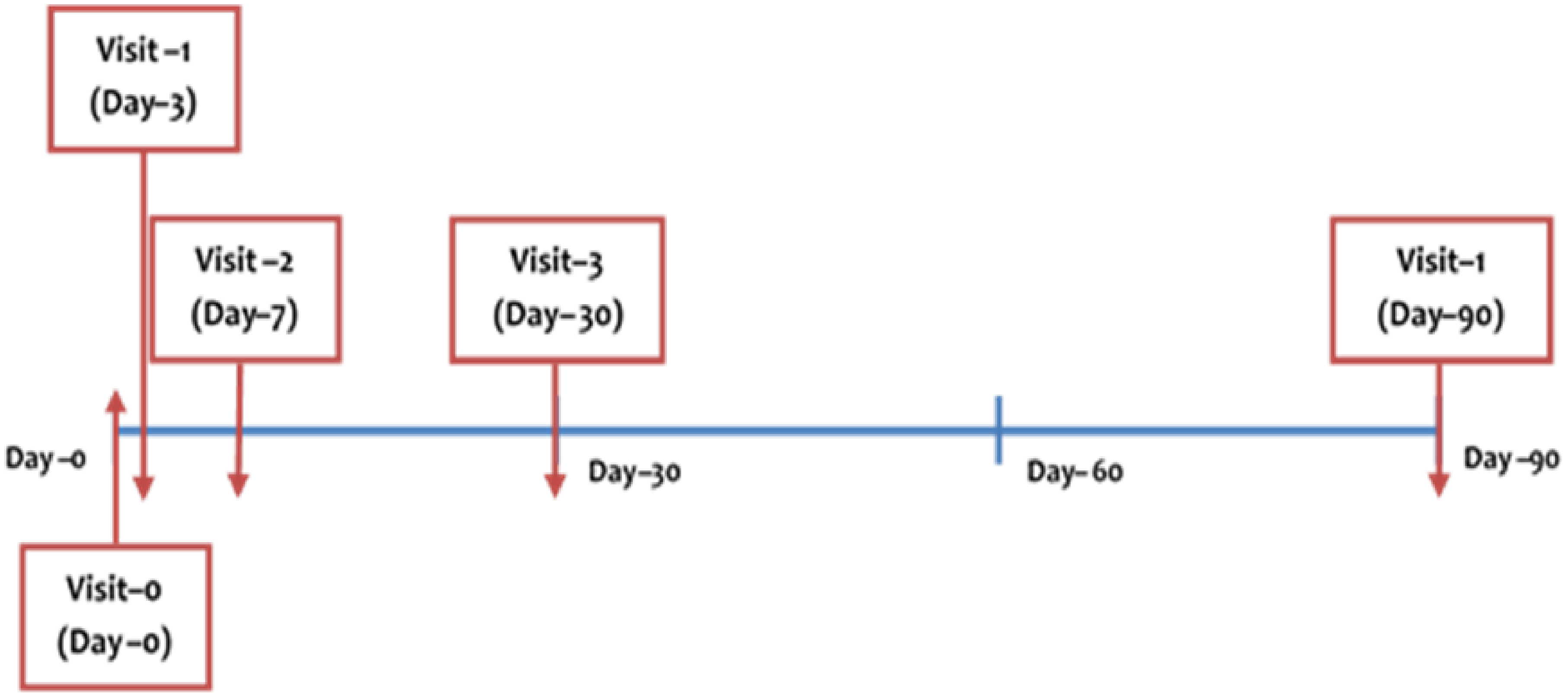

Research team will send reminder text messages to remind the parents:

1. Give the study medication regularly to their children
2. Complete the Symptom Diary regularly
3. Come to the hospital for follow-up visits

INITIAL / BASELINE VISIT

Nurse
[Nurse station]

- Identification of paediatric patients with AOM symptoms who come to the clinic using **FORM01. Study recruitment log book**
- General examination (weight, height, temperature, blood pressure)
- Inform the appointed nurse or prepare the study binders
- Provide **CRF01. Information sheet and consent form** and **CRF02. Study registration form** to be read and completed while waiting for consultation

Physician
[Consultation room]

- Identify the eligibility to the study using **CRF03. Eligibility form**
- Provide information of the study and obtain the consent from the parents to the study using **CRF01. Information sheet and consent form**
- Stratify based AOM severity using **CRF03. Eligibility form**
- Data collection (interview for clinical history and ENT, otoscopic and tympanometry examination) using **CRF04. Baseline information form** and **CRF05. Outcome form**
- Inform and teach the parents in **completing CRF06. Symptom diary**
- Prescribe study medication using **CRF07. Prescription of study medication** and other medications

Appointed Nurse

- Conduct randomisation using **CRF08. Randomisation form** by accessing the randomisation system website or calling the randomisation call-centre
- Hand over **CRF07. Prescription of study medication** for those who were allocated to the prednisolone group
- Complete **CRF09. Follow-up visit card** by writing scheduled follow-up visit for the next three months
- Provide information of prednisolone use for those who were allocated to the prednisolone group and provide **FORM09. Instruction of prednisolone use for parents**
- Confirm that the ability of the parents in completing **CRF06. Symptom diary**
- Remind the parents to keep the information of intervention allocation confidential from the physician and audiologist
- Provide the transportation reimbursement and study souvenirs
- Compile and check the completion of all study documents, including **CRF02. Study registration form** and store them in the binders
- Secure the binders in a locked filing cabinet

Pharmacy

- Prepare and dispense the study medication, including the sweetener syrup
- Record the dispensing process using **FORM03. Study medication dispensing form**

Audiologist / Trained staff
[Tympanometry room/corner]

- Tympanometry examination and record the results on **CRF05. Outcome form**. The Physician then will interpret the result

Follow-up Visit

Nurse
[Nurse station]

- Identify the patients as a study subject in OPAL study
- Perform general examination
- Report this to the Appointed Nurse with the results of general examination

Appointed Nurse

- Prepare the study documents in Case report form binder and copy the general examination results to **CRF05. Outcome form**
- Identify side effects by interview and check **CRF06. Symptom diary**. These will be reported to the physician without acknowledging him about the intervention allocation, if possible
- Collect the **First mini-booklet of symptom diary** and check its completion and the adherence in taking the study medication, as well as check the left-over study medication
- Report this subject to physician

Physician
[Consultation room]

- Identify any complications, symptoms, side effects, conduct ENT and otoscopic examination
- Record the examination results on **CRF05. Outcome form** or **CRF10. Serious adverse events reporting form** for any serious side effects
- Interpret the tympanometry results

Audiologist
[Tympanometry room/corner]

- Tympanometry examination and record its results on **CRF05. Outcome form**

Appointed nurse

- Ask the Physician for prescription of study medication, if needed
- Complete **CRF09. Follow-up visit card** and remind the parents for the scheduled next visit
- Provide the transportation reimbursement and study souvenirs
- Compile and check the completion of all study documents and store them in the binders
- Secure the binders in a locked filing cabinet

THE PRINCIPLES OF GOOD CLINICAL PRACTICE

39

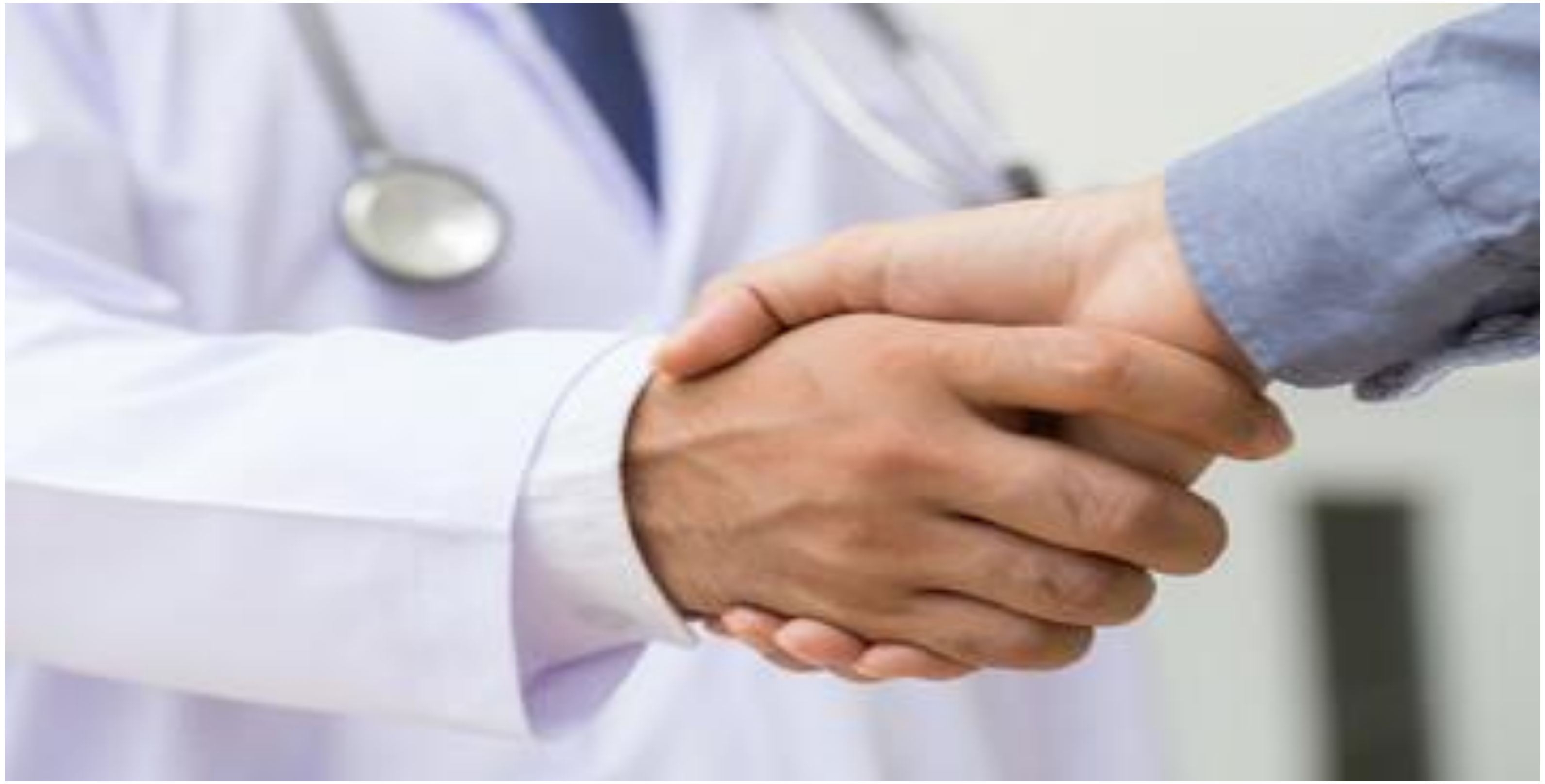

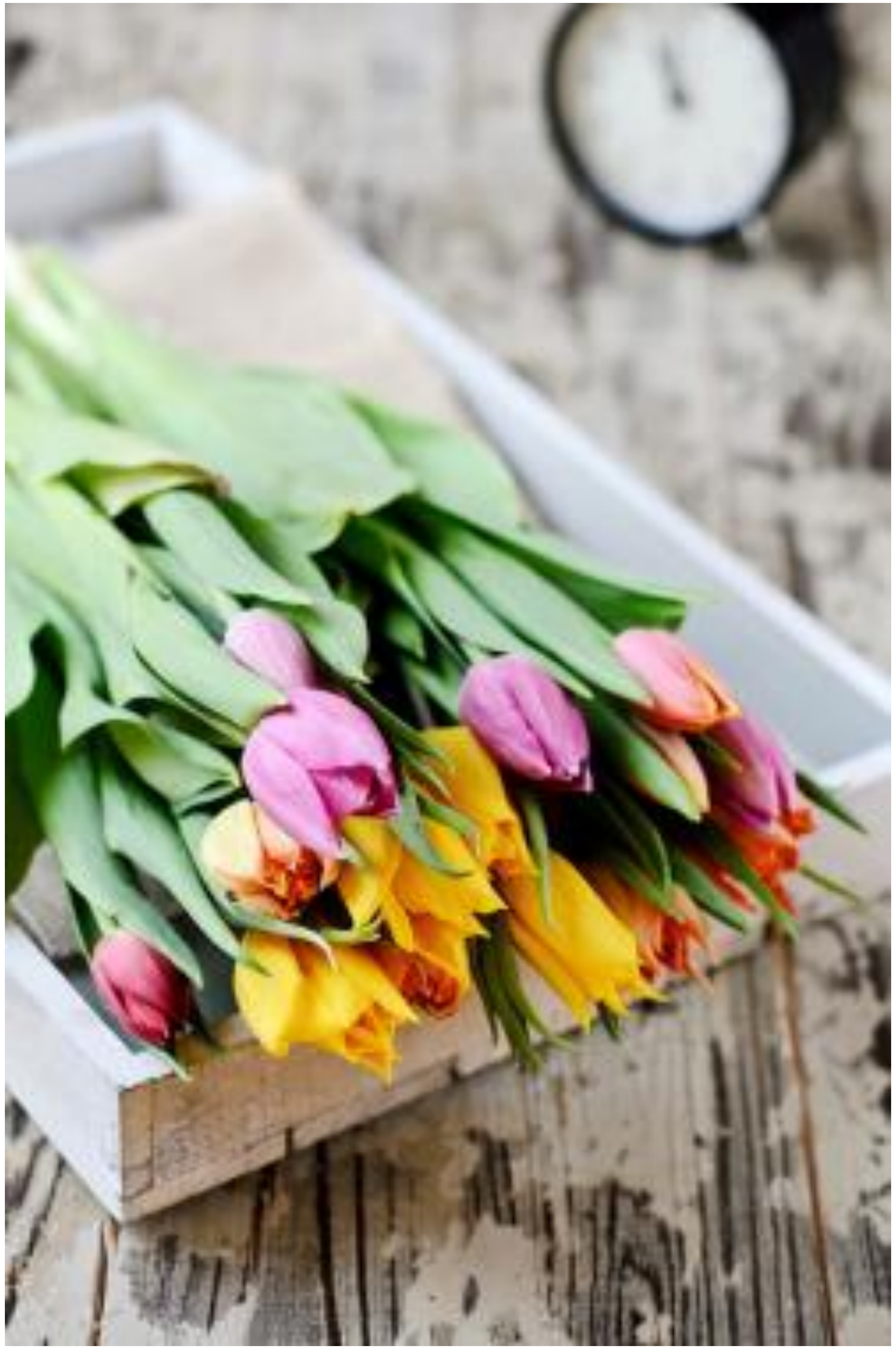

Good Clinical Practice (GCP)

Good Clinical Practice (GCP) is an international ethical and scientific quality standard for designing, conducting, recording and reporting trials that involve the participation of human subjects.

Compliance with this standard provides public assurance that the rights, safety and well-being of trial subjects are protected, consistent with the principles that have their origin in the Declaration of Helsinki, and that the clinical trial data are credible.

The principles of GCP (1)

1. Clinical trials should be conducted **in accordance with the ethical principles that have their origin in the Declaration of Helsinki, and that are consistent with GCP and the applicable regulatory requirement(s).**

2. Before a trial is initiated, **foreseeable risks and inconveniences should be weighed against the anticipated benefit for the individual trial subject and society**. A trial should be initiated and continued only if the anticipated benefits justify the risks.

3. **The rights, safety, and well-being of the trial subjects are the most important considerations** and should prevail over interests of science and society.

4. **The available nonclinical and clinical information on an investigational product should be adequate** to support the proposed clinical trial.

5. Clinical trials should be **scientifically sound, and described in a clear, detailed protocol**.

6. A trial should be conducted **in compliance with the protocol that has received prior institutional review board (IRB)/independent ethics committee (IEC) approval/favourable opinion**

The principle of GCP (2)

7. The medical care given to, and medical decisions made on behalf of, subjects should always be **the responsibility of a qualified physician** or, when appropriate, of a qualified dentist.

8. Each **individual involved in conducting a trial should be qualified by education, training, and experience** to perform his or her respective task(s).

9. Freely given informed consent should be obtained from every subject prior to clinical trial participation.

10. All **clinical trial information should be recorded, handled, and stored in a way that allows its accurate reporting, interpretation and verification**.

11. The confidentiality of records that could identify subjects should be protected, respecting the privacy and confidentiality rules in accordance with the applicable regulatory requirement(s).

12. Investigational products should be manufactured, handled, and stored in accordance with applicable good manufacturing practice (GMP). They should be used in accordance with the approved protocol.

13. Systems with procedures that assure the quality of every aspect of the trial should be implemented.

Essential terms in GCP (1)

- Protocol
- Randomisation
- Blinding
- Subject / Trial subject
- Vulnerable subjects
- Investigational product
- Investigator
- Sub-investigator
- Informed consent
- Impartial witness
- Subject identification code
- Case report form (CRF)
- Investigator's brochure
- Source documents
- Source data
- Adverse event (AE)
- Adverse drug reaction (ADR)
- Serious adverse drug reaction (serious ADR)
- Unexpected adverse dug reaction

SETTING-UP THE STUDY SITES

44

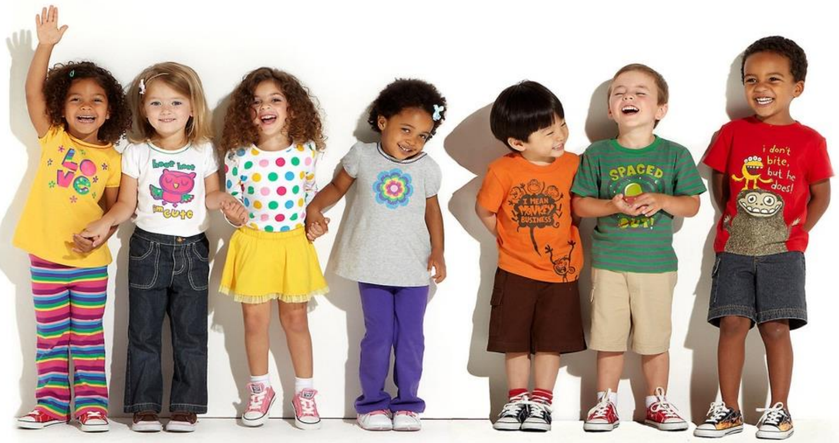

Rooms and equipment for the study (1)

1) Nurse station

2) Consultation room

3) Audiology room/corner

4) Private room for randomisation

5) Pharmacy

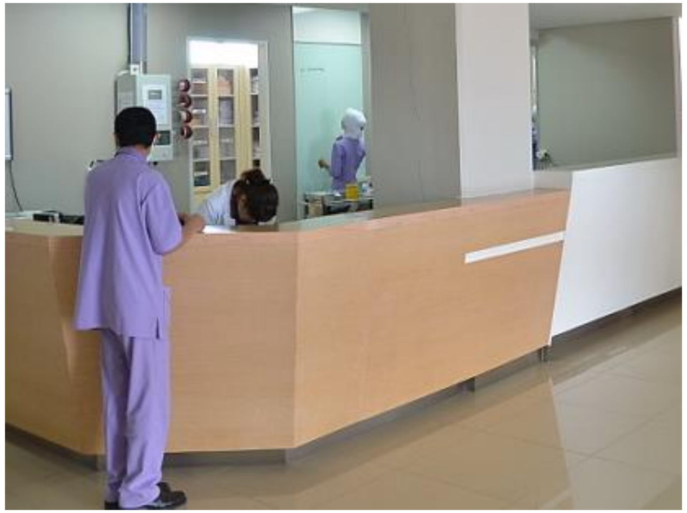

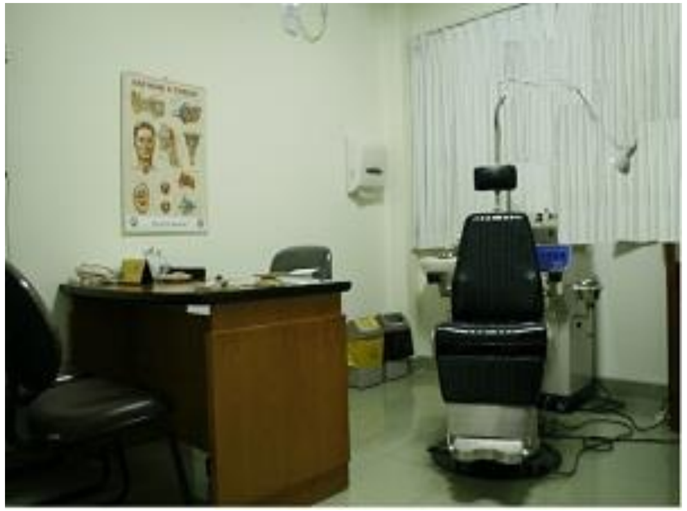

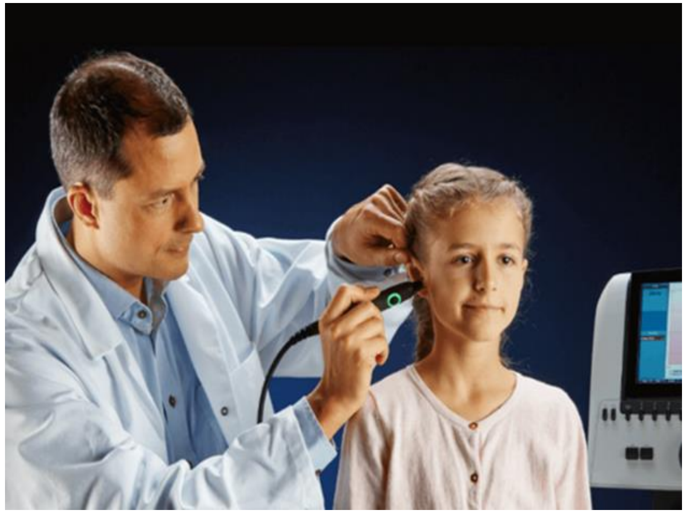

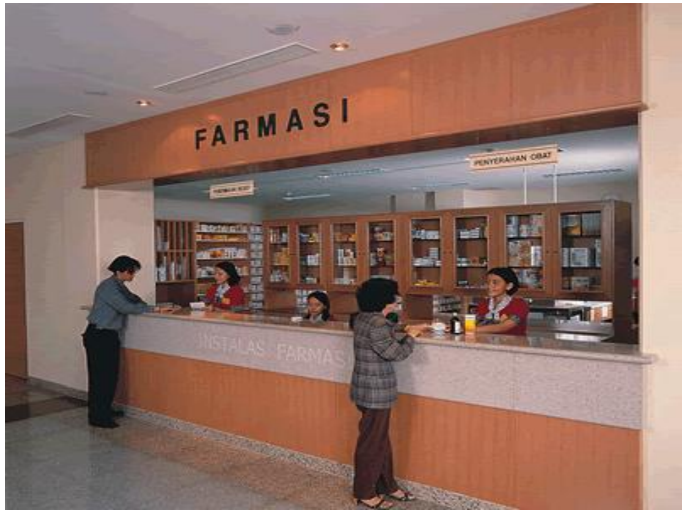

Rooms and equipment for the study (2)

1) Weight scale
2) Measuring tape
3) Thermometer
4) Paediatric tensimeter
5) Headlamp or penlight
6) ENT tools
7) Tympanometry
8) Scanner/copy machine
9) Smart phone or PC with internet connection
10) Filing cabinet with lock

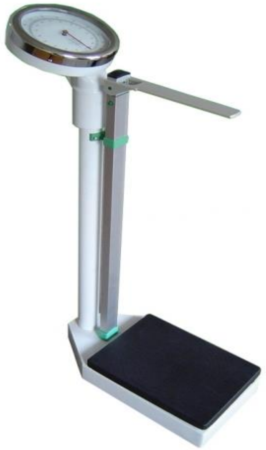

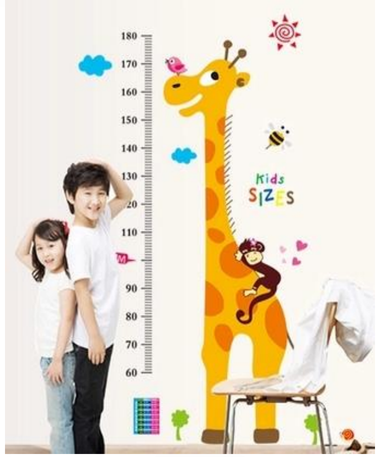

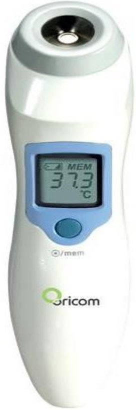

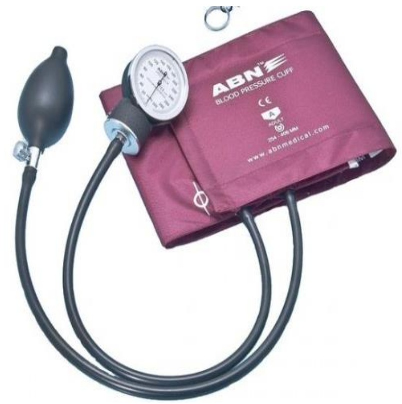

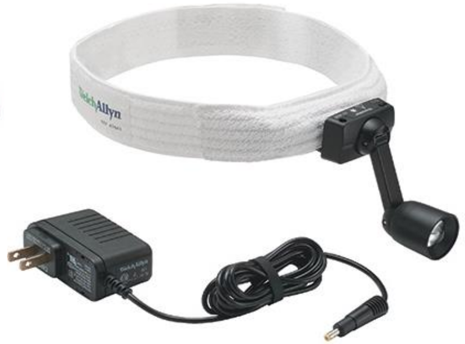

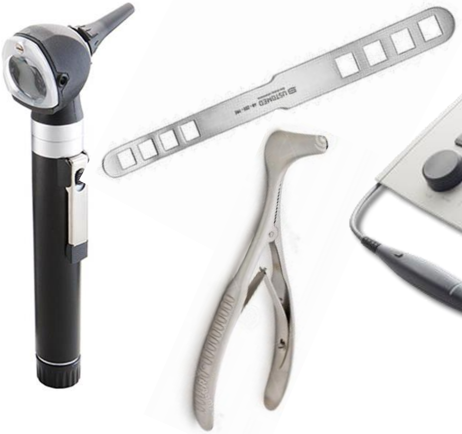

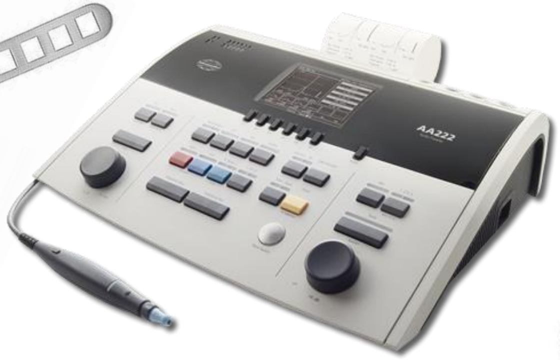

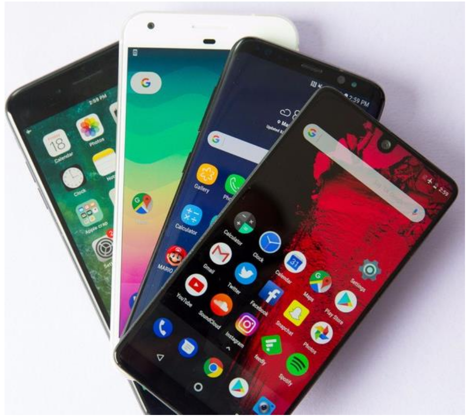

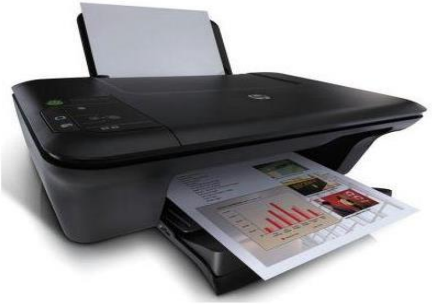

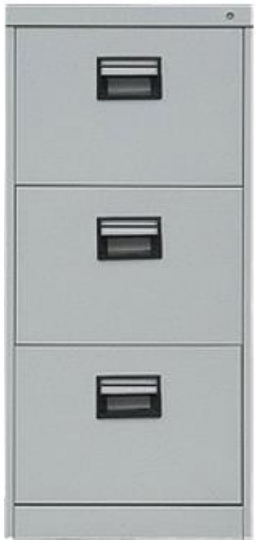

THE ASSESSMENT OF ADVERSE EFFECTS

47

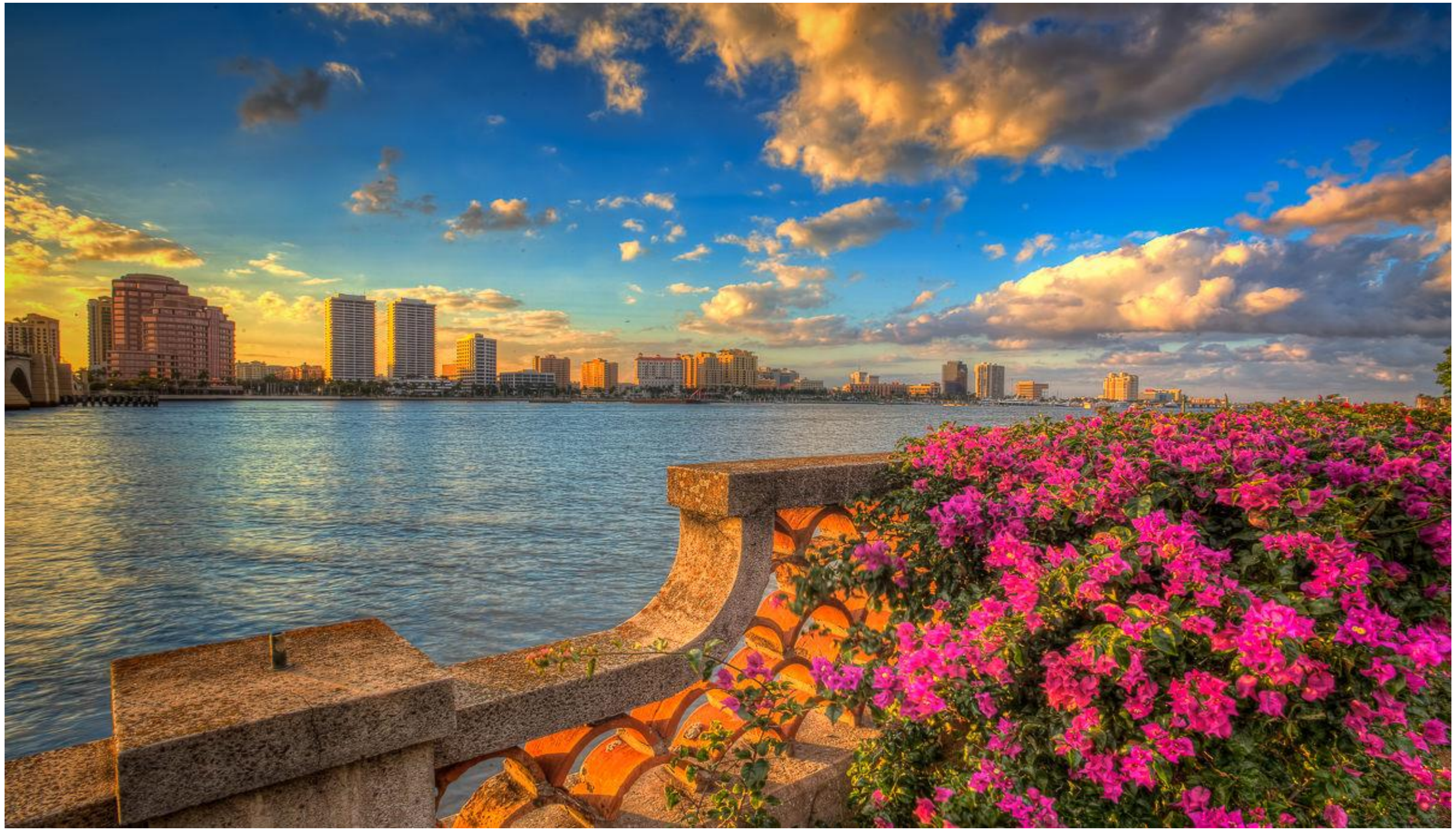

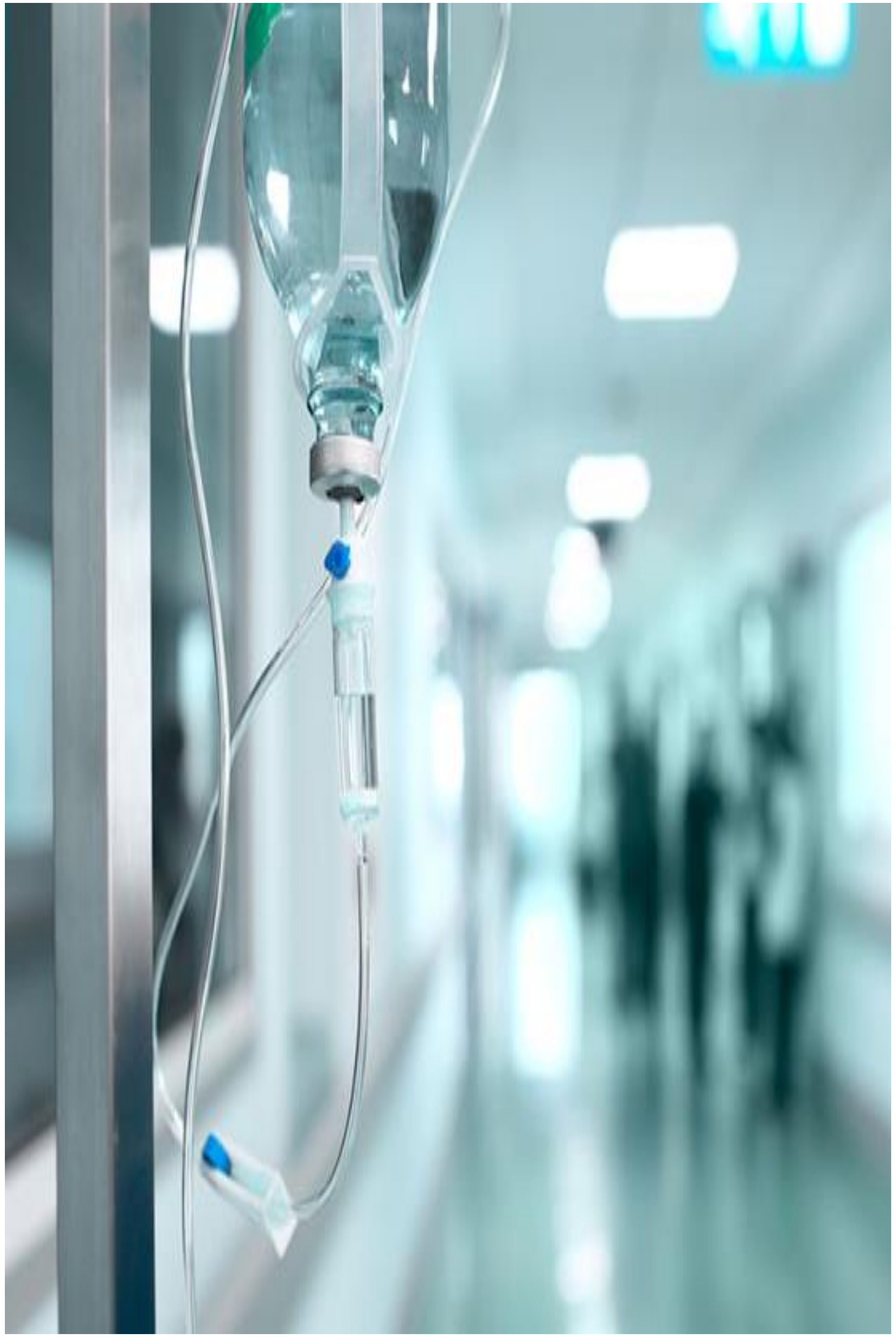

Adverse Effects

CRF05 – OUTCOME FORM

14 Side effects

Does your child have these complaints after taking the medicine

14.1 Increased appetite	O Yes	O No	14.8 Drowsiness	O Yes	O No
14.2 Increased urine amount	O Yes	O No	14.9 Anxiety/distractibility/mood swing	O Yes	O No
14.3 Weight gain	O Yes	O No	14.10 Headache	O Yes	O No
14.4 Gastritis/abdominal pain	O Yes	O No	14.11 Skin rash or diaper rash	O Yes	O No
14.5 Nausea	O Yes	O No	14.12 Candidiasis	O Yes	O No
14.6 Vomiting	O Yes	O No	14.13 Dry mouth / throat irritation	O Yes	O No
14.7 Diarrhea	O Yes	O No	14.14 Sleep disturbance	O Yes	O No

Others: ____________________

Did you bring your child to doctor (clinic or outpatient)?	O Yes	O No	Reason: ________ Medicine prescribed: ________
Has your child has been admitted to hospital?	O Yes	O No	Reason: ________ Medicine prescribed: ________

Regarding the side effects, your action is/are (you may answer more than one):

- O Discontinuation of the study drug (prednisolone)
- O Continuation of the study drug
- O Discontinuation of other concomitant drugs as follows:
  1. ________ 3. ________
  2. ________ 4. ________

The treatment you prescribed for the management of side effects

1. ________; Dose ________; Frequency ______ / day
2. ________; Dose ________; Frequency ______ / day
3. ________; Dose ________; Frequency ______ / day
4. ________; Dose ________; Frequency ______ / day

Does this child require specific or additional tests or examination?

- O No
- O Yes. Please specify with the results:
  1. ________
  2. ________
  3. ________

REGISTRATION ID ________

CRF10. SERIOUS ADVERSE EVENTS REPORTING FORM

**SUBJECT INFORMATION**			
Weight (kg)	|__|__|, |__|__| kg		
List any relevant tests, laboratory data, history, including pre-existing medical conditions	________		
Any concomitant medication	________		
**ADVERSE EVENT**			
Report type	☐ Initial report	☐ Follow-up	☐ Final
Reason for reporting	☐ Requires or prolongs hospitalization<br>☐ Permanently disabling or incapacitating<br>☐ Overdose<br>☐ Other (please specify) ________	☐ Congenital anomaly<br>☐ Life threatening<br>☐ Death<br>Date of death ________<br>Cause of death ________	
**SUSPECTED DRUG**			
Name of suspected drug	________	Generic name	________
Dose details	________	Name of manufacturer	________
Date of occurrence	|__|__| - |__|__| - |__|__|__|__| *(date – month – year)*		
Duration of event	|__|__| month(s) |__|__| day(s)		
Starting date of medication	|__|__| - |__|__| - |__|__|__|__| *(date – month – year)*		
Route of administration	________	Indication	________
Discontinuation of drug because of event	☐ No	☐ Yes	Dated (date / month / year) : ________
If stopped/lowered dose, did the event resolve after this?	☐ Yes	☐ No	☐ N/A
If reintroduced did the event reappear?	☐ Yes	☐ No	☐ N/A
Outcomes	☐ Recovered<br>☐ Change in SAE	☐ Recovered with sequelae<br>☐ Patient died	☐ Continuing<br>☐ Unknown
Severity	☐ Mild	☐ Moderate	☐ Severe
Action taken with study drug	☐ None<br>☐ Dose temporarily reduced	☐ Dose reduced<br>☐ Discontinued temporarily	☐ Discontinued
Other action*	☐ None	☐ Treated with medication	☐ Other

FEEDBACK

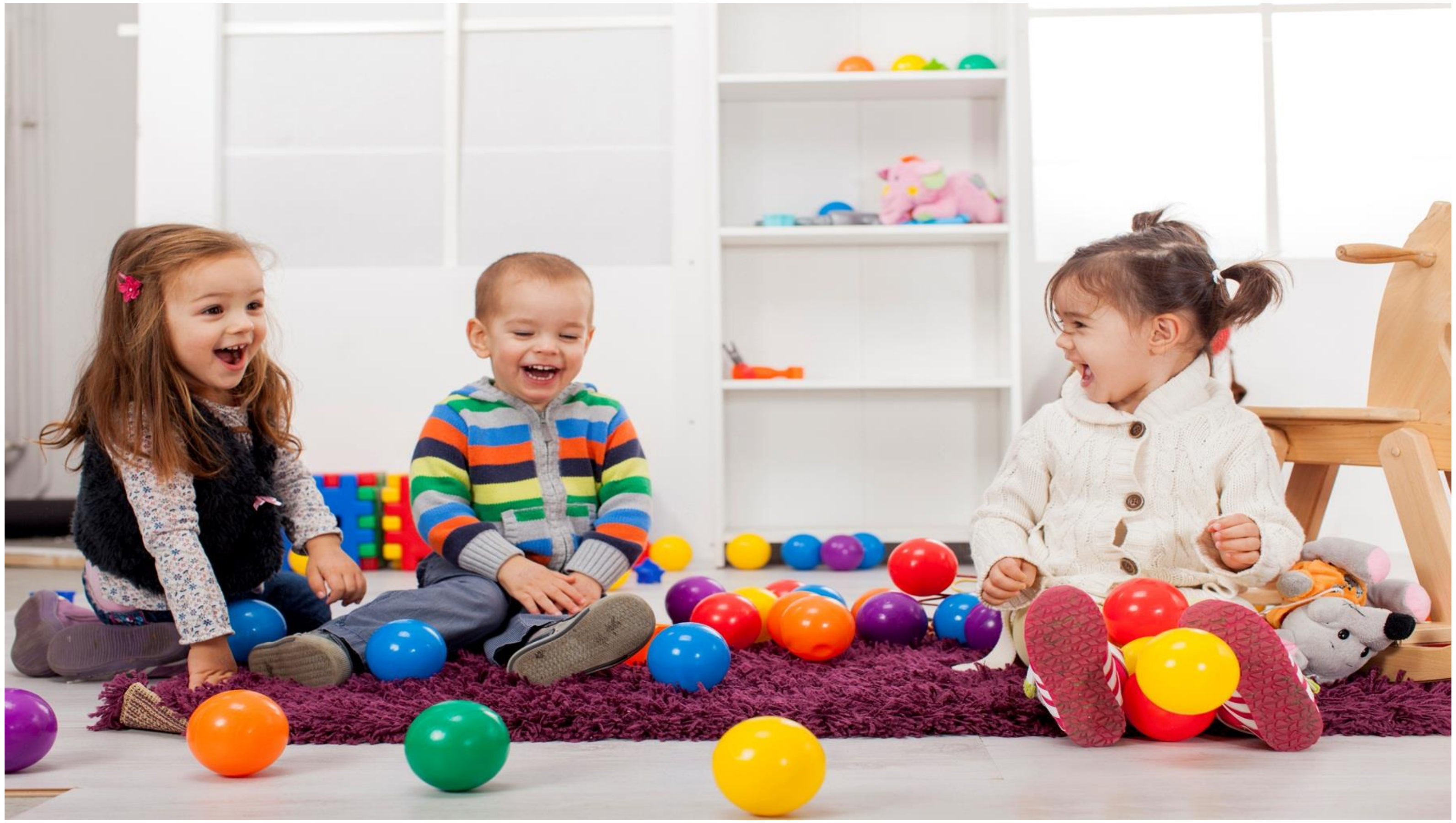

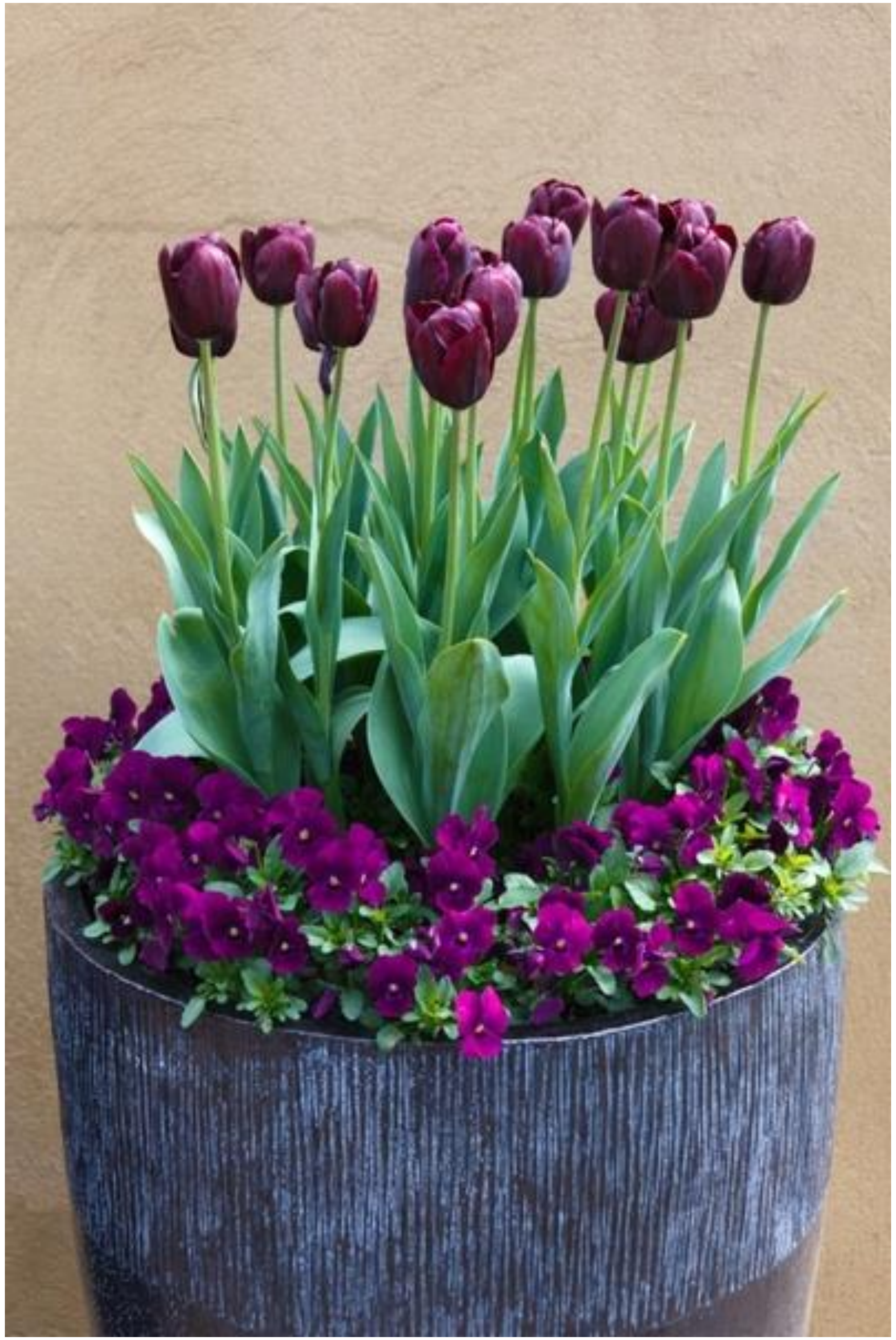

Feedback

Physicians

CRF11 – FEEDBACK FORM (for Physician only)

	Questions	Please place a checkmark (√) inthe box corresponding to your answer				
Information Sheet and Consent Form	How do you rate the process of providing patient information and informed consent to your patient?	☐ Very easy	☐ Easy	☐ Neutral	☐ Difficult	☐ Very difficult
	If your answer 'difficult' or 'very difficult', please place a checkmark (V) in the box corresponding to or write you reason(s). You may choose more than one	☐ It was too difficult to explain this to my patient/parent<br>☐ Time consuming<br>☐ There was too much information to explain<br>☐ I was not sure that my patient understood<br>☐ Others : ____				
Otoscopic Examination	How do rate the process of conducting an otoscopic examination to your patient?	☐ Very easy	☐ Easy	☐ Neutral	☐ Difficult	☐ Very difficult
	If your answer 'difficult' or 'very difficult', please place a checkmark (V) in the box corresponding to or write you reason(s). You may choose more than one	☐ Patient was not cooperative<br>☐ The ear canal was too narrow<br>☐ Insufficient tool (e.g. the otoscope cylinder was too large)<br>☐ Ear wax and it was too difficult to extract<br>☐ The symptoms are definitely celar showing AOM<br>☐ Others : ____				
Visual Analogue Scale (VAS)	How do you rate the process of providing related information and assisting your patient/parent to complete the visual analogue scale (VAS)?	☐ Very easy	☐ Easy	☐ Neutral	☐ Difficult	☐ Very difficult
	If your answer 'difficult' or 'very difficult', please place a checkmark (V) in the box corresponding to or write you reason(s). You may choose more than one	☐ It was too difficult to explain this to my patient/parent<br>☐ Time consuming<br>☐ I was not sure that my patient/parent understood<br>☐ My patient/parent seem not confidence with the answer<br>☐ Others : ____				
Acute Otitis Media –	How do you rate the process of providing related information and assisting your patient/parent to complete the acute otitis	☐ Very easy	☐ Easy	☐ Neutral	☐ Difficult	☐ Very difficult

Nurses

FEEDBACK FORM (for Nurses who conducts randomisation only)

	Questions	Please place a checkmark (√) inthe box corresponding to your answer				
Randomisation Process	How do you rate the randomisation process, in terms of obtaining the study ID and the allocation of the intervention (prednisolone group or control group)	☐ Very easy	☐ Easy	☐ Neutral	☐ Difficult	☐ Very difficult
	If your answer 'difficult' or 'very difficult', please place a checkmark (V) in the box corresponding to or write you reason(s). You may choose more than one.	☐ The CRF08. Randomisation form is too complicated<br>☐ The randomisation process was too confusing<br>☐ It was difficult to access the randomisation centre (randomisation website or by phone) to obtain the study ID and the allocation of the intervention<br>☐ It was difficult to explain to the patients that they were allocated to groups which reveice prednisolone or not<br>☐ Others ____				
Dispensing the Study Medication Prescription	How do rate the process of dispensing the study medication prescription and keep the intervention allocation concealed from their Physician and Audiologists?	☐ Very easy	☐ Easy	☐ Neutral	☐ Difficult	☐ Very difficult
	If your answer 'difficult' or 'very difficult', please place a checkmark (V) in the box corresponding to or write you reason(s). You may choose more than one	☐ This process was too time consuming<br>☐ I encountered difficulties when I was providing relevant information on the intervention they received<br>☐ It was difficult to ask my patients/parents to keep the information of intervention allocation confidential<br>☐ Others: ____				
The compilation and the Storage of Case report Forms	How do rate the process of the compilation and the storage of study documents and binders?	☐ Very easy	☐ Easy	☐ Neutral	☐ Difficult	☐ Very difficult
	If your answer 'difficult' or 'very difficult', please place a checkmark (V) in the box corresponding to or write you reason(s). You may choose more than one	☐ This process was too confusing<br>☐ This process was too time consuming<br>☐ It was difficult to find case report forms in the binder<br>☐ The checklist of case report forms was not helping<br>☐ Others: ____				

Audiologists

FEEDBACK FORM (for Audiologist/Trained Staff only)

	Questions	Please place a checkmark (√) inthe box corresponding to your answer				
Tympanomtry Examination and the Competion of Tympanometry Section in the Case report Form	How do you rate the process of tympanometry examination and completing the tympanometry section in CRF?	☐ Very easy	☐ Easy	☐ Neutral	☐ Difficult	☐ Very difficult
	If your answer 'difficult' or 'very difficult', please place a checkmark (V) in the box corresponding to or write you reason(s). You may choose more than one.	☐ The patients' parents seem did not understand teh reason this examination being performed<br>☐ It was difficult to conduct this examination to my patients<br>☐ The 'Tympanometry section' in CRF05. Outcome form is confusing. The provided examination components are unfamiliar or different<br>☐ It was difficult to find the 'Tympanometry section' in the CRF05. Outcome form<br>☐ It was difficult to print out the copy of tympanometry result<br>☐ There were few components of this examination not provided in the form<br>☐ Others: ____				

Feedback

Pharmacists

FEEDBACK FORM (for Pharmacists only)

	Questions	Please place a checkmark (V) inthe box corresponding to your answer				
Preparation and Dispensing teh Study Medication	How do rate the preparation and dispensing process of the study medication?	☐ Very easy	☐ Easy	☐ Neutral	☐ Difficult	☐ Very difficult
	If your answer 'difficult' or 'very difficult', please place a checkmark (V) in the box corresponding to or write you reason(s). You may choose more than one.	☐ The instruction in CRF07. Prescription was confusing<br>☐ The preparation of the study medication was too time-consuming<br>☐ I encountered difficulties when providing the information about the study medication to my patients<br>☐ Others: ____________				

Parents

FEEDBACK FORM (for Parents only)

Questions	Please place a checkmark (V) inthe box corresponding to your answer				
How do you rate the process in completing the pain scale below?<br>No Pain ——— Pain As Bad As It Could Possibly Be					
	☐ Very easy	☐ Easy	☐ Neutral	☐ Difficult	☐ Very difficult
If your answer 'difficult' or 'very difficult', please place a checkmark (V) in the box corresponding to or write you reason(s). You may choose more than one.	☐ I did not understand how to complte this scale<br>☐ I need more information from my doctor<br>☐ The provided instruction in the form was unclear<br>☐ My doctor could not provide additional information that I need<br>☐ Others : ____________				
How do you rate the process in completing the AOM-relevant symptom questionnaire below?					
	☐ Very easy	☐ Easy	☐ Neutral	☐ Difficult	☐ Very difficult
Apabila jawaban Anda 'Difficult' atau 'Very difficult', mohon berikan tanda centang di kotak yang sesuai atau berikan alasan Anda. Anda dipersilahkan untuk memilih lebih dari satu jawaban.	☐ It was difficult to understand the question(s)<br>☐ The options of answers were confusing<br>☐ The provided instruction in the form was unclear<br>☐ The question(s) was not suitable for my child, therefore I did not know how to answer the question(s): question no ____ ; ____ ; ____<br>☐ I do not know how to complete the questionnaire				

12 We are interest finding out how your child has been doing. For each question, please place a checkmark (V) in the circle corresponding to your child's symptoms. Please answer all questions.			
12.1 Over the past 12 h, has your child been tugging, rubbing, or holding the ear(s) more than usual?	O No	O A little	O A lot
12.2 Over the past 12 h, has your child been crying more than usual?	O No	O A little	O A lot
12.3 Over the past 12 h, has your child been more irritable or fussy than usual?	O No	O A little	O A lot
12.4 Over the past 12 h, has your child been having more difficulty sleeping than usual?	O No	O A little	O A lot
12.5 Over the past 12 h, has your child been less playful or active than usual?	O No	O A little	O A lot
12.6 Over the past 12 h, has your child been eating less than usual?	O No	O A little	O A lot
12.7 Over the past 12 h, has your child been having fever or feeling warm to touch?	O No	O A little	O A lot

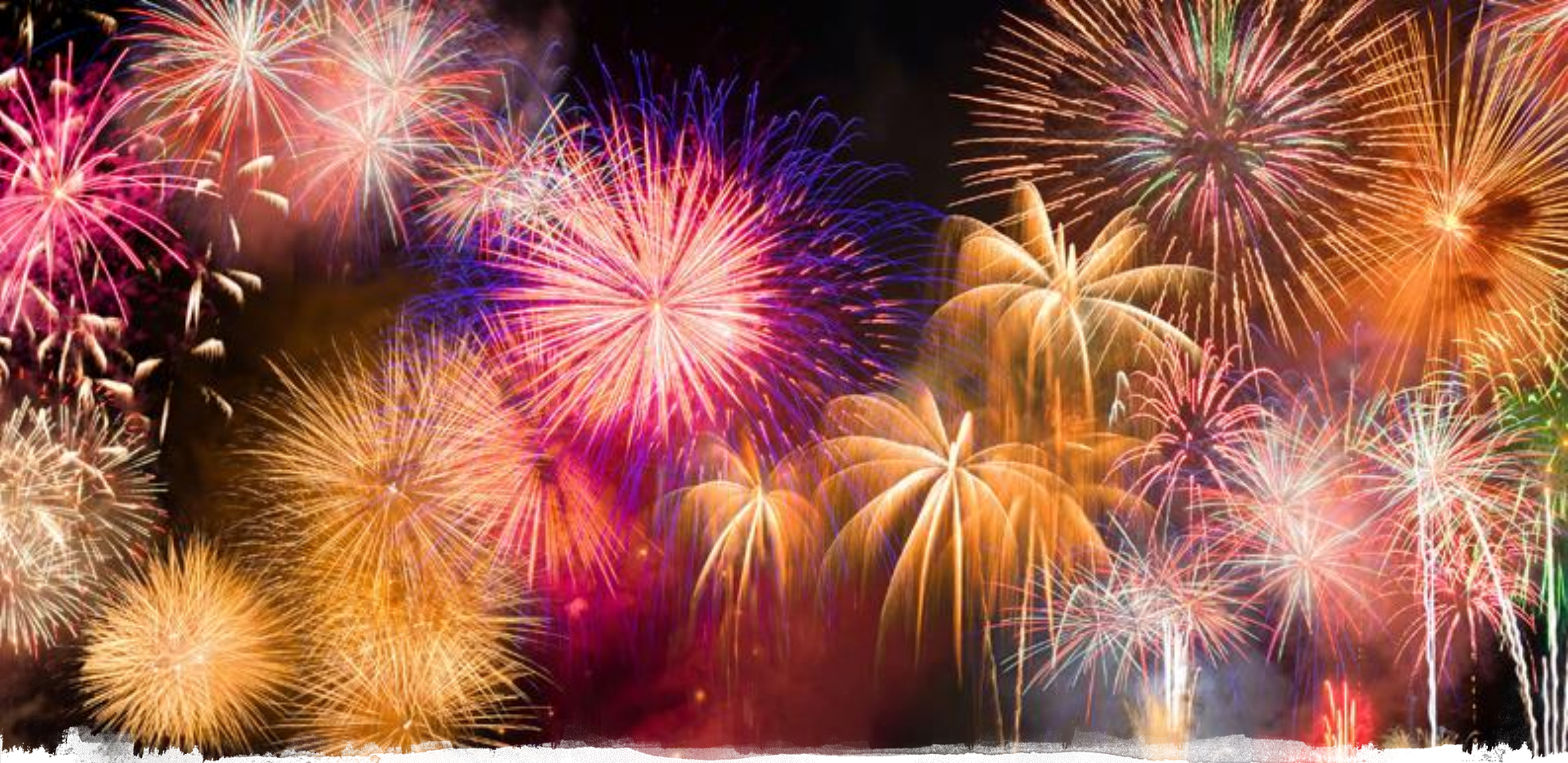

Thank you for your participation in the OPAL study.
Together, we will contribute in making Indonesian children' ears healthy!
